# *TMPRSS2-ERG* confers resistance of prostate cancer to antiandrogens

**DOI:** 10.1038/s44321-026-00423-7

**Published:** 2026-05-12

**Authors:** Arunachalam Sekar, Boobash Raj Selvadurai, Rishita Chatterjee, Lipika Pal, Aakanksha Verma, Nishanth Belugali Nataraj, Diana Drago Garcia, Suvendu Giri, Alessandro Genna, Feride Karatekin, Nitin Gupta, Deepthi Ramesh-Kumar, Mirie Zerbib, Yaron Vinik, Tamir Avioz, Eviatar Weizman, Eyal David, Alejandro A Schäffer, Yunqian Pan, Haojie Huang, Wytske M van Weerden, Eva Corey, Hazel Hunt, Andrew E Greenstein, Ronnie Blecher-Gonen, Roni Oren, Ariel Afek, Ido Amit, Sima Lev, Anson Ku, Sumeyra Kartal, Jack R Bright, Rosina T Lis, William L Dahut, Adam G Sowalsky, Eytan Ruppin, Yosef Yarden

**Affiliations:** 1 Department of Immunology and Regenerative Biology, Weizmann Institute of Science, Rehovot, Israel; 2 Cancer Data Science Laboratory, National Cancer Institute, National Institutes of Health, Bethesda, MD USA; 3 Department of Veterinary Resources, Weizmann Institute of Science, Rehovot, Israel; 4 Department of Molecular Cell Biology, Weizmann Institute of Science, Rehovot, Israel; 5 Department of Chemical and Structural Biology, Weizmann Institute of Science, Rehovot, Israel; 6 Department of Genomics Unit, Weizmann Institute of Science, Rehovot, Israel; 7 Department of Systems Immunology, Weizmann Institute of Science, Rehovot, Israel; 8 Department of Biochemistry and Molecular Biology, Mayo Clinic College of Medicine and Science, Rochester, MN USA; 9 Department of Urology, Erasmus University Medical Center, Rotterdam, The Netherlands; 10 Department of Urology, University of Washington School of Medicine, Seattle, WA USA; 11 Corcept Therapeutics, Menlo Park, CA USA; 12 Exelixis, Inc., 1851 Harbor Bay Pkwy, Alameda, CA USA; 13 Genitourinary Malignancies Branch, Center for Cancer Research, National Cancer Institute, National Institutes of Health, Bethesda, MD USA; 14 Present Address: Bugworks Research Inc, C-CAMP, NCBS Campus, Bangalore, India; 15 Present Address: The Francis Crick Institute, London, UK; 16 Present Address: National University of Singapore, Institute of Molecular and Cell Biology (IMCB), Singapore, Singapore

**Keywords:** Cancer, Urogenital System

## Abstract

Approximately 50% of prostate cancer (PCa) patients harbor fusions involving the *TMPRSS2* and *ERG* genes. Despite this, tailored therapies targeting the fused gene, *tERG*, remain undeveloped. Our study analyzed biopsy samples from two clinical trials assessing the efficacy of androgen receptor (AR) signaling inhibitors (ARSIs). The results revealed that *tERG* promotes resistance to ARSIs and is associated with elevated levels of the glucocorticoid receptor (GR). Subsequent assays showed that GR directly interacts with tERG, alleviates allosteric autoinhibition, and prevents chemotherapy-induced tERG degradation. In PCa models, either inhibiting GR or lowering cortisol levels suppressed tumor growth in tERG-positive models, but not in tERG-negative models. In addition, patient-derived fusion-positive xenografts displayed enhanced sensitivity to combined GR and AR inhibitors. Collectively, these findings highlight *TMPRSS2-ERG* as a new biomarker and propose that simultaneous inhibition of GR and AR may specifically benefit *tERG*-positive patients. However, GR stimulatory corticosteroid therapies may not be advisable for this patient subgroup.

## Synopsis

Reanalysis of clinical trials revealed that prostate cancers (PCa) harboring the fused tERG oncogene often resist antiandrogens. Resistance likely arises from direct tERG binding to the glucocorticoid receptor (GR). Co-targeting GR and AR overcomes resistance, but only in tERG-positive models.

- Data from two clinical trials (total, 95 patients) showed that expression of tERG associates with poorer response of patients with PCa to treatments with enzalutamide and abiraterone.
- Post treatment, patients display up-regulation of GR, which is normally repressed by AR.
- Through their DNA-binding domains GR and ERG form stable complexes.
- Complex formation stablizes ERG and alleviates its autoinhibition, thereby enhances its transcriptional activity towards MYC and other oncogenic pathways.
- In animal models, including patient-derived xenografts, co-inhibition of GR and AR, or reduction of cortisol levels, suppressed growth of tERG-positive xenografts, whereas fusion-negative models were unaffected.

## Abstract (web summary)

Reanalysis of clinical trials revealed that prostate cancers (PCa) harboring the fused tERG oncogene often resist antiandrogens. Resistance likely arises from direct tERG binding to the glucocorticoid receptor (GR). Co-targeting GR and AR overcomes resistance, but only in tERG-positive models.

## Graphical abstract

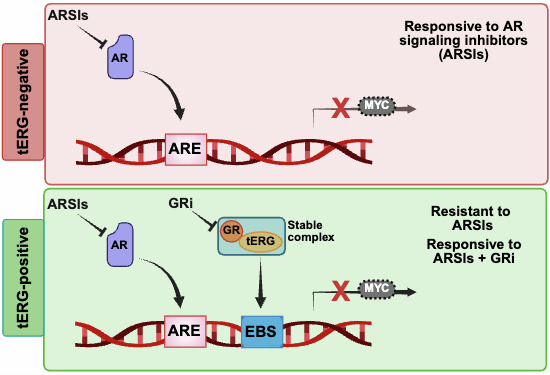

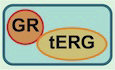

The paper explained **Problem**

Although every other patient with prostate cancer (PCa) harbors the *tERG* (*TMPRSS2-ERG)* fusion gene, so far, tERG has not been targeted for personalized anti-cancer treatments or utilized as a biomarker.

**Results**

According to our observations, patients who are positive for *tERG* are intrinsically resistant to antiandrogen therapy. This is likely due to physical complex formation between tERG and GR, which stabilizes ERG. In animal models, concurrent inhibition of both GR and AR effectively blocked *tERG*-positive prostate tumors but spared treatment from the *tERG* -negative models.

**Impact**

Future large-scale clinical trials may validate that combined treatment with AR and GR inhibitors can overcome drug resistance, but only in patients who are tERG-positive.

## Introduction

Prostate cancer (PCa) is the second most common cause of cancer-related deaths in men. The majority of tumors fall into one of seven subtypes defined by specific gene fusions or mutations (Cancer Genome Atlas Research, 2015). More than 50% of Caucasian patients with PCa harbor chromosomal rearrangements in genes encoding for specific ETS (E26 transformation-specific) family transcription factors (TFs) (Grasso et al, 2012; Paulo et al, 2012; Robinson et al, 2015; Tomlins et al, 2005). The most common rearrangement fuses the promoter region of the androgen-regulated gene *TMPRSS2* (transmembrane protease, serine 2), with the coding region of *ERG*, a member of the ETS family, which lies near *TMPRSS2* on human chromosome 21. Similar but less frequent ETS fusions engage *ETV1*, *ETV4*, or *ETV5*, which are on other chromosomes. One outcome of the *TMPRSS2-ERG (tERG)* gene fusion is androgen-inducible overexpression of the ERG protein. Notably, in normal benign prostatic tissue and benign prostatic hyperplasia *TMPRSS2* fusion genes do not form. However, *TMPRSS2-ERG* gene fusions are observed at the earliest stages of PCa development, including a subset of high-grade prostatic intraepithelial neoplasia (Perner et al, 2007). When present, the fusion gene is highly expressed in both primary and castration-resistant tumors (Robinson et al, 2015; Roudier et al, 2016). Although *tERG* is associated with high Gleason scores and the presence of *TMPRSS2-ERG* fusion marks relatively aggressive tumors, the role of *tERG* as a prognostic marker is complex.

ERG and other members of the ETS family share a well-conserved DNA-binding element, ETS, which binds with the DNA motif GGA(A/T) (Donaldson et al, 1996; Wei et al, 2010). In addition to the ETS domain, a subset of ETS proteins harbor the pointed domain (PNT), which facilitates protein-protein interactions and dimerization. tERG drives unique transcriptional programs, represses prostate epithelial differentiation genes, such as *PSA* and *SLC45A3*, and controls several downstream targets, including components of the NOTCH signaling pathway, MYC, EZH2, and WNT (Kron et al, 2017; Sun et al, 2008; Wu et al, 2013; Yu et al, 2010). Through binding with coactivators and corepressors, the transcriptional activity of ETS proteins is stringently modulated (Hollenhorst et al, 2007). For example, tERG can physically interact with BAF (SWI/SNF) complexes (Sandoval et al, 2018) to regulate several cellular processes in PCa cells, including gene expression and ERG-driven basal-to-luminal transition. In addition, the *tERG* fusion gives rise to various N-terminally truncated ERG proteins (Clark et al, 2007). The majority of the truncated fusions are resistant to degradation because they lack the degron motif for a ubiquitin ligase encoded by the *SPOP* gene, which is mutated in approximately 11% of PCa (An et al, 2015; Gan et al, 2015). There is an analogous ERG-inhibitory mechanism carried out by the ETS2 repressor factor, ERF, which undergoes inactivation in PCa by means of recurrent point mutations and focal deletions (Bose et al, 2017). Thus, hormonal control of ERG expression, competition with ERF, and avoidance of degradation offer therapeutic opportunities for targeting ERG in PCa.

The currently approved therapies for castration-resistant prostate cancer (CRPC) include both non-steroidal antiandrogens (NSAA, e.g., enzalutamide) and inhibitors of CYP17A1 (e.g., abiraterone), a cytochrome P450 enzyme that catalyzes the production of several steroid hormones, including androgens. Unfortunately, the initial positive responses to these therapies are typically followed by drug resistance and tumor relapses. Several studies have shown that activation of the glucocorticoid receptor (GR) permits resistance to enzalutamide (Arora et al, 2013; Isikbay et al, 2014; Li et al, 2017; Puhr et al, 2018; Shah et al, 2017). Both the endogenous steroid ligand of GR, cortisol, and synthetic GR ligands, such as dexamethasone (DEX), transduce their actions by binding to GR. GR activity is able to replace AR due to similar promoter sequences and downstream targets, including the anti-apoptotic mediators *DUSP1* and *SGK1*. Normally, GR expression is silenced by AR (Shah et al, 2017). Hence, prolonged inhibition of AR can result in GR upregulation, underscoring the selective pressure to maintain downstream target gene expression.

Earlier, we demonstrated that FLI1, along with other ETS family members, directly interacts with GR. Furthermore, inhibiting GR in Ewing sarcoma models—pediatric tumors driven by the *EWS-FLI1* fusion gene—led to decreased tumor growth and metastasis. Given these findings and GR’s role in resistance to AR signaling inhibitors (ARSIs) (Arora et al, 2013), we explored ETS-driven PCa. Reanalysis of data from two clinical trials revealed that PCa patients expressing *tERG*-positive fusions frequently exhibit resistance to antiandrogens, and this is linked to elevated GR levels after treatment. We also discovered that GR binds to ERG’s DNA-binding domain, stabilizing ERG and mitigating an intrinsic mechanism of allosteric autoinhibition. As a result, ERG’s transcriptional activity increases when it forms complexes with GR. In patient-derived xenografts, *tERG*-positive models demonstrated heightened sensitivity to GR inhibition compared to *tERG*-negative models. These findings identify fused *ETS* genes as a novel class of PCa biomarkers, predicting resistance to second-generation antiandrogens. In addition, we identify effective drug combinations and suggest that corticosteroid therapy may be contraindicated for patients with PCa expressing an *ETS* fusion gene.

## Results

### Tumoral tERG predicts resistance of PCa patients to antiandrogen treatments

**Figure 1:**
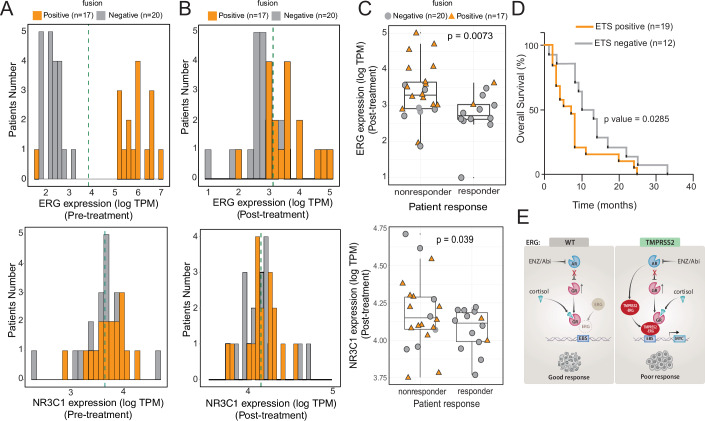
The status of ERG (wild-type or rearranged) and GR abundance predicts the responses of patients with prostate cancer to ADT plus enzalutamide (see also Fig. EV1). (**A**, **B**) Biopsies were obtained from 37 men with intermediate- to high-risk prostate cancer before receiving ADT plus enzalutamide for 6 months. The tissues were used for IHC, whole-exome sequencing, and RNA-sequencing. Shown are the distributions of pre- (**A**) and post-treatment (**B**) levels of ERG (upper panels) and GR (NR3C1; lower panels) in all patients. Fusion status of ERG in patients is shown in orange for positive fusion (tERG) and in gray for negative fusion (wild-type ERG). (**C**) Post-treatment ERG and GR expression levels are presented against patient response to treatment with ADT plus enzalutamide. The *P* values presented were determined using either one-sided Wilcoxon (ERG) or one-sided unpaired *T* test (GR). The central lines of the boxplots represent the medians, whereas box bounds represent the 25th and 75th percentiles, and the whiskers extend to the minimum and maximum values. (**D**) Data were derived from the control arm of clinical trial NCT01576172 (NCI 9012). All patients included in this arm (*n* = 58) were treated with abiraterone plus prednisone. Biopsy samples were obtained from metastatic sites and analyzed using RNA-seq and immunohistochemistry (for GR and ERG). Shown are progression-free survival (PFS) curves corresponding to the tERG-negative and tERG-positive groups (*P* = 0.0285; Gehan–Breslow-Wilcoxon two-sided test). (**E**) A working model depicting the inferred functional interaction between TMPRSS2-ERG and the hormone-activated glucocorticoid receptor (GR) in prostate cancer. Normally, AR suppresses GR in tumors driven by the androgen receptor (AR). However, by inhibiting AR signaling, drugs like enzalutamide (ENZ) and abiraterone (Abi) relieve this repression, hence they upregulate GR. In ~50% of prostate tumors, the androgen-inducible TMPRSS2 gene is fused to ERG. The fused gene, TMPRSS2-ERG (tERG), is induced by AR, and the encoded protein can form stable complexes with active GRs. However, this may not occur in fusion-negative tumors. Once formed, tERG–GR complexes bind with the ERG-binding site (EBS) on DNA to stimulate transcription of MYC and other oncogenic targets. This mechanism can explain why TMPRSS2-ERG-positive tumors can gain resistance to antiandrogen treatments. *P < 0.05; **P < 0.01; ***P < 0.001; ****P < 0.0001. Note that exact *P* values, statistical tests, sample sizes, and error bar definitions for all panels are provided in Appendix Table S3.

**Figure EV1:**
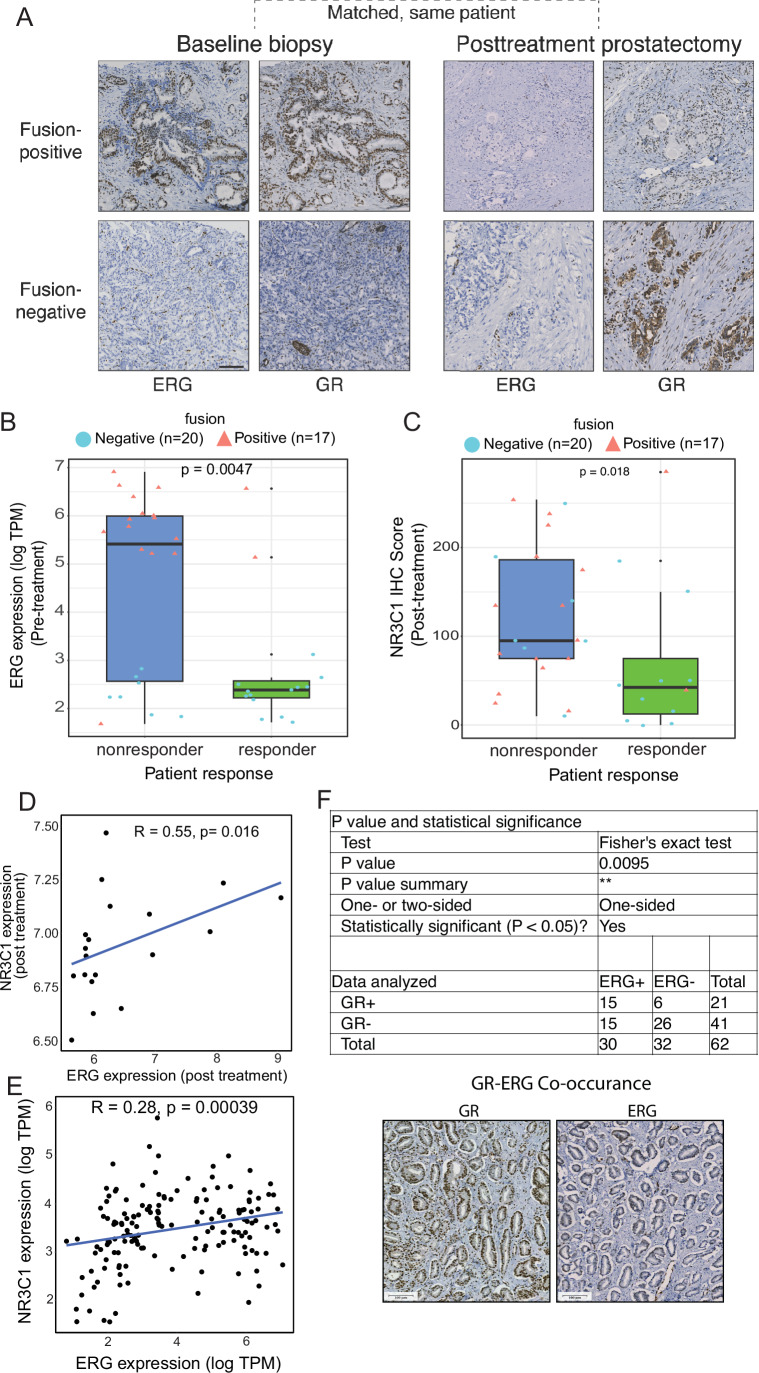
Immunohistochemical and computational analyses pre- and post-treatment with ADT plus enzalutamide indicate that tERG positivity and high GR (post-treatment) can predict resistance to antiandrogens (related to Fig. 1). (**A**) Targeted biopsies were obtained from 37 men with intermediate to high-risk PCa before receiving ADT plus enzalutamide for 6 months. Shown are representative micrographs of IHC staining with anti-ERG and anti-GR antibodies applied to serial sections of matched baseline (left) and posttreatment (right) prostate tumor tissues from a TMPRSS2-ERG fusion-positive (top) and from a fusion-negative (bottom) case. Bar, 100 µm. (**B**) ERG mRNA levels in responders and nonresponders before treatment. Statistical analysis was performed using paired *t* test (or Wilcoxon signed-rank test). *P < 0.05; **P < 0.01; ***P < 0.001 and ****P < 0.0001. In boxplots, the central line represents the median, the box bounds represent the 25th and 75th percentiles, and the whiskers extend to the minimum and maximum values. (**C**) GR protein expression assessed by IHC scoring in matched pre- and post-treatment samples from the responder cohort. Paired comparisons between pre- and post-treatment samples were performed using the Wilcoxon signed-rank test. (**D**) Shown are Spearman rank correlations between ERG and NR3C1 expression in 19 patients (from GSE102124) post-treatment with abiraterone plus ADT. (**E**) Spearman rank correlation between ERG and GR (NR3C1) expression post-ADT treatment, based on analysis of prostate RNA-seq data from 160 patients (WCDT dataset). (**F**) ERG and GR are favored to be mutually co-existing. Thin tissue sections were prepared from sixty-two patients who underwent standard-of-care prostatectomies with no hormonal therapy. Immunohistochemistry analysis was performed using anti-GR and anti-ERG antibodies. (upper panel) The table presents results obtained after performing a one-sided Fisher’s exact test that examined whether the co-occurrence of GR and ERG was favored. Listed are the numbers of ETS-positive and ETS-negative tumors, along with the status of GR expression. (lower panel) Shown are example IHC analyses from a representative ERG-positive tumor. Exact *P* values, statistical tests, sample sizes, and error bar definitions for all panels are provided in Appendix Table S3.

We began by re-analyzing data collected by a recent PCa clinical trial, National Cancer Institute (NCI) 15-c-0124 (NCT02430480). This study performed immunohistochemical (IHC) analysis, whole-exome and RNA-sequencing on biopsy samples from 37 men with PCa, before they received ADT plus enzalutamide for 6 months (Karzai et al, 2021; Wilkinson et al, 2021). As expected, there is a bimodal distribution of ERG’s mRNA abundance in the pre-treatment data, and this corresponds to the presence of *ERG* fusions in the high IHC group (Figs. 1A and EV1A). Due to regulation by androgens, ERG levels were significantly altered post antiandrogen treatment: reduced in the positive tERG patients (*P *= 5.19E-07, one-sided Wilcoxon test) and increased in the negative tERG patients (*P* = 0.00037; Fig. 1B). Notably, along with AR and ERG, GR (encoded by the *NR3C1* gene) has been reported to play an important role in PCa (Arora et al, 2013; Montgomery et al, 2015; Zhao et al, 2022). In contrast to ERG, GR displayed a unimodal pre-treatment distribution but, irrespective of ERG’s status, GR’s IHC increased substantially post treatment (Figs. 1A,B and EV1A), likely due to relief of the previously reported AR-mediated suppression of GR (Puhr et al, 2018; Shah et al, 2017). Quite surprisingly, the majority of patients with tERG and high *ERG* expression at baseline exhibited resistance to the antiandrogen treatment (*P* = 0.0047; one-sided Wilcoxon test; Fig. EV1B). Treatment resistance was also associated with high post-treatment ERG (*P* = 0.0073; Fig. 1C, upper panel), as well as with high post-treatment GR levels, which were determined using either mRNA abundance (*P* = 0.039; Fig. 1C, lower panel) or IHC (*P* = 0.018; Fig. EV1C). Together, these observations uncovered a functional association between the mutant form of ERG, high GR, and resistance to enzalutamide.

Next, we asked if the correlation between tERG expression and lack of response to antiandrogen treatment extends to metastatic castration-resistant prostate cancer (mCRPC). For this, we analyzed the results of another clinical trial, NCT01576172 (NCI 9012) (Hussain et al, 2018). All patients of this trial underwent metastatic site biopsy, stratified by the status of ERG and ETV1 (using IHC and in situ hybridization, respectively), and randomly assigned to abiraterone plus prednisone, either without (arm A) or with an inhibitor of poly(ADP-ribose) polymerase-1 (PARP1), veliparib (arm B). We focused on a subset of arm A patients (*N* = 58) for whom survival data were available. These patients presented samples with matching RNA-seq, GR, and ERG immunohistochemistry, and their PSA responses were available. A summary Kaplan–Meier survival analysis is presented in Fig. 1D. As predicted, the median progression-free survival (PFS) of the *tERG*-negative group (11.5 months) was significantly longer (*P* = 0.0285; Gehan–Breslow–Wilcoxon test) than the PFS rate of the *tERG*-positive cohort (7.0 months).

In conclusion, according to the analysis of the results reported by two independent clinical trials (a total of 95 patients), expression of the fused form of the *ERG* gene might predict relatively poor response of patients with PCa to treatments with two different ARSIs, enzalutamide and abiraterone plus prednisone.

### The association between *tERG* and both resistance to ARSIs and relatively high GR offers a working model

To further examine potential clinical associations between the levels of ERG and GR, we analyzed two post-treatment datasets: a microarray set, GSE102124 (Sowalsky et al, 2018), derived from 19 patients previously treated with ADT plus abiraterone (Fig. EV1D), and an RNA-seq dataset (Lundberg et al, 2023; Quigley et al, 2018), derived from 160 patients that were treated with front-line ADT (Fig. EV1E). The presented scatter plots and respective *P* values, 0.016 and 0.00039, further supported the existence of a positive association between ERG and GR in clinical specimens.

Taken together with the previously reported stimulatory interactions between GR and another ETS family member, FLI1 (Srivastava et al, 2019), the data shown in Figs. 1 and EV1 suggest the following working model (see Fig. 1E): Under normal conditions, GR expression is suppressed by androgen signaling (Puhr et al, 2018; Shah et al, 2017; Xie et al, 2015). However, ARSIs like enzalutamide (ENZ) and abiraterone (Abi) can lift this suppression and increase GR expression. In tumors positive for the *TMPRSS2-ERG* fusion, GR may physically interact with tERG, in similarity to the reported GR-FLI1 complexes. Once the putative GR-tERG complexes are formed, they bind to the ERG-binding site (EBS) on DNA, thereby promoting the transcription of oncogenic targets like MYC. Notably, upregulation of GR can bypass AR signaling by activating a partly overlapping set of target genes (Arora et al, 2013; Puhr et al, 2018). According to our results, this predicted sequence of events may not occur in *TMPRSS2*-negative tumors, which typically respond well to antiandrogen treatments (Fig. 1C). To initiate validation of this model, we performed dual-stain IHC (using anti-GR and anti-ERG antibodies) on serial sections from prostate tumors of 62 patients who underwent prostatectomy without prior hormonal therapy (Fig. EV1F). Co-expression was assessed using a one-sided Fisher’s exact test to determine whether co-occurrence was favored. In line with our working model, this analysis revealed that cells expressing tERG were significantly more likely to also express GR (p = 0.0095), indicating that ERG and GR preferentially co-exist within the same cells, rather than exhibiting mutual exclusivity.

### GR forms druggable complexes with oncogenic ETS proteins

**Figure EV2:**
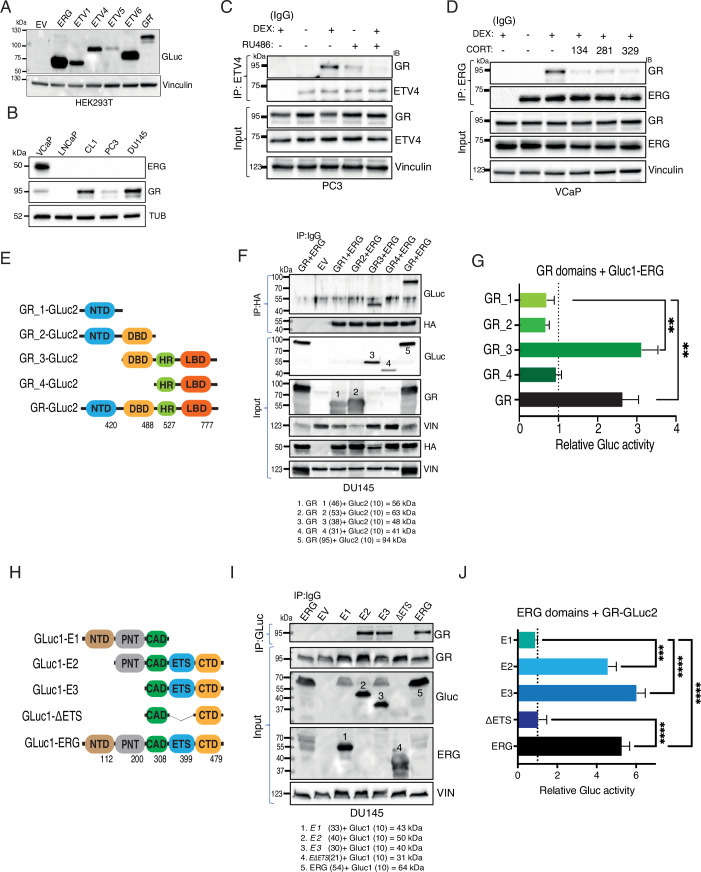
The DBD-HR-LBD region of GR interacts with the ETS domain of ERG (related to Fig. 2). (**A**) An immunoblot showing the levels of the Gluc fusion proteins we transiently expressed in HEK293T cells (5x10⁵). Cells were harvested 24 h post-transfection and processed for immunoblotting. Vinculin was used as the gel loading control. (**B**) Comparative analysis of ERG and GR levels in the PCa cell lines we employed. Whole extracts of the indicated cell lines were prepared and subjected to immunoblotting for ERG and GR. Tubulin (TUB) was used to ensure equal gel loading. (**C**) Serum-starved PC3 cells (naturally overexpressing ETV4) were treated for 60 min with vehicle, DEX (1 μM), RU486 (1 μM), or the combination of drugs. Extracts were processed for co-immunoprecipitation (IP) and immunoblotting (IB). IgG, control antibody. (**D**) Serum-starved VCaP cells were treated for 60 min with vehicle (CON) or DEX (1 μM), in the absence or presence of the indicated SGRM compounds (C134, C281, or C329; 10 μM each). Whole-cell extracts were processed for co-immunoprecipitation of ERG and GR, followed by immunoblotting. (**E**, **F**) A schematic representation of the various domains of GR fused to Gluc2, along with their levels of expression in HEK293T cells. The indicated domains were inserted N-terminally to Gluc2. NTD N-terminal domain, DBD DNA-binding domain, HR hinge region, LBD ligand binding domain. DU145 cells were transfected with Gluc1-ERG plasmids encoding different deletion mutants of ERG (see molecular weight calculations at the bottom of the panel). The Gluc1-ERG proteins were immunoprecipitated using an antibody specific to HA. Immunoblotting was performed using antibodies that detected the endogenous forms of GR. (**G**) HEK293T cells (6 × 10³) were co-transfected with Gluc2 plasmids encoding different domains of GR and a Gluc1 plasmid encoding ERG (full length). Following 24 h of incubation, cells were starved overnight and then treated for 60 min with vehicle, or with DEX (1 μM). The normalized luminescence activity of each construct is presented. Data are presented as mean ± SEM. Statistical analysis was performed using two-way ANOVA with the Tukey’s multiple comparisons test. The experiment was repeated thrice with biological triplicates. Error bars represent the means ± SEM. (**H**, **I**) A schematic representation of the various domains of ERG fused to Gluc1, along with their levels of expression in transfected DU145 cells. The indicated domains were inserted C-terminally to Gluc1. NTD N-terminal domain, Pointed domain, CAD central activation domain, ETS DNA-binding domain, CTD C-terminal domain. See cloning primer sequences in Appendix Table S1. (**J**) The Gluc2 plasmid encoding full-length GR was co-transfected in DU145 cells together with the indicated Gluc1 plasmids encoding different domains of ERG. Following 24 h of incubation, cells were starved overnight and then treated for 60 min with vehicle or DEX (1 μM). Luminescence was determined in biological triplicate. All experiments were repeated three times. Data are presented as mean ± SEM. Statistical analysis was performed using two-way ANOVA with Tukey’s multiple comparisons test. The experiment was repeated twice with biological triplicates. Error bars represent mean ± SEM. **P < 0.01; ***P < 0.001; ****P < 0.0001. Note: the quantitative analyses (**F**, **I**) were repeated at least twice. Exact *P* values, statistical tests, sample sizes, and error bar definitions for all panels are provided in Appendix Table S3.

**Figure 2:**
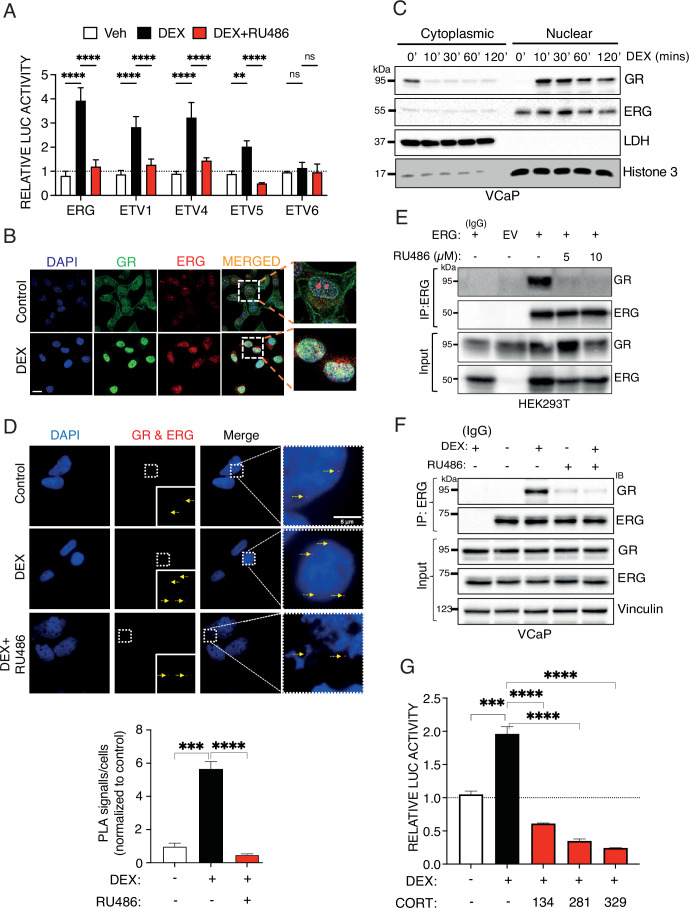
The physical interactions between GR and ERG take place in the nucleus, and they can be disrupted by a GR antagonist (see also Fig. EV2). (**A**) HEK293T cells growing in charcoal-stripped serum (CSS) were transfected (in sextuplicate) with the GR-Gluc2 plasmid, along with the indicated Gluc1-ETS plasmids. Twenty-four hours post transfection, cells were treated with vehicle, DEX (1 μM), or a combination of DEX and RU486 (1 μM). Shown are normalized fold changes in luminescence. Data were analyzed by ordinary two-way ANOVA with Tukey’s multiple comparisons test. *n* = 3 biological replicates per condition. **P < 0.01; ****P < 0.0001; ns, not significant. Note that ETV6 was used as a negative control. (**B**) CSS-grown HeLa cells were stimulated with either DEX or vehicle (control) for 10 min. Thereafter, cells were fixed and processed for immunofluorescence analysis that used DAPI (blue; nucleus), and antibodies specific to GR (green) and ERG (red). The framed areas in the merged column are magnified in the rightmost column. Scale bar, 20 μm. (**C**) Shown are representative images of VCaP prostate cancer cells that were starved overnight for serum factors and then treated for 30 min with either DEX (1 µM), prednisone (1 µM), or RU486 (10 µM). The PLA test detected short-range (10–40 nm) interactions between GR and ERG by means of a pair of oligonucleotide-labeled secondary antibodies and hybridizing connector oligonucleotides, which link the PLA probes only if they are near each other. Nuclei were labeled with DAPI (blue). Shown are the images of ERG-GR clusters (in red; arrowheads) and merge panels, whereas the bottom panel shows a histogram with the quantification of the PLA signals (*n* > 40 cells per condition). The experiment was repeated twice (*P* < 0.001). The error bars indicate SEM. Scale bar, 5 µm. (**D**) Serum-starved VCaP cells expressing tERG were treated with DEX (1 μM) for the indicated time intervals. Untreated and DEX-treated cells were subjected to subcellular fractionation and immunoblotting for GR and ERG. LDH and histone 3 were used as cytoplasmic and nuclear markers, respectively. Yellow arrows indicate the nuclear co-localization of GR and ERG following DEX treatment. (**E**) HEK293T cells were transfected with an empty vector (EV) or with a plasmid encoding ERG, and 24 h post transfection cells were treated or not with RU486 for 8 h. Thereafter, we subjected cell extracts to immunoprecipitation with an anti-ERG antibody or with a control antibody (IgG) and immunoblotted with the indicated antibodies. A fraction (5%) of the lysate was used as an input control. (**F**) Serum-starved VCaP cells were treated for 60 min with vehicle, DEX (1 μM), RU486 (1 μM), or the combination of drugs. Extracts were processed for co-immunoprecipitation (IP) and immunoblotting (IB). Note that two different anti-ERG antibodies were used, one from Cell Signaling Technology (CST) and the other from Santa Cruz Biotechnology (SCB). IgG, control antibody. (**G**) Serum-starved VCaP cells were treated for 60 min with DEX (1 μM) in the absence or presence of the following selective GR modulators (SGRMs, 10 μM): CORT125134 (C134, relacorilant), CORT125281 (C281, exicorilant), and CORT125329 (C329). Luminescence was determined in biological triplicate. Data are presented as mean ± SEM. Statistical analysis was performed using one-way ANOVA with Tukey’s multiple comparisons test. All experiments were repeated three times. **P < 0.01; ***P < 0.001. Exact *P* values, statistical tests, sample sizes, and error bar definitions for all panels are provided in Appendix Table S3. Source data are available online for this figure.

In anticipation of direct interactions between ERG and the hormone-bound form of GR, we employed the previously described protein complementation assay (reviewed in (Michnick et al, 2007)). The modified protocol we used utilized inactive fragments of Gaussia luciferase (Gluc), which were split into an amino-terminal fragment, Gluc1, and a carboxyl-terminal fragment, Gluc2. Firstly, we constructed a library of Gluc1 fusion proteins that included the ETS proteins frequently rearranged in PCa (i.e., ERG, ETV1, ETV4, and ETV5), along with ETV6, as a negative control. The levels of expression of the Gluc proteins are shown in Fig. EV2A. Note that Gluc1-ETV5 displayed relatively low expression. The normalized signals presented in Fig. 2A indicated that stimulation with DEX can significantly increase the interaction between GR and all ETS proteins we analyzed, except for ETV6. In addition, the hormone-induced interactions were inhibited when cells were treated with a combination of DEX and RU486 (mifepristone), a GR antagonist.

Next, we visualized the interaction between GR and ERG at the subcellular level. Due to the relatively low signal-to-noise ratio observed with PCa cells, we initially utilized HeLa cells that were pre-starved, stimulated with DEX, and then examined. Once stimulated with the steroid hormone, the endogenous GR rapidly translocated to the nucleus (Fig. 2B). In parallel, the diffusely distributed nuclear ERG redistributed into intranuclear structures that contained the translocated GR. However, some ERG-containing puncta, which were devoid of GR, acquired polarized peripheral locations of an unknown nature. Subcellular fractionation corroborated these observations (Fig. 2C): as early as 10 min after exposure to DEX, the initially cytoplasmic GRs translocated to the nucleus, which contained most of the cellular ERG molecules. Notice that we used a nuclear marker along with a cytoplasm-specific marker and observed early DEX-dependent enhancement of the GR protein and late downregulation of this receptor, probably due to the previously described “homologous downregulation” process (Oakley and Cidlowski, 1993).

To validate physical interactions between GR and tERG in living cells, we first analyzed their expression levels in a battery of PCa cell lines (Fig. EV2B) and carried out proximity ligation assays (PLA) that used the respective antibodies. Prior to the assay, VCaP prostate cancer cells were starved overnight for serum factors and then treated for 30 min with either DEX or a combination of DEX and RU486. By means of a pair of oligonucleotide-labeled secondary antibodies and hybridizing connector oligonucleotides, this test detected short-range (10–40 nm) interactions between GR and ERG (Fig. 2D). Notably, the interaction could be stimulated by DEX and inhibited by RU486. In line with these observations, when untreated HEK293T cells ectopically expressing ERG or an empty vector (EV), were subjected to co-immunoprecipitation assays we detected high levels of the endogenous GR protein that was pulled-down with an anti-ERG antibody (Fig. 2E). Congruent with specific interactions, treatment with RU486 abolished complex formation. Similarly, in ERG immunoprecipitates obtained from tERG-expressing PCa cells (VCaP), which were pre-starved for steroid hormones, DEX increased the amount of GR in ERG immunoprecipitates (Fig. 2F). Although *TMPRSS2*-*ETV4* fusions are less frequently overexpressed than *TMPRSS2-ERG* among human patients, increased abundance of ETV4 promotes tumor growth in xenograft models (Hollenhorst et al, 2011b; Pellecchia et al, 2012). As expected, analysis of PC3 cells, which endogenously overexpress ETV4, confirmed physical GR-ETV4 associations that were increased by DEX and decreased by RU486 (Fig. EV2C).

Because non-steroidal GR antagonists hold promise for PCa treatment (Chai et al, 2023), we examined a series of three non-steroidal selective GR antagonists (from Corcept Therapeutics). In similarity to RU486, each of the three antagonists inhibited the ability of DEX to drive formation of the GR-ERG complex (Fig. EV2D), as well as reduced the luminescence signals observed when combined with DEX (Fig. 2G). In summary, following stimulation with DEX, the glucocorticoid receptor translocates to the nucleus and forms non-covalent bonds with ERG, but these interactions can be inhibited using pharmacological agents.

### The DNA-binding domain of GR and the ETS domain of ERG are engaged in complex formation

Similar to other ETS family members, ERG harbors an ∼80-residue domain, PNT, and a conserved ETS domain, which binds DNA with preference for an invariant 5′-GGA(A/T)-3′ core (Hollenhorst et al, 2011a). Likewise, GR contains a DNA-binding domain (DBD), an N-terminal modulatory domain (NTD), a hinge region (HR), and a C-terminal ligand-binding domain, LBD (Frank et al, 2021). To map the domains mediating the interaction between GR and ERG, we made use of several deletion mutants of each protein. The GR deletion mutants schematically presented in Fig. EV2E were cloned into the Gluc2 plasmid and expressed in both DU145 (Fig. EV2F) and HEK293T cells (Fig. EV2G), along with either full-length HA-tagged ERG (Fig. EV2F) or Gluc1-ERG (Fig. EV2G). The results of the co-immunoprecipitation and protein complementation assays revealed that the region comprising DBD + HR + LBD (mutant denoted GR_3) generated essentially similar signals to those generated by the full-length GR (Fig. EV2F; Fig. EV2G). No protein complementation signal was observed when using another GR deletion mutant that included HR + LBD. Hence, we concluded that the DBD of GR mediates the interaction with ERG. Similar analysis of a set of ERG deletion mutants (Fig. EV2H; see list of primers in Appendix Table S1) indicated that the ETS domain is critical for the interaction (Fig. EV2I,J). In conclusion, the DNA-binding domain of GR forms a physical complex with the DNA-binding domain of ERG.

### A complex comprising GR and HSP90 protects tERG from degradation

**Figure 3:**
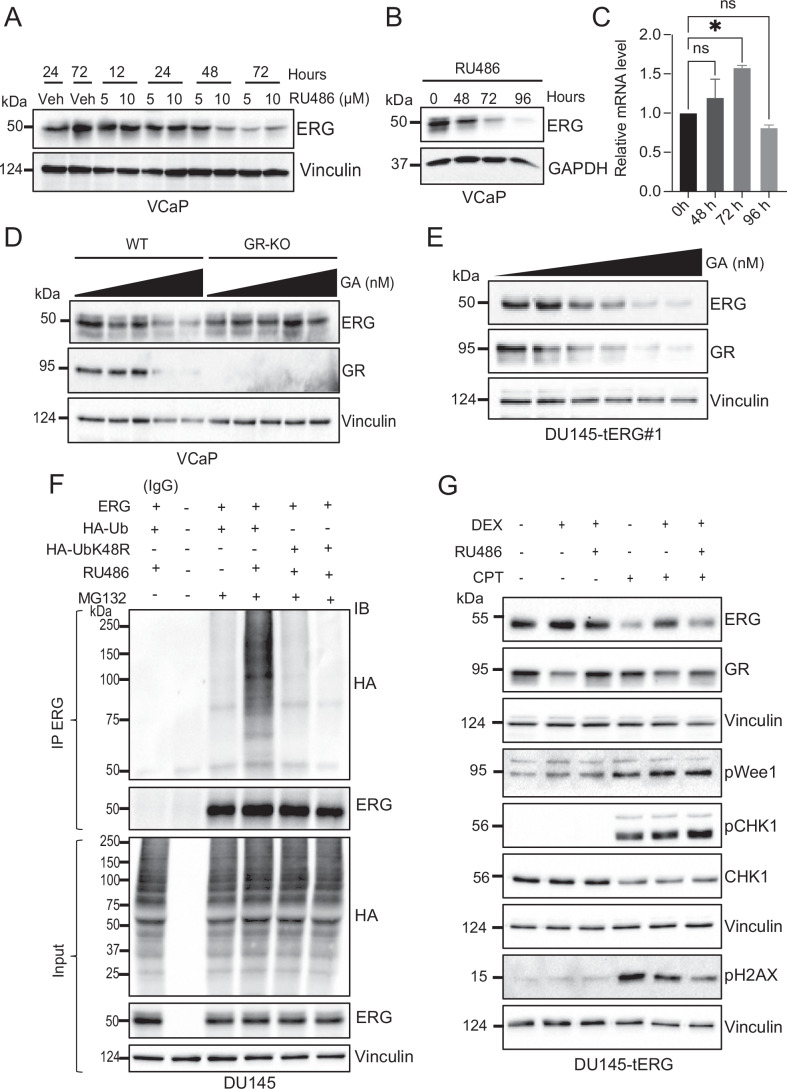
GR inhibition leads to ubiquitination and degradation of the ERG oncoprotein (see also Fig. EV3). (**A**) VCaP cells were treated for 12, 24, 48, and 72 h with the indicated concentrations of RU486 (either 5 or 10 microM) and, thereafter, whole-cell extracts were probed for the endogenous ERG or vinculin. (**B**) VCaP cells were treated with RU486 (10 μM) for the indicated time intervals, and ERG protein levels were assayed using immunoblotting. Note that the control cells were treated with the solvent (ethanol) for 72 h. GAPDH served as a loading control. (**C**) ERG mRNA levels from (**B**) were assayed using RT-qPCR and specific primers. Data are presented as mean ± SEM (*n* = 2) relative to 0-h control. Statistical analysis was performed using one-way ANOVA with Dunnett’s multiple comparisons test (compared to 0 h). The experiment was repeated twice, and representative results are shown. *P < 0.05; ns, not significant. (**D**) Wild-type (WT) VCaP cells and the respective GR-KO cells were treated with the HSP90 inhibitor geldanamycin (GA). Twenty-four hours later, whole cell extracts were prepared and subjected to immunoblotting that detected ERG, GR, and vinculin, the gel loading control protein. (**E**) DU145 cells stably overexpressing tERG were treated for 48 h with the indicated concentrations of GA. Post treatment, the cells were collected and their cleared extracts subjected to immunoblotting that used the indicated antibodies. (**F**) DU145 cells were transfected with HA-Ub and the indicated ERG-encoding plasmids. Sixteen hours post transfection; the cells were treated for 48 h with RU486. MG132 (25 μM) was added 8 h prior to the end of the incubation. Cell extracts were subjected to immunoprecipitation using an anti-ERG antibody. Immunoblotting (IB) used an anti-HA-ubiquitin antibody. A fraction (5%) of the total extract was used as the input control. (**G**) DU145-tERG cells were treated for 8 h with camptothecin (CPT; 0.5 μM), in the absence or presence of DEX (1 μM), either alone or in combination with RU486 (10 μM). Cleared cell extracts were resolved using electrophoresis and western blotting that employed the indicated antibodies, including antibodies specific to the phosphorylated forms of CHK1, Wee1, and histone 2AX. Note that the exact *P* values, statistical tests, sample sizes, and error bar definitions are provided in Appendix Table S3. Source data are available online for this figure.

**Figure EV3:**
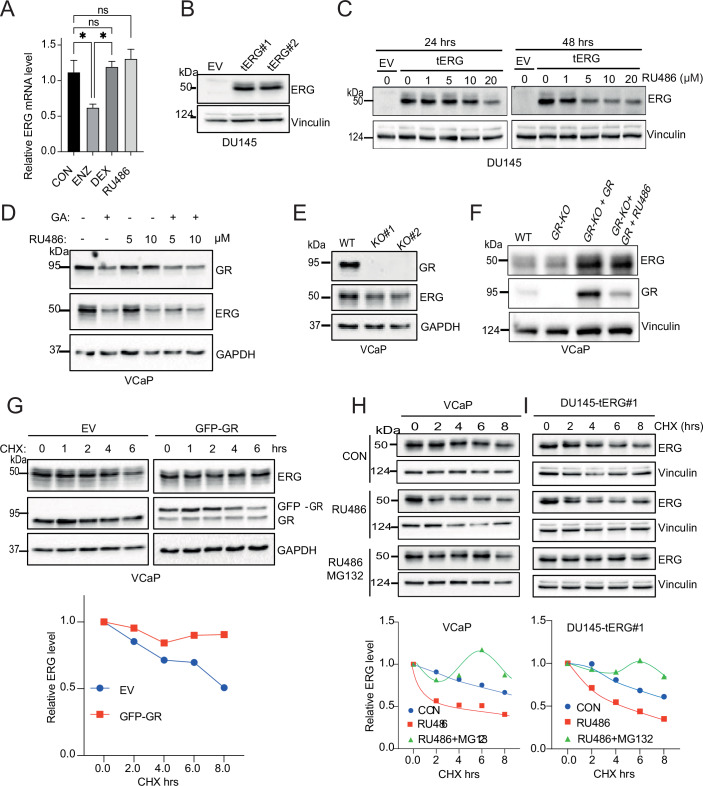
Inhibition of GR destabilizes ERG (related to Fig. 3). (**A**) VCaP cells were treated with vehicle, DEX, ENZ, or RU486 (10 μM, each) for 48 h and later subjected to RT-qPCR that determined ERG transcript levels. Statistical analysis was performed using one-way ANOVA with Dunnett’s multiple comparisons test. The experiment was performed twice with *n* = 2 biological replicates. (**B**) DU145 cells were transfected with EV or with tERG-encoding plasmids and selected with blasticidin for 10 days. The indicated positive clones were used as the stable tERG-overexpressing cells. (**C**) DU145 cells stably overexpressing tERG were treated for 24 or 48 h with increasing concentrations of RU486. Thereafter, whole cell extracts were prepared and subjected to immunoblotting, as indicated. (**D**) VCaP cells were treated with geldanamycin (GA; 1 μg/ml), RU486 (either 5 or 10 μg/ml) or with the combination of drugs for 48 h. Whole-cell extracts were prepared and subjected to immunoblotting. (**E**) GR was stably knocked out in VCaP cells using the CRISPR/Cas9 system and specific sgRNAs. Two cell clones were separately established. WT cells were transfected with a control guide RNA. Cell extracts were examined using immunoblotting for GR and ERG. GAPDH was used to control gel loading. (**F**) GR-knockout VCaP cells were transfected with a GR expression vector and treated with RU486 (10 μM) for 48 h. Whole-cell extracts were subjected to immunoblotting with antibodies against ERG, GR, and vinculin (the loading control). (**G**) VCaP cells were transfected with a GFP-GR plasmid and later treated with cycloheximide (CHX; 50 μg/ml). Cells were harvested for immunoblotting at the indicated time points. The corresponding signals from the blot are presented in a graph. (**H**, **I**) VCaP (**H**) and DU145-tERG#1 (**I**) cells were treated with vehicle or RU486 (10 μM) for 48 h and then treated with cycloheximide (CHX; 50 μg/ml) and/or MG132 (25 μM). Cells were harvested at the indicated time points and extracts prepared for immunoblotting. Western blots that were quantified and normalized are shown as a line graph. Note: all assays were repeated 2–3 times. For the exact *P* values, statistical tests, sample sizes, and error bar definitions for all panels, see Appendix Table S3.

Degradation of the ERG oncoprotein suppresses PCa progression, and likewise, resistance to degradation serves as a mechanism that contributes to disease progression (An et al, 2015; Gan et al, 2015; Hong et al, 2020). Hence, we set out to investigate whether by forming a physical complex with GR, tERG gains stability. To test this model, we treated VCaP cells with RU486. As shown in Fig. 3A,B, RU486 treatment reduced the abundance of ERG in a time- and dose-dependent manner. These observations proposed two types of potential mechanisms: transcriptional or posttranslational. The transcriptional option was weakened because we observed inconsistent DEX- and RU486-induced changes in the abundance of mRNAs encoding tERG (Fig. 3C), although a treatment making use of enzalutamide, an AR inhibitor, transcriptionally reduced ERG’s mRNA levels (Fig. EV3A). To further examine the possibility that GR transcriptionally or otherwise activates ERG, we transfected the ERG-low DU145 cells with a tERG-encoding plasmid and established two independent cell lines (Fig. EV3B). As expected, the stable cellular derivatives retained RU486-induced reduction in the level of tERG (Fig. EV3C). Taken together, these results indicated that GR regulates tERG at the posttranslational level.

Like other steroid hormone receptors, GR functionally depends on the HSP90 molecular chaperone (Kirschke et al, 2014). Because our results raised the possibility that GR can chaperone ERG, inhibiting the GR-HSP90 complex offers an alternative way to destabilize ERG using geldanamycin (GA), a specific HSP90 inhibitor. In agreement with this scenario, treating VCaP cells with GA decreased the levels of GR, and this was associated with lower tERG levels (Fig. EV3D). In the next step, we established GR-knockout VCaP cells (Fig. EV3E) and treated them with GA. In line with functional GR-ERG complexes, GA was unable to downregulate ERG in the absence of GR (Fig. 3D).

An alternative interpretation posits that the reported ability of HSP90 to chaperone AR (Centenera et al, 2015), rather than GR, accounts for the capacity of GA to reduce ERG abundance. To test this possibility, we employed the above-mentioned DU145-tERG cells, since their parental line expresses only negligible levels of AR. Notably, even in these cells, GA induced a clear and concomitant downregulation of both GR and ERG (Fig. 3E). Taken together, these findings indicate that inhibition of GR—either directly or indirectly through HSP90—can reduce tERG abundance, consistent with a model in which HSP90-bound GR molecules physically chaperone tERG. As a final validation of this model, we rescued GR in GR-knockout VCaP cells and assessed ERG levels in the transfected cells. In contrast to the partial reduction of ERG observed upon GR loss, GR re-expression robustly increased ERG abundance (Fig. EV3F), reinforcing the notion that GR exerts a stabilizing effect on tERG.

### GR protects tERG from chemotherapy-induced proteasomal degradation

Next, we made use of cycloheximide (CHX, a protein synthesis inhibitor), a GFP-tagged GR, and VCaP cells. Densitometry analysis and normalization of immunoblots shown in Fig. EV3G indicated that the projected half-life of ERG was prolonged when GR was overexpressed, and the cells were pre-treated with CHX. Next, we checked if the enhanced degradation rate of ERG following RU486 treatment of PCa cells (VCaP and DU145-tERG) would be rescued by MG132, a 26S proteasome inhibitor (Fig. EV3H,I). As expected, RU486 shortened the half-life of ERG, but the ability of this drug to enhance ERG degradation was nearly nullified when cells were pre-treated with MG132. These results predicted that blocking GR sorts the physically associated ERG proteins to proteasomal degradation by means of a preparatory step involving conjugation of poly-ubiquitin chains that use lysine-48 of ubiquitin as a branching point. This model was tested in cells co-transfected with HA peptide-tagged ubiquitin constructs, either WT or the K48R mutant form of ubiquitin, along with an ERG plasmid. As shown in Fig. 3F, immunoprecipitation resolved a fraction of ERG that underwent poly-ubiquitination under basal conditions (untreated cells), but this relatively small fraction was strongly enhanced following treatment with RU486. Still, no conjugation of the K48R ubiquitin mutant was detectable. In conclusion, inhibiting GR instigates K48-branched chains of ubiquitin, which likely sort tERG molecules to degradation by the proteasome.

It has been reported that irradiation or treatment with certain chemotherapeutic agents, such as camptothectin (CPT), enhances proteasomal degradation of both wild-type ERG and tERG in a mechanism that requires pre-phosphorylation of ERG at two sites: threonine 187 (by GSK3β) and tyrosine 190, by a cascade that includes ATR, CHK1, and WEE1 (Hong et al, 2020). We confirmed that CPT-induced DNA damage activated CHK1 and WEE1 in CPT-treated DU145-tERG cells and showed that this culminated in phosphorylation of histone H2AX (gamma-H2AX), a marker of double-strand breaks (Fig. 3G). In line with the previous report, CPT enhanced ERG degradation. However, treatment with DEX prevented stress-induced degradation of ERG, and as expected, RU486 eliminated the effect of DEX in cells exposed to CPT. In summary, the GR-HSP90 complex not only physically binds with ERG but also protects the complex from proteasomal degradation in both naive cells and in chemotherapy-treated PCa cells. These observations might bear clinical significance, especially for chemotherapy-treated tERG-positive patients with PCa.

### Tethering enables GR to augment the transcriptional activity of tERG

**Figure 4:**
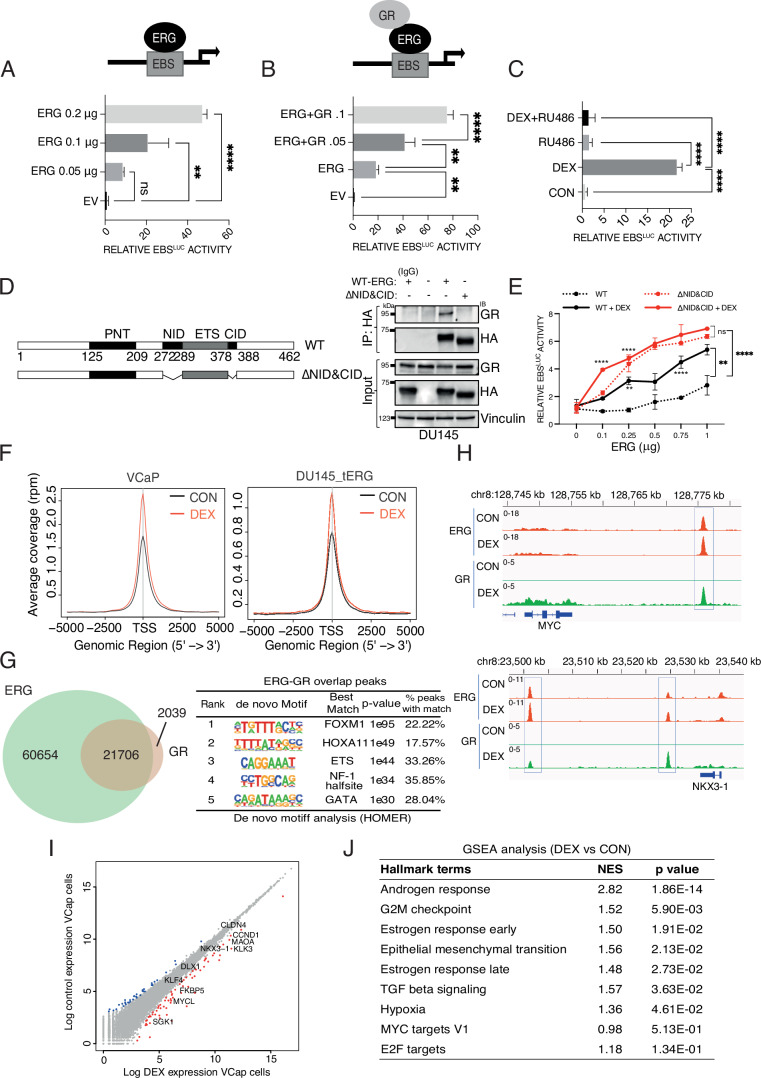
By relieving allosteric autoinhibition, GR enhances the transcriptional function of ERG (see also Fig. EV4). (**A**) HEK293T cells were co-transfected with an ETS binding site (EBS)-luciferase reporter plasmid and the indicated amount (in micrograms) of a plasmid encoding ERG. Luciferase activity was determined 24 h later, using the Dual-Luciferase Assay kit (from Promega). Data are presented as mean ± SEM (*n* = 3). Statistical analysis was performed using one-way ANOVA with Dunnett’s multiple comparisons test (compared to EV control). (**B**) HEK293T cells were co-transfected as in A with an EBS-luciferase reporter, an ERG plasmid, and the indicated amounts of a plasmid encoding GR. Twenty-four hours later, luciferase activity was determined using the Dual-Luciferase Assay kit (from Promega). Data are presented as mean ± SEM (*n* = 3). Statistical analysis was performed using one-way ANOVA with Tukey’s multiple comparisons test. (**C**) HEK293T cells growing in medium supplemented with charcoal-stripped serum (10%) were co-transfected with plasmids encoding EBS-luciferase and an ERG plasmid (or an empty vector). Sixteen hours post transfection, cells were stimulated for 60 min with DEX (1 μM), RU486 (1 μM) or the combination. Luciferase activity was determined. Data are presented as mean ± SEM (*n* = 3). Statistical analysis was performed using one-way ANOVA with Tukey’s multiple comparisons test. (**D**) (Left panel) Schematic representations of the domain structures of wild-type ERG, along with the corresponding double deletion mutant lacking both the N- and C- terminal autoinhibitory domains (∆NID&CID). (Right panel) DU145 cells were transfected with the following plasmids: HA-tagged forms of ERG-WT and ΔNID&CID. Whole-cell extracts were prepared 24 h post transfection and processed for immunoprecipitation using an anti-HA antibody and immunoblotted for GR. IgG, control immunoglobulin. (**E**) HEK293T cells grown in medium supplemented with charcoal-stripped serum (10%) were co-transfected with an EBS-luciferase reporter, as well as with the WT or mutant ERG plasmids at increasing doses. Sixteen hours post transfection; cells were stimulated with DEX (1 μM). Luciferase activity was measured using a kit (from Promega). Data are presented as mean ± SEM (*n* = 2). Statistical analysis was performed using two-way ANOVA with Tukey’s multiple comparisons test. The experiment was performed twice with two biological replicates; representative results are shown. (**F**) CSS starved VCaP and DU145-tERG cells were treated for 1 h, in duplicates, with either vehicle or DEX. Thereafter, cells were fixed, and ChIP was performed using an antibody specific to ERG. The ChIP peak profile plots present genome-wide changes in ERG enrichment (average coverage) before (black) and after (red) DEX stimulation. TSS, transcription start site. (**G**) Venn diagram illustrating the overlap of ERG and GR-enriched peaks in DEX-treated VCaP cells. De novo motif analysis (HOMER) of the ERG and GR genomic overlap peaks was inferred. Shown are the top 5 enriched motifs (ranked by *P* value). (**H**) Integrated Genome View (IGV) tracks for ERG and GR within two target genes of ERG, MYC, and NKX3-1, in VCaP cells treated without or with DEX (1 mM). (**I**) VCaP cells grown in medium containing charcoal-stripped serum (10%) were treated with DEX (1 mM) for 24 h. Post-treatment, RNA was isolated and analyzed using nucleotide sequencing. Data are shown as log2 of average replicated DESeq2 normalized expression. Blue dots denote fold differential genes in Control vs DEX, and red dots denote fold differentially expressed genes in DEX vs Control. Previously identified ERG and GR target genes are labeled. The full list of DEGs is shown in Appendix Table S2. (**J**) Gene Set Enrichment Analysis (GSEA) showing significantly upregulated pathways in DEX-treated VCaP cells, in comparison to untreated cells. Statistical significance of pathway enrichment was determined using GSEA with a permutation-based false discovery rate. **P < 0.01; ****P < 0.0001; ns, not significant. Exact *P* values, statistical tests, sample sizes and error bar definitions for all panels are provided in Appendix Table S3. Source data are available online for this figure.

**Figure EV4:**
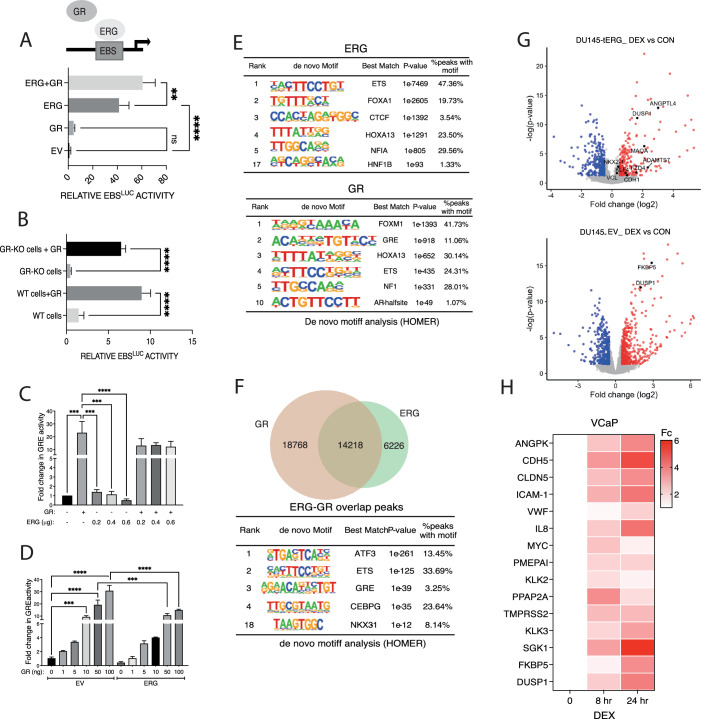
Genome-wide analysis of ERG-binding sites following stimulation with DEX (related to Fig. 4). (**A**) HEK293T cells were co-transfected with an EBS-luciferase reporter plasmid and the indicated plasmids encoding ERG and GR, or their combination. Luciferase activity was determined 24 h later using the Dual-Luciferase Assay kit (from Promega). Statistical analysis was performed using one-way ANOVA with Tukey’s multiple comparisons test. Three biological triplicates were used, and the experiments were repeated twice. Data are presented as mean ± SEM. Error bars represent the mean ± SEM. *P < 0.05; **P < 0.01; ***P < 0.001; ****P < 0.0001; ns, not significant. (**B**) Wild-type VCaP cells or the respective GR-KO derivative cells were co-transfected with an EBS-luciferase plasmid. A GR-encoding plasmid, or a control vector, was co-transfected, and 24 h later we performed a luciferase assay, in triplicates. Statistical analysis was performed using one-way ANOVA with Tukey’s multiple comparisons test. The experiment was repeated thrice. Data are presented as mean ± SEM. Error bars represent mean ± SEM. *P < 0.05, **P < 0.01, ***P < 0.001, ****P < 0.0001. ****P < 0.0001. (**C**, **D**) Serum-starved HEK293T cells were co-transfected with a GRE-luciferase promoter reporter, along with the indicated amounts of the ERG or/and GR expression vectors. Twenty-four hours later, luciferase activity was measured using a luciferase assay kit. Statistical analysis was performed using one-way ANOVA with Dunnett’s multiple comparisons test. We used biological triplicates and repeated the experiment twice. Data are presented as mean ± SEM. Statistical significance is denoted as *P < 0.05; **P < 0.01; ***P < 0.001 and ****P < 0.0001. (**E**) Shown are the results of de novo motif analysis featuring top-enriched motifs of ERG and GR peaks (deduced from ChIP-seq analysis of DEX-treated VCaP cells). (**F**) Venn diagram illustrating the overlap of ERG and GR-enriched peaks in DEX-treated DU145-tERG cells. Shown are de novo motif analyses (HOMER) of ERG-GR overlapping peaks featuring the top enriched motifs (ranked by *P* value). (**G**) CSS starved DU145-EV and tERG cells were treated with vehicle or DEX for 24 h. Post treatment, RNA was extracted for sequencing. The volcano plots display the DEGs. ERG and GR target genes are labeled in the plots. Statistical analysis was performed using DESeq2 (Wald test). (**H**) Serum-starved VCaP cells were treated with DEX (1 μM) for 8 or 24 h. Post-treatment, RNA was harvested and subjected to RT-qPCR using specific primers. GAPDH was used as a housekeeping control transcript. Note: the data shown in (**A**–**D**) were verified in two experiments. Note that the exact *P* values, statistical tests, sample sizes, and error bar definitions for all panels are provided in Appendix Table S3.

Upon hormonal stimulation, GR translocates to the nucleus to transcriptionally activate target genes containing the glucocorticoid response element (GRE). Alternatively, without binding directly to DNA, GR physically associates with and controls tethered TFs, either coactivators or corepressors (Gerber et al, 2021). To examine the prediction that GR enhances rather than represses the transcriptional program of ERG, we employed an ETS binding site (EBS)-luciferase reporter (Nhili et al, 2013). As expected, when the reporter was expressed together with increasing amounts of an ERG plasmid, we observed gradually increasing luciferase signals (Fig. 4A). This signal was further increased when GR was co-expressed (Fig. 4B). To exclude the possibility that GR directly activates transcription from the EBS, the reporter was used in the absence of an ectopic ERG. As shown in Fig. EV4A, GR alone could not enhance the luciferase signal in the absence of an exogenously expressed ERG, implying a piggyback (tethering) mode of GR action (see a scheme in Fig. 4B). Next, we confirmed that the GR-induced transcriptional activation of ERG could be modulated by GR-specific drugs: after co-transfection of an ERG plasmid and the EBS reporter, HEK293 cells were stimulated with DEX, RU486 or the combination. As anticipated, DEX robustly increased the EBS transcriptional signal, whereas the combination with RU486 abolished the effect of DEX (Fig. 4C). In conclusion, the ability of ERG to upregulate transcription from the EBS can be significantly enhanced by the agonist-activated form of GR.

To further support this scheme, we utilized GR-knockout VCaP cells (Fig. EV3E). Wild-type and KO cells were co-transfected with the EBS reporter, and luciferase signals were subsequently determined. In comparison to WT cells, GR-KO clones displayed relatively low EBS signals (Fig. EV4C), but transfection of GR partly rescued the luciferase signal, in line with the proposed model. Since GR and ERG form a physical complex and each component of the complex binds with distinct DNA response elements, we next asked if ERG would transactivate, or trans-repress, transcription from the GRE. To examine this, we transfected a GRE luciferase reporter, along with increasing amounts of an ERG plasmid. As expected, control experiments using an ectopically expressed GR showed enhanced transcription from the GRE (Fig. EV4C). Interestingly, however, the ectopically expressed ERG inhibited, rather than enhanced, basal expression from the GRE. This unexpected inhibitory effect was confirmed by another experiment that detected consistently reduced effects of GR on transcription from the GRE when ERG was overexpressed (Fig. EV4D). Conceivably, opposite to the stimulatory effect of GR on transcription from the EBS, ERG reciprocally induces an inhibitory effect on transcription from the GRE.

### Transactivation by GR involves alleviation of an ERG allosteric inhibitory mechanism and enables GR to enhance transcription from the ERG’s DNA-binding site

Previous analyses uncovered an intrinsic allosteric mechanism that autoinhibits ERG’s binding to DNA (Regan et al, 2013). Accordingly, two short segments of ERG, the N- and C-terminal inhibitory domains (NID and CID, respectively), each flanking the DNA-binding region (ETS; see scheme in Fig. 4D), regulate intramolecular interactions. Our finding that GR engages the DNA-binding domain of ERG raised the possibility that GR can alleviate autoinhibition when in complex with ERG. To test this model, we utilized an HA-tagged double deletion mutant of ERG, ∆NID&CID, lacking both autoinhibitory domains. Control immunoprecipitation assays indicated that the double deletion mutant lost the ability to bind with GR (Fig. 4D). Next, increasing doses of plasmids encoding the mutant or the wild-type forms of ERG were expressed in hormone-starved cells, along with the EBS-luciferase reporter (Fig. 4E). As predicted, treatment of WT-ERG-expressing cells with DEX was associated with increasing luciferase signals. In contrast, although the signals observed with the ∆NID&CID double mutant were higher, cell treatment with DEX did not further increase transcription. These observations are consistent with the autoinhibition model, and they support the possibility that GR can directly or allosterically abolish the autoinhibitory mechanism of ERG.

### Chromatin immunoprecipitation and RNA sequencing confirm the ability of GR to enhance transcription from the ERG’s DNA-binding site

Having shown that GR can transactivate transcription from the EBS, we next applied chromatin immunoprecipitation (ChIP) to determine the genome-wide effect of GR on the ability of tERG to bind with chromatin. Prior to ChIP analysis, CSS (charcoal-stripped serum) grown VCaP and DU145-tERG cells were treated with DEX. Consistent with the promoter reporter assays, DEX stimulation increased by >60% the average ERG’s ChIP-seq signal in both VCaP and DU145-tERG cells (Fig. 4F; note that the full results were deposited and they are available at Omnibus GSE247347). As expected, de novo motif analysis of ERG (82,360 binding sites) and GR binding to chromatin (23,745 sites) in DEX-stimulated VCaP cells detected high enrichment for the ETS and GRE motifs (Fig. EV4E). Interestingly, most GR’s chromatin binding sites (21,706 of 23,745) were co-occupied by ERG in DEX-stimulated VCaP cells. Analysis of the de novo DNA motifs found that EBS topped the list of motifs shared by ERG and GR (Fig. 4G). To investigate whether the global chromatin binding sites of ERG and GR vary in different cell lines, we similarly analyzed the ERG (20,444) and GR (32,986) sites in DEX-treated DU145-tERG cells. This analysis found that 43% of the GR binding sites were shared by ERG (Fig. EV4F). In addition, the DNA-binding motifs of ERG (i.e., EBS) and GR (GRE) were highly enriched within the overlapping 14,218 ERG and GR sites. Examples of the integrated genome view (IGV) tracks corresponding to *MYC* and *NKX3-1*, which are regulated by ERG and GR, are shown in Fig. 4H. RNA-sequencing and PCR analyses performed with VCaP and DU145-tERG cells complemented the ChIP-seq analysis (Figs. 4I and EV4G,H; full data deposited at Omnibus GSE247422). Evidently, DEX treatment enhanced expression of several bona fide targets of ERG, including *MYCL*, *NKX3-1*, *CCND1*, *FZD4*, *CDH1*, *KLF4*, *ANGPTL4*, and *ADAMTS7* (see Appendix Table S2). In contrast to DU145-tERG and VCaP cells, we observed no changes in ERG target genes in the control, ERG-low DU145-EV cells (Fig. EV4G). Still, known GR target genes (e.g., *FKBP5* and *DUSP1*) were upregulated following stimulation of these cells with DEX. Consistent with these observations, gene set enrichment analysis (GSEA) uncovered enrichment for PCa-related pathways, as well as MYC-target genes, upon treatment of VCaP cells with DEX (Fig. 4J). Taken together, these results unraveled genome-wide co-occupancy of specific sites by ERG and GR, as well as demonstrated transactivation of ERG target genes by GR.

### Inhibition of GR selectively decreases the growth and survival of *TMPRSS2-ERG*-positive cells

**Figure 5:**
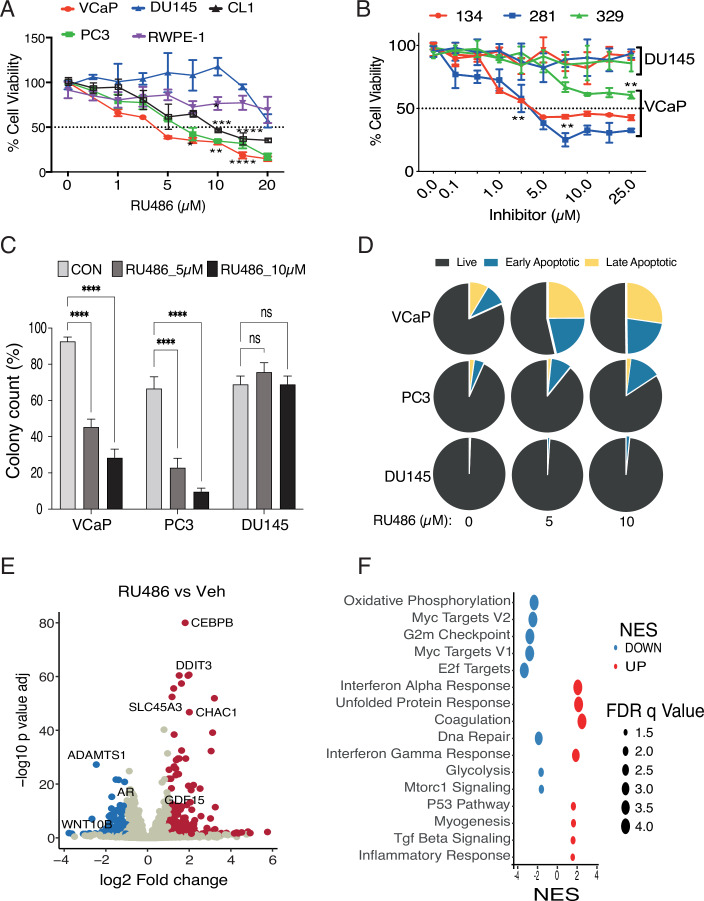
Inhibition of GR specifically decreases growth and survival of tERG-positive PCa cells (see also Fig. EV5). (**A**) The XTT cell viability assay was performed with the following prostate cell lines: VCaP (ERG + ), PC3 (ETV4 + ), CL1 (ETV1 + ), DU145 (ERG-low), and RWPE-1 (normal prostate epithelial cells). All cell lines were treated in triplicate for 96 h with increasing concentrations of RU486. Statistical analysis was performed using two-way ANOVA with Tukey’s multiple comparisons test. We performed the experiments at least twice with triplicate (VCaP, DU145, RWPE-1, CL1) or duplicate (PC3). Data are presented as means ± SEM. Statistical significance is denoted as *P < 0.05; **P < 0.01; ***P < 0.001, and ****P < 0.0001. (**B**) VCaP and DU145 cells were treated for 96 h with increasing concentrations of the indicated non-steroidal inhibitors of GR, and later subjected to cell viability assays, as in (**A**). Data are presented as mean ± SEM (*n* = 2 or 3). Statistical analysis was performed using two-way ANOVA with Tukey’s multiple comparisons test. The experiment was performed twice with two biological replicates, and representative results are shown. (**C**) VCaP, PC3, and DU145 cells were sparsely seeded in 6-well plates and later untreated or treated on every other day with either vehicle or RU486 (5 or 10 µM). Ten days later, all cells were fixed and stained with crystal violet. The bar plots present quantification of colonies observed in 5 non-overlapping microscope fields. Data are presented as mean ± SEM (*n* = 4). Statistical analysis was performed using one-way ANOVA with Tukey’s multiple comparisons test. *P < 0.05; **P < 0.01; ***P < 0.001; ****P < 0.0001. (**D**) The indicated cell lines were seeded in 100-mm dishes. Thereafter, they were treated for 72 h with the vehicle (CON) or RU486 (1 μM). Shown are the results of an apoptosis assay performed using an annexin V/7-AAD kit (from BioLegend). (**E**) VCaP cells were treated for 24 h with RU486 (1 μM) or with the corresponding vehicle, and RNA was extracted for RNA-seq analysis. The volcano plot shows the differentially expressed genes. Significantly up- or downregulated genes are colored in red and blue, respectively. The ERG target genes and the enriched apoptotic pathway genes are marked in the plots. (**F**) The presented pathway analysis of differentially expressed genes (DEGs) used EnrichR and selected genes with a fold change >1, or < -1, and an adjusted *P* value smaller than 0.05. The most significantly enriched pathways from MSigDB Hallmark 2020 are depicted. See Appendix Table S3. Source data are available online for this figure.

**Figure EV5:**
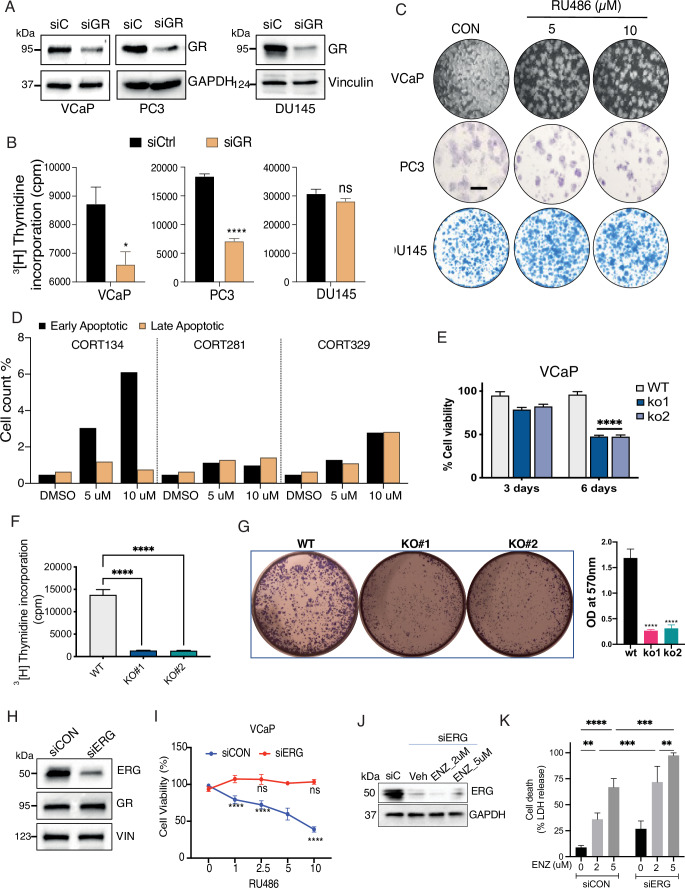
Both inhibition and genetic ablation of GR associate with decreased proliferation of PCa cells expressing ETS fusions (related to Fig. 5). (**A**, **B**) VCaP, PC3, and DU145 cells were transfected with either GR-specific (siGR) or control (scrambled) siRNAs (siCON). Knockdown efficiency was monitored after 48 h using immunoblotting with antibodies to GR. Cell proliferation was measured 72 h later by applying a radioactive thymidine incorporation assay. Statistical analysis was performed using an unpaired *t* test. The experiment was repeated twice and performed with biological duplicates. Note that representative results are shown. Error bars represent mean ± SEM. *P < 0.05; ****P < 0.0001; ns, not significant. (**C**) Shown are representative images corresponding to the colony formation assay and the bar plots shown in Fig. 5. Scale bar, 0.1 mm. (**D**) VCaP cells were seeded in 100-mm dishes. Thereafter, they were treated for 72 h with the vehicle (DMSO; CON) or with the indicated non-steroidal GR antagonists. Shown are the results of an apoptosis assay performed in duplicates using an annexin V/7-AAD kit (from BioLegend). (**E**) GR-knockout VCaP cells were seeded in 96-well plates, and cell viability was measured using the XTT colorimetric assay following 3 or 6 days of incubation. Statistical analysis was performed using unpaired *t* test. The experiment was performed with two biological replicates, and representative results are shown. Data are presented as means ± SEM. (**F**) DNA replication by GR-KO VCaP cells (2 clones) was measured using the thymidine incorporation assay. Statistical analysis was performed using unpaired *t* test. The experiment was repeated twice and performed with two biological replicates; representative results are shown. Data are presented as means ± SEM. (**G**) WT and GR-KO VCaP cells were sparsely seeded in six-well plates. Fifteen days later, cells were fixed and stained with crystal violet. Photos are shown along with bar plots presenting the quantification of colonies. Statistical analysis was performed using unpaired *t* test. The experiment was performed twice with two biological replicates. Data are presented as means ± SEM. ****P < 0.0001. (**H**, **I**) VCaP cells were transfected with either control oligonucleotides or with siRNAs targeting ERG. Forty-eight hours post transfection, the cells were harvested and subjected to immunoblotting. Alternatively, siCON- or siERG-transfected cells were seeded in 96-well plates and treated with RU486 for 48 h. Cell viability was measured using the XTT assay after 48 additional hours. Statistical analysis was performed using two-way ANOVA with Sidak’s multiple comparisons test. The experiment was performed with two biological replicates, and representative results are shown. Data are presented as means ± SEM. ****P < 0.0001; ns, not significant. (**J**) VCaP cells pre-transfected with siERG or siControl were treated with enzalutamide (2 and 5 μM) for 48 h and subjected to western blot analysis for ERG and GAPDH. (**K**) Shown are the results of LDH cytotoxicity assays measuring cell death in siControl and siERG-transfected VCaP cells treated with enzalutamide. Statistical analysis was performed using two-way ANOVA with Sidak’s multiple comparisons test. The experiment was performed twice with two biological replicates; representative results are shown. Data are presented as mean ± SEM. **P < 0.01; ***P < 0.001. Note that the exact *P* values, statistical tests, sample sizes, and error bar definitions for all panels are provided in Appendix Table S3.

tERG-overexpressing PCa cells often rely on the fused gene for survival and proliferation (Khosh Kish et al, 2022; Lorenzin and Demichelis, 2022). However, the relative contribution of the GR-ERG protein complex remains unknown. Hence, we treated with RU486 a panel of prostate cells, which included RWPE, a normal prostate epithelial line, the tERG-expressing VCaP cells, PC3 cells, which express ETV4, and the tERG-low DU145 cells. The results showed that, unlike the tERG-low/negative lines (DU145 and RWPE-1), the tERG-positive cells were sensitive to RU486 (Fig. 5A). Similarly, applying non-steroidal GR antagonists, instead of RU486, confirmed inhibition of the ETS-positive line, VCaP, but DU145 cells were not affected (Fig. 5B). In addition, we asked if inhibition of GR using RNA interference would similarly retard cell proliferation. GR was downregulated in VCaP, PC3, as well as in the tERG-low DU145 cells, using GR-specific siRNAs (Fig. EV5A). This led to significant inhibition of the ability of VCaP and PC3 cells to incorporate a radioactive nucleoside into chromosomal DNA during mitosis (Fig. 5B). In contrast, we observed no significant inhibitory effect when DU145 cells (tERG-low) were treated with GR-specific siRNAs. Hence, we concluded that both pharmacological and genetic inhibition of GR can lead to reduced growth of ETS-positive PCa cells. Similar conclusions were reached on the basis of colony formation assays performed with VCaP, DU145, and PC3 cells (Figs. 5C and EV5C). In line with these observations, we noted that treatment with RU486, or with non-steroidal GR antagonists, increased to a variable extent both early and late apoptosis of ETS-positive cells (Fig. 5D; Fig. EV5D). In similarity to the other assays, when tested on DU145 cells, RU486 induced no detectable apoptosis. Next, we applied gene ablation and RNA interference as alternative strategies to address the function of the GR-ETS complex: in comparison to wild-type cells, GR-knockout VCaP cells displayed lower cell viability (Fig. EV5E), DNA synthesis (Fig. EV5F), and colony formation (Fig. EV5G). Likewise, targeting ERG using specific siRNA nucleotides revealed that downregulation of *tERG* abolished cellular sensitivity to RU486 (Fig. EV5H,I) but sensitized cells to enzalutamide (Fig. EV5J,K). In conclusion, inhibition of either component of the GR-tERG complex can impair the growth of several different PCa cells, implying that a substantial fraction of the mitogenic activity of tERG might be attributable to the formation of GR-tERG complexes.

To uncover transcriptional programs that may underlie the action of the GR-tERG complex, we treated VCaP cells with RU486, performed RNA-seq analysis (Fig. 5E), and listed the pathways regulated by the differentially expressed genes (DEGs; Fig. 5F). This analysis identified the most significantly enriched pathways according to MSigDB Hallmark 2020 (Liberzon et al, 2015). As expected, the list included several previously reported ERG target genes, which were downregulated, along with apoptosis pathway genes that were upregulated by RU486. For example, C/EBP-beta, which mediates the expression of several interferon gamma-regulated genes, was upregulated in RU486-treated cells. Conversely, this drug inhibited the expression of amphiregulin, a growth factor, and WNT10B, a canonical WNT ligand that controls malignant propensity. Likewise, we observed downregulation of genes regulating cell cycle progression (i.e., the E2F pathway), along with several *MYC* targets. The latter, as well as AR, FOXA1, and ERG, drive PCa cell proliferation (Xiao et al, 2022). Taken together, these observations imply that GR cooperates with ERG by augmenting cell proliferation and overcoming apoptosis, while harnessing the major TFs of PCa.

### Akin to reducing cortisol levels, GR antagonism selectively inhibits TMPRSS2–ERG-positive cell lines and corresponding xenograft models

**Figure EV6:**
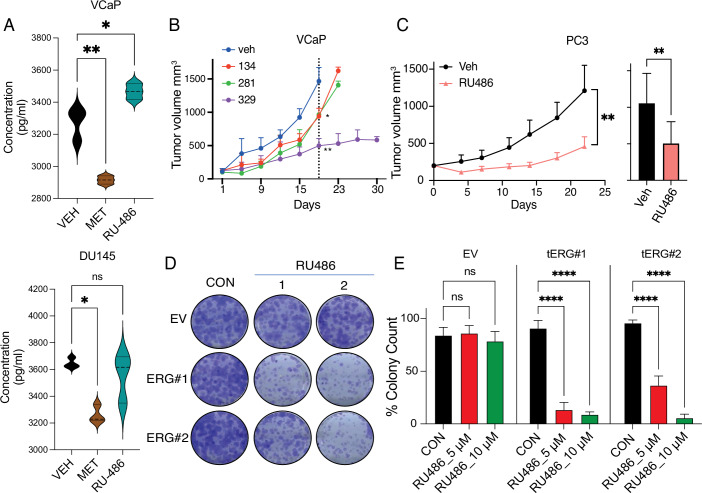
GR inhibition suppresses the growth of ERG-expressing PCa cells, whereas cells lacking tERG show no response to the treatment (see Fig. 6). (**A**) Corticosterone concentrations (pg/ml) were measured in tumor tissues from Fig. 6A,B. Tumors were processed in parallel, and equal amounts of total protein were analyzed using the DetectX® Corticosterone Enzyme Immunoassay Kit. Data are presented as violin plots with corticosterone concentration and 3 tumors per group. Statistical analysis was performed using one-way ANOVA with Dunnett’s multiple comparisons test. *P < 0.05; **P < 0.01; n.s., not significant. (**B**) VCaP cells (2 × 10⁶) were implanted subcutaneously in athymic mice. Once tumors became palpable, animals were randomized into four groups (3 animals per group), which were daily treated with vehicle or with the indicated non-steroidal GR antagonists (50 mg/kg). The rates of tumor growth are shown. Statistical analysis was performed using two-way RM ANOVA with Dunnett’s multiple comparisons test. There were three animals per group. Data are presented as means ± SEM. *P < 0.05; **P < 0.01. (**C**) PC3 cells (5 × 10⁶) were implanted in animals, which were randomized into groups that were daily treated with vehicle or with RU486 (1 mg/kg). The rates of tumor growth (left panel), along with tumor volumes on day 22 (bar plot), are shown. Statistical analysis was performed using unpaired *t* test. The following numbers of mice were used per group: Vehicle = 8, RU486 = 9. Data are presented as mean ± SEM. **P < 0.01. (**D**, **E**) Two clones of DU145 cells stably expressing tERG were sparsely seeded in six-well plates. Cells were later treated once every other day with either vehicle or RU486 (5 µM and 10 µM). Ten days later, all cells were fixed and stained with crystal violet. Representative photos are shown along with bar plots presenting the quantification of colony numbers in five non-overlapping microscope fields. The experiment was repeated twice. Statistical analysis was performed using one-way ANOVA with Dunnett’s multiple comparisons test. The number of fields per condition was 5. Data are presented as means ± SEM. ****P < 0.0001; ns, not significant. Note that the exact *P* values, statistical tests, sample sizes, and error bar definitions for all panels are provided in Appendix Table S3.

**Figure 6:**
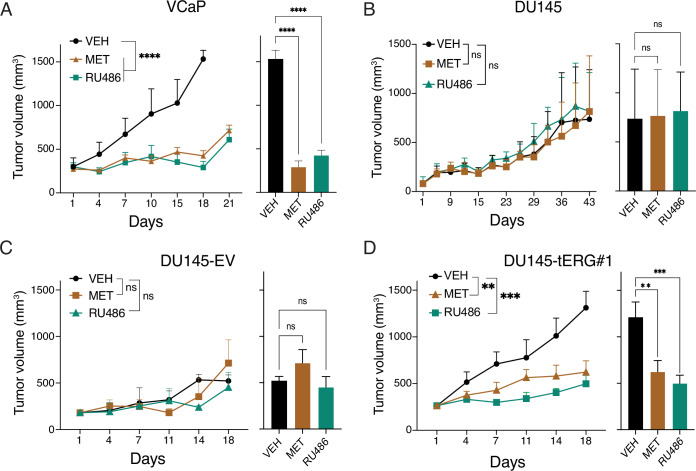
Both antagonizing GR and lowering cortisol inhibit TMPRSS2-ERG+ xenograft models of PCa (see also Fig. EV6). (**A**, **B**) VCaP cells (2 × 10⁶) and DU145 cells (5 × 10⁶) were implanted subcutaneously in athymic mice. Once tumors became palpable, animals were randomized into three groups (5-6 animals per group), which were treated daily with vehicle, RU486 (1 mg/kg), or metyrapone (25 mg/kg). The rates of tumor growth are presented (left panels), along with the average tumor volumes on day 21 (VCaP) or day 43 (DU145), which are presented in the right part of each panel. (**C**, **D**) DU145 cells stably expressing tERG (5 × 10⁶) and control DU145 cells (EV, 5 × 10⁶) were pre-established. Cells were implanted subcutaneously in nude mice and, once tumors became palpable, animals were randomized into three groups, which were treated daily with vehicle, RU486 (1 mg/kg), or metyrapone (25 mg/kg). The bar graphs present tumor volumes corresponding to day 18. The following number of animals were used: *n* = 2–3 (DU145-EV: Veh=2, Met=3, RU486 = 2) and *n* = 5–6 (DU145-tERG: Veh=5, RU486 = 6, Met=5) animals per group. Data are shown as means ± SEM. Statistical analysis was performed using one-way ANOVA with Dunnett’s multiple comparisons test. **P < 0.01; ***P < 0.001; ****P < 0.0001; ns, not significant. Exact *P* values, statistical tests, sample sizes, and error bar definitions for all panels are provided in Appendix Table S3. Source data are available online for this figure.

To examine the translational significance of the direct GR-to-tERG interactions, we treated three different PCa xenografts with two clinically approved anti-GR drugs: RU486 (mifepristone) and metyrapone (Fleseriu and Petersenn, 2015). In addition, we used relacorilant (CORT125329), a non-steroidal selective GR antagonist. Initial experiments tested the effects of the approved drugs on cortisol levels within VCaP and DU145 xenografts. The results confirmed a lowering effect of metyrapone but the effect of RU486 was less clear (Fig. EV6A). These observations are in line with the respective mechanisms of action: while metyrapone blocks the final step of cortisol biosynthesis, RU486 acts as a competitive GR antagonist. As shown in Figs. 6A and EV6B, treatments making use of RU486, metyrapone, and relacorilant clearly inhibited growth of the tERG-positive VCaP xenografts. In addition, PC3 cells that naturally overexpress another ETS protein, ETV4, were similarly inhibited by RU486 (Fig. EV6C).

In contrast with VCaP and PC3 xenografts, neither RU486 nor metyrapone inhibited the growth of the DU145 cells (Fig. 6B), which lack the TMPRSS2–ERG gene fusion and do not express ERG at the mRNA or protein level. These observations led us to test in vitro the drug sensitivities of two clones of tERG-overexpressing DU145 cells. As expected, treatment with RU486 significantly reduced colony formation by the tERG-overexpressing clones, but growth of control cells pre-transfected with an empty vector (EV) was not inhibited by the drug (Fig. EV6D,E). Consistent with the in vitro data, both the parental and the control DU145-EV cells displayed no response to RU486, but a tERG-overexpressing clone was significantly inhibited when tested in vivo (Fig. 6C,D). In summary, blocking GR signaling using both steroidal and non-steroidal GR antagonists, as well as a blocker of cortisol synthesis, inhibited the growth of tERG-positive tumors, but the ERG-negative PCa models we employed displayed a lack of response.

### Combining blockers of AR and GR selectively inhibits tERG-positive xenografts derived from patients with PCa

**Figure EV7:**
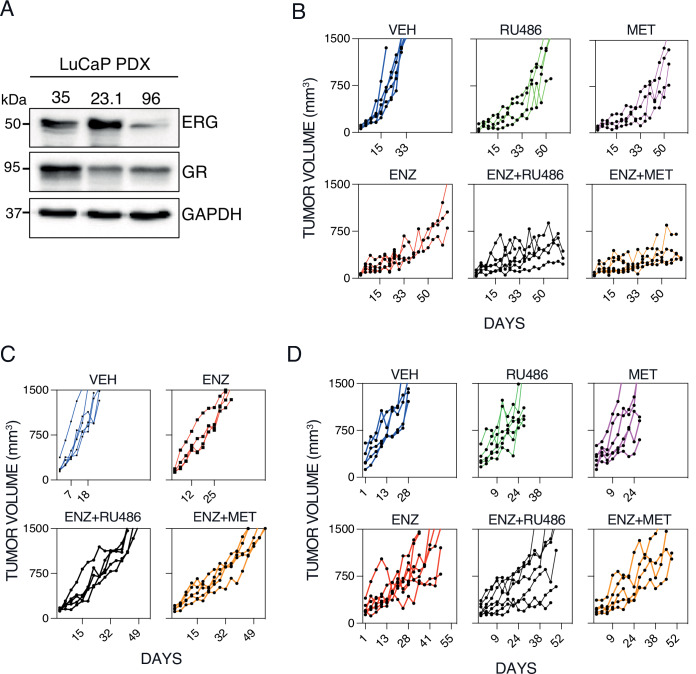
Growth rates of three different PDX models in individual mice treated with combinations of GR and AR antagonists (related to Fig. 7). (**A**) Whole extracts of the indicated PDX models were subjected to immunoblotting for ERG and GR. GAPDH was used to control gel loading. (**B**) Growth curves of individual tumors derived from the tERG-positive LuCaP 23.1 PDX model. Each line corresponds to one animal. (**C**) Growth curves of individual tumors derived from the tERG-positive LuCaP 35 PDX model. (**D**) Growth curves of individual tumors derived from the tERG-negative LuCaP 96 PDX model.

**Figure 7:**
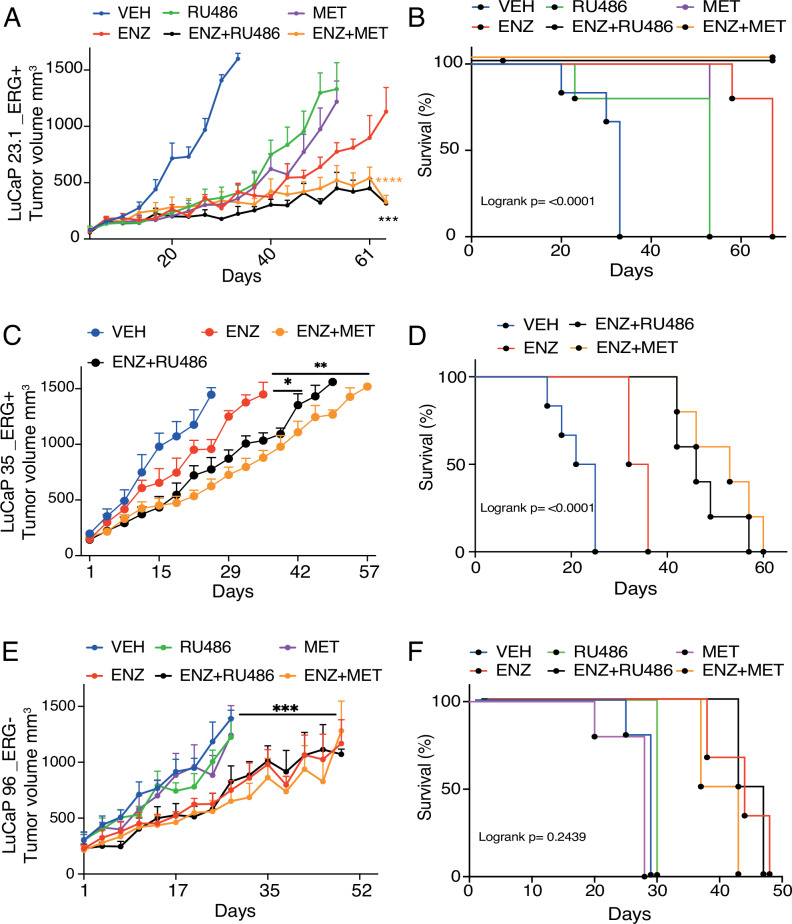
Combinations of GR and AR inhibitors selectively retard tERG-positive xenografts derived from patients with prostate cancer (see also Fig. EV7). (**A**, **B**) Fragments of the tERG-positive LuCaP 23.1 PDX model were subcutaneously implanted in the flanks of 4–6 NSG mice. Once tumors reached 150 mm³, animals were randomized to the indicated groups, which were daily treated with enzalutamide (ENZ; 10 mg/kg, oral gavage), RU486 (1 mg/kg, intraperitoneal injection), metyrapone (MET; 25 mg/kg, oral gavage), or with the indicated drug combinations. Tumor volumes were monitored twice per week, and body weights were recorded once per week. Shown are average tumor growth rates of mice treated with single drugs or with the indicated dual drug combinations. Also shown are Kaplan–Meier plots corresponding to survival analyses. Note that mice were euthanized when the tumor size reached 1500 mm³. Animal numbers per group were as follows: *n* = 6 (Vehicle), *n* = 6 (ENZ), *n* = 4 (RU486), *n* = 4 (MET), *n *= 6 (ENZ + MET), *n* = 6 (ENZ + RU486). Survival analysis used the log-rank (Mantel–Cox) test. (**C**, **D**) Fragments of the tERG-positive LuCaP 35 PDX model were subcutaneously implanted in the flanks of NSG mice and treated as in (**A**). The Kaplan–Meier plots present the respective animal survival data. The number of animals per experimental arm are as follows: *n* = 6 (Vehicle), *n* = 4 (ENZ), *n* = 5 (ENZ + RU486), *n* = 5 (ENZ + MET). Survival analysis used the log-rank (Mantel–Cox) test. (**E**, **F**) Fragments of the tERG-negative LuCaP 96 PDX model were subcutaneously implanted in the flanks of NSG mice and treated as in (**A**). The Kaplan–Meier plots present the respective animal survival data. The numbers of animals per experimental arm were as follows: *n* = 6 (Vehicle), *n* = 7 (ENZ), *n* = 4–6 (RU486), *n* = 5 (MET), *n* = 6 (ENZ + MET), *n* = 5 (ENZ + RU486) animals per group. Data are mean ± SEM. Statistical analysis was performed using one-way ANOVA with Dunnett’s multiple comparisons test. *P < 0.05; **P < 0.01; ***P < 0.001; ****P < 0.0001; ns, not significant. Exact *P* values, statistical tests, sample sizes, and error bar definitions for all panels are provided in Appendix Table S3. Source data are available online for this figure.

In the next step, we examined in mice the potential benefit of combining GR inhibitors and enzalutamide, a synthetic non-steroidal AR antagonist. These experiments employed three patient-derived xenografts (PDX): two tERG-positive models (LuCaP 23.1 and LuCaP 35) and a tERG-negative PDX model (LuCaP 96) (Navone et al, 2018). Comparative analysis of the three PDX models confirmed very low ERG abundance in LuCaP 96 (Fig. EV7A). Fragments of the LuCaP 23.1 model were implanted in NSG mice, which were later randomized into several groups that were treated with enzalutamide, RU486, or metyrapone. Daily treatments with enzalutamide, for 6 weeks, inhibited tumor growth, but a few weeks later all tumors adopted the rapid growth typical to the control untreated mice (Figs. 7A and EV7B; see survival curves in Fig. 7B). Similarly, the RU486-treated group and the metyrapone-treated animals displayed delayed emergence of resistance. Importantly, however, combining enzalutamide and either GR-targeting drug resulted in monotonic, relatively slow tumor growth, which significantly prolonged animal survival.

The other ERG-positive PDX model, LuCaP 35, displayed more aggressive growth and only weak response to enzalutamide monotherapy (Figs. 7C and EV7C). Still, combining enzalutamide and either RU486 or metyrapone partly and significantly inhibited tumor growth, as well as prolonged animal survival (Fig. 7C,D). Interestingly, the efficacies achieved by each pair of drugs were comparable and significantly better than the effect of enzalutamide monotherapy. In conclusion, both ERG-positive PDX models we tested clearly displayed sensitivity to the doublet combinations of enzalutamide and an anti-GR drug. As expected from the in vitro data and from the results obtained with xenografts of the ERG-low DU145 cell line, the third PDX model, LuCaP 96, which is tERG-negative and expresses only low ERG levels, displayed resistance to anti-GR drugs (Figs. 7E,F and EV7D). Although this model partly responded to enzalutamide, neither anti-GR drug could enhance enzalutamide’s effect in terms of tumor growth and animal survival. Hence, in view of the herein reported physical interaction between ERG and GR, the observations made in tumor-bearing animals indicate that simultaneously targeting both components of the GR-tERG complex has an added pharmacological value that is limited to fusion-positive tumors.

In conclusion, analysis of patient data from two clinical trials suggested that the presence of *TMPRSS2-ERG* (tERG) favors resistance to ARSIs and initiates drug-induced upregulation of GR. To resolve the underlying mechanism, we performed protein complementation assays and additional tests. These studies revealed that the glucocorticoid receptor physically interacts with tERG, relieving its intrinsic autoinhibition and protecting it from both basal and chemotherapy-induced degradation. As a result, GR boosts activation of tERG target genes, including the MYC proto-oncogene. Critically, this mechanism is absent in tERG-negative tumors, which are more responsive to ARSIs. Consistent with the idea that GR–tERG complexes confer distinct vulnerabilities, blockade of GR or suppression of cortisol synthesis induced apoptosis and inhibited proliferation in tERG-positive prostate cancer cells, while exerting only modest effects on tERG-negative cells. In vivo, tERG-positive tumor models showed enhanced antitumor efficacy when antiandrogens were combined with inhibitors of GR signaling. Collectively, by establishing GR as a key oncogenic partner of tERG, this work reveals new therapeutic opportunities to counteract drug resistance and improve outcomes in a substantial subset of prostate cancer patients.

## Discussion

Two decades ago, it was discovered that approximately every other patient with PCa harbors tumors expressing fusions between *TMPRSS2* and an *ETS* family gene (Tomlins et al, 2005). However, unlike other fusion genes and activated mutant oncogenes like BCR-ABL and B-RAF, which are targetable by several anti-cancer drugs, no clinically approved drug targets TMPRSS2-ETS, and the presence of the rearranged genes has not been translated to predictive biomarkers. The clinical data analyses we present confirmed a bimodal distribution of untreated PCa tumors: those harboring *TMPRSS2-ERG* displayed much higher expression of ERG, but furthermore the respective group of patients exhibited statistically significant resistance to treatments that made use of antiandrogens. As far as we are aware, this is the first demonstration of the ability of *TMPRSS2-ERG* to confer resistance to antiandrogens. Because the resistant patients displayed relatively high GR levels and previous studies established critical roles for GR in resistance to antiandrogens (Arora et al, 2013; Narayanan et al, 2016), our search for the underlying mechanism focused on GR and its previously reported ability to form complexes with several ETS family members (Srivastava et al, 2019). Protein complementation and additional assays revealed that GR directly interacts with ERG, thereby it alleviates ERG’s allosteric autoinhibition, as well as stabilizes ERG and boosts transactivation of the respective target genes. Thus, although *TMPRSS2-ERG* gene fusions emerge at relatively early stages of PCa development (Perner et al, 2007) and they are associated with aggressive phenotypes, our results portray the respective tumors as vulnerable cancers due to acquired dependence on several onco-proteins: AR and GR in the first place, but also HSP90, which controls both receptors, and tERG, which is controled by androgens. The dependence on GR becomes especially critical when the AR pathway is blocked: normally, AR suppresses GR (Shah et al, 2017), but following treatment with antiandrogens, GR undergoes upregulation and provides an escape route that engages most of the AR target genes. The inferred mechanism explains why our tERG-positive xenografts displayed sensitivity to combinations of GR and AR blockers, but the tERG-negative animal models we utilized were significantly less responsive.

Notably, because GR can circumvent AR inhibition and sustain tumor growth, targeting GR represents a potential strategy to restore sensitivity to antiandrogen therapy. Nevertheless, a clinical trial evaluating ORIC-101—an orally bioavailable small-molecule GR antagonist—in metastatic castration-resistant prostate cancer reported no meaningful antitumor activity in an unselected mCRPC population progressing on enzalutamide (Abida et al, 2024). Our results suggest that enriching for tERG-positive patients may reveal clinical benefit from GR-targeting agents such as ORIC-101. Beyond the implications for resistance to antiandrogens, our study is relevant to the application of corticosteroids in PCa treatment. Although most castration-resistant prostate cancer patients benefit from abiraterone plus prednisone, resistance eventually emerges. However, this may be reversed by switching to the longer-lived corticosteroid, dexamethasone (Romero-Laorden et al, 2018; Teo and Scher, 2018; Yang et al, 2021). Likewise, daily oral corticosteroids are routinely combined with abiraterone to mitigate symptoms of mineralocorticoids (Ndibe et al, 2015) and, similarly, prednisone augments the efficacy of docetaxel in patients with PCa (Teply et al, 2016). However, according to our observations, corticosteroids might accelerate disease progression only in patients expressing a fused form of an ETS family member. In line with this scheme, high GR expression is associated with an increased risk of disease progression in several carcinomas (Bakour et al, 2021) and treatment of castration-resistant PCa cells with AR inhibitors frequently involves upregulation of GR, which stimulates a subset of AR-regulated genes (Arora et al, 2013; Puhr et al, 2018).

Remarkably, the uncovered ability of hormone-activated GR molecules to bind with and enhance the oncogenic activity of tERG might explain the recently reported post hoc analysis of the AFFIRM phase III clinical trial (NCT00974311). This study randomized 1199 patients to enzalutamide or placebo and allowed corticosteroids at study entry and during the trial. Along with confirming the clinical efficacy of enzalutamide, the new study addressed the clinical impact of concurrent corticosteroid use (CCU) on enzalutamide-treated patients with metastatic disease (Zhao et al, 2022). Unexpectedly, a multivariate analysis found that baseline CCU was independently associated with decreased overall patient survival. Interestingly, an earlier post hoc analysis of another clinical trial, COU-AA-301, which tested abiraterone plus prednisone versus prednisone in metastatic PCa patients made a similar observation but reached a different conclusion (Montgomery et al, 2015): Although in similarity to the AFFIRM data, COU-AA-301’s data found that baseline corticosteroids were associated with shorter overall patient survival, the authors concluded that this inferiority was due to an association between the use of baseline corticosteroid and patients having worse disease characteristics. In aggregate, the reviewed lines of evidence raise the possibility that corticosteroid therapy may be contraindicated in PCa patients with tumors expressing an ETS fusion gene.

In conclusion, our in vivo and other observations identify a protein complex comprising GR and tERG as a driver of resistance to antiandrogens. In addition, our results shed light on the underlying molecular mechanism: blocking AR elevates GR, which directly boosts the oncogenic attributes of tERG. Thus, GR agonists like DEX and prednisone might accelerate the progression of prostate tumors expressing tERG. From the medical point of view, these molecular mechanisms may translate to a new predictive biomarker and a recommendation to limit the use of CCU to patients with tERG-negative PCa. Thus, the reported findings might motivate the development of new anti-GR drugs, such as small molecules sharing the ability to intercalate into the newly identified GR-ERG cleft. Alternatively, if validated by additional studies, our observations might justify the development of next-generation GR antagonists and AR-GR dual antagonists.

## Methods

**Table:** Reagents and tools table

Reagent or resource	Source	Identifier
**Antibodies**		
Normal Rabbit IgG	Cell Signaling Technology	Cat# 2729
ERG (A7L1G), Rabbit monoclonal	Cell Signaling Technology	Cat#97249
Erg-1/2/3 (D-3), Mouse monoclonal	Santa Cruz Biotechnology	Cat#sc271048
GR (G-5), Mouse monoclonal	Santa Cruz Biotechnology	Cat# sc-393232; RRID: AB_2687823
GR, Rabbit polyclonal	Santa Cruz Biotechnology	Cat# sc-8992; RRID: AB_2155784
ETV4, Rabbit Polyclonal	Thermo Fisher Scientific	Cat# PA5-13595
GAPDH, Mouse monoclonal	Millipore	Cat# MAB374
Gaussia-luciferase, Rabbit polyclonal	Nanolight Technology	Cat # 401 P
HA tag, Rabbit monoclonal	Cell Signaling Technology	Cat#3724
Vinculin, Mouse monoclonal	Sigma-Aldrich	Cat# V9264
Flag tag, Rabbit Polyclonal	Proteintech	Cat#20543-1-AP
HA tag, Rabbit Polyclonal	Proteintech	Cat#51064-2-AP
Phospho-Chk1 (Ser345), Rabbit Polyclonal	Cell Signaling Technology	Cat#2341
Chk1 (2G1D5), Mouse monoclonal	Cell Signaling Technology	Cat#2360
Phospho-Wee1 (Ser642) (D47G5), Rabbit monoclonal	Cell Signaling Technology	Cat#4910
Gamma-H2AX (S139), Rabbit monoclonal	Cell Signaling Technology	Cat#2577
Histone 3, Rabbit polyclonal	Abcam	Cat#AB1791
LDH, Goat Polyclonal	Santa Cruz Biotechnology	Cat#SC27230
Anti-mouse Alexa Fluor 555-conjugated secondary antibody	Thermo Fisher Scientific	Cat#A31572
Anti-rabbit Alexa Fluor 488-conjugated secondary antibody	Thermo Fisher Scientific	Cat#A21202
**Biological samples**		
Patient-derived xenograft (PDX) LuCaP 23.1	Prof. Eva Corey, University of Washington, Seattle, Washington.	N/A
Patient-derived xenograft (PDX) LuCaP 35	Prof. Eva Corey, University of Washington, Seattle, Washington.	N/A
Patient-derived xenograft (PDX) LuCaP 96	Prof. Eva Corey, University of Washington, Seattle, Washington.	N/A
**Chemicals, peptides, and recombinant proteins**		
Dexamethasone	Sigma-Aldrich	Cat# D4902
RU486	Sigma-Aldrich	Cat# M8046
Metyrapone	Sigma-Aldrich	Cat# M2696
Enzalutamide	MedChem express	Cat# HY-70002
Coelentrazine	Nanolight Technology	Cat#303
Cycloheximide solution	Sigma-Aldrich	Cat# C4859
Geldanamycin	MedChem express	Cat# HY-15230
MG-132	MedChem express	Cat# HY-13259
Blasticidin	Sigma-Aldrich	Cat# SBR00022
Cultrex® RGF BME	R&D System	Cat# 3433-005-02
SYBR Green PCR Master Mix	Thermo Fisher Scientific	Cat# 4309155
^3^[H]-thymidine	Perkin Elmer, USA	Cat#NET027Z001MC
CORT125134 (C134, relacorilant)	Corcept Therapeutics, USA	N/A
CORT125281 (C281, exicorilant)	Corcept Therapeutics, USA	N/A
CORT125329 (C329)	Corcept Therapeutics, USA	N/A
Camptothecin (CPT)	MedChem express	Cat# HY-16560
**Critical assays and kits**		
Dual-Luciferase Assay kit	Promega	Cat#E1910
Cell Proliferation Kit (XTT based)	Biological Industries	Cat# SKU: 20-300-1000
FITC Annexin V Apoptosis Detection Kit	BD PharMingen	Cat#556547
**Deposited data**		
Raw and analyzed RNA seq data	This paper	GSE247422
Raw and analyzed ChIP seq data	This paper	GSE247347
**Experimental models: Cell lines**		
VCaP	ATCC	Cat#CRL-2876
PC3	Gift from Prof. Haim Werner, Tel Aviv University, Israel	N/A
CL1	Gift from Prof. Haim Werner, Tel Aviv University, Israel	N/A
DU145	ATCC	Cat#HTB-81
RWPE-1	ATCC	Cat#CRL-11609
HEK293T	ATCC	Cat#CRL-3216
HeLa	ATCC	Cat#CCL-2
**Experimental models: organisms/strains**		
CD1 nude mice (HsdHli:CD1-Foxn1nu)	Envigo Israel	N/A
NSG mice	The Jackson Laboratory	RRID: IMSR_JAX:005557
**Oligonucleotides**		
Primers for cloning in Appendix Table S1	This paper	N/A
siRNA-GR	Dharmacon	ON-Target SMART oligonucleotides L-003424-00-0005
siRNA-ERG	Dharmacon	ON-Target SMART oligonucleotides L-003886-00-0005
NR3C1 sgRNA 1: TATGTATTTGTTTAAATAC	This paper	N/A
NR3C1 sgRNA 2: TAACACTGTTCTTCCCCTTCT	This paper	N/A
**Recombinant DNA**		
Gluc1-ETS fusion plasmids	This paper	N/A
Domains of GR plasmids	Srivastava et al	N/A
Domains of ERG plasmids	This paper	N/A
pLX304-ERG	Addgene	Plasmid #118625
HA-ERG	Gift from Prof. Haojie Huang, Mayo Clinic College of Medicine and Science, Rochester, MN.	N/A
Flag-WT-UB	This paper	N/A
Flag-K48R-UB	This paper	N/A
pEGFP GR	Addgene	Plasmid #47504
pGL3-GRE	Gift from Prof. Anne Gompel, Paris Descartes University	N/A
pGL3-EBS-WT-6R	Gift from Prof. Marie-Hélène David-Cordonnier, INSERM, France	N/A
GR expression vector	Gift from Prof. Andrew Cato, Karlsruhe Institute of Technology, Germany	N/A
HA-ΔNID &CID ERG	Vector Builder	N/A
pCEFL-HA-ERG-delta ETS	Gift from Prof Shai Izraeli, Schneider Children Medical Center of Israel	N/A
**Softwares**		
GraphPad Prism v8.0.2	GraphPad Software	RRID:SCR_002798
ImageJ v1.53t	National Institute of Health (NIH)	RRID:SCR_003070
Image Lab v6.0.1	Bio-Rad	RRID:SCR_014210

### Methods and protocols

#### Clinical samples and patient data analysis

The following clinical samples and datasets were analyzed in this study: (i) Patient samples and data from clinical trial NCT02430480 (Karzai et al, 2021; Wilkinson et al, 2021). This study recruited 37 men with PCa, who received ADT and enzalutamide. Biopsy samples and IHC staining of baseline and post-treatment biopsies were made available. (ii) Results reported by clinical data NCT01576172 (Hussain et al, 2018). All patients in this trial underwent metastatic site biopsy and were assigned to either abiraterone plus prednisone or to a combination of a PARP-1 inhibitor (veliparib) and abiraterone plus prednisone. Note that we only analyzed patients who were untreated with veliparib (58 patients). (iii) A dataset collected by the West Coast Dream Team collaborative group (Lundberg et al, 2023). Notably, all previously published experiments involving human subjects conformed to the principles set out in the WMA Declaration of Helsinki and the Department of Health and Human Services Belmont Report.

### Cell lines and reagents

PC3 cells were received from Prof. Haim Werner (Tel Aviv University). Other cells were from the American Type Tissue Culture Collection (ATCC). Human embryonic kidney cells, HEK293T, were cultured in Dulbecco’s modified Eagle medium (DMEM) supplemented with fetal bovine serum (FBS; 10%). DU145 and PC3 cells were grown in Roswell Park Memorial Institute (RPMI) medium supplemented with 10% FBS. VCaP cells were grown in RPMI supplemented with 10% FBS, glutamine, and sodium pyruvate. siRNA transfections used ON-Target SMART oligonucleotides from Dharmacon.

### Generation of stable cell derivatives

To knockout GR in VCaP cells, we used the CRISPR–Cas9 system and created a double-stranded break next to the Protospacer Adjacent Motif (PAM). The target site was selected using the ENSEMBL database. The selected targets (21 bp) included the PAM sequences in exon 5. For the establishment of DU145-tERG overexpressing cells, lentiviral particles were produced in HEK293T cells by co-transfecting lentiviral expression vectors containing the coding region of ERG (pLX304-ERG), together with second-generation viral packaging plasmids (VSVG; Addgene #14888) and psPAX2 (Addgene #12260). Twenty-four hours after transduction, the virus-containing medium was replaced with selection media containing blasticidin.

### Protein complementation assay (PCA)

The PCA method, which employs two inactive fragments of luciferase, was used essentially as previously described (Michnick et al, 2007). Prior to the assay, HEK293T cells were transfected in 48-well tissue culture plates with the Gluc1 and Gluc2 plasmids (25 ng, each), using the JetPEI reagent. Twenty-four hours later, the cells were extracted in luciferase lysis buffer (25 mM Tris, pH 8.5, 150 mM NaBr, 5 mM EDTA, 0.1% NP40, 5% glycerol, 65 μM sodium oxalate, 0.5 mM reduced glutathione, and 0.5 mM oxidized glutathione). Native coelenterazine (Nanolight) was diluted in luciferase assay buffer (25 mM Tris, pH 7.75, 1 mM EDTA, 0.5 mM reduced glutathione, 0.5 mM oxidized glutathione, and 75 mM urea) to a final concentration of 20 μM. Luminescence signals were determined using a Veritas microplate luminometer (Turner BioSystems).

### Cell lysis, immunoblotting, and co-immunoprecipitation assays

Post treatment, cells were washed twice with ice-cold saline. Cell lysates were collected in a mild lysis buffer (50 mM HEPES, pH 7.5, 10% glycerol, 150 mM NaCl, 1% Triton X-100, 1 mM EDTA, 1 mM EGTA, 10 mM NaF, and 30 mM β-glycerol phosphate). Proteins were immunoprecipitated from cell lysates using beads conjugated to an antibody. Following 2 h of incubation at 4 °C, complexes were washed three times, and bound proteins were eluted in concentrated gel sample buffer. Eluates were subjected to electrophoresis and immunoblotting. For immunoblotting, cleared cell lysates were resolved using electrophoresis, which was followed by electrophoretic transfer to a nitrocellulose membrane. Membranes were blocked in TBS-T (tris-buffered saline containing Tween-20) containing 1% low-fat milk, blotted overnight with a primary antibody, washed three times in TBS-T, incubated for 30 min with a secondary antibody linked to horseradish peroxidase, and washed once again with TBS-T. Immunoreactive bands were detected using the ECL reagent (Bio-Rad, USA).

### Cell growth and immunoblotting assays

For XTT assays, cells (2 × 10^4^) were seeded in a 96-well plate, in triplicates, and treated with drugs for the indicated time intervals. Subsequently, XTT was added and following 3 h at 37 °C we determined optical density at 490 and 640 nm. For the thymidine incorporation assay, cells were plated onto 24-well plates at a density of 5 × 10^4^ cells/well, followed by the indicated treatments. Sixteen hours later, serum-containing media were replaced with fresh serum-free medium containing ^3^[H]-thymidine (1 μCi). After 48 h, the reaction was terminated by the addition of ice-cold trichloroacetic acid (5%; TCA). Five minutes later, the cells were solubilized in 1 N NaOH (for 10 min) followed by 1 N HCl. Samples were collected into scintillation vials containing scintillation fluid. Radioactivity was determined in a scintillation counter. The results shown are representative of experiments performed in quadruplicate. Primary antibodies were used at the following dilutions: anti-ERG (Cell Signaling Technology, #97249; 1:1000), anti-ERG (Santa Cruz, sc-271048; 1:500), anti-GR (Santa Cruz, sc-393232; 1:500), anti-GR (Santa Cruz, sc-8992; 1:1000), anti-ETV4 (Thermo Fisher, PA5-13595; 1:1000), anti-GAPDH (Millipore, MAB374; 1:5000), anti-Gaussia luciferase (Nanolight, 401 P; 1:2000), anti-HA (Cell Signaling Technology, #3724; 1:1000), anti-HA (Proteintech, 51064-2-AP; 1:2000), anti-vinculin (Sigma-Aldrich, V9264; 1:5000), anti-Flag (Proteintech, 20543-1-AP; 1:2000), anti-phospho-Chk1 Ser345 (Cell Signaling Technology, #2341; 1:1000), anti-Chk1 (Cell Signaling Technology, #2360; 1:1000), anti-phospho-Wee1 Ser642 (Cell Signaling Technology, #4910; 1:1000), anti-γH2AX S139 (Cell Signaling Technology, #2577; 1:1000), anti-Histone 3 (Abcam, ab1791; 1:5000), anti-LDH (Santa Cruz, sc-27230; 1:500), and normal rabbit IgG (Cell Signaling Technology, #2729; 1:1000). HRP-conjugated secondary antibodies were used at 1:10,000 dilution.

### Colony formation and apoptosis assays

Cells (150–300) were seeded in six-well plates. Ten days later, cells were fixed in paraformaldehyde (4%) and stained with crystal violet. Cells were then photographed and analyzed using ImageJ. Apoptosis assays were performed using the FITC Annexin V Apoptosis Detection Kit with 7-AAD (from BioLegend) and analyzed using flow cytometry. The assays were performed on a BD FACSAria Fusion instrument controlled by BD FACS Diva software v8.0.1 (BD Biosciences). Further analysis was performed using the FlowJo software v10.2 (Tree Star).

### RNA isolation and real-time PCR analysis

Total RNA was extracted using the PerfectPure RNA Cultured Cell Kit (5-prime, Hamburg) according to the manufacturer’s instructions. RNA quantity and quality were determined using the NanoDrop ND-1000 spectrophotometer (Thermo Fischer Scientific, Waltham, MA). Complementary DNA was synthesized using the High-Capacity Reverse Transcription kit (Applied Biosystems, Life Technologies, Carlsbad, CA, USA). Real-time qPCR analysis was performed with SYBR Green (Applied Biosystems) and specific primers on the StepOne Plus Real-Time PCR system (Applied Biosystems). qPCR signals (cT) were normalized to beta2-microglobulin (B2M).

### Luciferase reporter assays

Cells were co-transfected with a luciferase plasmid containing the consensus glucocorticoid response element (GRE) or the ERG’s DNA-binding site (EBS). Additionally, the pGL3-Control vector encoding Renilla luciferase (Promega, Madison, WI) was transfected as a control for transfection efficiency. Luciferase activity was determined using the Dual-Luciferase reporter assay system, according to the manufacturer’s instructions (Promega). Firefly luciferase luminescence values were normalized to Renilla luminescence and quantified relative to the control.

### Immunofluorescence analyses

Cells were washed with saline and treated with an acidic buffer (100 mM glycine-HCl, pH 3.0) for 3 min. Later, cells were washed and fixed for 15 min using 4% PFA. Next, cells were washed and permeabilized for 10 min with 0.2% Triton X-100 in saline, and then subjected to blocking using 3% albumin. Thereafter, the cells were incubated overnight with primary antibodies specific to ERG or GR. After incubation, cells were washed and incubated with a secondary antibody (from Thermo Fisher Scientific) and DAPI, followed by washing and mounting on slides for imaging. For immunofluorescence, primary antibodies were used at the following dilutions: anti-GR (Santa Cruz, sc-393232; 1:200) and anti-ERG (Cell Signaling Technology, #97249; 1:200). The secondary antibodies we used were: anti-mouse Alexa Fluor 555 (Thermo Fisher, A31572; 1:500) and anti-rabbit Alexa Fluor 488 (Thermo Fisher, A21202; 1:500).

### Ubiquitination assays

Cells were co-transfected with HA-tagged ERG and Flag-tagged wild-type and K48R ubiquitin plasmids. Post-transfection, cells were treated for 48 h with RU486. MG132 was added 8 h prior to the end of the incubation. Ubiquitinated ERG from cell lysates was immunoprecipitated using an anti-HA antibody. Immunoblotting used an anti-FLAG antibody. A fraction (5%) of the total extract was used as the input control.

### Subcellular fractionation

Cell pellets were lysed in 0.1 ml cytoplasmic lysis buffer (10 mM HEPES pH 7.9, 10 mM KCl, 0.1 mM EGTA, 0.1 mM EDTA, 1 mM DTT, and 0.5% NP-40). The cytoplasmic fraction was collected using centrifugation (600 g for 5 min). Nuclei were washed and resuspended in 50 µl nuclear lysis buffer (20 mM HEPES pH 7.9, 0.4 M NaCl, 1 mM EGTA, 1 mM EDTA, and 1 mM DTT) using repeated freezing and thawing. Supernatants containing the nuclear fraction were collected by centrifugation at 12,000 rpm for 20 min.

### Nucleotide sequencing of RNA

RNA was isolated using Dynabeads mRNA Direct Kit (Thermo Fisher Scientific). NGS libraries were prepared using a modified version of Transeq. In brief, RNA was barcoded and reverse-transcribed using poly-T primers, followed by the addition of an exonuclease to remove excess of the PCR primers. The single-stranded cDNA was converted to double-stranded DNA. The template DNA was then removed using DNase, and the generated RNA was fragmented and ligated to barcoded Illumina adapters. Reverse transcription of the ligation product was performed using primers specific to the Illumina adapters, and libraries of the resulting cDNA were generated and enriched by performing 12–15 PCR cycles. RNA-seq libraries (pooled at equimolar concentrations) were sequenced on an Illumina NextSeq 500 at a median sequencing depth of ∼10 million reads per sample. Sequences were mapped to the human genome (hg38), demultiplexed, and filtered.

### Analysis of RNA sequencing data

Single-end 100 bp reads were sequenced on Illumina NovaSeq. We obtained ~12 million reads per sample. Poly-A/T stretches and Illumina adapters were trimmed from the reads using cutadapt, and reads shorter than 30 bp were discarded. Reads were mapped to the human reference genome GRCh38_p13 using STAR (Dobin et al, 2013). Reads with the same UMI were removed using the PICARD MarkDuplicate tool. Gene expression levels were quantified using htseq-count. Differentially expressed genes were identified using DESeq2 (Love et al, 2014) with the betaPrior, cooksCutoff, and independentFiltering parameters set to False. Raw *P* values were adjusted for multiple testing using the procedure of Benjamini and Hochberg (Benjamini and Hochberg, 1995).

### Pathway analysis

The tool EnrichR (Chen et al, 2013) was used to perform pathway enrichment analysis. Selected genes were used for overrepresentation analysis; in the gene expression analysis, genes with a fold change of at least 1, or −1, and an adjusted *P* value smaller than 0.05 were analyzed.

### Chromatin immunoprecipitation (ChIP)

Cell fixation, chromatin immunoprecipitation, and nucleotide sequencing were performed as described (Blecher-Gonen et al, 2013). ChIP assays were carried out using ~10 million VCaP cells per sample. Cells were crosslinked for 10 min in 1% formaldehyde at 37 °C. This reaction was subsequently quenched in 2.5 M glycine for 5 min. Chromatin from formaldehyde-fixed cells was fragmented to a size range of 200–700 bases using a sonicator. Solubilized chromatin was immunoprecipitated overnight at 4 °C with GR- or ERG-specific antibodies. Antibody-chromatin complexes were pulled-down using protein G-Dynabeads (Life Technologies), washed, and then eluted. After crosslinking reversal, RNase A and proteinase K treatment, the immunoprecipitated DNA was extracted using AMP Pure beads (Beckman Coulter). During ChIP-seq library preparation, DNA isolated from ChIP experiments, as well as input control DNA, was end-repaired, A-tailed, ligated to barcoded Illumina adapters, PCR-amplified, and pooled for sequencing. For ChIP, antibodies were used at the following dilutions: anti-ERG (Cell Signaling Technology, #97249; 10 µg per ChIP), anti-GR (Santa Cruz, sc-8992; 10 µg per ChIP).

### Prostate cancer animal models

All animal experiments were approved by the Weizmann Institute’s Animal Care and Use Committee and the Institute’s Review Board (IRB). Male CB17/*SCID* mice (5-6 weeks old) were subcutaneously implanted in the right dorsal flank with 2.5 million VCaP, PC3, or DU145 cells suspended in saline (0.1 ml). Three prostate cancer PDX models, LuCaP 23.1 (ERG positive), LuCaP 35 (ERG positive), and LuCaP 96 (ERG-negative), were previously established (Nguyen et al, 2017). These models were expanded in NSG mice. Following euthanasia, tumors were removed from donor mice and cut into small fragments. A small pouch was made in the lower back of 5–6 week-old mice, and one tumor fragment was later inserted into the pouch. Mice were labeled with RF identification chips (from Trovan, Melton, UK). Tumor volume (*V*/mm^3^) was estimated using vernier caliper measurements of the longest axis, *α*/mm, and the perpendicular axis, *b*/mm. Tumor volume was calculated in accordance with the equation *V*  =  (4*π*/3) x (*α*/2)^2^ x (*b*/2). When the volume of xenografts reached approximately 150 mm^3^, mice were randomized into groups and treatments were initiated. Animals were intraperitoneally treated once per day with RU486 (1 mg/kg; in 0.1% Tween 80) or with Corcept compounds (50 mg/kg; in 0.1% Tween 80, 0.5% hydroxypropyl methylcellulose, HPMC). Alternatively, mice were orally treated with enzalutamide (10 mg/kg; 0.1% Tween 80, 0.5% HPMC) or metyrapone (25 mg/kg, in water). Animals were euthanized when tumors reached 1500 mm^3^.

### Determination of cortisol levels

Approximately 50 mg of tissue was collected from each tumor sample. The tissue pieces were briefly washed in phosphate-buffered saline (PBS) and then thoroughly minced and homogenized in PBS. The homogenate was centrifuged at 5000×*g* for 5 min at 4 °C to precipitate the cells and debris. The resulting pellet was gently resuspended in RIPA lysis buffer, and a protease inhibitor cocktail was added. To ensure complete disruption of all cells, two rapid freeze–thaw cycles were performed. Lysates were then centrifuged at 12,000×*g* for 15 min at 4 °C. The clear supernatant was collected for downstream analysis. Total protein concentration in each sample was determined using the BCA assay. After normalization, an equal amount of protein from each group was used for the ELISA. Corticosterone levels were measured using the DetectX® Corticosterone Enzyme Immunoassay Kit (catalog number K014-H1), following the manufacturer’s instructions. In brief, samples and standards of defined concentrations were added to the assay plate, along with the DetectX® Corticosterone Antibody and DetectX® Corticosterone Conjugate. The plate was incubated for 1.5 h. After incubation, each well was washed five times with wash buffer. Next, 0.1 ml of TMB substrate solution was added to each well and incubated at room temperature for 15 min. The reaction was stopped by adding 0.050 ml of the Stop solution. Absorbance was determined at 450 nm. Absolute corticosterone concentrations (pg/ml) were determined from the standard curve and presented for each experimental group.

### Immunohistochemical analyses

Formalin-fixed, paraffin-embedded blocks of matched untreated biopsy tissue or posttreatment whole-mount prostatectomies were sectioned at 5 µm thickness onto SuperFrost Plus charged glass slides. Slides were stained with antibodies against ERG (clone EPR3864) and GR (clone D6H2L) and analyzed as previously described (Wilkinson et al, 2021). Briefly, after deparaffinization and rehydration, antigen retrieval was performed in a pressure cooker at 110 °C for 15 min in Diva Decloaker buffer (Biocare) and stained for ERG (1:200 dilution) or GR (1:400 dilution) for 1 h using a Biocare IP FLX autostainer. Secondary detection was performed with Mach 4 polymer/probe reagents, developed with DAB, and counterstained with hematoxylin. After mounting, slides were scanned using an AxioScan.Z1 slide scanner. Fully quantitative enumeration of tumor cells expressing ERG and GR was performed using Definiens XD 64 software with 12 staining subsets per slide and segmentation level 9. A percent positive weighted histology index (between 0 and 1) was used to report expression of ERG and GR nuclear intensities. Semi-quantitative H-scoring (between 0 and 300) was used to report the nuclear intensity of ERG and GR in prostatectomy tissues. All tissue analyses were performed by a genitourinary pathologist.

### Statistical analyses

All data were analyzed using R and GraphPad Prism v8.0.2. Statistical comparisons between two groups were performed using an unpaired two-tailed Student’s *t* test or Mann–Whitney *U* test. Comparisons among three or more groups were performed using one-way ANOVA followed by Tukey’s or Dunnett’s post hoc test for multiple comparisons, as indicated in the figure legends. For experiments with two independent variables, two-way ANOVA with Tukey’s post hoc test was used. Survival analyses were performed using the Kaplan–Meier method, and differences between groups were assessed using the log-rank (Mantel–Cox) test. For clinical cohort analyses, one-sided Wilcoxon rank-sum test (ERG) or one-sided unpaired *t* test (GR) was used, and progression-free survival was compared using the Gehan–Breslow–Wilcoxon test. For ChIP-seq motif enrichment, the hypergeometric test was used (HOMER). For RNA-seq differential expression analysis, DESeq2 with Wald test and Benjamini–Hochberg correction were used. Pathway enrichment analysis was performed using Fisher’s exact test (EnrichR) or permutation-based GSEA. Statistical significance thresholds are denoted as *P < 0.05; **P < 0.01; ***P < 0.001 and ****P < 0.0001. All exact *P* values are reported in the corresponding figure legends and in Appendix Table S3. No statistical method was used to predetermine sample size. For xenograft experiments, mice were randomly assigned to experimental groups once tumors reached the target initial volume (~150 mm³). Wherever feasible, the investigators were blinded to group allocation during data collection and analysis. All experiments were independently replicated at least twice (typically three times). For experiments with *n* = 2, we showed representative results are shown.

## Supplementary information

Appendix

Peer Review File

Source data Fig. 2

Source data Fig. 3

Source data Fig. 4

Source data Fig. 5

Source data Fig. 6

Source data Fig. 7

Expanded View Figures

## Data Availability

Reagents and data generated during this study will be made available after contacting the corresponding author. Raw RNA-seq data have been deposited in the Gene Expression Omnibus (GEO) under accession number GSE247422. In addition, raw ChIP-seq data have been deposited under accession number GSE247347. The source data of this paper are collected in the following database record: biostudies:S-SCDT-10_1038-S44321-026-00423-7.
