# Supplementary material for: TMPRSS2-ERG confers resistance of prostate cancer to antiandrogens
Source: EMBO Mol Med. 2026 May 12;18(6):2062–97. doi: 10.1038/s44321-026-00423-7 (PMC13269939; doi:10.1038/s44321-026-00423-7)
Supplement: Supplementary file 1 — Appendix [file 44321_2026_423_MOESM1_ESM.pdf]

Appendix

Table of contents	Page no.
Appendix Table S1	1
Appendix Table S2	2
Appendix Table S3	4

Appendix Table S1: List of all primers used for cloning and validation (related to Figure 2).

Cloning primers	Sequence
ERG-E1 forward	TGGTGGGTCCTCCGGAATTCAGACTGTCCCGGACCCA
ERG-E1 Reverse	AAACGGGCCCTCTAGATTAGGAGCTGTCCGACAGGAGCTC
ERG-E2 forward	TGGTGGGTCCTCCGGAAGCTACATGGAGGAGAAGCACATG
ERG-E2 Reverse	AAACGGGCCCTCTAGATTAGTAGTAAGTGCCCAGATGAGA
ERG-E3 forward	TGGTGGGTCCTCCGGACTCCACTACCTCAGAGAGACT
ERG-E3 Reverse	Same as E2 reverse
ΔETS forward	Same as E3 forward (used ΔETS plasmid as template)
ΔETS Reverse	Same as E3 reverse (used ΔETS plasmid as template)

Appendix Table S2: List of down- or up-regulated genes in DEX-treated VCaP cells. (related to Figure 4I). CSS-grown VCaP cells were treated in triplicates with DEX (1 μM), or with vehicle. Twenty-four hours later, cells were harvested and RNA was isolated and sequenced.

Upregulated genes		Downregulated genes
ADRA2A	PER1	RASAL2
TFEB	FAM43A	KCNK15
SDK1	MAOA	LMO4
HSD17B6	MYBPC1	INTS4P1
FKBP5	TGM2	GCNT1
HOMER2	MAFB	LINC00173
GHRHR	CEBPD	7SK
TSPAN14	PHGR1	NRXN1
GALNT7	NKILA	FOLH1
ZBTB16	TMPRSS5	TNFSF15
LOX	LRG1	SGMS1-AS1
BCL6	CCNJ	ABCB5
TFCP2L1	RHOU	PLD1
IL1R1	CLIP1	SMARCA1
SDC1	CLGN	ITGB8
MYCL	APOD	SEPTIN4
SGK1	MYH13	FOXN1
ELL2	TUBA3D	STX2
NPY	SORBS1	ZC4H2
PMEPA1	SLA2	ARHGAP24
MAP1B	TRIM35	FRAS1
KLF4	DUSP4	GALNT6
KLK3	FMOD	CCDC15
TMEFF2	AKAP12	SLAMF9
AGTR1	HSPA12B	CNIH2
ARMC12	DNAJC14	COL14A1
MYO1E	SLC30A2	SNTG2-AS1
ERG	IL6R	RETREG1-AS1
SLC45A3	CA4	HOXC13-AS
PDE9A	CYP4F22	PRKACB-DT
KLK2	RFX6	PTPRN
IRS1	KRT14	PLXNA2
TCAF2	THSD4	HGFAC
SPSB1	ZNF470	ATF3

KLK15	ZBTB8B	KLHDC8A
		FBXL7
		GOT1-DT
		LINC00926

Appendix Table S3

Figure	Panel	Comparison	Statistical Test	Exact p-value	n	Error bars
1	C (ERG)	Responders vs Non-responders	One-sided Wilcoxon	0.0073	37 patients	N/A (box plot: median, 25th–75th percentiles, min–max whiskers)
1	C (GR)	Responders vs Non-responders	One-sided unpaired t-test	0.039	37 patients	N/A (box plot: median, 25th–75th percentiles, min–max whiskers)
1	D	tERG− vs tERG+ PFS	Gehan-Breslow-Wilcoxon (two-sided)	0.0285	58 patients	N/A (Kaplan-Meier)
2	A (ERG)	Vehicle vs DEX	Two-way ANOVA with Tukey's	6.89×10^{-4}	3	Mean ± SEM
2	A (ERG)	DEX vs DEX+RU486	Two-way ANOVA with Tukey's	1.46×10^{-3}	3	Mean ± SEM
2	A (ETV1)	Vehicle vs DEX	Two-way ANOVA with Tukey's	1.89×10^{-3}	3	Mean ± SEM
2	A (ETV1)	DEX vs DEX+RU486	Two-way ANOVA with Tukey's	5.53×10^{-3}	3	Mean ± SEM
2	A (ETV4)	Vehicle vs DEX	Two-way ANOVA with Tukey's	3.11×10^{-3}	3	Mean ± SEM
2	A (ETV4)	DEX vs DEX+RU486	Two-way ANOVA with Tukey's	8.34×10^{-3}	3	Mean ± SEM
2	A (ETV5)	Vehicle vs DEX	Two-way ANOVA with Tukey's	1.85×10^{-3}	3	Mean ± SEM
2	A (ETV5)	DEX vs DEX+RU486	Two-way ANOVA with Tukey's	4.11×10^{-4}	3	Mean ± SEM

2	A (ETV6)	Vehicle vs DEX	Two-way ANOVA with Tukey's	0.256 (ns)	3	Mean ± SEM
2	C	Control vs DEX (PLA)	Mann-Whitney U (two-sided)	1.61×10^{-4}	>40 cells per condition	SEM
2	C	DEX vs DEX+RU486 (PLA)	Mann-Whitney U (two-sided)	5.26×10^{-4}	>40 cells per condition	SEM
2	C	Control vs DEX+RU486 (PLA)	Mann-Whitney U (two-sided)	0.093 (ns)	>40 cells per condition	SEM
2	G	DEX vs DEX+CORT125134	One-way ANOVA with Tukey's	6.62×10^{-3}	2	Mean ± SEM
2	G	DEX vs DEX+CORT125281	One-way ANOVA with Tukey's	4.97×10^{-3}	2	Mean ± SEM
2	G	DEX vs DEX+CORT125329	One-way ANOVA with Tukey's	4.08×10^{-3}	2	Mean ± SEM
3	C	0h vs 48h	One-way ANOVA with Dunnett's	0.357 (ns)	2	Mean ± SEM
3	C	0h vs 72h	One-way ANOVA with Dunnett's	1.25×10^{-3}	2	Mean ± SEM
3	C	0h vs 96h	One-way ANOVA with Dunnett's	0.016	2	Mean ± SEM
4	A	EV vs ERG 0.05 µg	One-way ANOVA with Dunnett's	5.35×10^{-5}	3	Mean ± SEM
4	A	EV vs ERG 0.1 µg	One-way ANOVA with Dunnett's	0.026	3	Mean ± SEM
4	A	EV vs ERG 0.2 µg	One-way ANOVA with Dunnett's	3.04×10^{-6}	3	Mean ± SEM

4	B	ERG vs ERG+GR 0.05 µg	One-way ANOVA with Tukey's	7.59×10^{-3}	3	Mean ± SEM
4	B	ERG vs ERG+GR 0.1 µg	One-way ANOVA with Tukey's	3.94×10^{-5}	3	Mean ± SEM
4	C	Vehicle vs DEX	One-way ANOVA with Tukey's	2.96×10^{-6}	3	Mean ± SEM
4	C	Vehicle vs RU486	One-way ANOVA with Tukey's	0.126 (ns)	3	Mean ± SEM
4	C	DEX vs DEX+RU486	One-way ANOVA with Tukey's	3.08×10^{-5}	3	Mean ± SEM
4	E	WT vs ΔNID&CID (overall, Vehicle)	Two-way ANOVA (Construct × Dose)	Construct: $p < 0.0001$; Interaction: $p < 0.0001$	2	Mean ± SEM
4	E	WT vs ΔNID&CID at 0.1 µg (Veh)	Two-way ANOVA with Tukey's	8.37×10^{-3}	2	Mean ± SEM
4	E	WT vs ΔNID&CID at 0.25 µg (Veh)	Two-way ANOVA with Tukey's	0.015	2	Mean ± SEM
4	E	WT vs ΔNID&CID at 0.5 µg (Veh)	Two-way ANOVA with Tukey's	8.55×10^{-3}	2	Mean ± SEM
4	E	WT vs ΔNID&CID at 0.75 µg (Veh)	Two-way ANOVA with Tukey's	1.79×10^{-4}	2	Mean ± SEM
4	E	WT vs ΔNID&CID at 1.0 µg (Veh)	Two-way ANOVA with Tukey's	0.020	2	Mean ± SEM
4	F	ChIP-seq peak profiles	N/A (genome-wide)	N/A	3	N/A
4	G	ERG–GR peak overlap	Hypergeometric test (HOMER)	See motif enrichment p-values in panel	N/A (ChIP-seq)	N/A
4	H	IGV tracks (MYC, NKX3-1)	N/A (qualitative)	N/A	N/A	N/A
4	I	RNA-seq volcano plot	DESeq2 (Wald test, BH-adjusted)	See Appendix Table S2	3	N/A

				for full DEG list		
4	J	GSEA pathways (DEX upregulated)	GSEA permutation-based FDR	See GSEA output; FDR $q < 0.05$	N/A	N/A
5	A (VCaP)	Vehicle vs 1.0 µM RU486	Two-way ANOVA with Tukey's	1.09×10^{-4}	3	Mean ± SEM
5	A (VCaP)	Vehicle vs 5.0 µM	Two-way ANOVA with Tukey's	2.20×10^{-4}	3	Mean ± SEM
5	A (VCaP)	Vehicle vs 10.0 µM	Two-way ANOVA with Tukey's	1.55×10^{-6}	3	Mean ± SEM
5	A (VCaP)	Vehicle vs 20.0 µM	Two-way ANOVA with Tukey's	5.38×10^{-7}	3	Mean ± SEM
5	A (PC3)	Vehicle vs 5.0 µM	Two-way ANOVA with Tukey's	2.49×10^{-4}	2	Mean ± SEM
5	A (PC3)	Vehicle vs 10.0 µM	Two-way ANOVA with Tukey's	3.74×10^{-5}	2	Mean ± SEM
5	A (PC3)	Vehicle vs 20.0 µM	Two-way ANOVA with Tukey's	9.55×10^{-4}	2	Mean ± SEM
5	A (CL1)	Vehicle vs 5.0 µM	Two-way ANOVA with Tukey's	9.67×10^{-3}	3	Mean ± SEM
5	A (CL1)	Vehicle vs 10.0 µM	Two-way ANOVA with Tukey's	2.75×10^{-5}	3	Mean ± SEM
5	A (CL1)	Vehicle vs 20.0 µM	Two-way ANOVA with Tukey's	1.63×10^{-5}	3	Mean ± SEM
5	A (DU145)	Vehicle vs 20.0 µM	Two-way ANOVA with Tukey's	1.43×10^{-3}	3	Mean ± SEM
5	A (DU145)	Vehicle vs other doses	Two-way ANOVA with Tukey's	ns at 0.5–15 µM	3	Mean ± SEM
5	A (RWPE-1)	Vehicle vs each dose	Two-way ANOVA with Tukey's	ns at all doses tested	3	Mean ± SEM

5	B (VCaP)	Vehicle vs CORT134 (≥1 μM)	Two-way ANOVA with Tukey's	$p < 0.0001$ at ≥1 μM	2–3	Mean ± SEM
5	B (VCaP)	Vehicle vs CORT281 (≥0.5 μM)	Two-way ANOVA with Tukey's	$p = 0.019$ at 0.5 μM; $p < 0.01$ at ≥5 μM	2–3	Mean ± SEM
5	B (VCaP)	Vehicle vs CORT329 (≥8 μM)	Two-way ANOVA with Tukey's	$p < 0.001$ at ≥8 μM	2–3	Mean ± SEM
5	B (DU145)	Vehicle vs each SGRM dose	Two-way ANOVA with Tukey's	ns at all doses tested	2–3	Mean ± SEM
5	C (VCaP)	CON vs RU486 10 μM	One-way ANOVA with Tukey's	1.70×10^{-5}	4	Mean ± SEM
5	C (VCaP)	CON vs RU486 5 μM	One-way ANOVA with Tukey's	6.29×10^{-5}	4	Mean ± SEM
5	C (PC3)	CON vs RU486 10 μM	One-way ANOVA with Tukey's	1.29×10^{-4}	4	Mean ± SEM
5	C (PC3)	CON vs RU486 5 μM	One-way ANOVA with Tukey's	1.68×10^{-3}	4	Mean ± SEM
5	C (DU145)	CON vs RU486 10 μM	One-way ANOVA with Tukey's	1.00 (ns)	4	Mean ± SEM
5	C (DU145)	CON vs RU486 5 μM	One-way ANOVA with Tukey's	0.362 (ns)	4	Mean ± SEM
5	E	RNA-seq volcano (VCaP ± RU486)	DESeq2 (Wald test, BH-adjusted)	See DEG list	3	N/A
5	F	Pathway enrichment (EnrichR)	Fisher's exact / hypergeometric	Adjusted $p < 0.05$ cutoff	N/A	N/A
6	A (VCaP)	Vehicle vs Metyrapone	Two-way RM ANOVA with Dunnett's	0.025 (Day 15); 2.10×10^{-5} (Day 18)	5	Mean ± SEM
6	A (VCaP)	Vehicle vs RU486	Two-way RM ANOVA with Dunnett's	0.015 (Day 15); 1.51×10^{-5} (Day 18)	5	Mean ± SEM

6	B (DU145)	Vehicle vs RU486	Two-way RM ANOVA with Dunnett's	0.765 (ns, Day 43)	6	Mean ± SEM
6	B (DU145)	Vehicle vs Metyrapone	Two-way RM ANOVA with Dunnett's	0.803 (ns, Day 43)	6	Mean ± SEM
6	C (DU145-tERG)	Vehicle vs RU486	One-way ANOVA with Dunnett's	0.626 (ns)	Veh=2, RU486=2	Mean ± SEM
6	C (DU145-tERG)	Vehicle vs Metyrapone	One-way ANOVA with Dunnett's	0.388 (ns)	Veh=2, Met=3	Mean ± SEM
6	D (DU145-EV)	Vehicle vs RU486	One-way ANOVA with Dunnett's	1.88×10^{-3}	Veh=5, RU486=6	Mean ± SEM
6	D (DU145-EV)	Vehicle vs Metyrapone	One-way ANOVA with Dunnett's	0.012	Veh=5, Met=5	Mean ± SEM
7	A (LuCaP 23.1)	Vehicle vs ENZ	One-way ANOVA with Dunnett's	< 0.0001 (Day 30)	VEH=6, ENZ=6	Mean ± SEM
7	A (LuCaP 23.1)	Vehicle vs RU486	One-way ANOVA with Dunnett's	< 0.0001 (Day 30)	VEH=6, RU486=4	Mean ± SEM
7	A (LuCaP 23.1)	Vehicle vs MET	One-way ANOVA with Dunnett's	< 0.0001 (Day 30)	VEH=6, MET=4	Mean ± SEM
7	A (LuCaP 23.1)	Vehicle vs ENZ+MET	One-way ANOVA with Dunnett's	< 0.0001 (Day 30)	VEH=6, ENZ+MET=6	Mean ± SEM
7	A (LuCaP 23.1)	Vehicle vs ENZ+RU486	One-way ANOVA with Dunnett's	< 0.0001 (Day 30)	VEH=6, ENZ+RU=6	Mean ± SEM

7	B (LuCaP 23.1)	VEH vs ENZ	Log-rank (Mantel-Cox)	2.63×10^{-3}	VEH =6, ENZ =5	N/A (Kaplan-Meier)
7	B (LuCaP 23.1)	VEH vs RU486	Log-rank (Mantel-Cox)	0.037	VEH =6, RU486=5	N/A (Kaplan-Meier)
7	B (LuCaP 23.1)	VEH vs MET	Log-rank (Mantel-Cox)	2.63×10^{-3}	VEH =6, MET =5	N/A (Kaplan-Meier)
7	B (LuCaP 23.1)	VEH vs ENZ+MET	Log-rank (Mantel-Cox)	1.24×10^{-3}	VEH =6, ENZ +MET=6	N/A (Kaplan-Meier)
7	B (LuCaP 23.1)	VEH vs ENZ+RU486	Log-rank (Mantel-Cox)	2.63×10^{-3}	VEH =6, ENZ +RU =6	N/A (Kaplan-Meier)
7	C (LuCaP 35)	Vehicle vs ENZ	One-way ANOVA with Dunnett's	7.66×10^{-3} (Day 25)	VEH =6, ENZ =4	Mean ± SEM
7	C (LuCaP 35)	Vehicle vs ENZ+RU486	One-way ANOVA with Dunnett's	4.16×10^{-3} (Day 25)	VEH =6, ENZ +RU =5	Mean ± SEM
7	C (LuCaP 35)	Vehicle vs ENZ+MET	One-way ANOVA with Dunnett's	1.45×10^{-4} (Day 25)	VEH =6, ENZ +MET=5	Mean ± SEM
7	D (LuCaP 35)	VEH vs ENZ	Log-rank (Mantel-Cox)	5.75×10^{-3}	VEH =6, ENZ =4	N/A (Kaplan-Meier)
7	D (LuCaP 35)	VEH vs ENZ+RU486	Log-rank (Mantel-Cox)	2.44×10^{-3}	VEH =6, ENZ +RU =5	N/A (Kaplan-Meier)

7	D (LuCaP 35)	VEH vs ENZ+MET	Log-rank (Mantel-Cox)	2.44×10^{-3}	VEH =6, ENZ+MET=5	N/A (Kaplan-Meier)
7	E (LuCaP 96)	Vehicle vs ENZ	One-way ANOVA with Dunnett's	7.17×10^{-4} (Day 28)	VEH =6, ENZ =7	Mean ± SEM
7	E (LuCaP 96)	Vehicle vs RU486	One-way ANOVA with Dunnett's	0.481 (ns, Day 28)	VEH =6, RU486=4–6	Mean ± SEM
7	E (LuCaP 96)	Vehicle vs MET	One-way ANOVA with Dunnett's	0.645 (ns, Day 28)	VEH =6, MET =5	Mean ± SEM
7	E (LuCaP 96)	Vehicle vs ENZ+MET	One-way ANOVA with Dunnett's	1.65×10^{-3} (Day 28)	VEH =6, ENZ+MET=6	Mean ± SEM
7	E (LuCaP 96)	Vehicle vs ENZ+RU486	One-way ANOVA with Dunnett's	0.014 (Day 28)	VEH =6, ENZ+RU =5	Mean ± SEM
7	F (LuCaP 96)	VEH vs ENZ	Log-rank (Mantel-Cox)	0.014	VEH =5, ENZ =3	N/A (Kaplan-Meier)
7	F (LuCaP 96)	VEH vs ENZ+RU486	Log-rank (Mantel-Cox)	0.030	VEH =5, ENZ+RU =2	N/A (Kaplan-Meier)
7	F (LuCaP 96)	VEH vs ENZ+MET	Log-rank (Mantel-Cox)	0.030	VEH =5, ENZ+MET=2	N/A (Kaplan-Meier)
7	F (LuCaP 96)	VEH vs RU486	Log-rank (Mantel-Cox)	0.371 (ns)	VEH =5, RU486=4	N/A (Kaplan-Meier)

7	F (LuCaP 96)	VEH vs MET	Log-rank (Mantel-Cox)	0.937 (ns)	VEH =5, MET =5	N/A (Kaplan-Meier)
EV1	B	Pre vs Post ERG mRNA (responders)	Paired t-test or Wilcoxon signed-rank	0.0047	Responder subset of 37 patients	Box plot: median, 25th–75th percentiles, min–max whiskers
EV1	C	GR IHC pre vs post (Wilcoxon)	Wilcoxon signed-rank test	0.018	Responder cohort	N/A
EV1	D	Spearman correlation ERG vs NR3C1	Spearman rank correlation	0.016	19 patients (GSE102124)	N/A
EV1	E	Spearman correlation ERG vs GR	Spearman rank correlation	0.00039	160 patients (WCDT)	N/A
EV1	F	Fisher's exact test (ERG/GR co-occurrence)	One-sided Fisher's exact test	0.0095	62 patients	N/A
EV2	G	GR-FL vs GRΔ1	Two-way ANOVA with Tukey's	0.013	3	Mean ± SEM
EV2	G	GR-FL vs GRΔ2	Two-way ANOVA with Tukey's	0.010	3	Mean ± SEM
EV2	G	GR-FL vs GRΔ3 (DBD-HR-LBD)	Two-way ANOVA with Tukey's	0.457 (ns)	3	Mean ± SEM
EV2	G	GR-FL vs GRΔ4	Two-way ANOVA with Tukey's	0.018	3	Mean ± SEM
EV2	G	GRΔ3 vs GRΔ1	Two-way ANOVA with Tukey's	5.56×10^{-3}	3	Mean ± SEM

EV2	G	GRΔ3 vs GRΔ2	Two-way ANOVA with Tukey's	4.31×10^{-3}	3	Mean ± SEM
EV2	J	ERG-FL vs E1 (NTD)	Two-way ANOVA with Tukey's	7.56×10^{-5}	3	Mean ± SEM
EV2	J	ERG-FL vs E2 (Pointed)	Two-way ANOVA with Tukey's	0.136 (ns)	3	Mean ± SEM
EV2	J	ERG-FL vs E3 (ETS)	Two-way ANOVA with Tukey's	0.080 (ns)	3	Mean ± SEM
EV2	J	ERG-FL vs E4 (CTD)	Two-way ANOVA with Tukey's	2.74×10^{-4}	3	Mean ± SEM
EV3	A	Vehicle vs ENZ (RT-qPCR)	One-way ANOVA with Dunnett's	0.0429	2	Mean ± SEM
EV3	A	ENZ vs DEX (RT-qPCR)	One-way ANOVA with Dunnett's	0.0267	2	Mean ± SEM
EV4	A	EV vs ERG	One-way ANOVA with Tukey's	8.71×10^{-4}	3	Mean ± SEM
EV4	A	EV vs ERG+GR	One-way ANOVA with Tukey's	4.83×10^{-4}	3	Mean ± SEM
EV4	A	EV vs GR	One-way ANOVA with Tukey's	5.48×10^{-3}	3	Mean ± SEM
EV4	A	ERG vs ERG+GR	One-way ANOVA with Tukey's	0.057 (ns)	3	Mean ± SEM
EV4	B	WT vs WT+GR	One-way ANOVA with Tukey's	4.01×10^{-4}	3	Mean ± SEM
EV4	B	WT vs GR-KO	One-way ANOVA with Tukey's	0.041	3	Mean ± SEM
EV4	B	GR-KO vs GR-KO+GR	One-way ANOVA with Tukey's	3.60×10^{-5}	3	Mean ± SEM
EV4	B	WT+GR vs GR-KO+GR	One-way ANOVA with Tukey's	0.021	3	Mean ± SEM

EV4	C	GRE vs GRE+GR	One-way ANOVA with Dunnett's	0.012	3	Mean ± SEM
EV4	C	GRE+GR vs GRE+GR+ERG(0.2)	One-way ANOVA with Dunnett's	0.167 (ns)	3	Mean ± SEM
EV4	C	GRE+GR vs GRE+GR+ERG(0.4)	One-way ANOVA with Dunnett's	0.136 (ns)	3	Mean ± SEM
EV4	C	GRE+GR vs GRE+GR+ERG(0.6)	One-way ANOVA with Dunnett's	0.125 (ns)	3	Mean ± SEM
EV4	D (with out GR)	EV vs 1 ng	One-way ANOVA with Dunnett's	1.30×10^{-3}	3	Mean ± SEM
EV4	D (with out GR)	EV vs 5 ng	One-way ANOVA with Dunnett's	6.63×10^{-5}	3	Mean ± SEM
EV4	D (with out GR)	EV vs 10 ng	One-way ANOVA with Dunnett's	1.37×10^{-4}	3	Mean ± SEM
EV4	D (with out GR)	EV vs 50 ng	One-way ANOVA with Dunnett's	1.43×10^{-3}	3	Mean ± SEM
EV4	D (with out GR)	EV vs 100 ng	One-way ANOVA with Dunnett's	3.19×10^{-4}	3	Mean ± SEM
EV4	D (with GR)	EV vs 1 ng	One-way ANOVA with Dunnett's	0.018	3	Mean ± SEM
EV4	D (with GR)	EV vs 5 ng	One-way ANOVA with Dunnett's	3.68×10^{-4}	3	Mean ± SEM
EV4	D (with GR)	EV vs 10 ng	One-way ANOVA with Dunnett's	2.97×10^{-6}	3	Mean ± SEM
EV4	D (with GR)	EV vs 50 ng	One-way ANOVA with Dunnett's	2.36×10^{-4}	3	Mean ± SEM

EV4	D (with GR)	EV vs 100 ng	One-way ANOVA with Dunnett's	3.40×10^{-6}	3	Mean ± SEM
EV5	B (VCaP)	siCON vs siGR	Unpaired t-test	0.028	4	Mean ± SEM
EV5	B (PC3)	siCON vs siGR	Unpaired t-test	1.21×10^{-6}	4	Mean ± SEM
EV5	B (DU145)	siCON vs siGR	Unpaired t-test	0.023	4	Mean ± SEM
EV5	E (3 days)	WT vs KO1	Unpaired t-test	5.37×10^{-3}	4	Mean ± SEM
EV5	E (3 days)	WT vs KO2	Unpaired t-test	0.013	4	Mean ± SEM
EV5	E (6 days)	WT vs KO1	Unpaired t-test	2.55×10^{-5}	3	Mean ± SEM
EV5	E (6 days)	WT vs KO2	Unpaired t-test	3.06×10^{-5}	3	Mean ± SEM
EV5	F	WT vs KO1	Unpaired t-test	7.68×10^{-7}	4	Mean ± SEM
EV5	F	WT vs KO2	Unpaired t-test	7.59×10^{-7}	4	Mean ± SEM
EV5	G	WT vs KO1	Unpaired t-test	1.61×10^{-4}	3	Mean ± SEM
EV4	E	De novo motif analysis (ChIP-seq)	HOMER motif enrichment	See motif p-values in panel	N/A (ChIP-seq)	N/A
EV4	F	ERG–GR peak overlap (DU145-tERG ChIP)	HOMER motif enrichment	See motif p-values in panel	N/A (ChIP-seq)	N/A
EV4	G	DU145-EV vs tERG ± DEX (RNA-seq volcano)	DESeq2 (Wald test, BH-adjusted)	0.05	3	N/A
EV4	H	RT-qPCR VCaP ± DEX (8h, 24h)	N/A (qualitative)	0.05	3	RelativeFC
EV5	G	WT vs KO2	Unpaired t-test	2.32×10^{-4}	3	Mean ± SEM
EV5	I	siCON vs siERG ± RU486 (XTT)	Two-way ANOVA with Sidak's	0.0001	2	Mean ± SEM
EV5	K (siCON)	CON vs ENZ 2 µM	Two-way ANOVA with Sidak's	1.52×10^{-4}	4	Mean ± SEM
EV5	K (siCON)	CON vs ENZ 5 µM	Two-way ANOVA with Sidak's	9.21×10^{-6}	4	Mean ± SEM
EV5	K (siERG)	CON vs ENZ 2 µM	Two-way ANOVA with Sidak's	1.87×10^{-3}	4	Mean ± SEM

EV5	K (siERG)	CON vs ENZ 5 µM	Two-way ANOVA with Sidak's	2.25×10^{-6}	4	Mean ± SEM
EV5	K	siCON vs siERG at CON	Two-way ANOVA with Sidak's	4.16×10^{-3}	4	Mean ± SEM
EV5	K	siCON vs siERG at ENZ 2 µM	Two-way ANOVA with Sidak's	4.77×10^{-3}	4	Mean ± SEM
EV5	K	siCON vs siERG at ENZ 5 µM	Two-way ANOVA with Sidak's	3.89×10^{-4}	4	Mean ± SEM
EV6	A (VCaP)	Vehicle vs Metyrapone	One-way ANOVA with Dunnett's	3.81×10^{-3}	3	Violin plot
EV6	A (VCaP)	Vehicle vs RU486	One-way ANOVA with Dunnett's	0.039	3	Violin plot
EV6	A (DU145)	Vehicle vs Metyrapone	One-way ANOVA with Dunnett's	9.18×10^{-4}	3	Violin plot
EV6	A (DU145)	Vehicle vs RU486	One-way ANOVA with Dunnett's	0.431 (ns)	3	Violin plot
EV6	B	Vehicle vs C134 (Day 20)	Two-way RM ANOVA with Dunnett's	0.051 (ns)	3	Mean ± SEM
EV6	B	Vehicle vs C281 (Day 20)	Two-way RM ANOVA with Dunnett's	0.048	3	Mean ± SEM
EV6	B	Vehicle vs C329 (Day 20)	Two-way RM ANOVA with Dunnett's	1.99×10^{-3}	3	Mean ± SEM
EV6	C	Vehicle vs RU486 (Day 22)	Unpaired t-test	0.049	Veh=8, RU486=9	Mean ± SEM
EV6	E (EV)	CON vs RU486 10 µM	One-way ANOVA with Dunnett's	0.400 (ns)	4	Mean ± SEM
EV6	E (EV)	CON vs RU486 5 µM	One-way ANOVA with Dunnett's	0.723 (ns)	4	Mean ± SEM
EV6	E (tERG#1)	CON vs RU486 10 µM	One-way ANOVA with Dunnett's	8.97×10^{-7}	4	Mean ± SEM

EV6	E (tER G#1)	CON vs RU486 5 μM	One-way ANOVA with Dunnett's	5.80×10^{-6}	4	Mean ± SEM
EV6	E (tER G#2)	CON vs RU486 10 μM	One-way ANOVA with Dunnett's	2.22×10^{-8}	4	Mean ± SEM
EV6	E (tER G#2)	CON vs RU486 5 μM	One-way ANOVA with Dunnett's	1.64×10^{-5}	4	Mean ± SEM
EV7	B	Individual tumor growth (LuCaP 23.1)	N/A (individual curves)	N/A	See Fig 7A	N/A
EV7	C	Individual tumor growth (LuCaP 35)	N/A (individual curves)	N/A	See Fig 7C	N/A
EV7	D	Individual tumor growth (LuCaP 96)	N/A (individual curves)	N/A	See Fig 7E	N/A
ns = not significant						
