# Supplementary figures and images for: TMPRSS2-ERG confers resistance of prostate cancer to antiandrogens
Source: EMBO Mol Med. 2026 May 12;18(6):2062–97. doi: 10.1038/s44321-026-00423-7 (PMC13269939; doi:10.1038/s44321-026-00423-7)

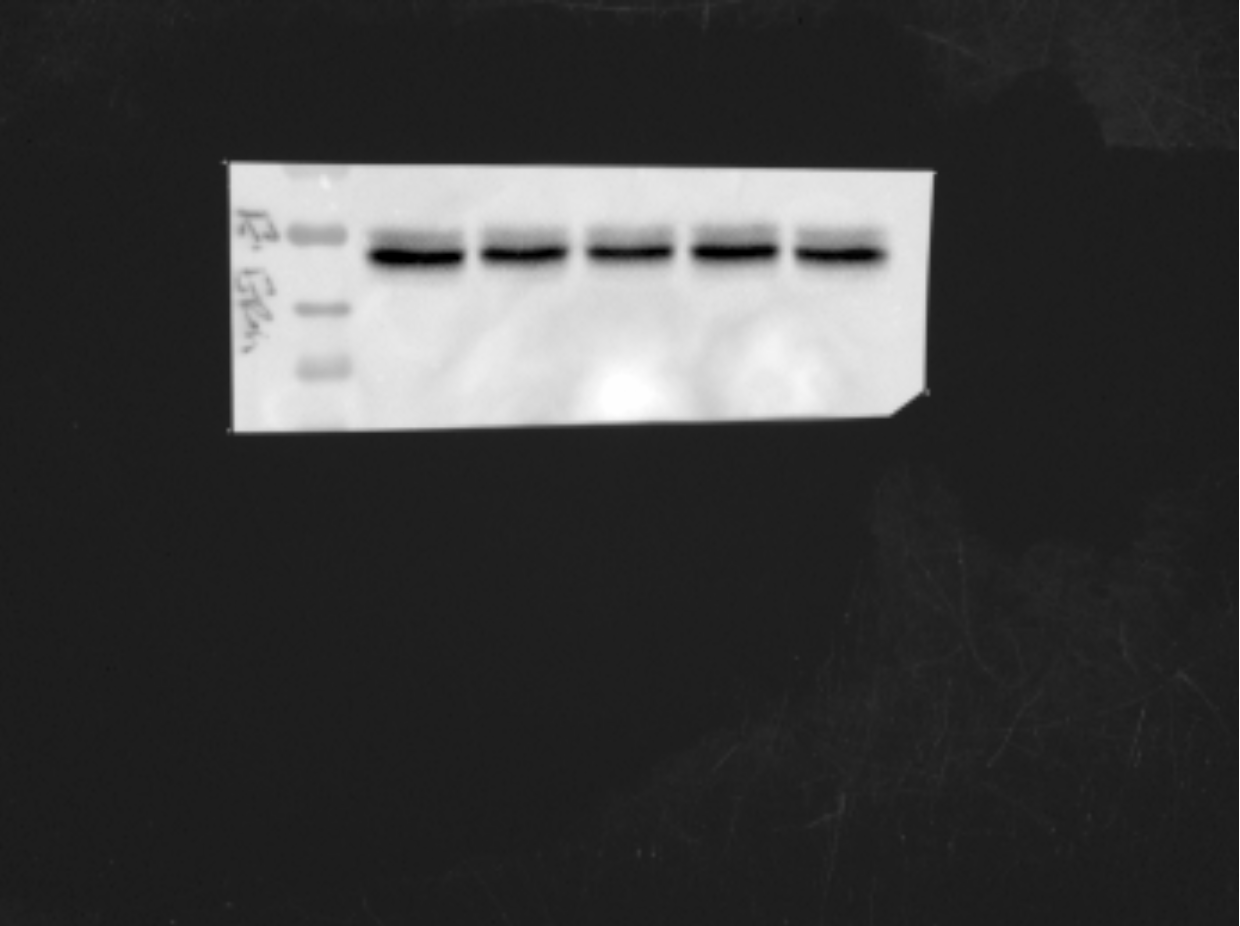

Supplement: Supplementary file 3 — Source data Fig. 2 [file 44321_2026_423_MOESM3_ESM.zip › Figure 2/2F/WB IN ERG.tif]

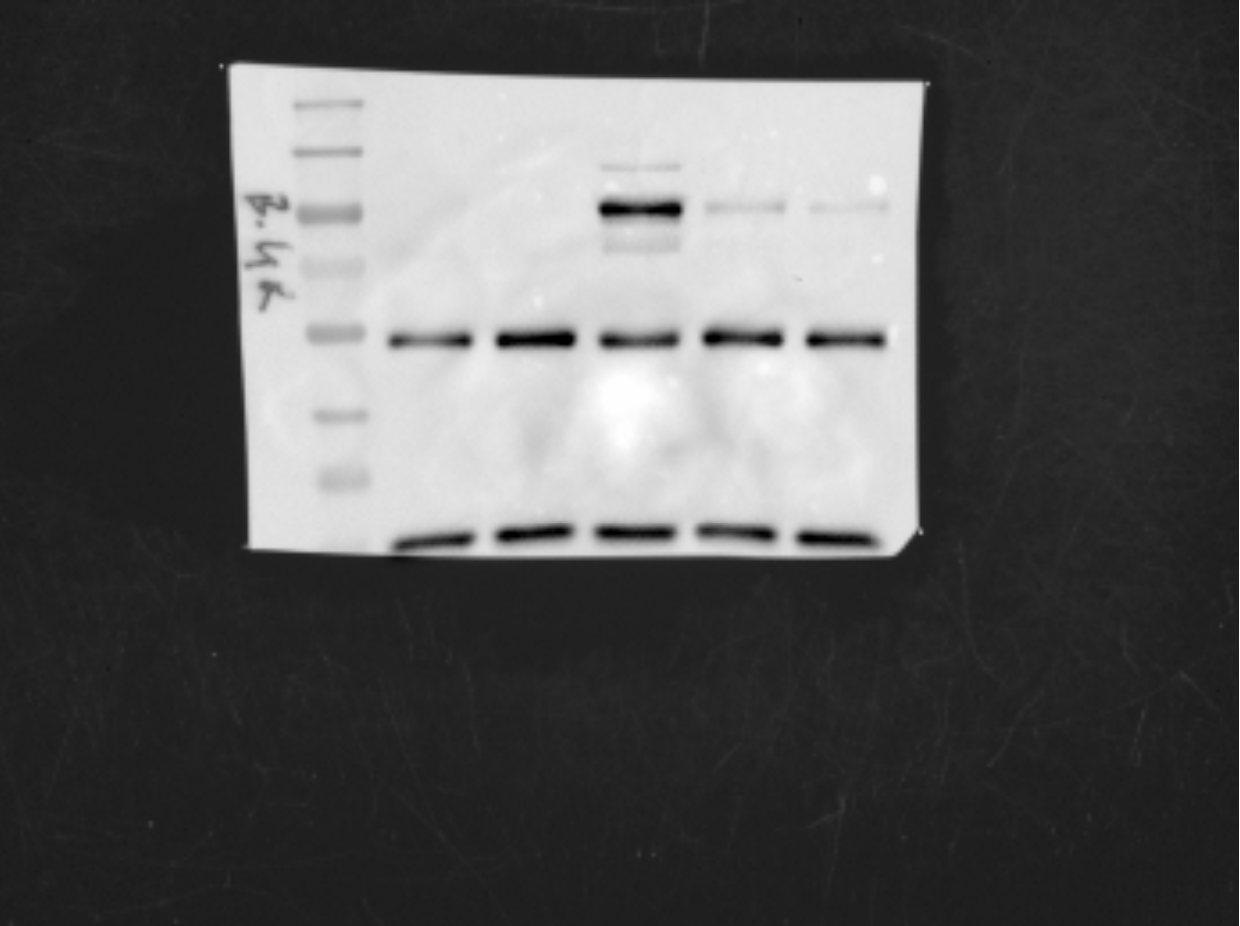

Supplement: Supplementary file 3 — Source data Fig. 2 [file 44321_2026_423_MOESM3_ESM.zip › Figure 2/2F/WB IB .tif]

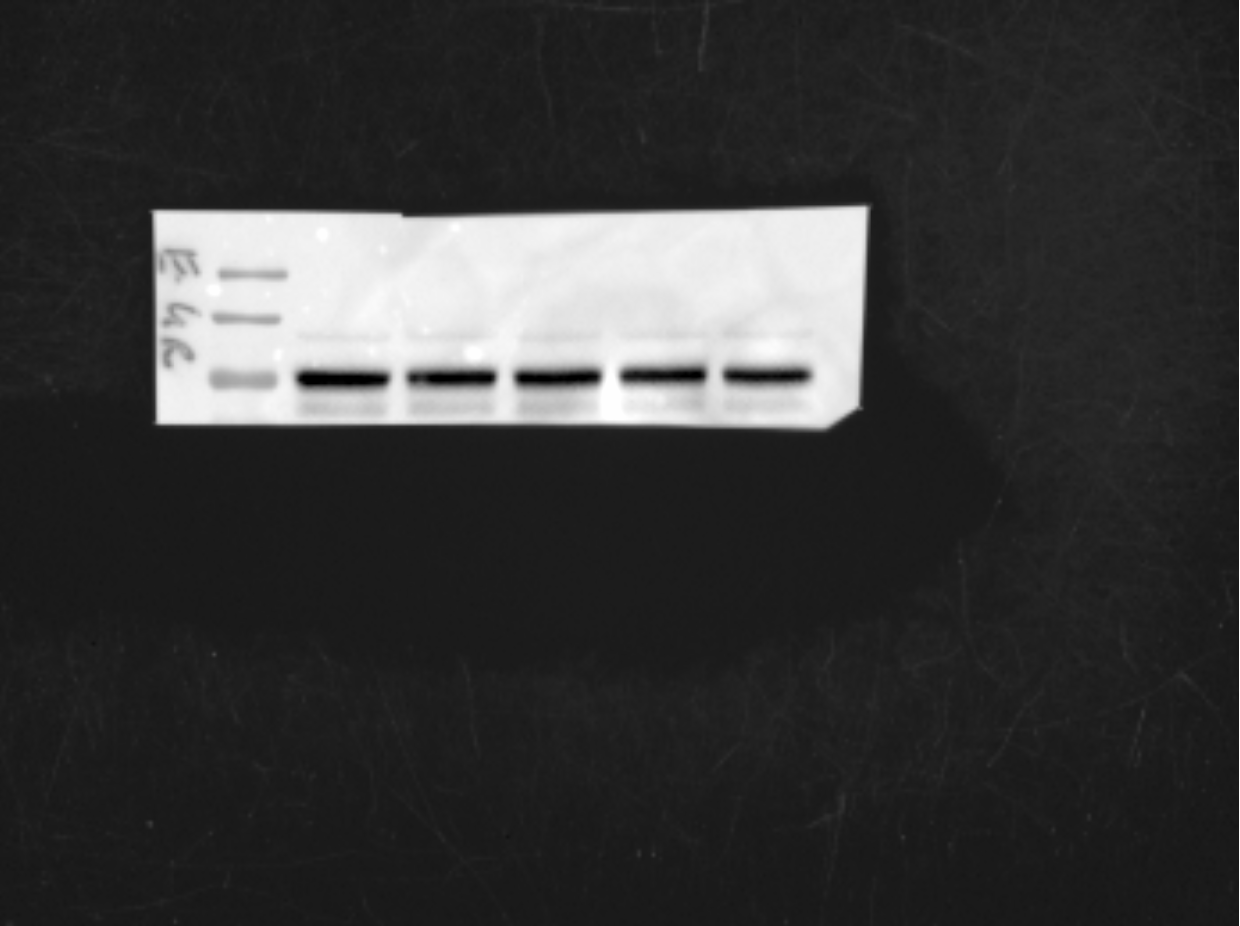

Supplement: Supplementary file 3 — Source data Fig. 2 [file 44321_2026_423_MOESM3_ESM.zip › Figure 2/2F/WB IN GR.tif]

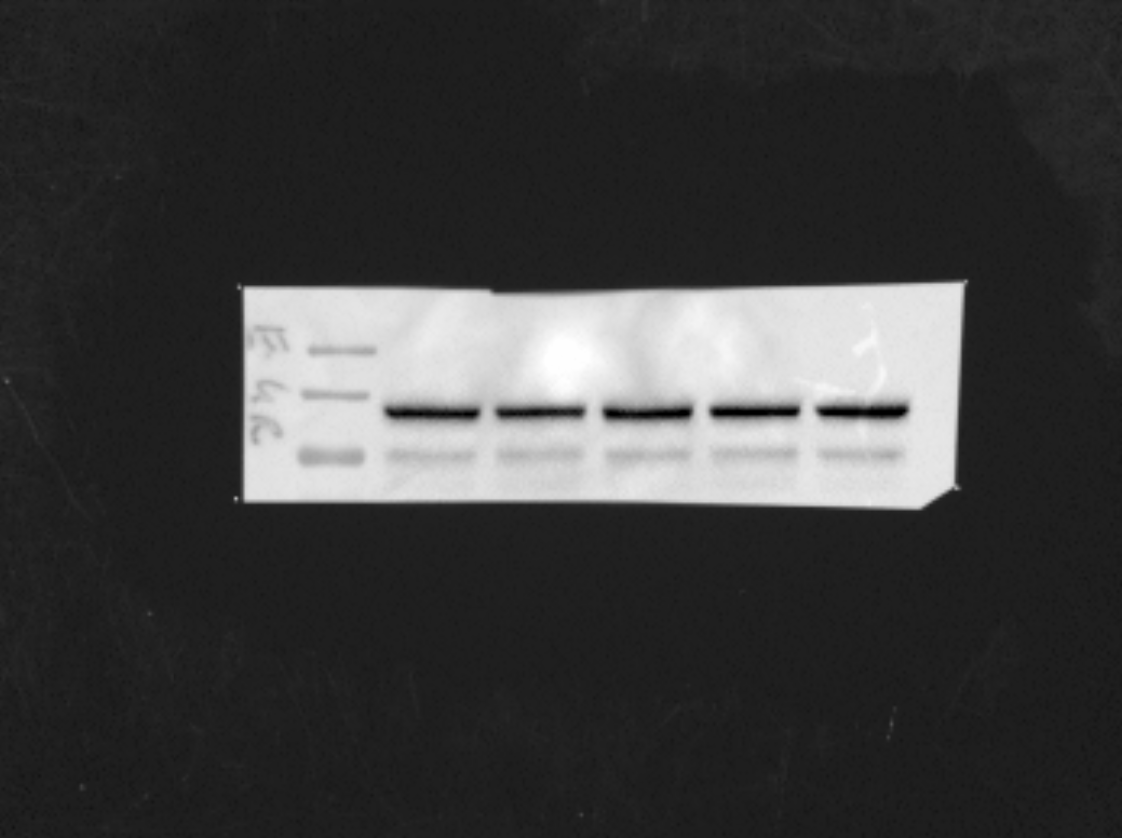

Supplement: Supplementary file 3 — Source data Fig. 2 [file 44321_2026_423_MOESM3_ESM.zip › Figure 2/2F/WB VINCULIN.tif]

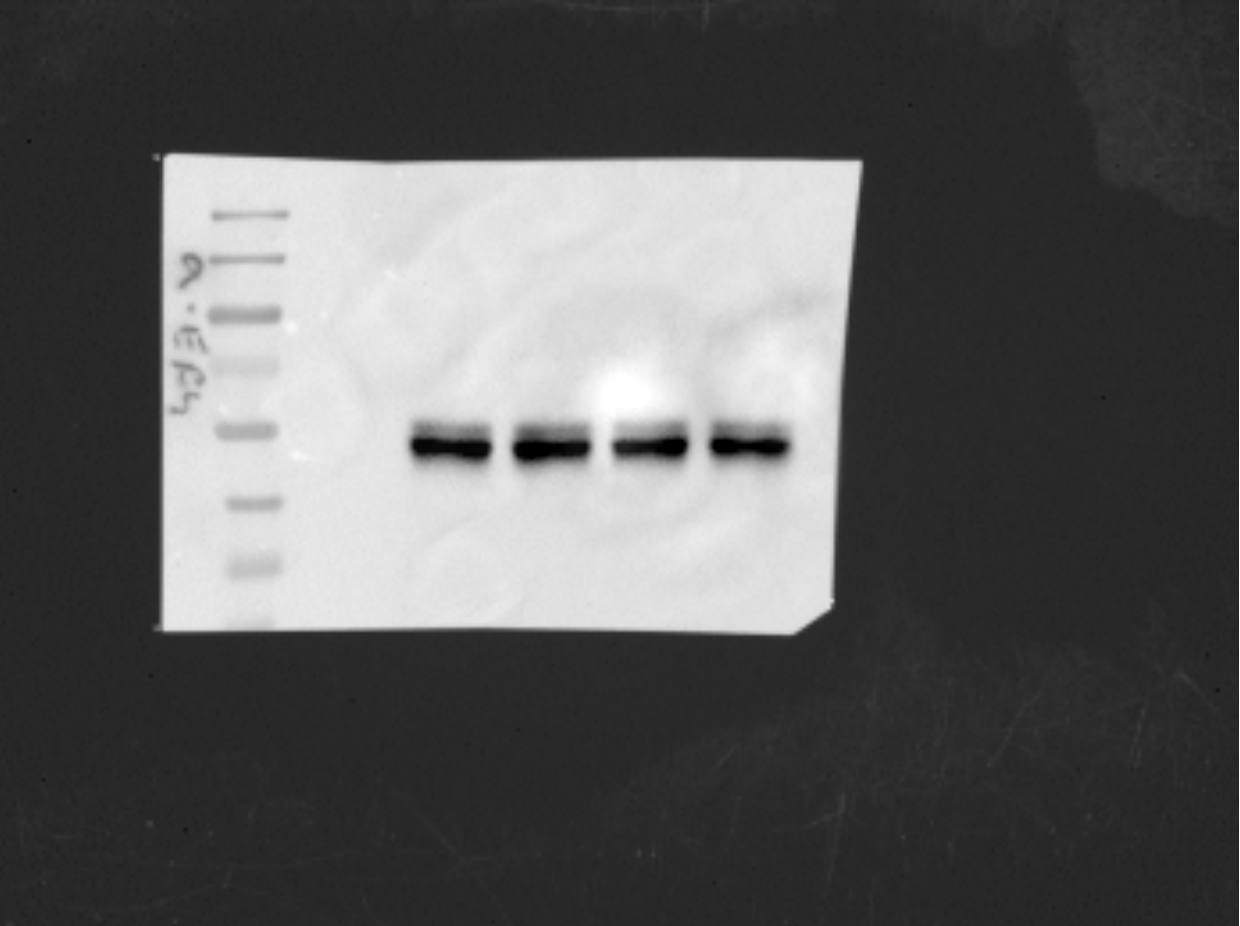

Supplement: Supplementary file 3 — Source data Fig. 2 [file 44321_2026_423_MOESM3_ESM.zip › Figure 2/2F/WB IP ERG IB ERG.tif]

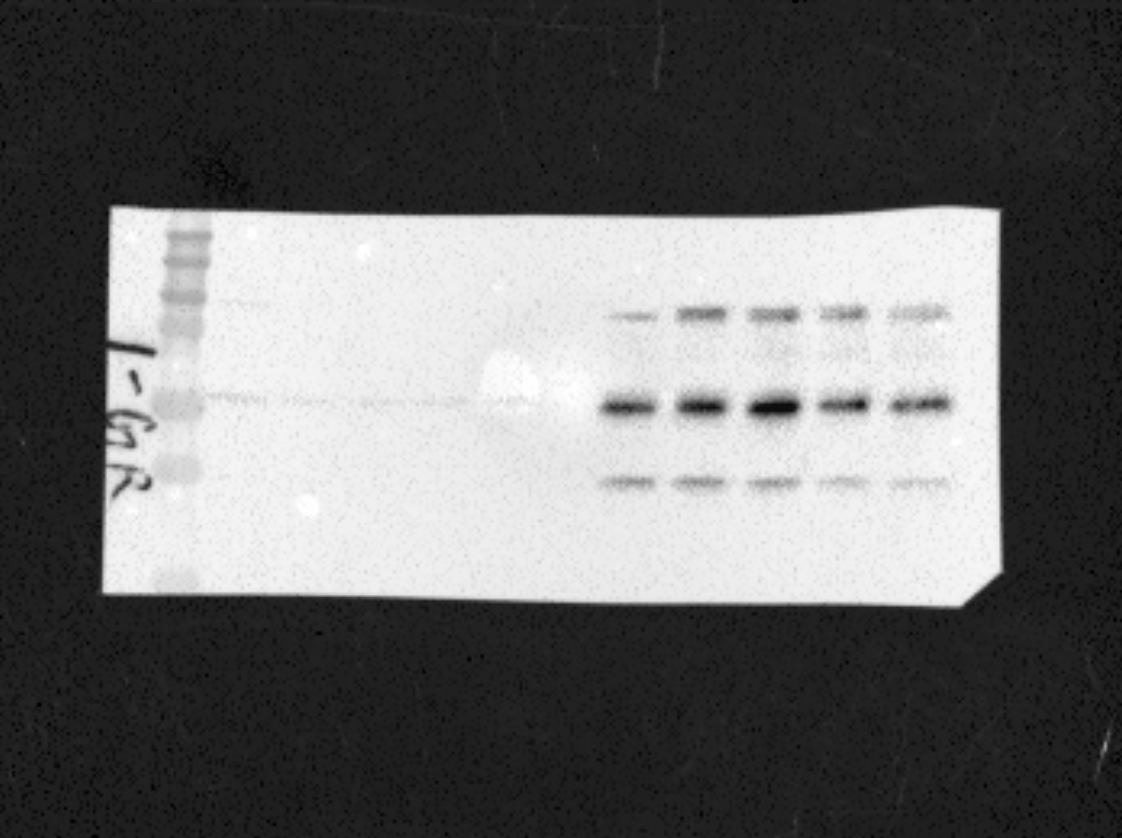

Supplement: Supplementary file 3 — Source data Fig. 2 [file 44321_2026_423_MOESM3_ESM.zip › Figure 2/2C/WB ERG.tif]

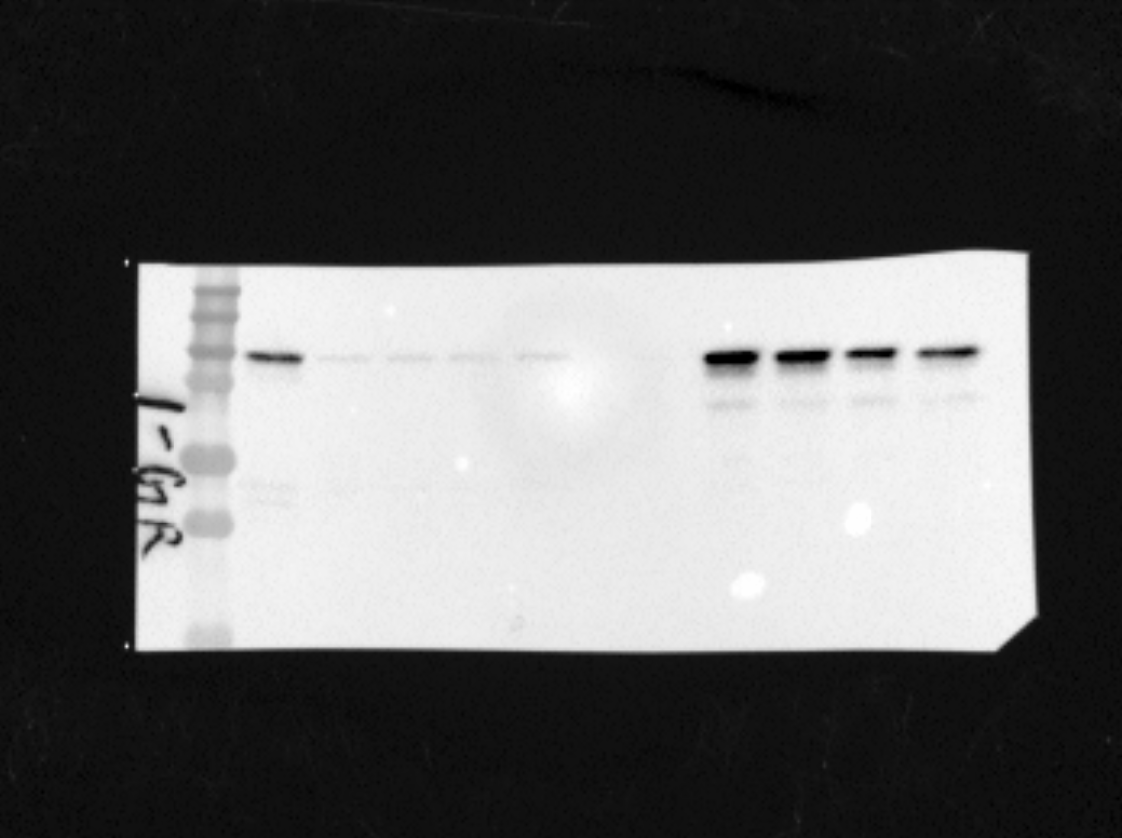

Supplement: Supplementary file 3 — Source data Fig. 2 [file 44321_2026_423_MOESM3_ESM.zip › Figure 2/2C/WB GR.tif]

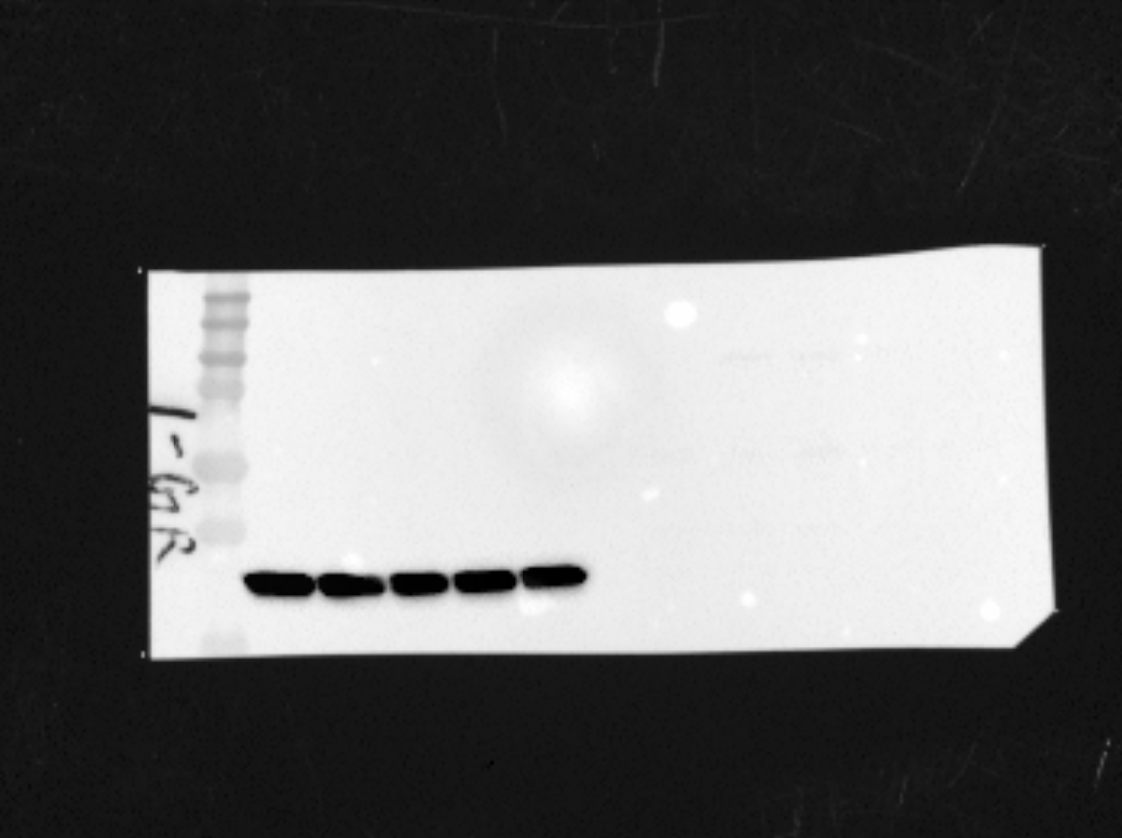

Supplement: Supplementary file 3 — Source data Fig. 2 [file 44321_2026_423_MOESM3_ESM.zip › Figure 2/2C/WB LDH.tif]

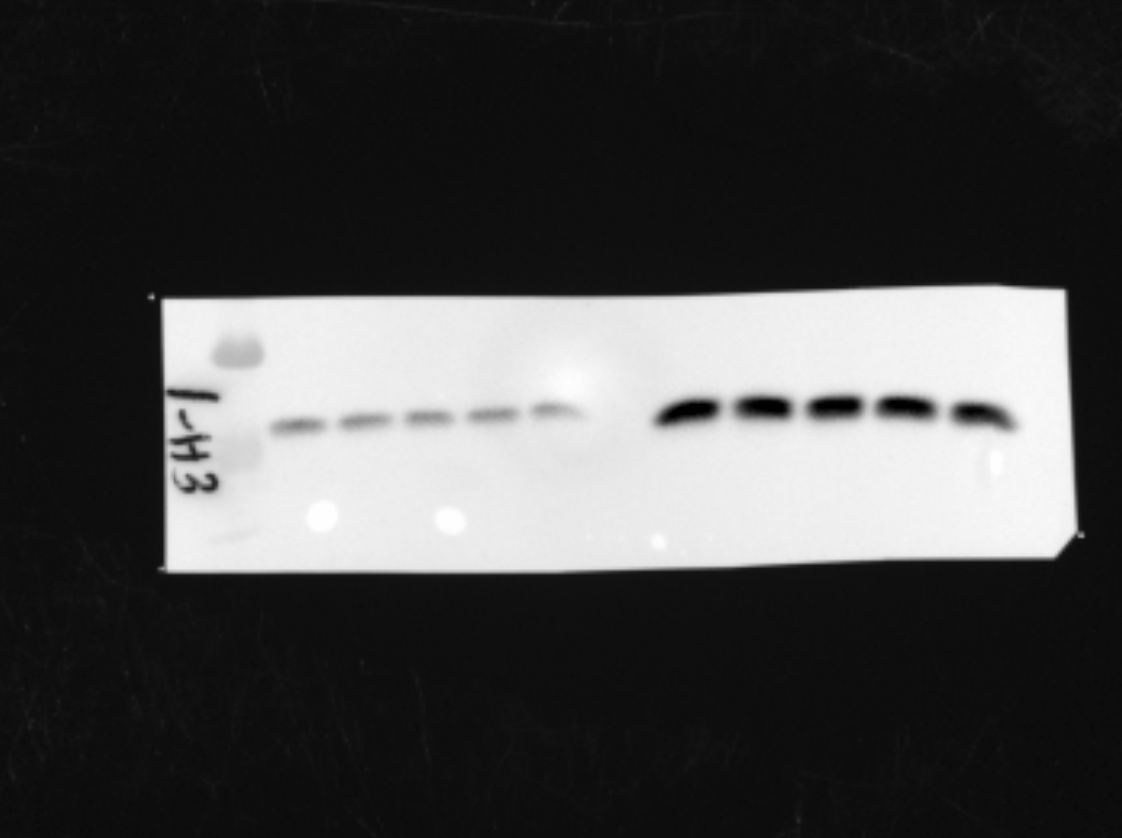

Supplement: Supplementary file 3 — Source data Fig. 2 [file 44321_2026_423_MOESM3_ESM.zip › Figure 2/2C/WB Histone3.tif]

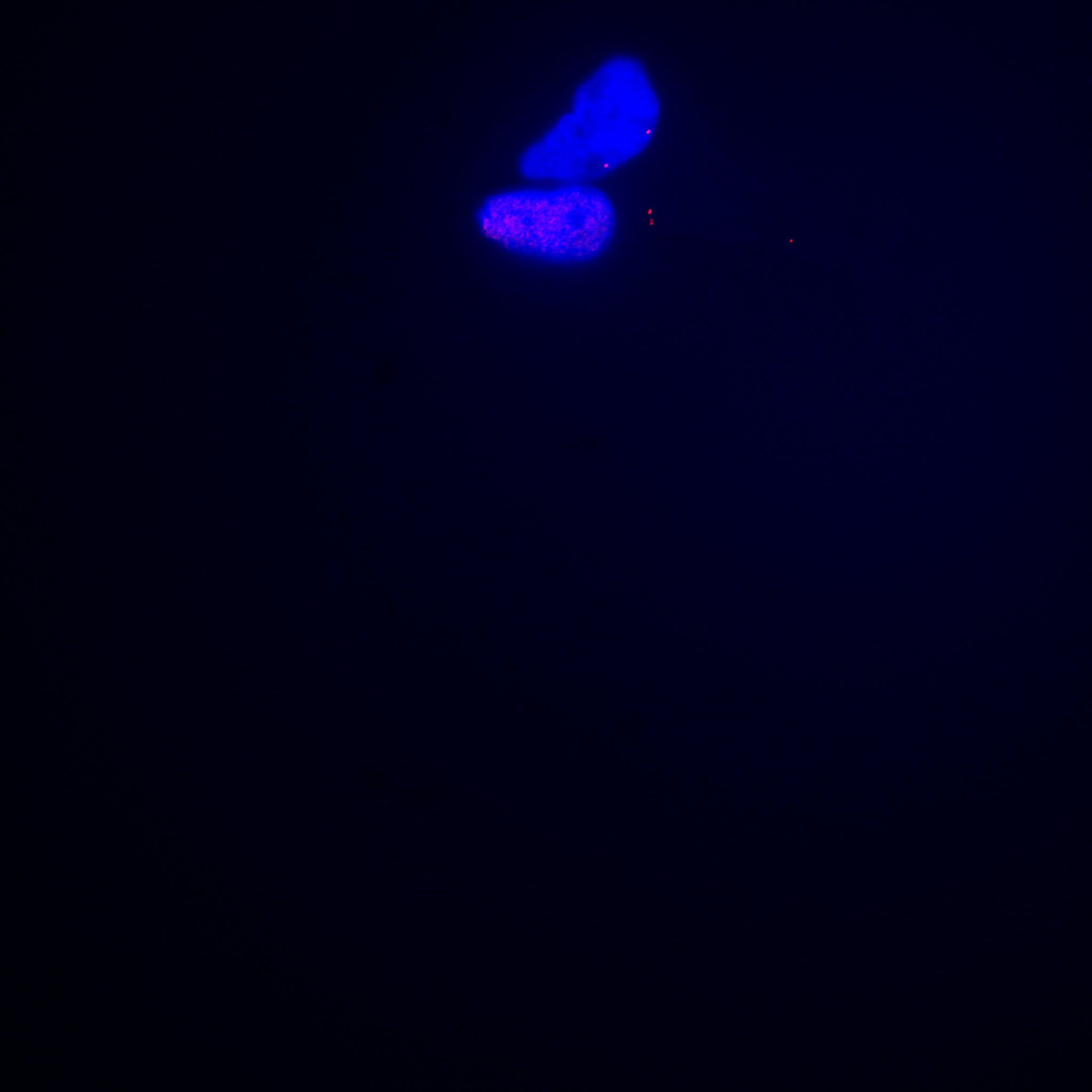

Supplement: Supplementary file 3 — Source data Fig. 2 [file 44321_2026_423_MOESM3_ESM.zip › Figure 2/2D/PLA control.jpg]

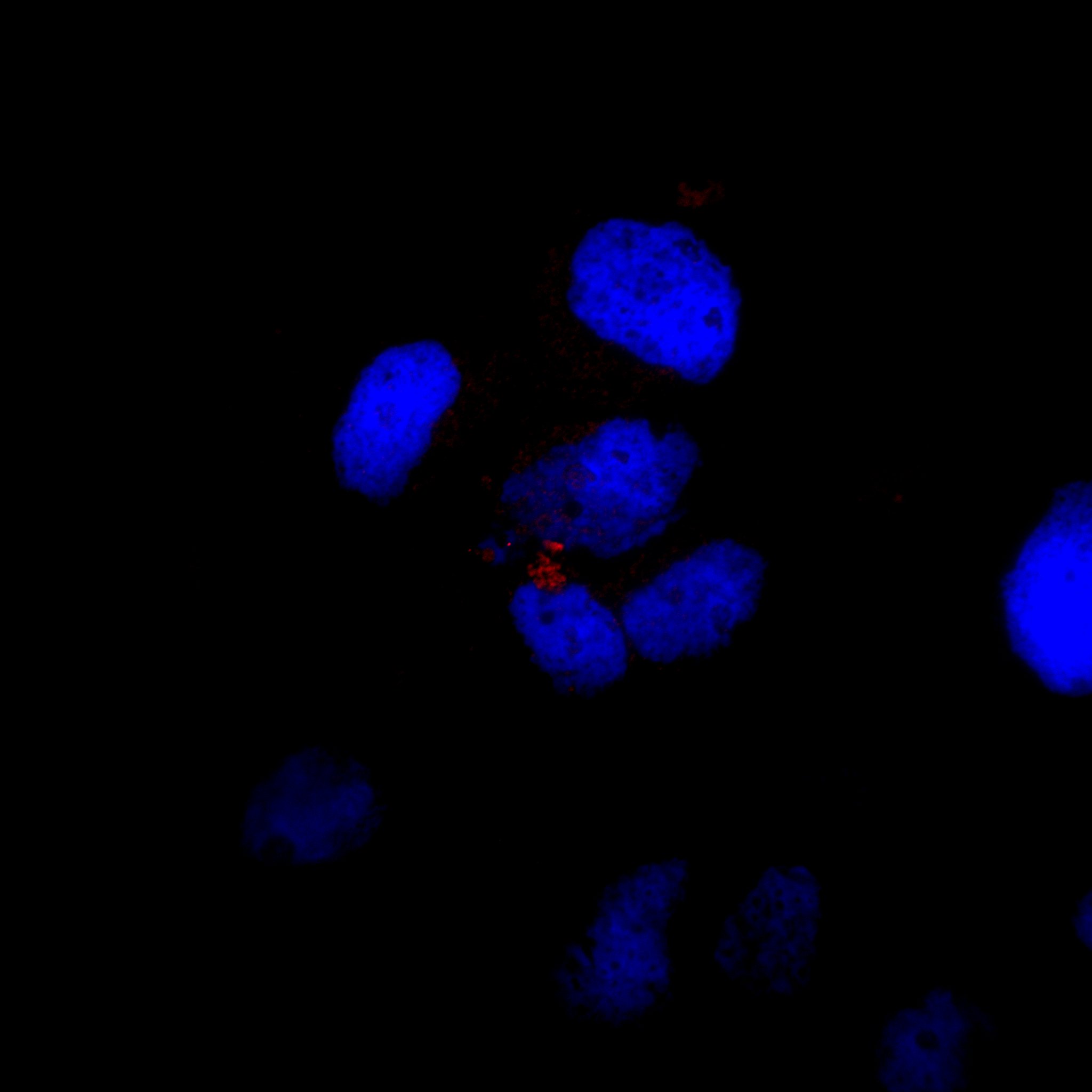

Supplement: Supplementary file 3 — Source data Fig. 2 [file 44321_2026_423_MOESM3_ESM.zip › Figure 2/2D/PLA DEX RU.jpg]

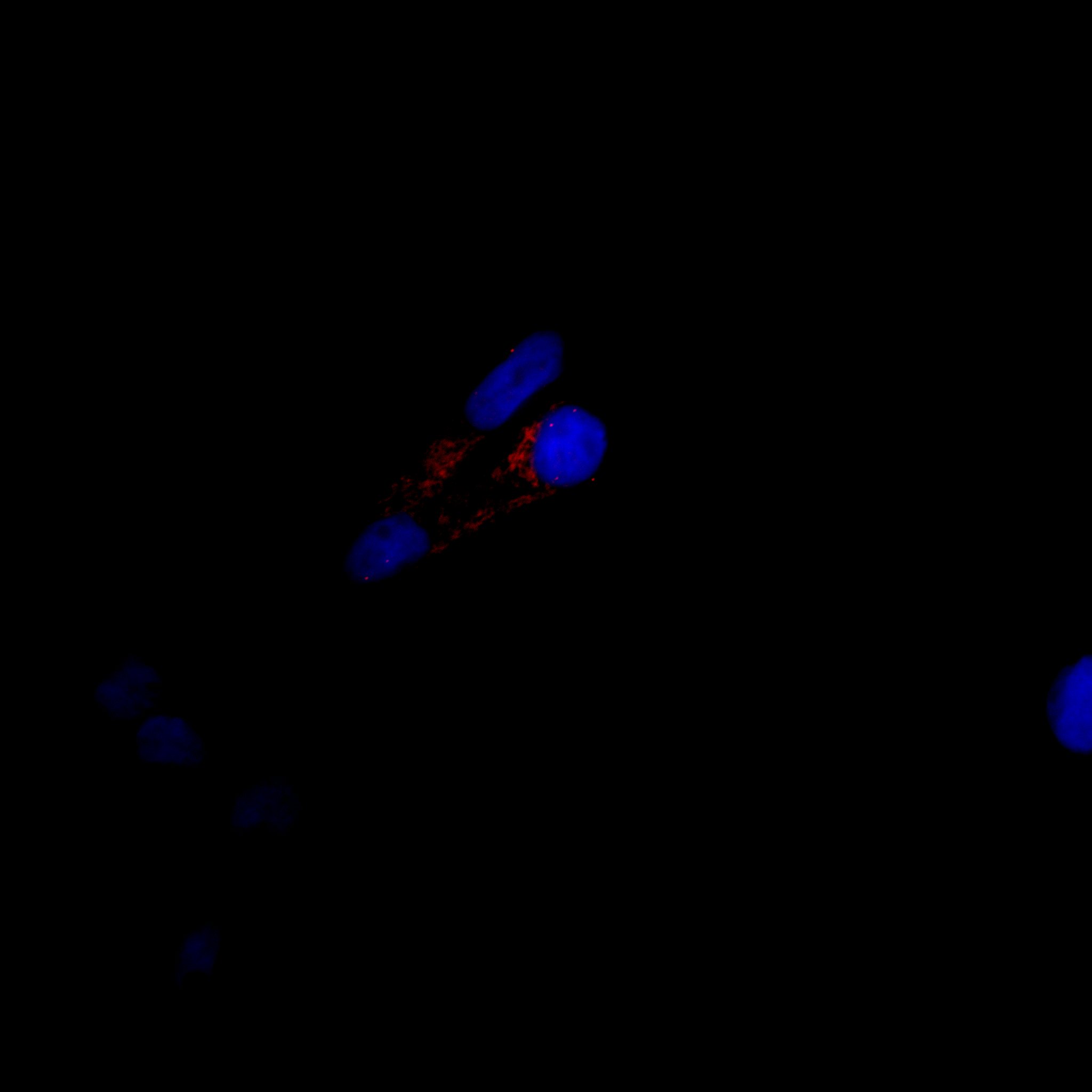

Supplement: Supplementary file 3 — Source data Fig. 2 [file 44321_2026_423_MOESM3_ESM.zip › Figure 2/2D/PLA DEX.jpg]

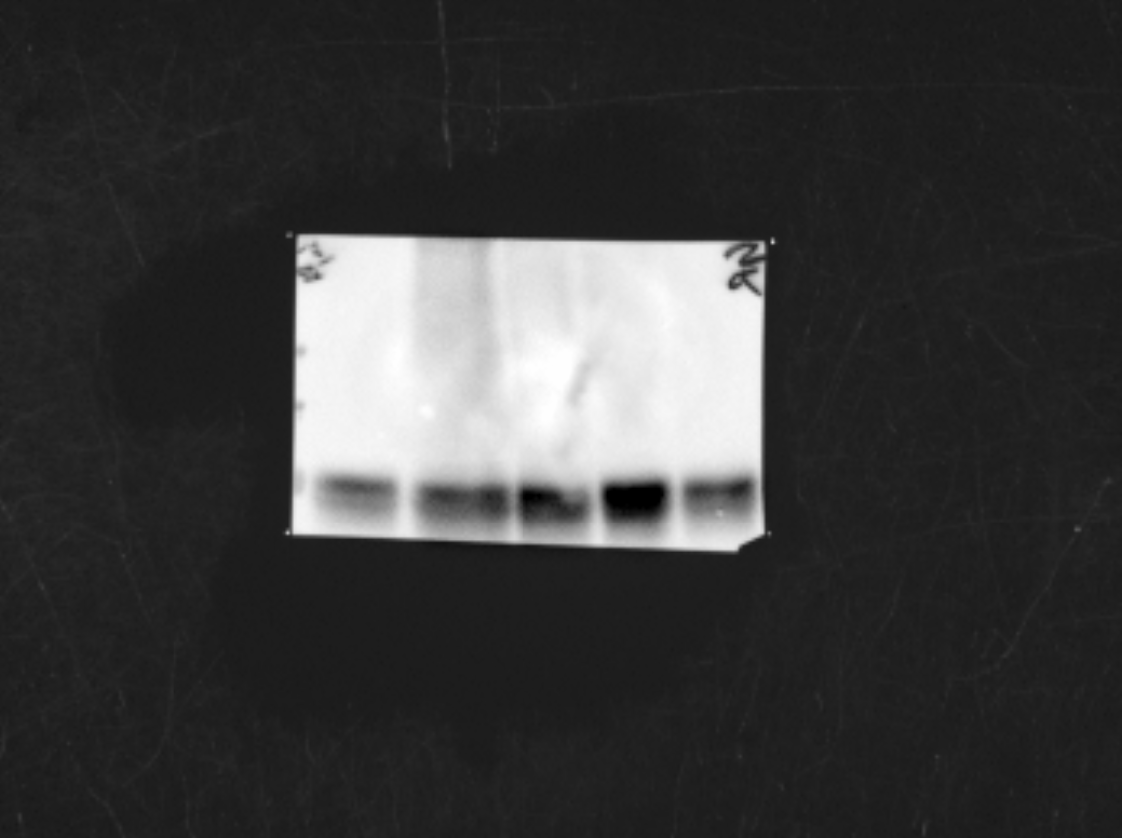

Supplement: Supplementary file 3 — Source data Fig. 2 [file 44321_2026_423_MOESM3_ESM.zip › Figure 2/2E/WB IB GR.tif]

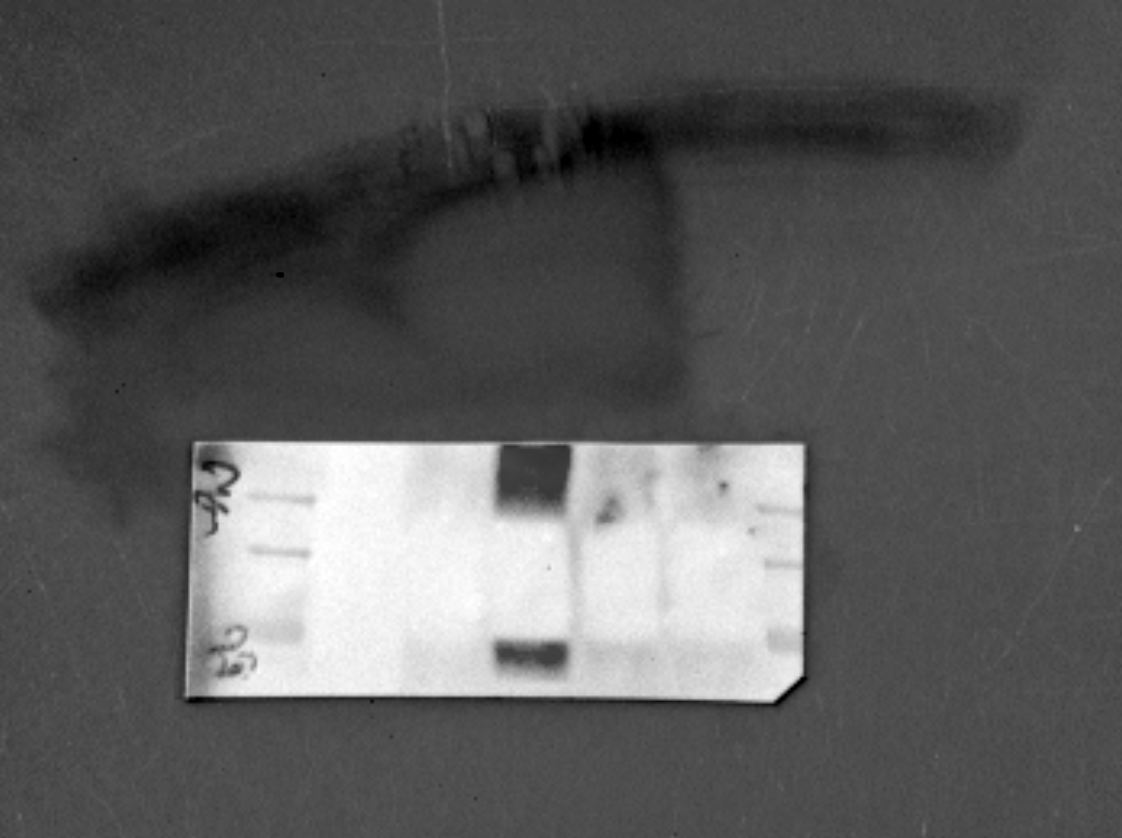

Supplement: Supplementary file 3 — Source data Fig. 2 [file 44321_2026_423_MOESM3_ESM.zip › Figure 2/2E/WB IP ERG IB GR.tif]

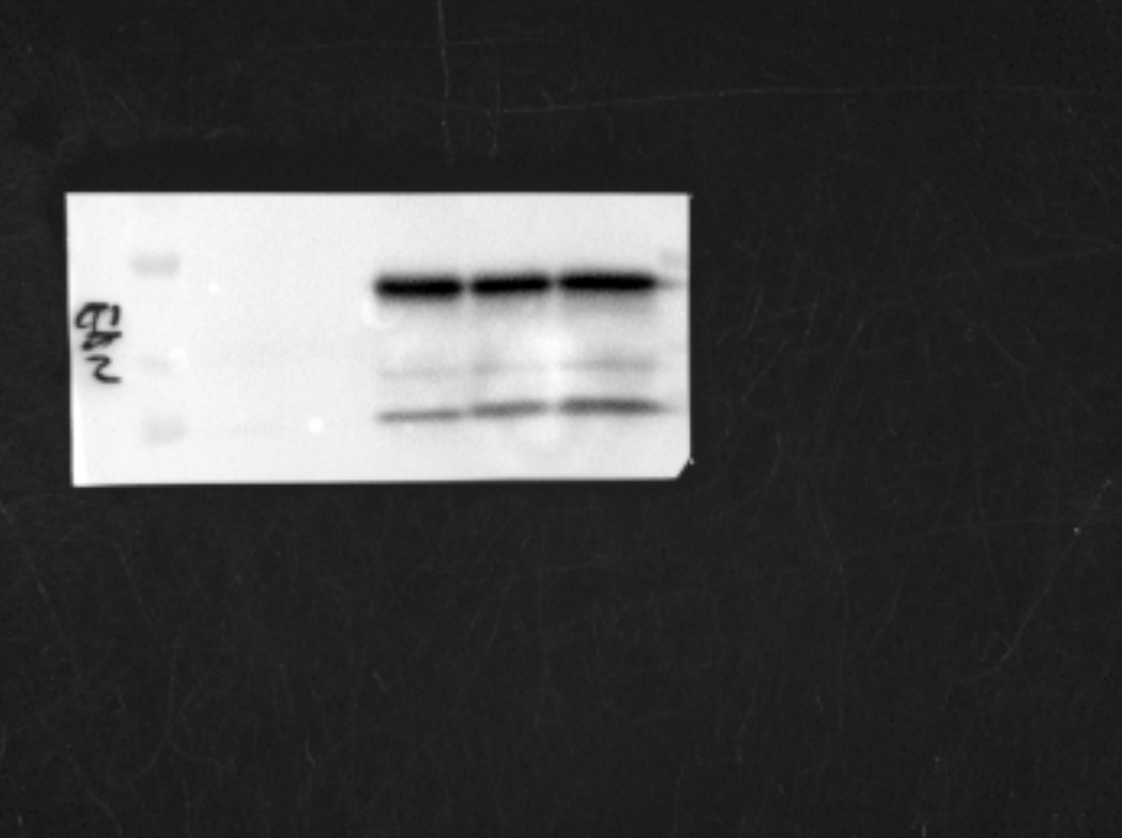

Supplement: Supplementary file 3 — Source data Fig. 2 [file 44321_2026_423_MOESM3_ESM.zip › Figure 2/2E/WB IP ERG IB ERG.tif]

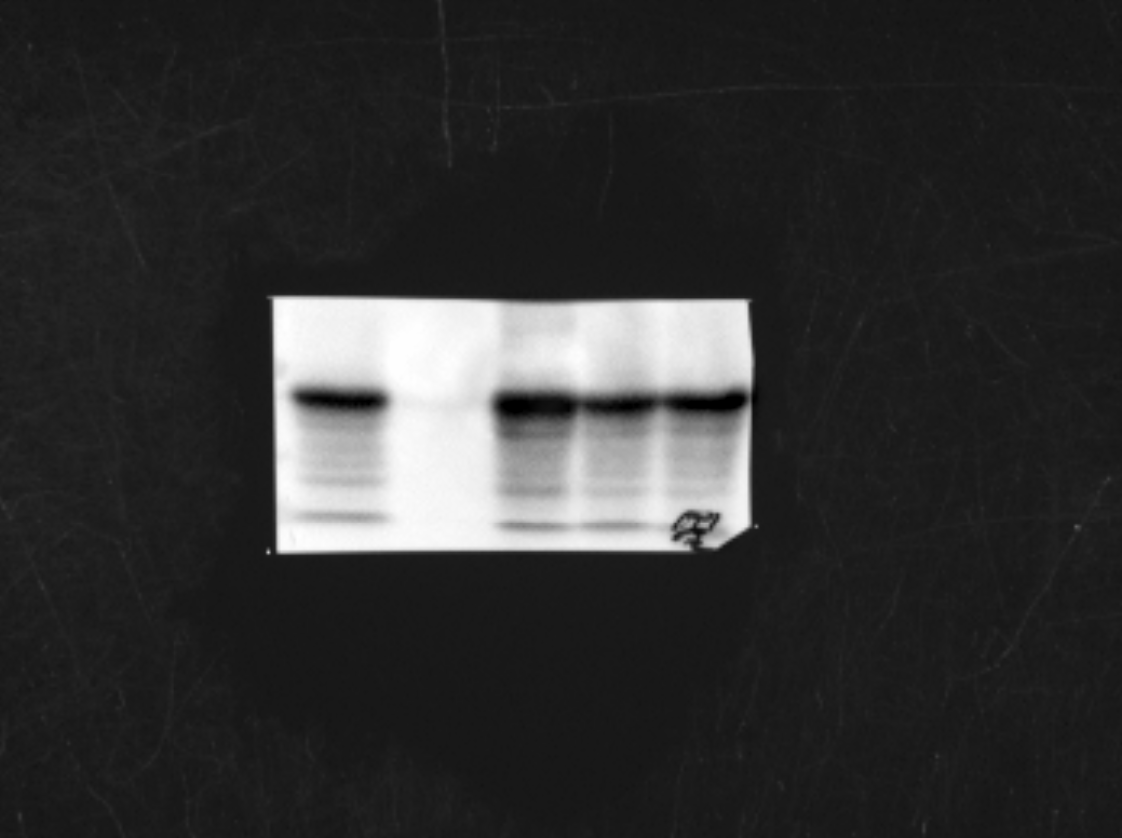

Supplement: Supplementary file 3 — Source data Fig. 2 [file 44321_2026_423_MOESM3_ESM.zip › Figure 2/2E/WB IB ERG.tif]

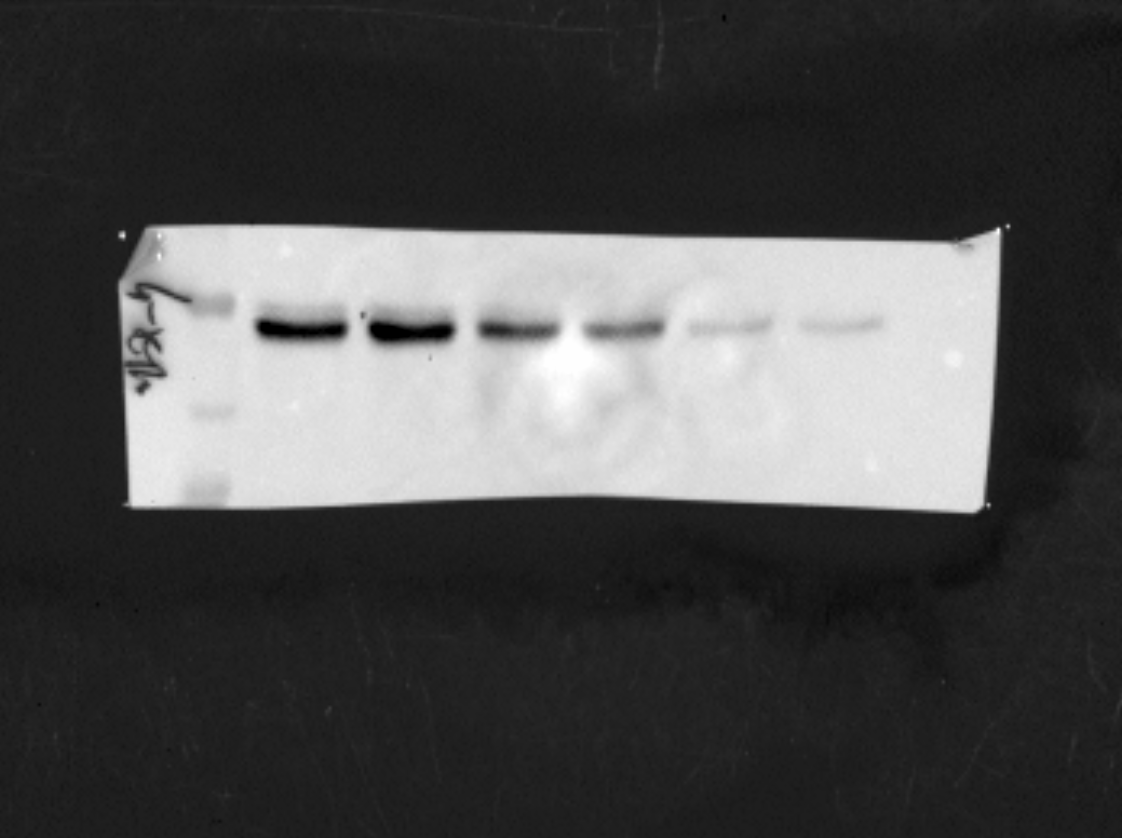

Supplement: Supplementary file 4 — Source data Fig. 3 [file 44321_2026_423_MOESM4_ESM.zip › Figure 3/3E/WB ERG.tif]

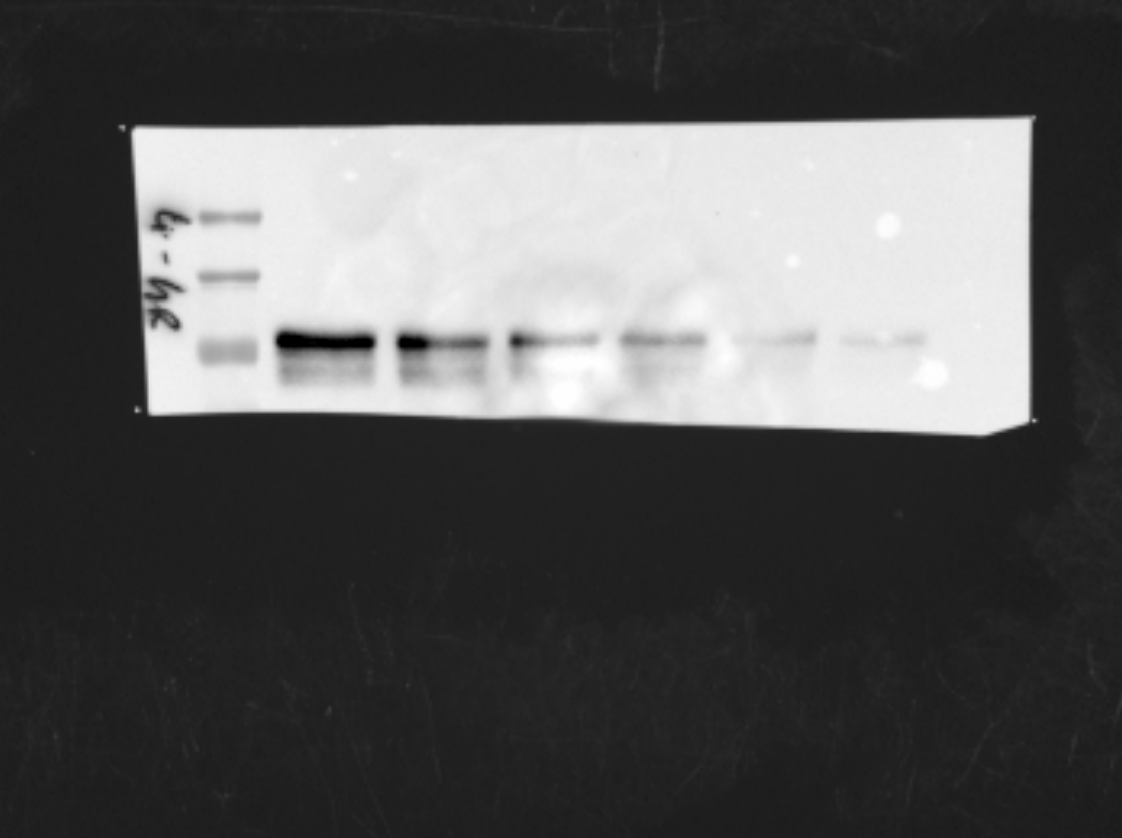

Supplement: Supplementary file 4 — Source data Fig. 3 [file 44321_2026_423_MOESM4_ESM.zip › Figure 3/3E/WB GR.tif]

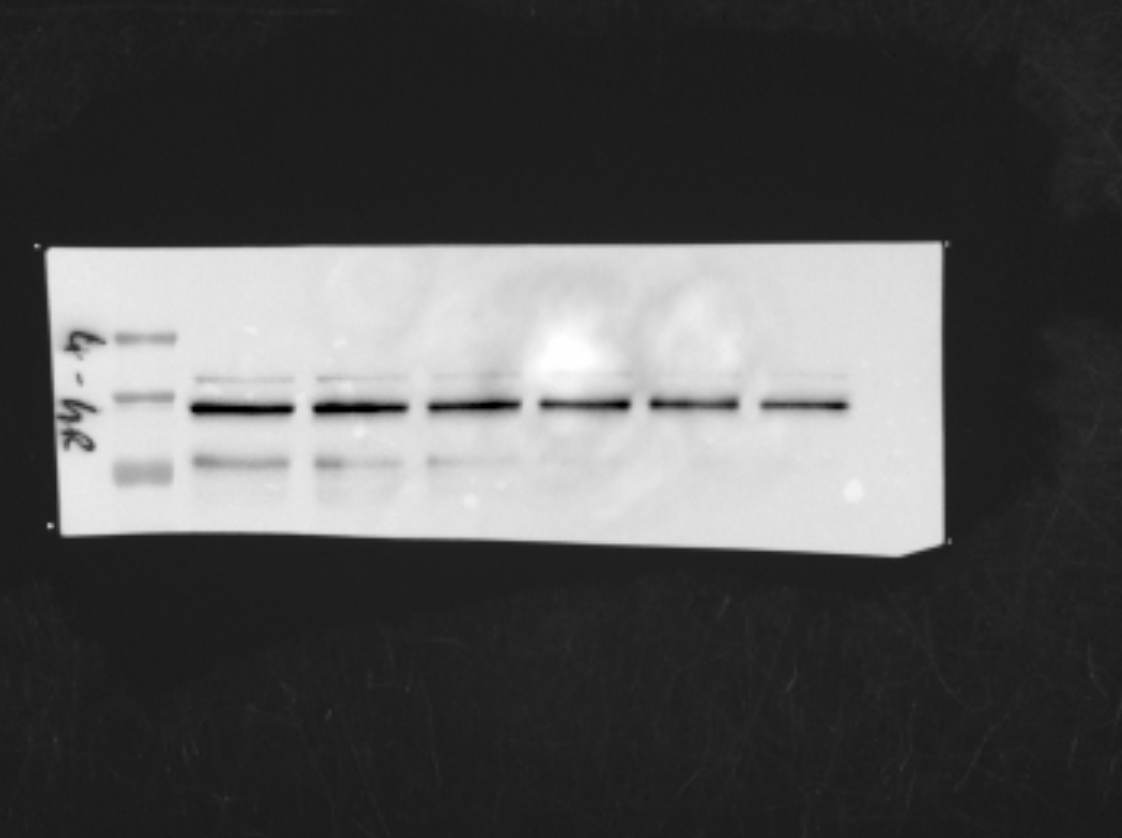

Supplement: Supplementary file 4 — Source data Fig. 3 [file 44321_2026_423_MOESM4_ESM.zip › Figure 3/3E/WB VINCULIN.tif]

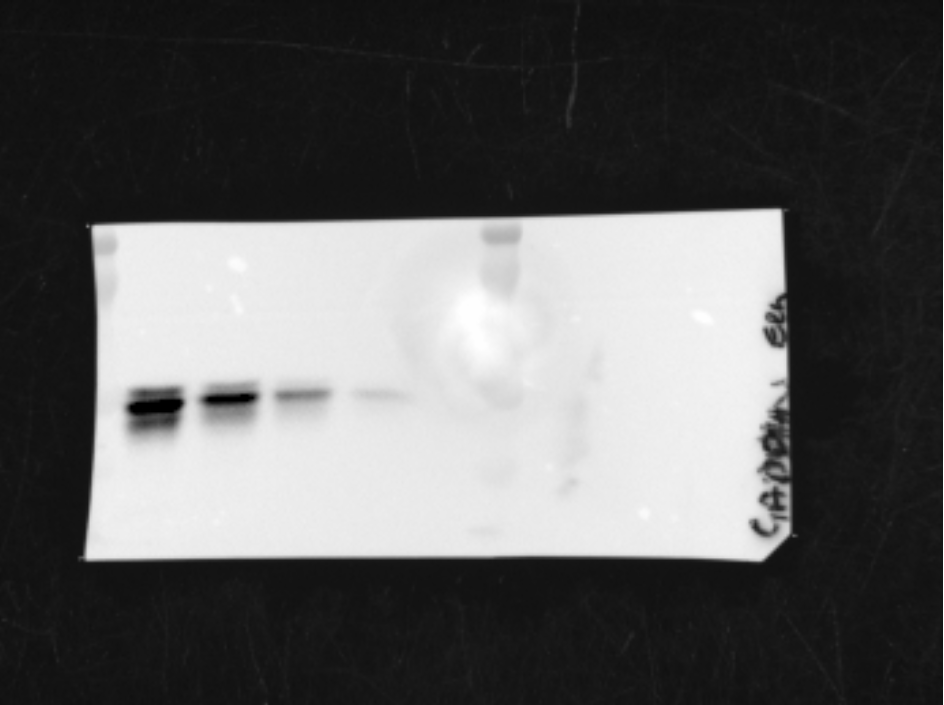

Supplement: Supplementary file 4 — Source data Fig. 3 [file 44321_2026_423_MOESM4_ESM.zip › Figure 3/3B/WB ERG.tif]

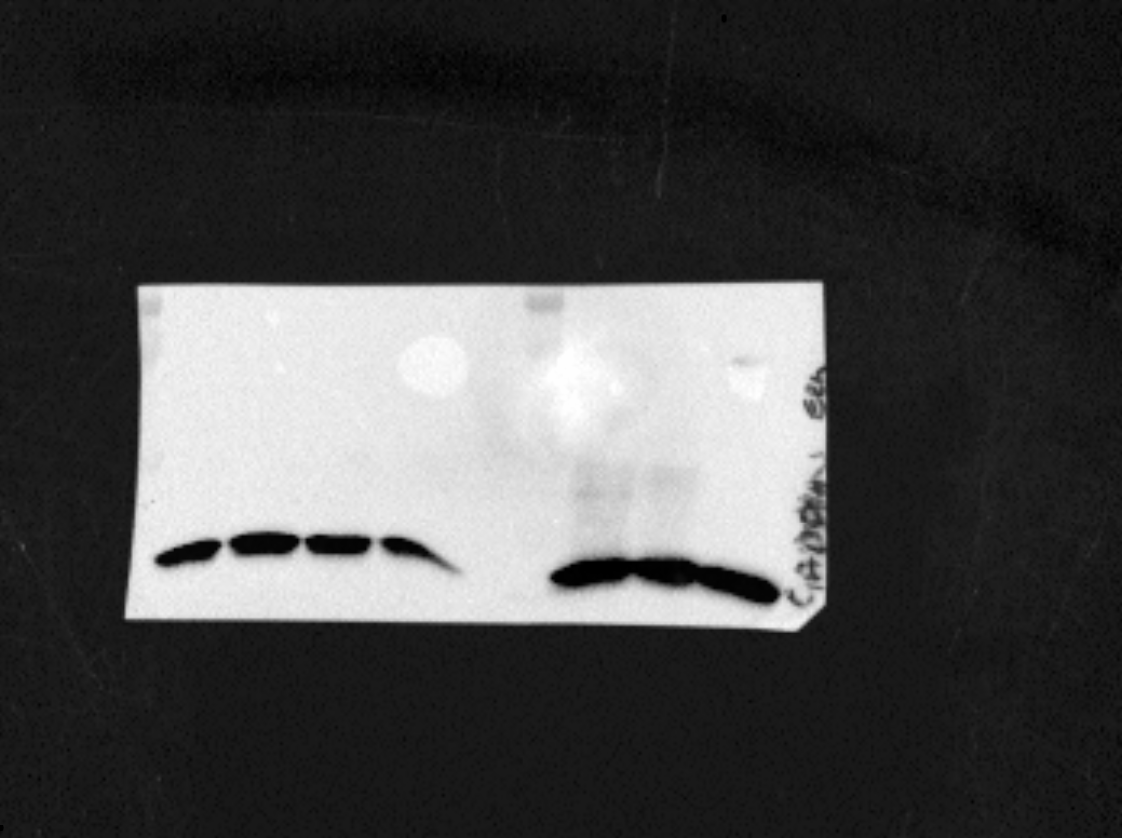

Supplement: Supplementary file 4 — Source data Fig. 3 [file 44321_2026_423_MOESM4_ESM.zip › Figure 3/3B/WB GAPDH.tif]

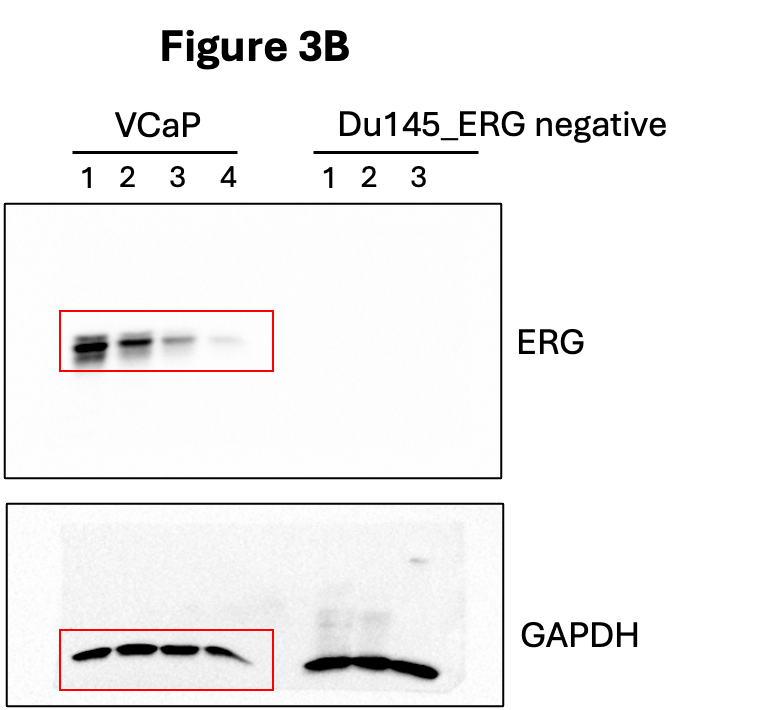

Supplement: Supplementary file 4 — Source data Fig. 3 [file 44321_2026_423_MOESM4_ESM.zip › Figure 3/3B/3b.png]

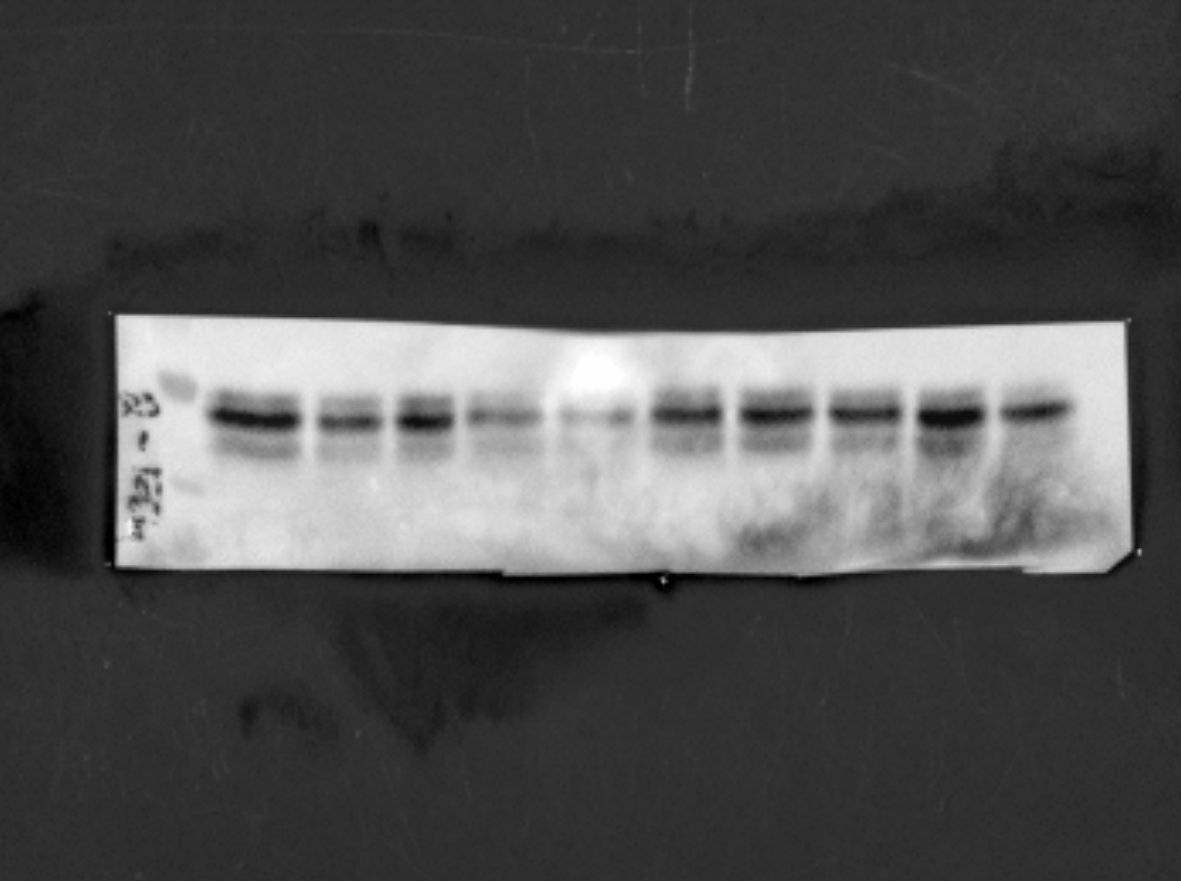

Supplement: Supplementary file 4 — Source data Fig. 3 [file 44321_2026_423_MOESM4_ESM.zip › Figure 3/3D/WB ERG.tif]

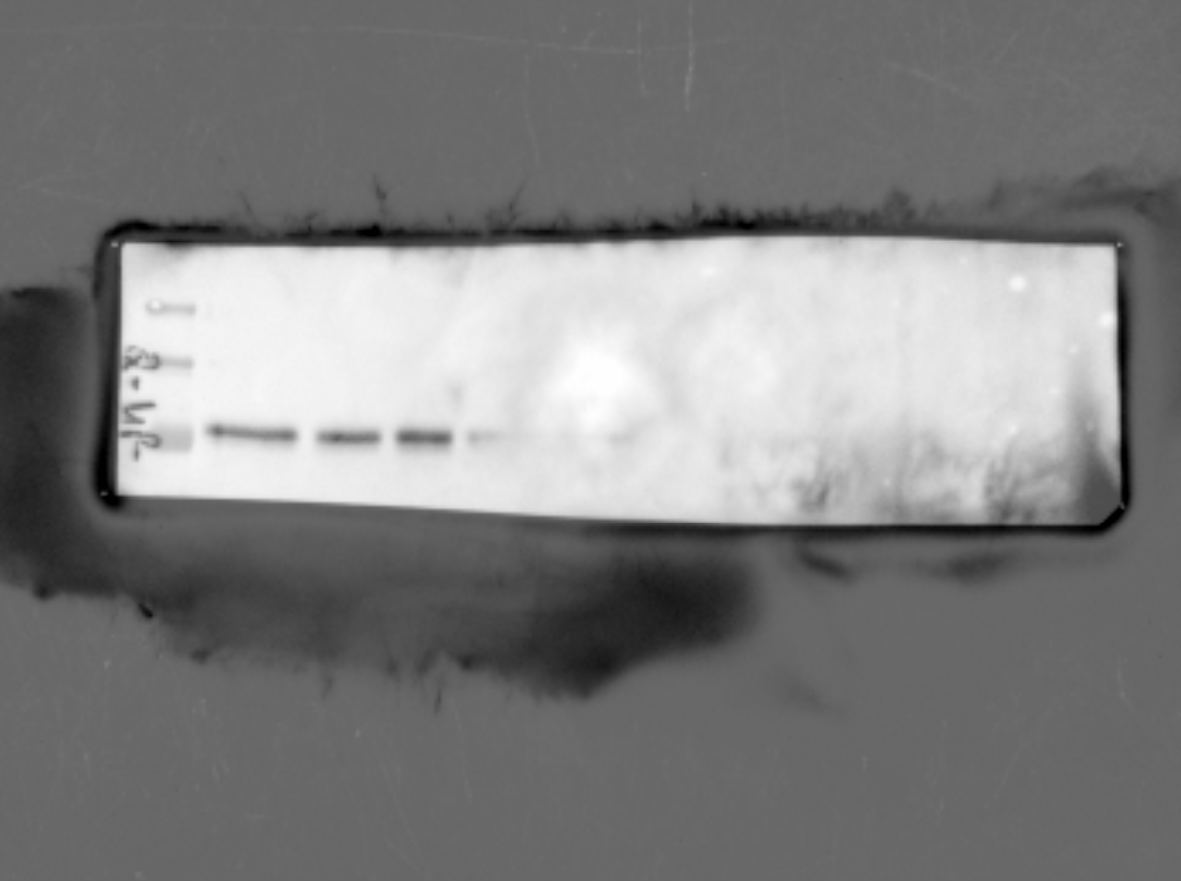

Supplement: Supplementary file 4 — Source data Fig. 3 [file 44321_2026_423_MOESM4_ESM.zip › Figure 3/3D/WB GR.tif]

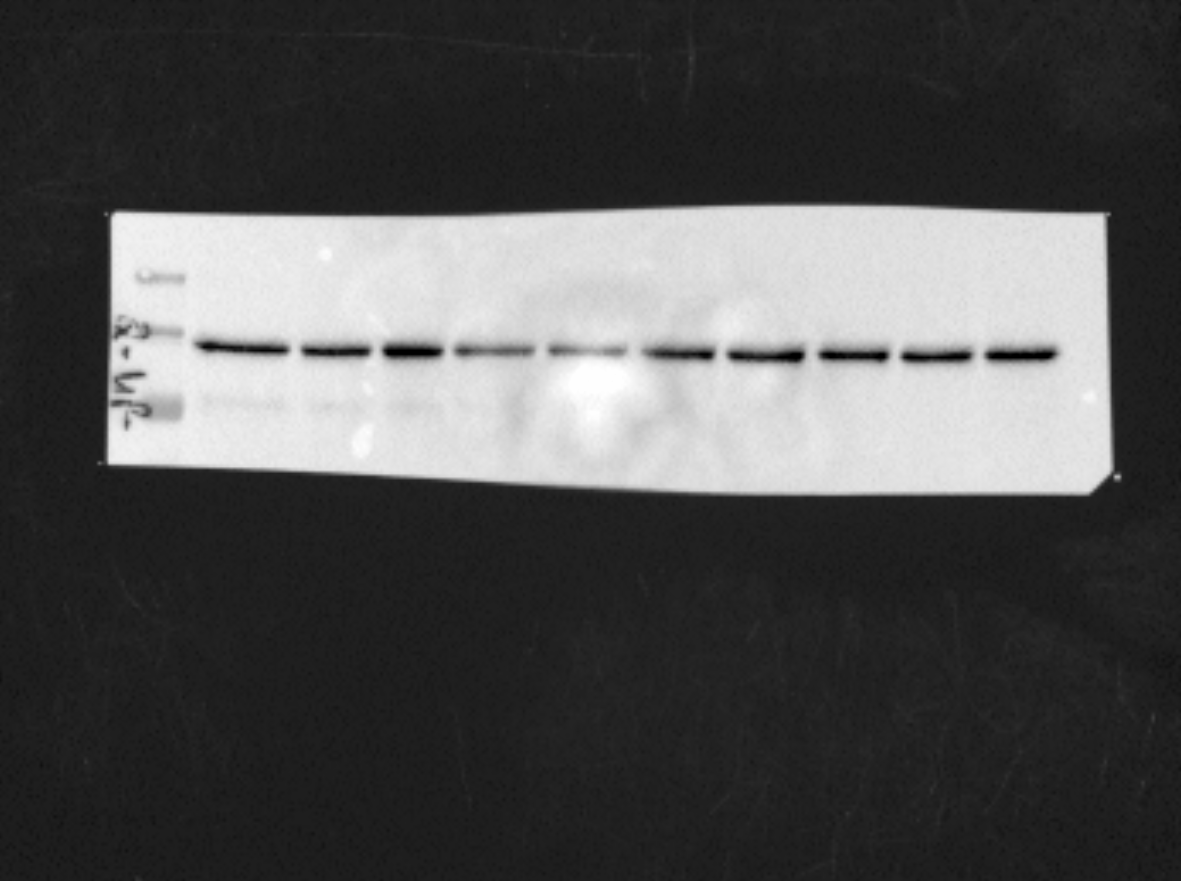

Supplement: Supplementary file 4 — Source data Fig. 3 [file 44321_2026_423_MOESM4_ESM.zip › Figure 3/3D/WB VINCULIN.tif]

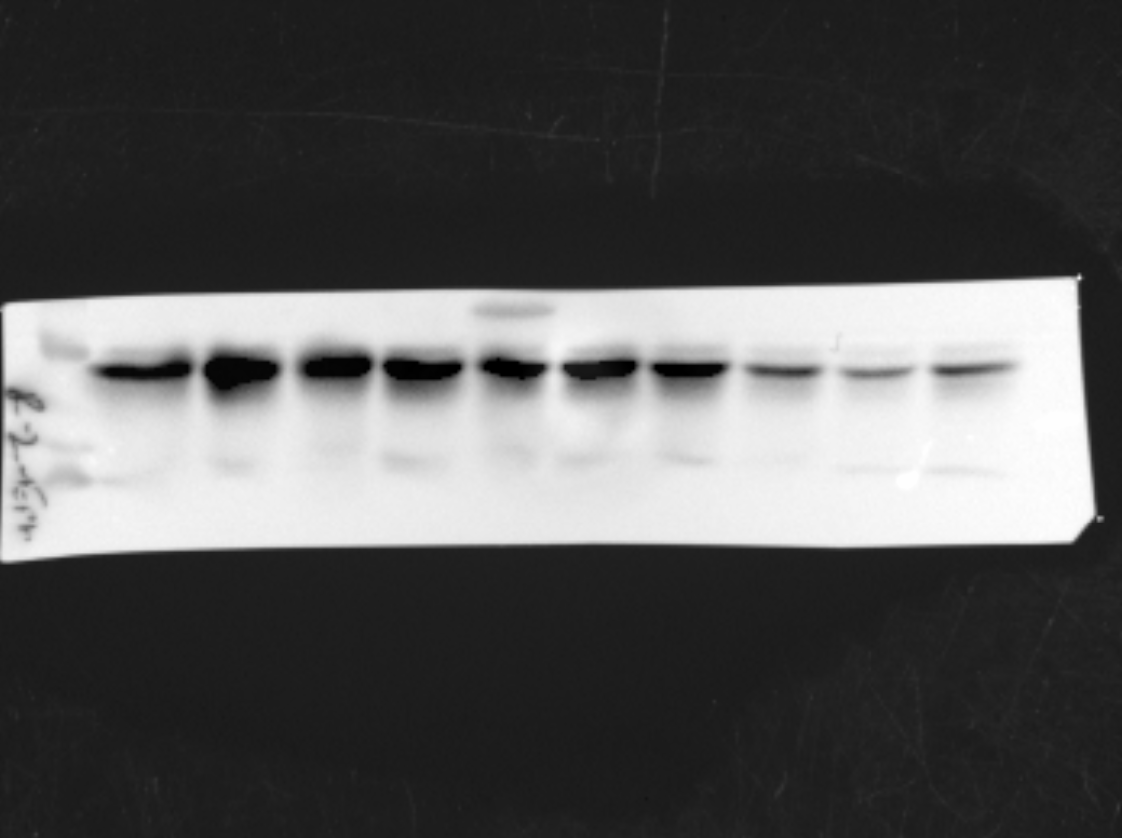

Supplement: Supplementary file 4 — Source data Fig. 3 [file 44321_2026_423_MOESM4_ESM.zip › Figure 3/3A/WB ERG.tif]

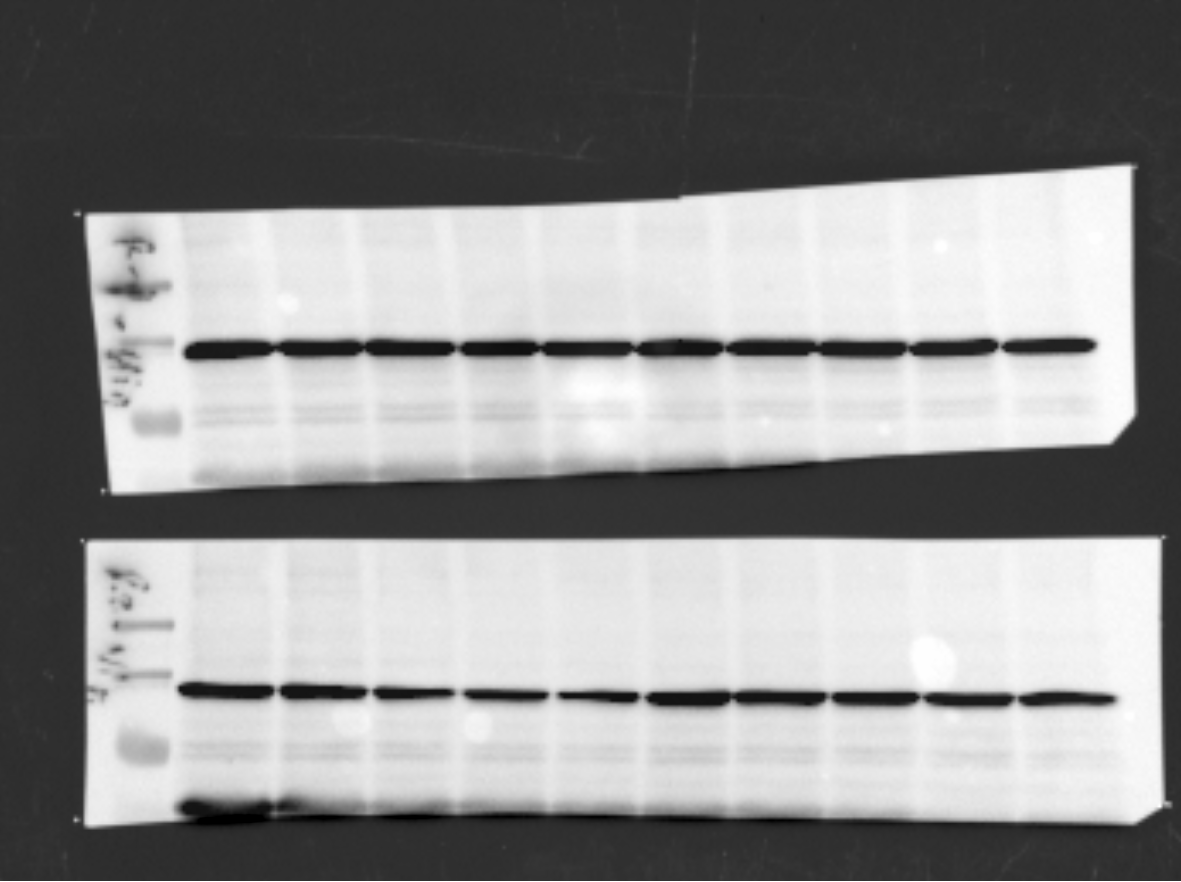

Supplement: Supplementary file 4 — Source data Fig. 3 [file 44321_2026_423_MOESM4_ESM.zip › Figure 3/3A/WB VINCULIN.tif]

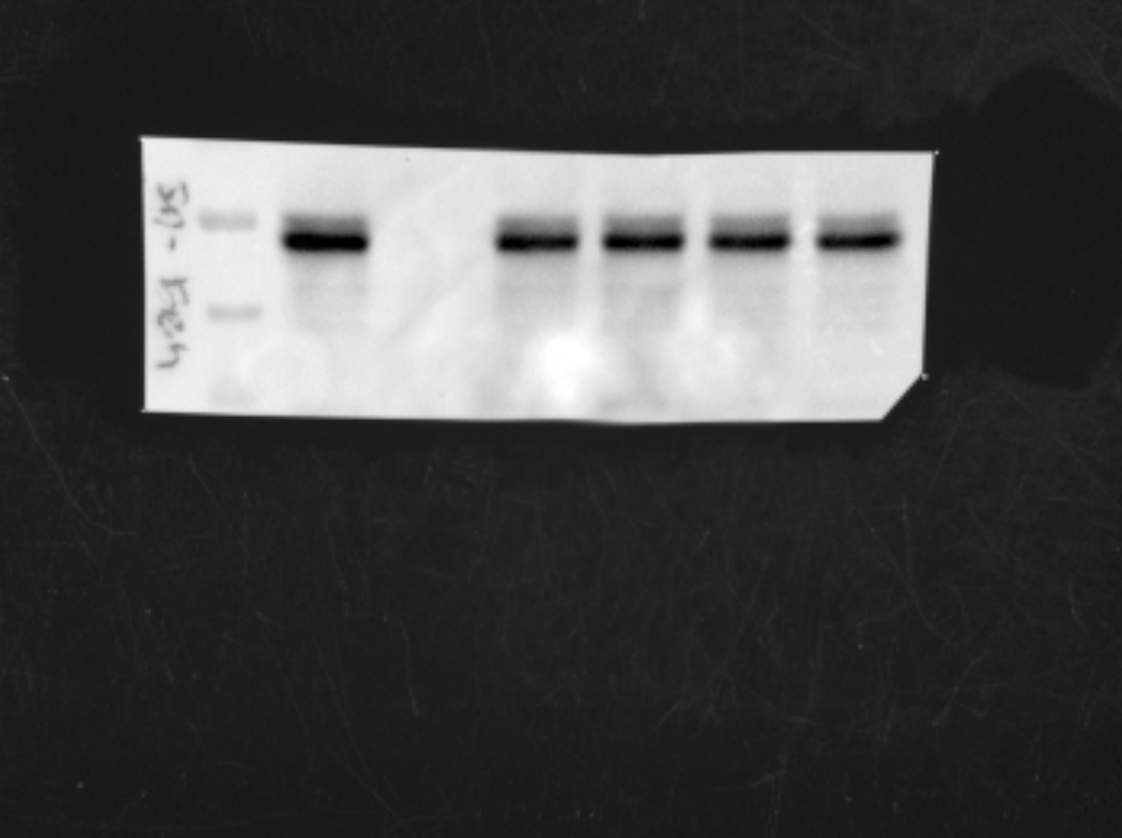

Supplement: Supplementary file 4 — Source data Fig. 3 [file 44321_2026_423_MOESM4_ESM.zip › Figure 3/3F/WB IN ERG.tif]

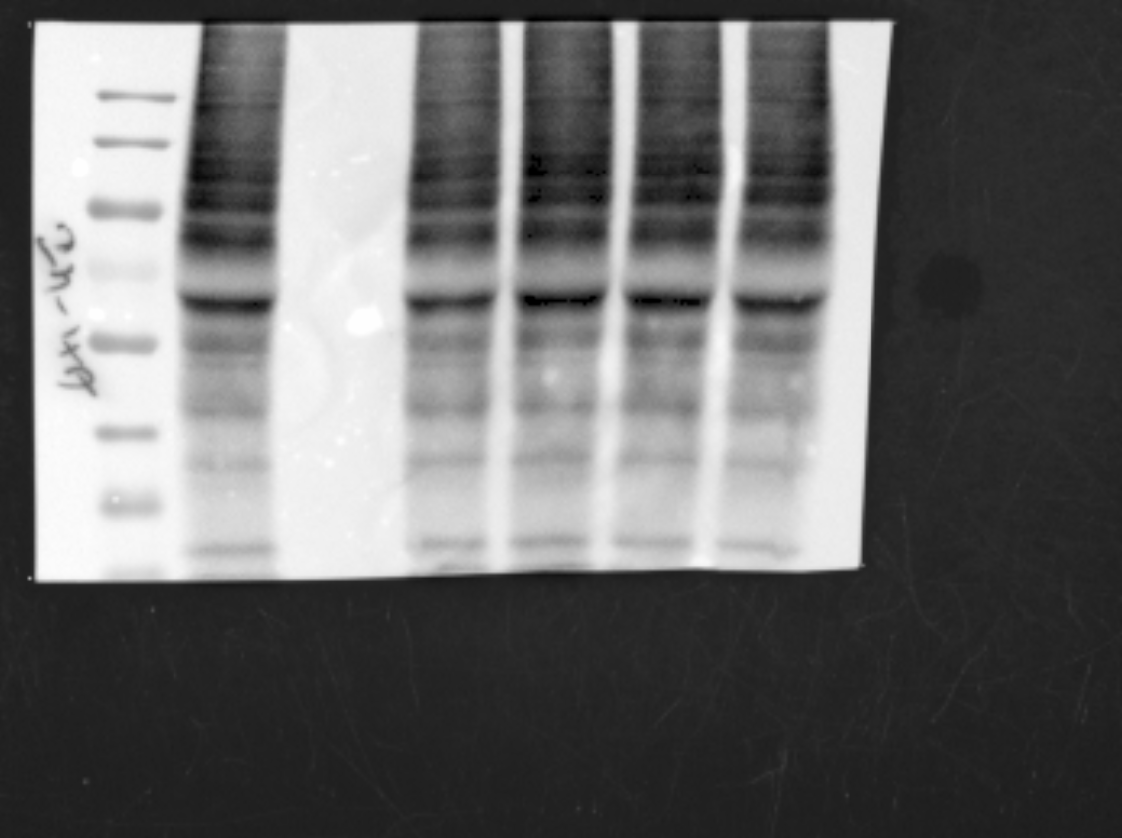

Supplement: Supplementary file 4 — Source data Fig. 3 [file 44321_2026_423_MOESM4_ESM.zip › Figure 3/3F/WB IN HA.tif]

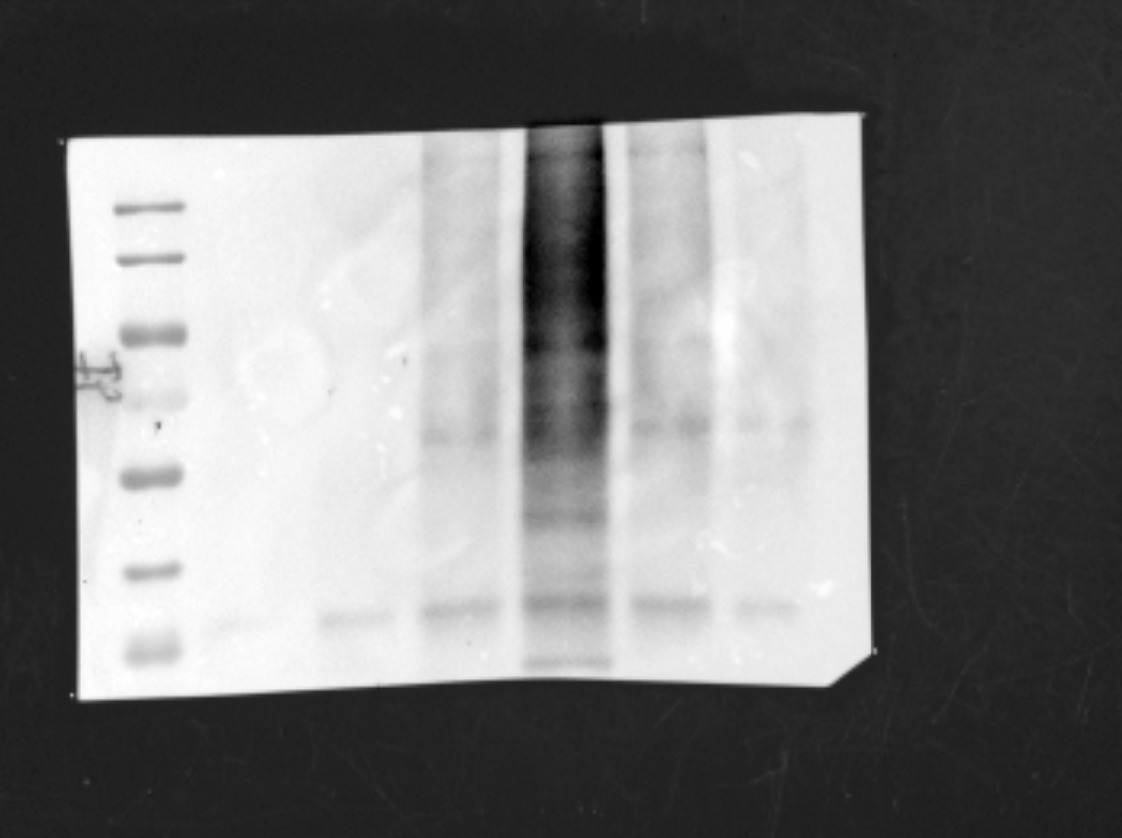

Supplement: Supplementary file 4 — Source data Fig. 3 [file 44321_2026_423_MOESM4_ESM.zip › Figure 3/3F/WB IP ERG IB HA.tif]

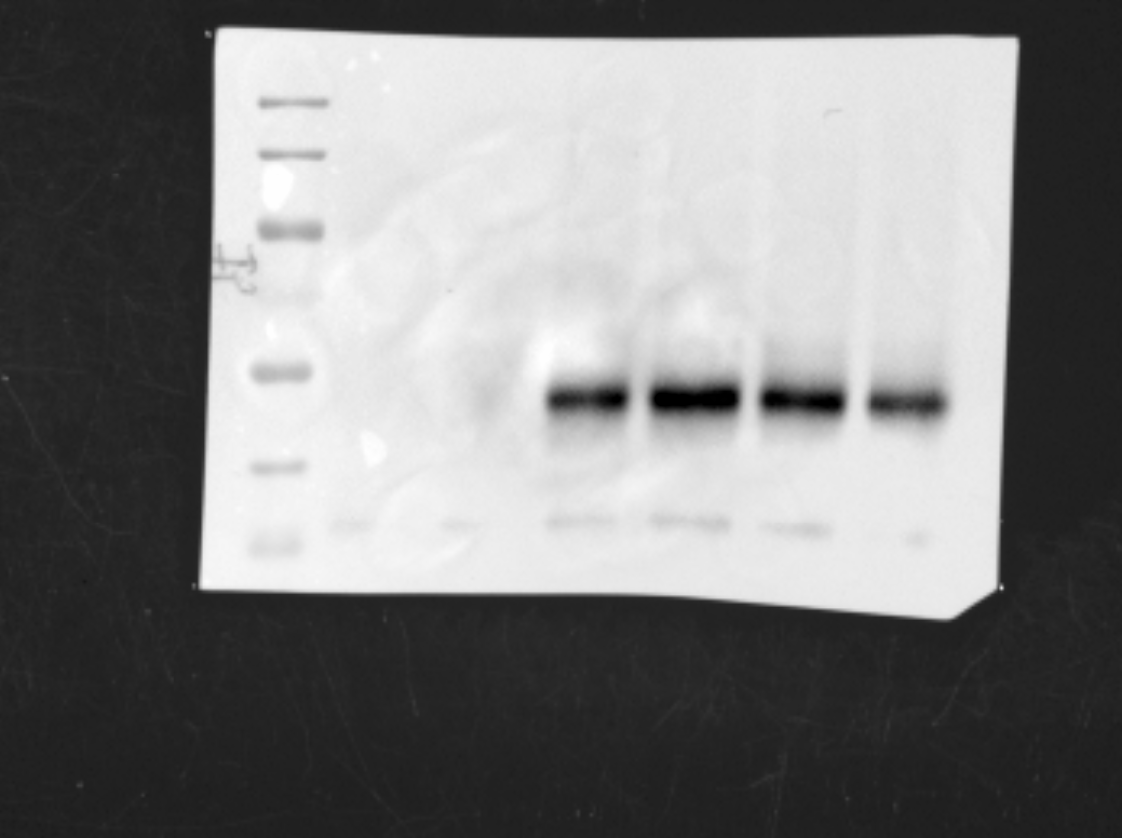

Supplement: Supplementary file 4 — Source data Fig. 3 [file 44321_2026_423_MOESM4_ESM.zip › Figure 3/3F/WB IP ERG.tif]

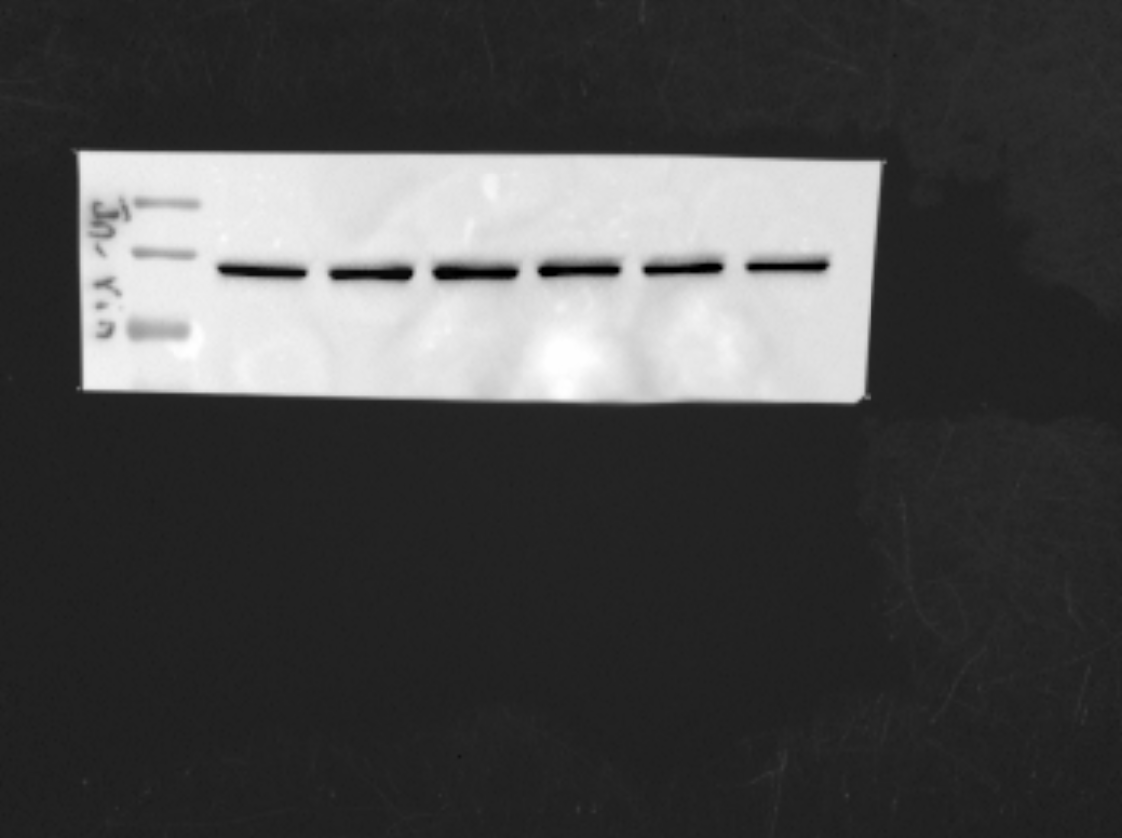

Supplement: Supplementary file 4 — Source data Fig. 3 [file 44321_2026_423_MOESM4_ESM.zip › Figure 3/3F/WB VIN.tif]

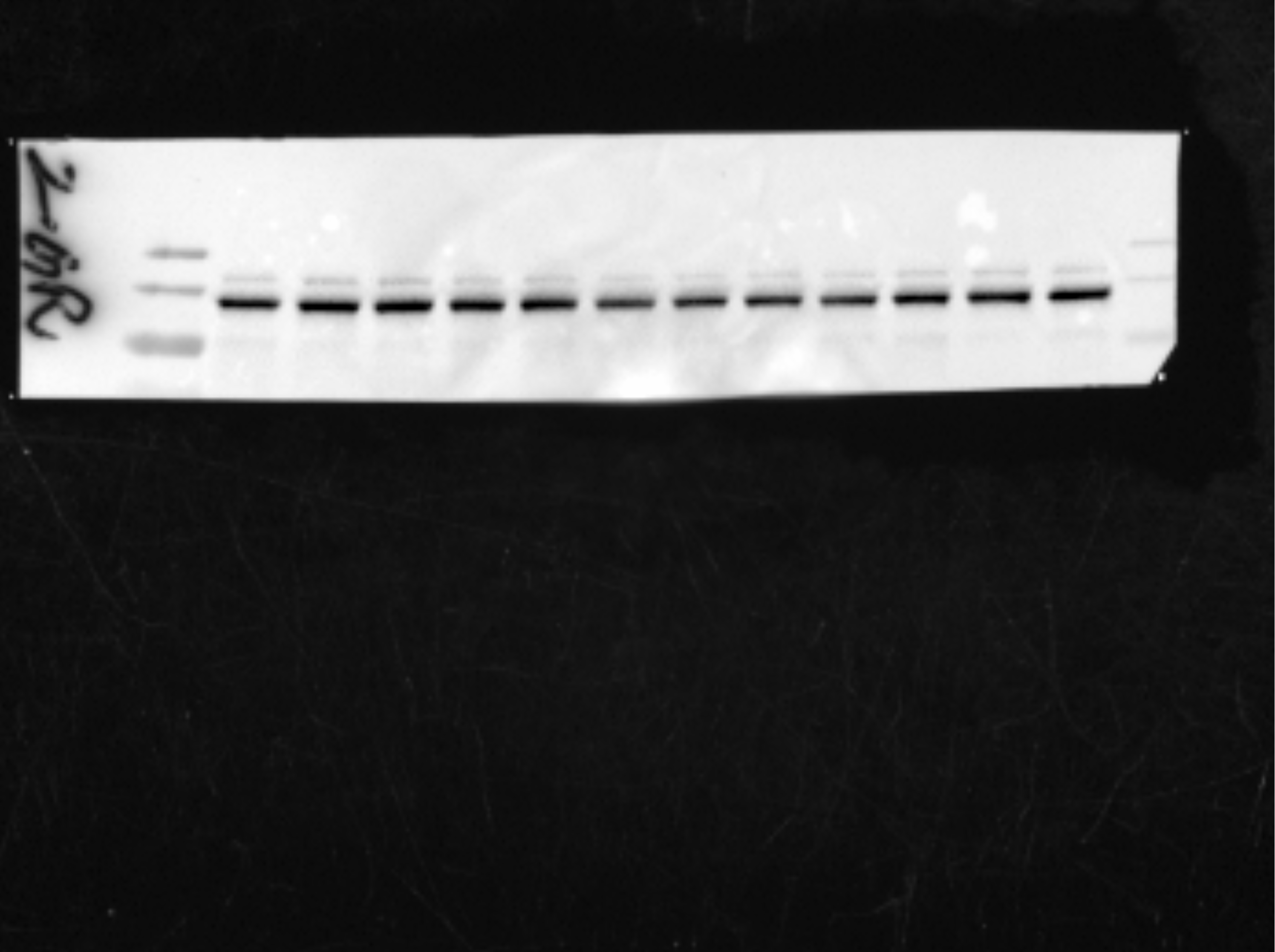

Supplement: Supplementary file 4 — Source data Fig. 3 [file 44321_2026_423_MOESM4_ESM.zip › Figure 3/3G/vin+L.tif]

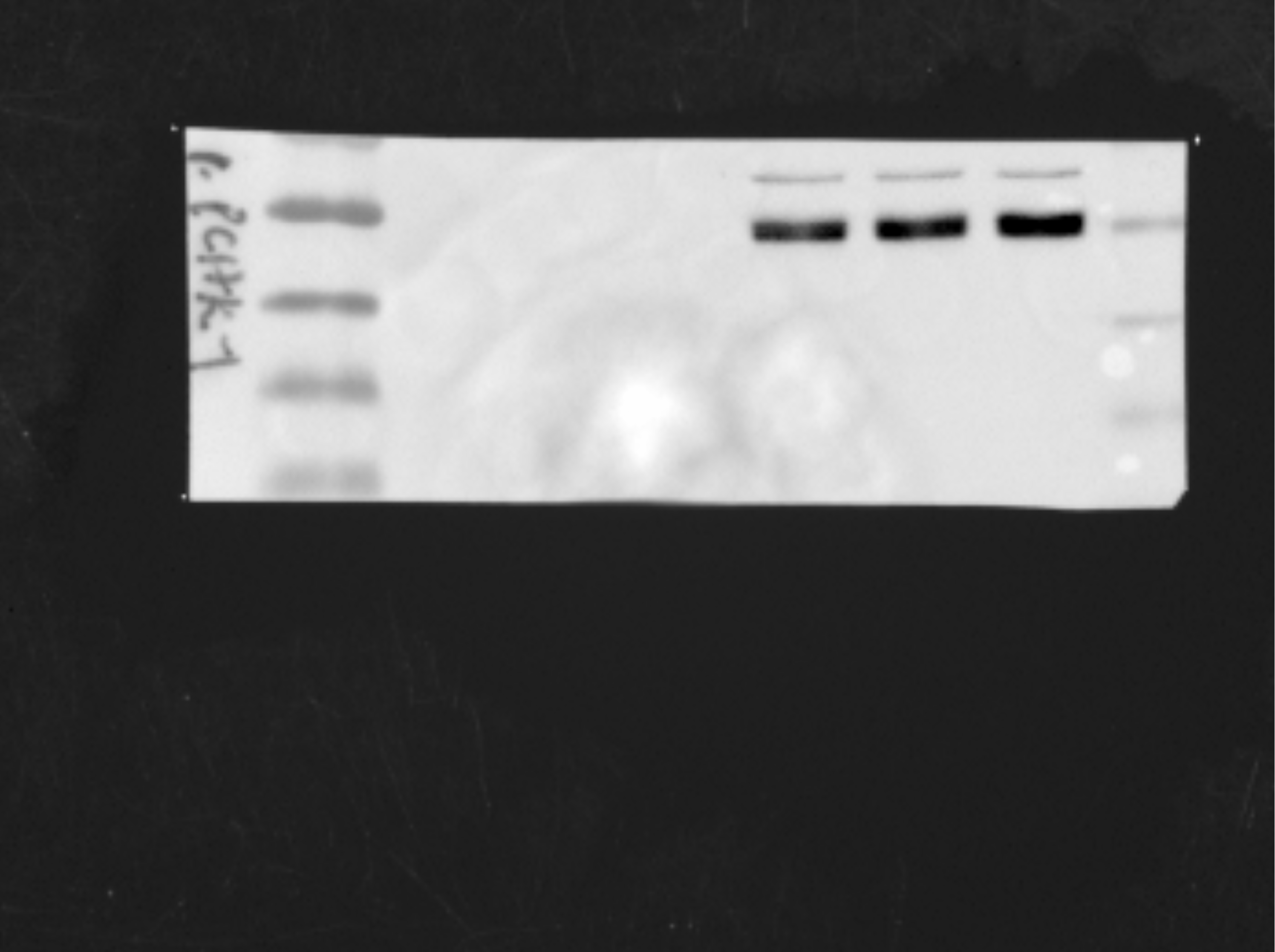

Supplement: Supplementary file 4 — Source data Fig. 3 [file 44321_2026_423_MOESM4_ESM.zip › Figure 3/3G/pCHK1+L.tif]

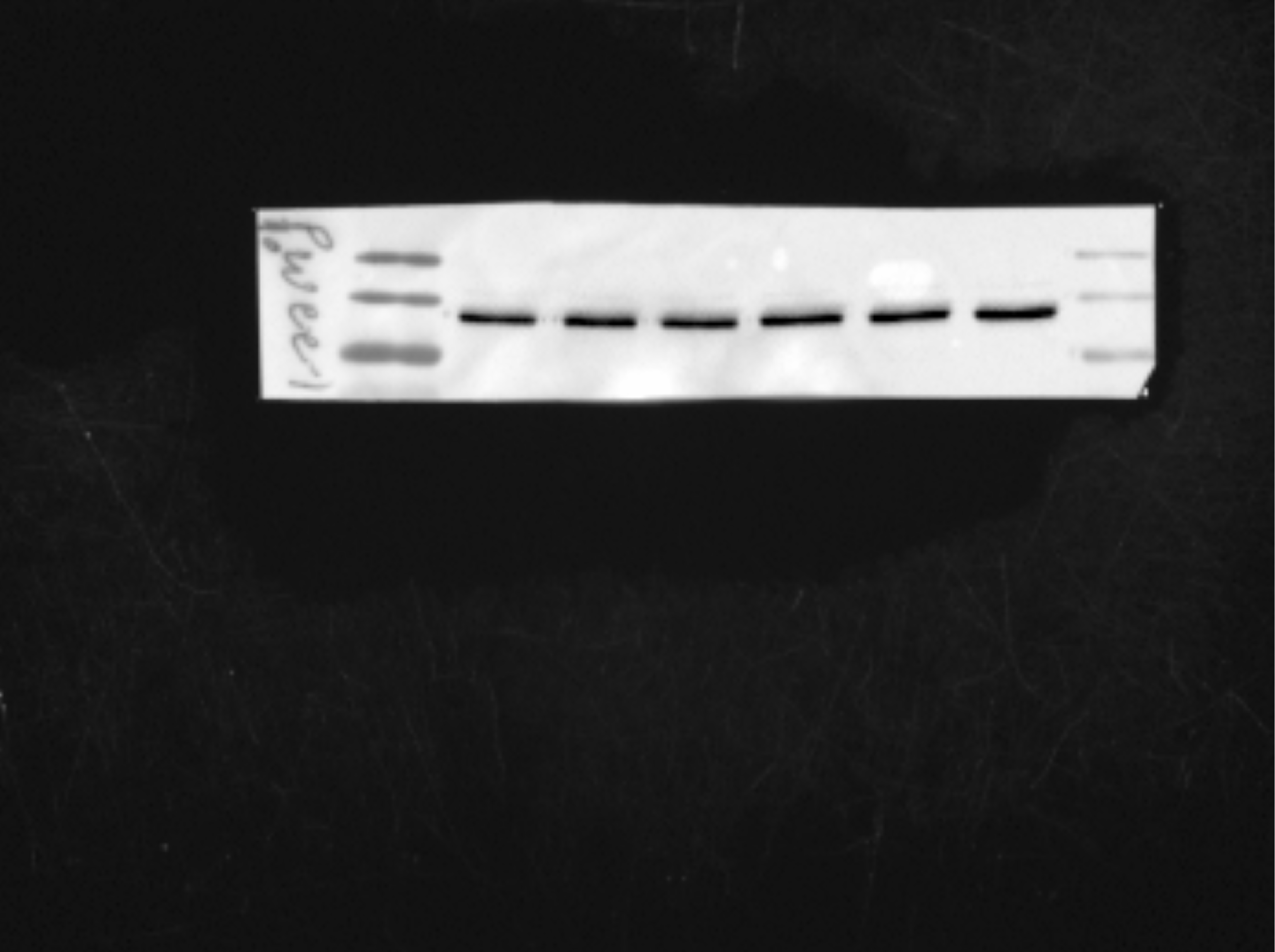

Supplement: Supplementary file 4 — Source data Fig. 3 [file 44321_2026_423_MOESM4_ESM.zip › Figure 3/3G/vin 10%+L.tif]

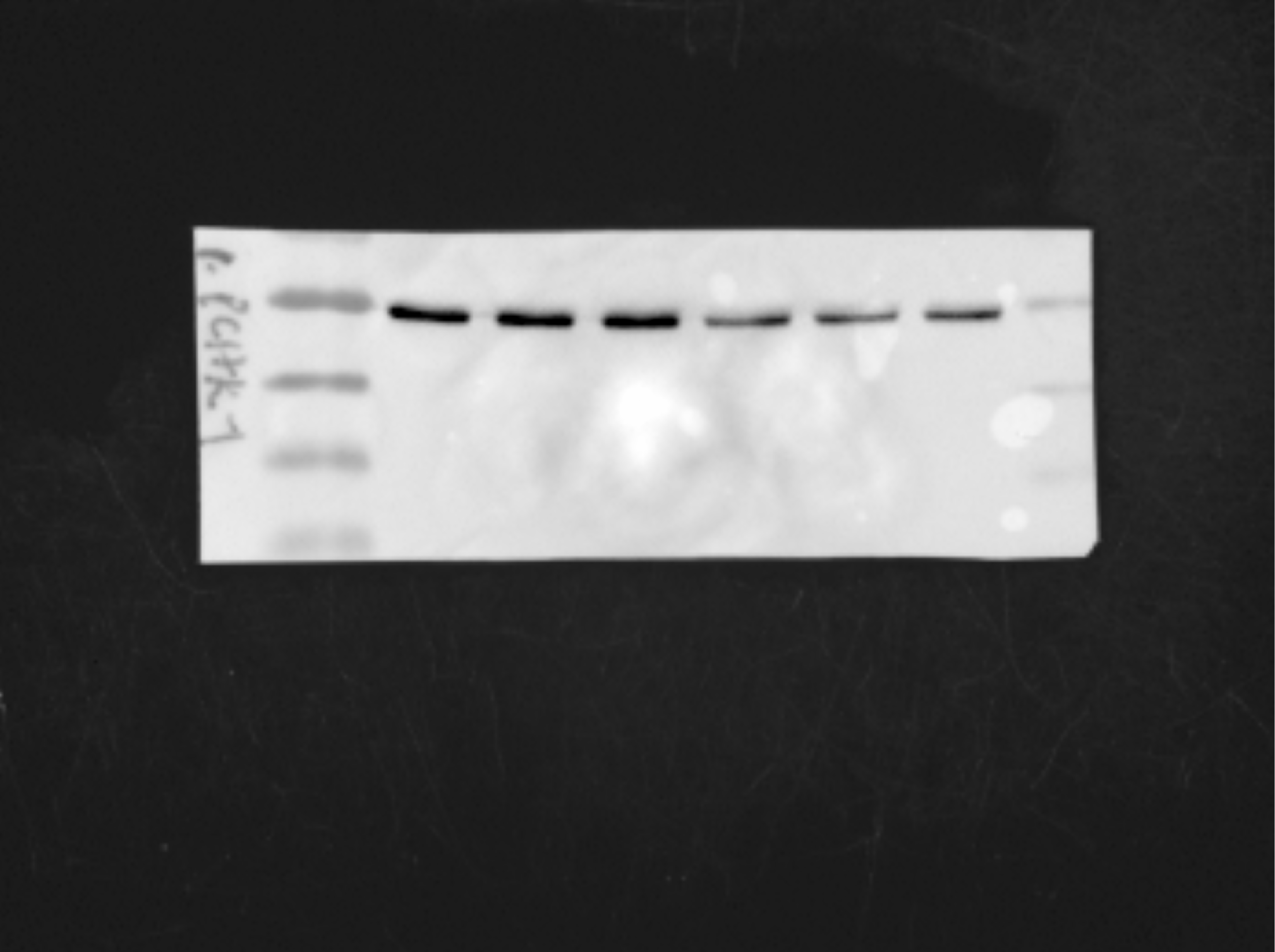

Supplement: Supplementary file 4 — Source data Fig. 3 [file 44321_2026_423_MOESM4_ESM.zip › Figure 3/3G/CHK1+L.tif]

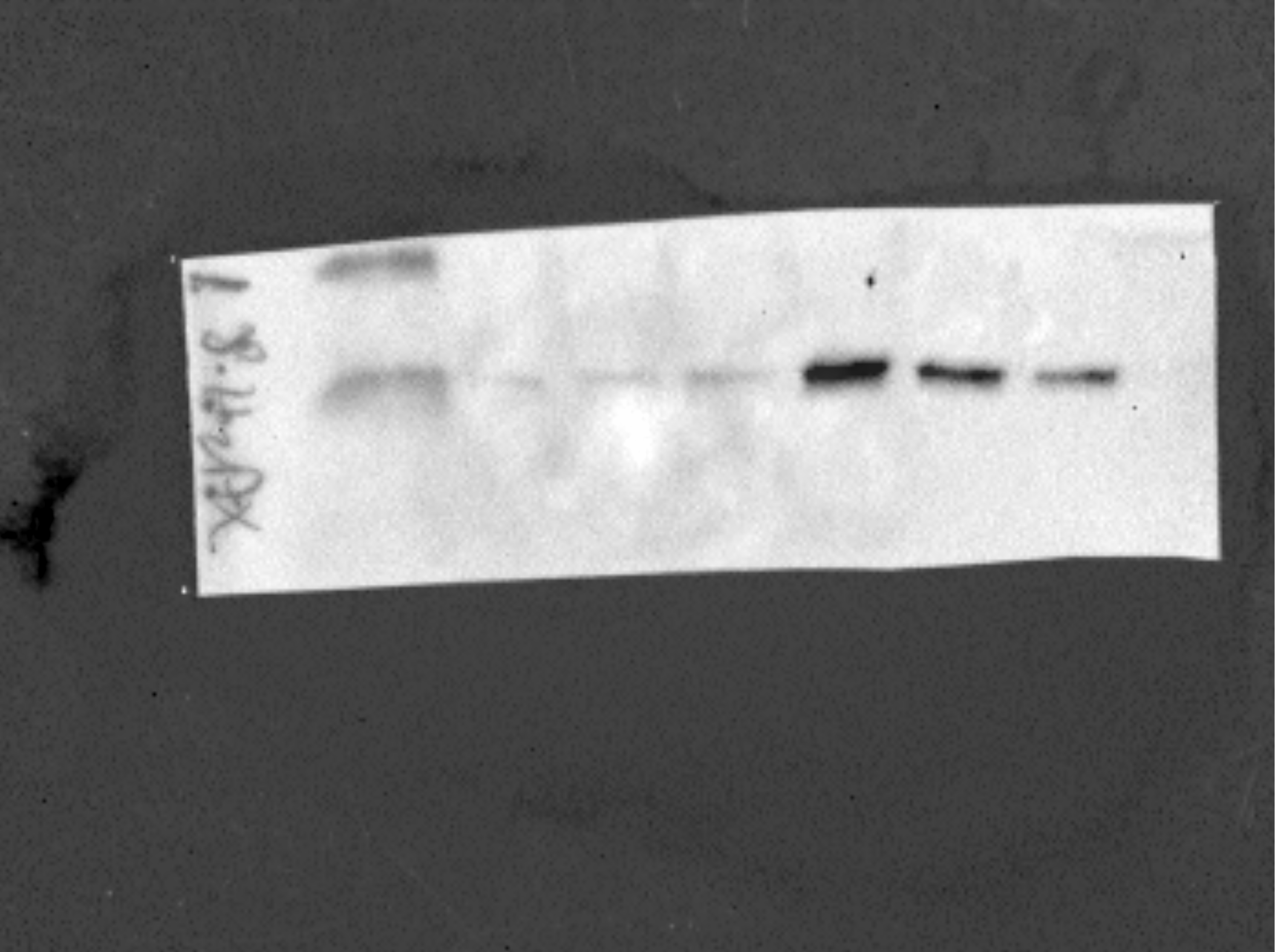

Supplement: Supplementary file 4 — Source data Fig. 3 [file 44321_2026_423_MOESM4_ESM.zip › Figure 3/3G/pH2AX+L.tif]

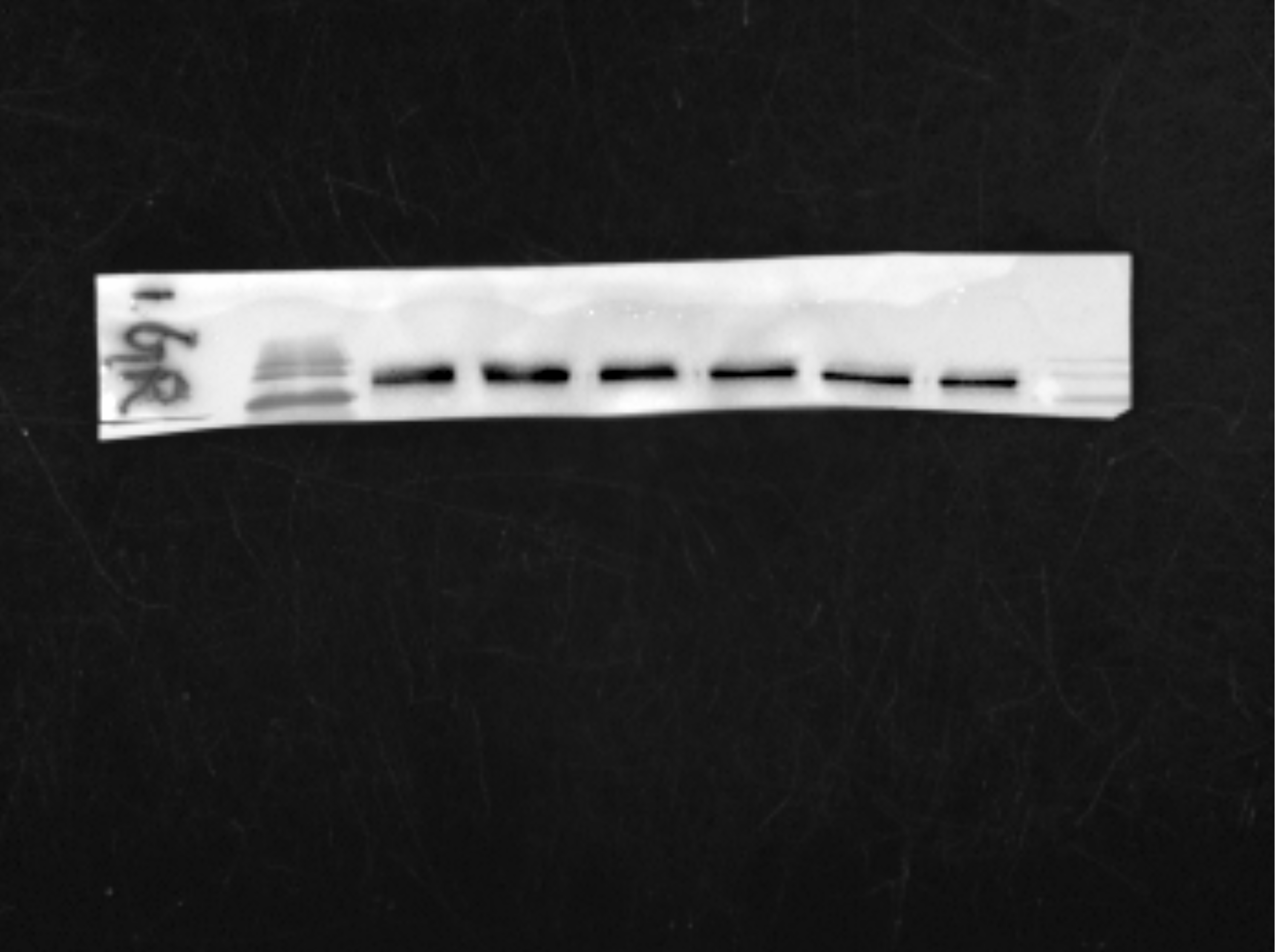

Supplement: Supplementary file 4 — Source data Fig. 3 [file 44321_2026_423_MOESM4_ESM.zip › Figure 3/3G/vin 15%+L.tif]

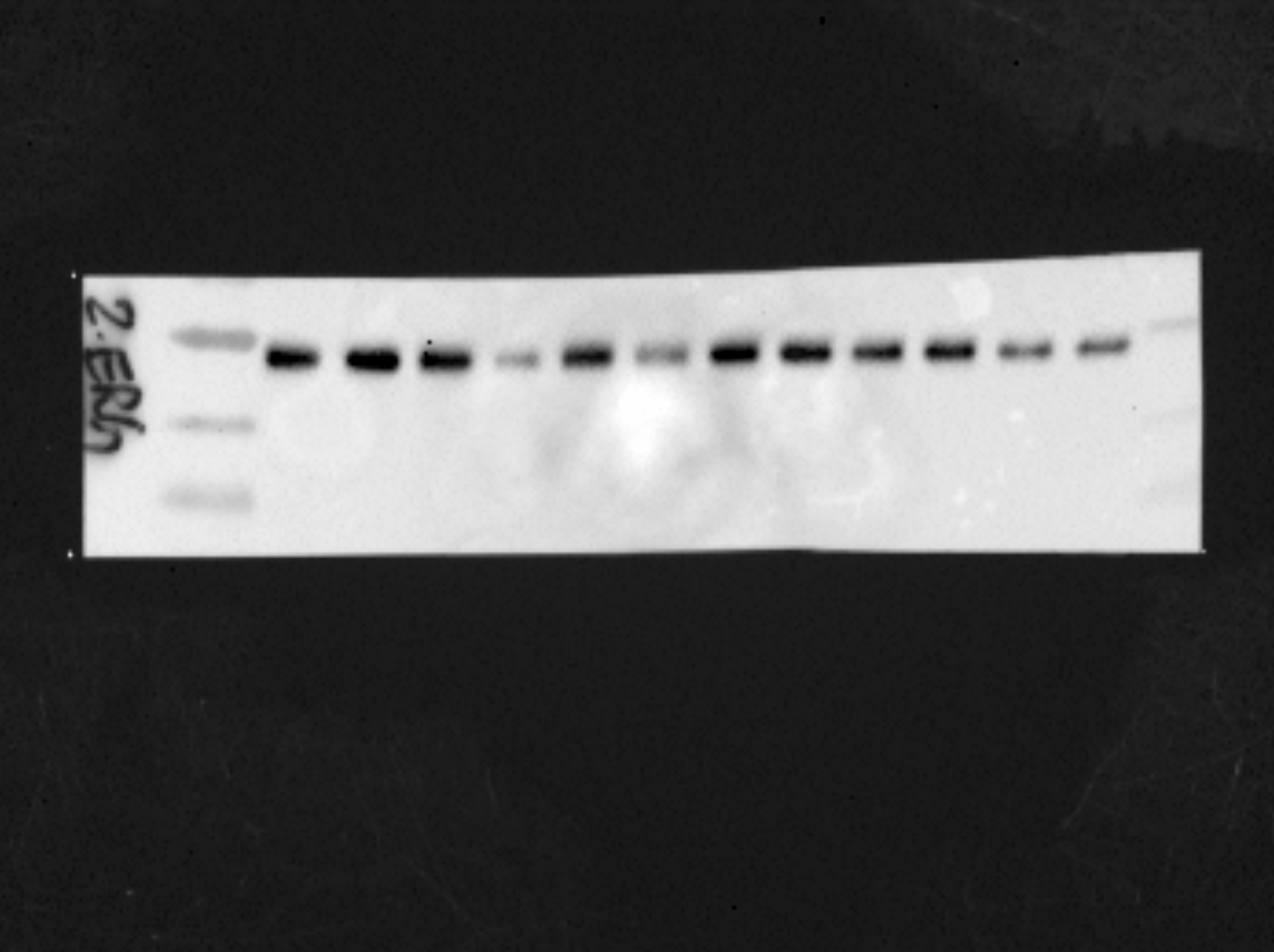

Supplement: Supplementary file 4 — Source data Fig. 3 [file 44321_2026_423_MOESM4_ESM.zip › Figure 3/3G/ERG+L.tif]

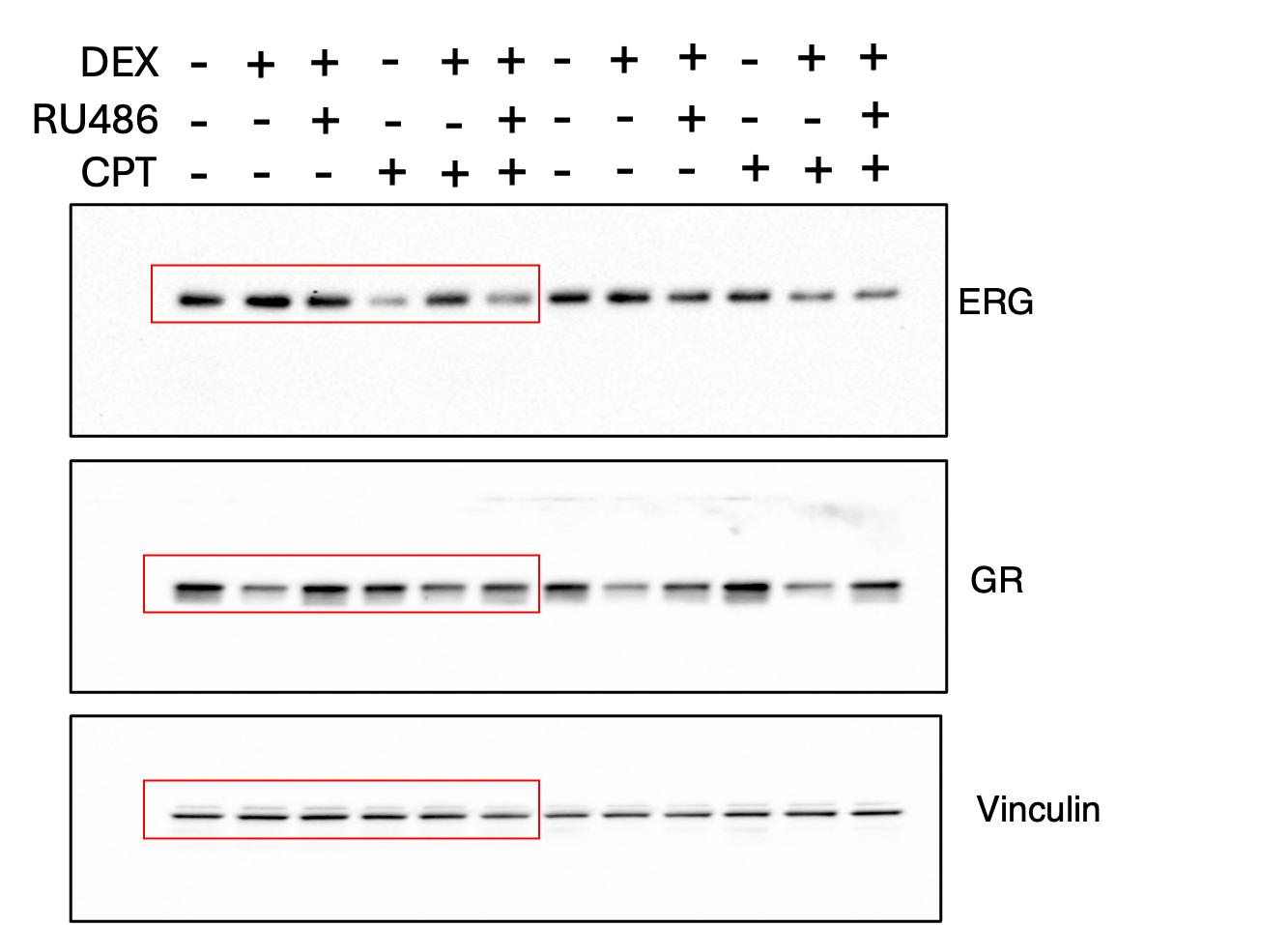

Supplement: Supplementary file 4 — Source data Fig. 3 [file 44321_2026_423_MOESM4_ESM.zip › Figure 3/3G/3g.png]

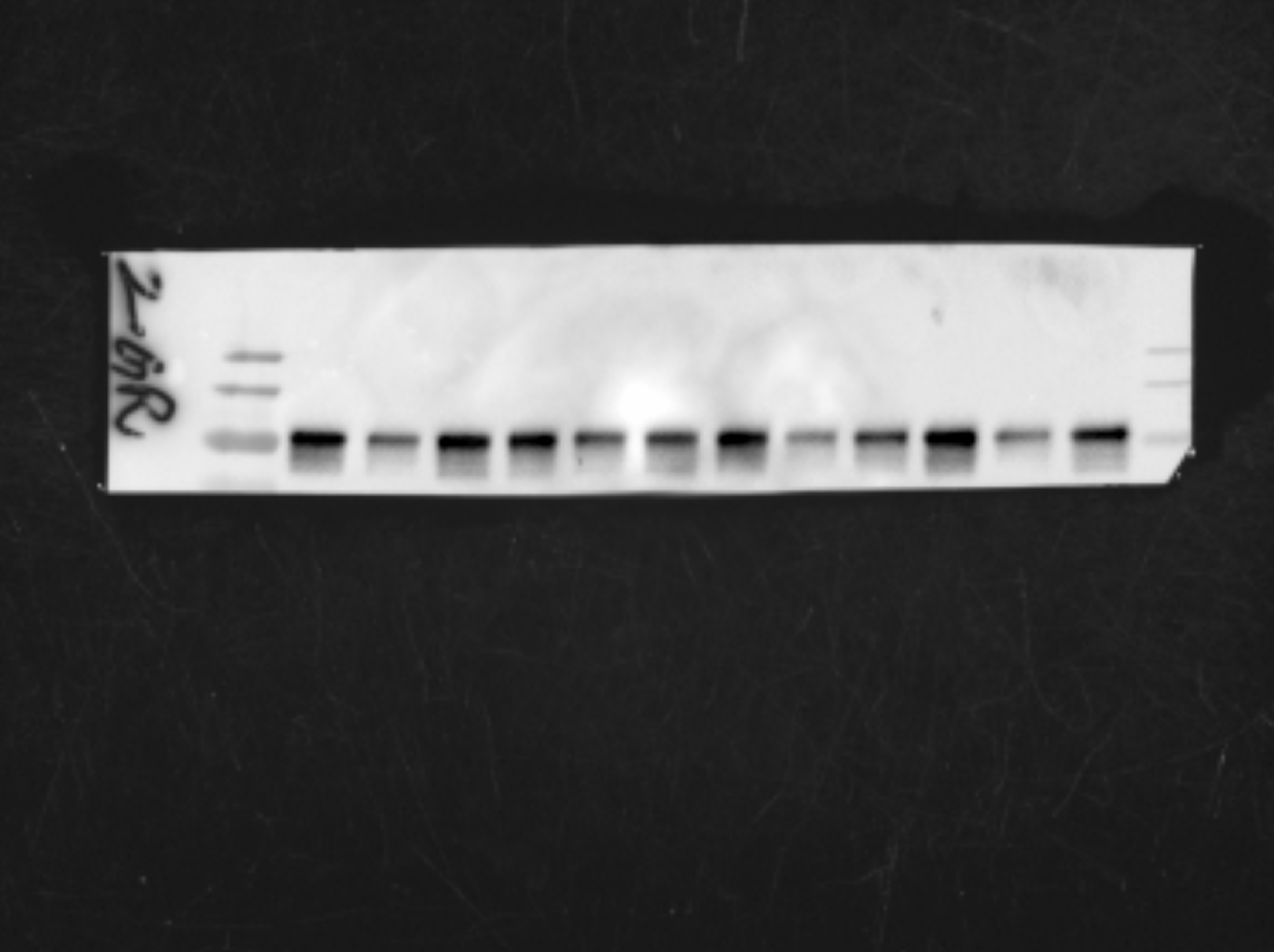

Supplement: Supplementary file 4 — Source data Fig. 3 [file 44321_2026_423_MOESM4_ESM.zip › Figure 3/3G/GR+L.tif]

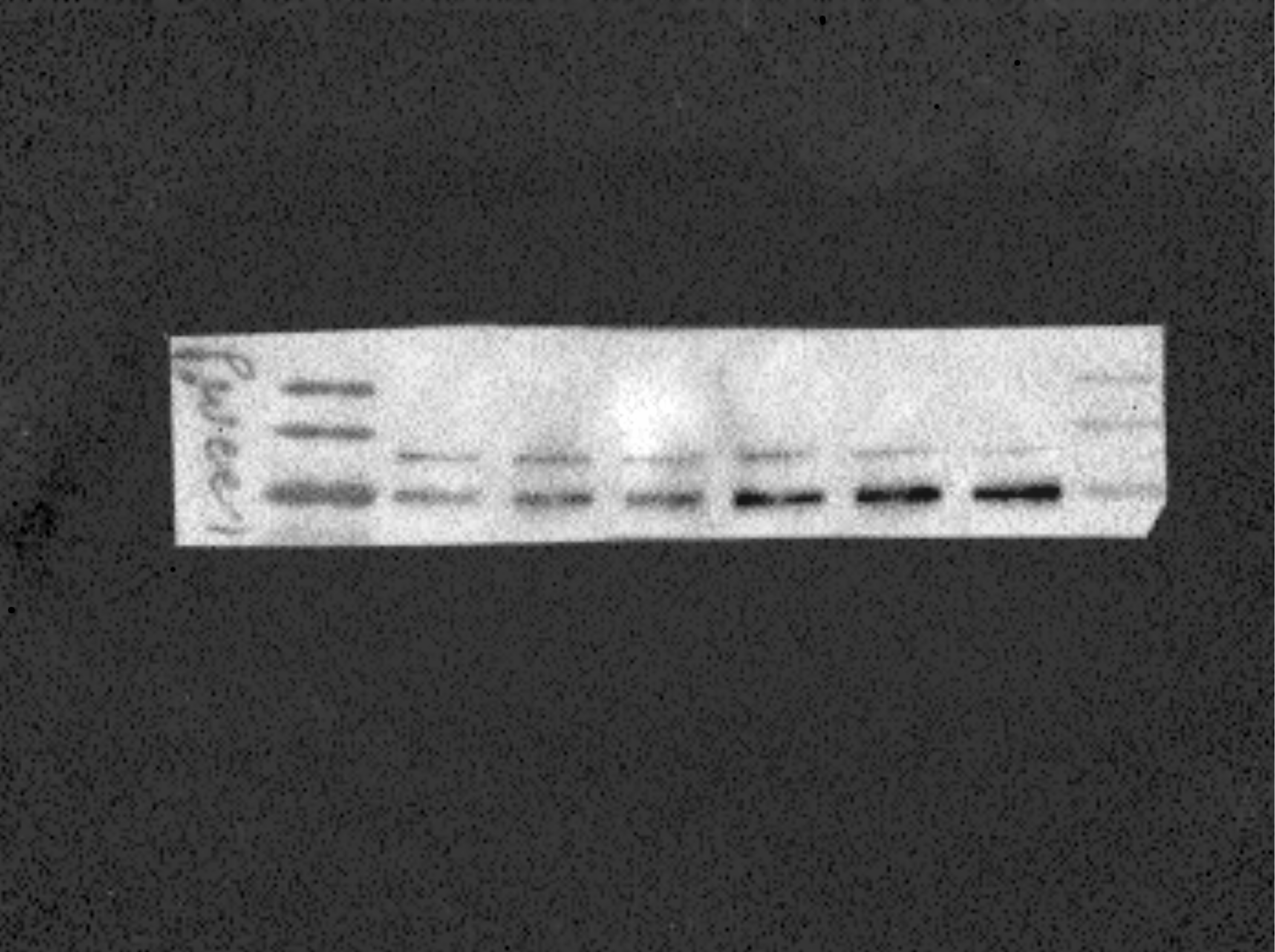

Supplement: Supplementary file 4 — Source data Fig. 3 [file 44321_2026_423_MOESM4_ESM.zip › Figure 3/3G/pWee1+L.tif]

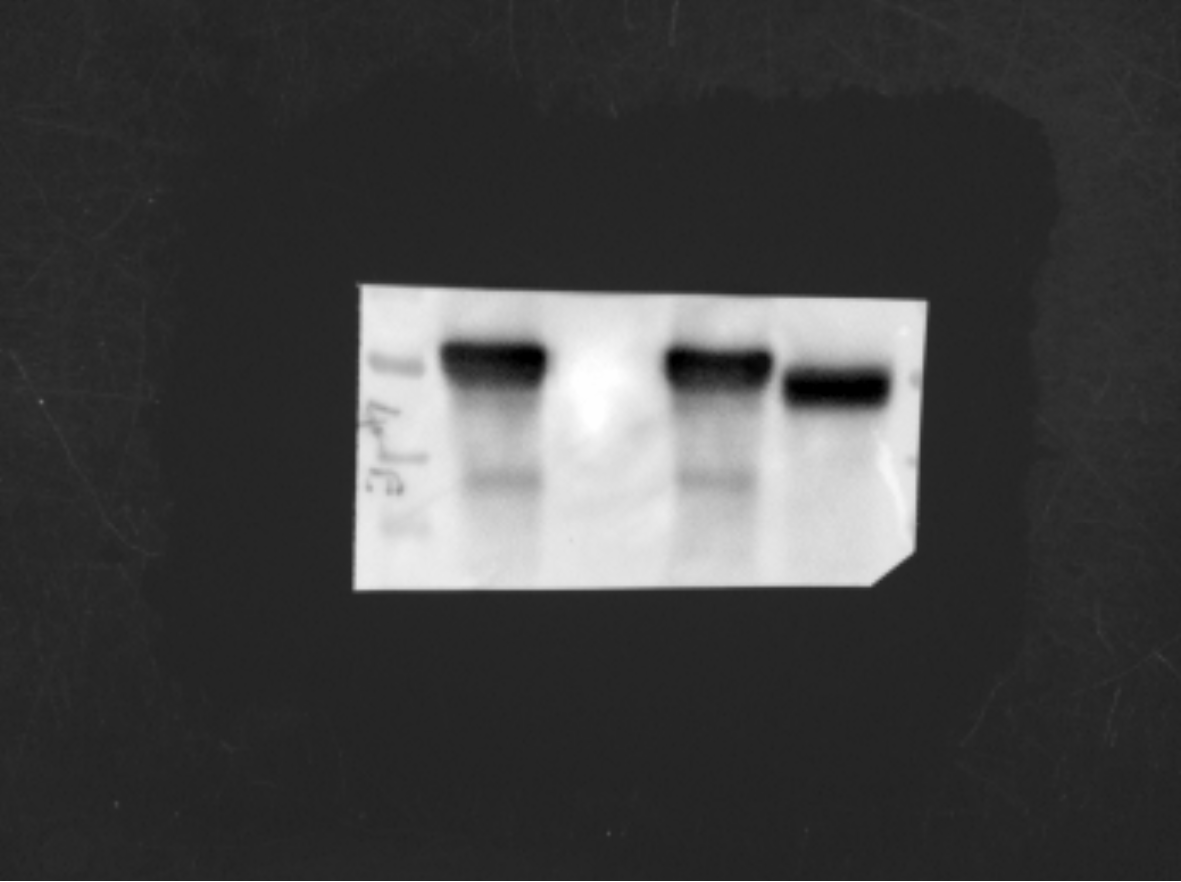

Supplement: Supplementary file 5 — Source data Fig. 4 [file 44321_2026_423_MOESM5_ESM.zip › Figure 4/4F/WB IN HA.tif]

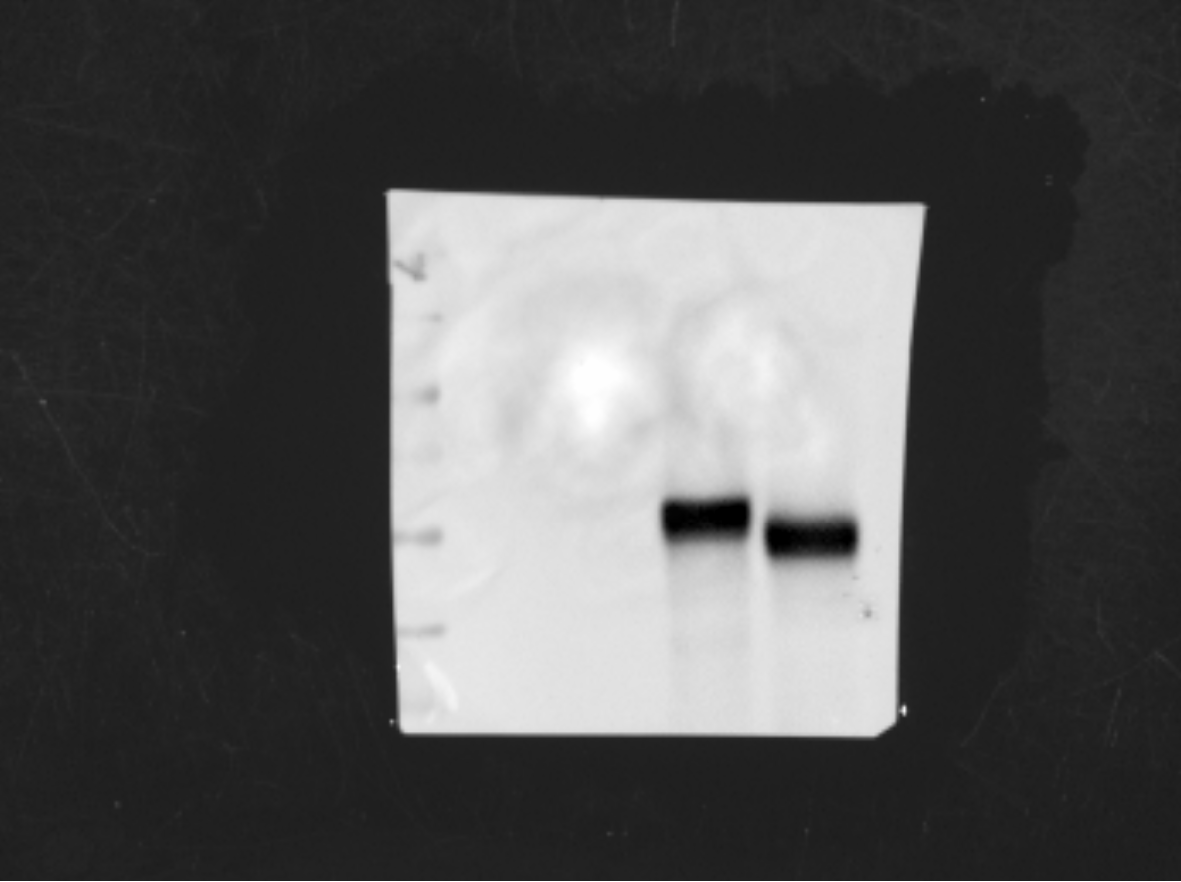

Supplement: Supplementary file 5 — Source data Fig. 4 [file 44321_2026_423_MOESM5_ESM.zip › Figure 4/4F/WB IP HA IB HA.tif]

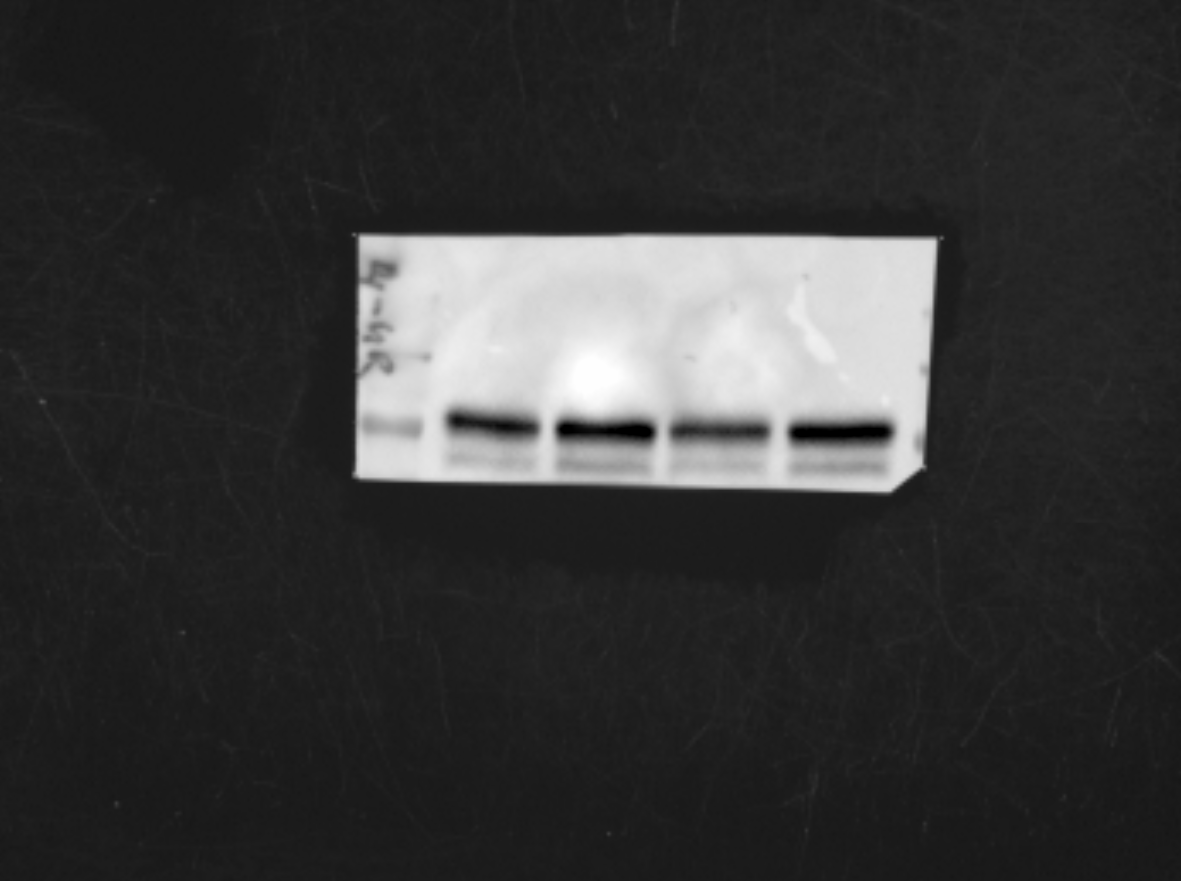

Supplement: Supplementary file 5 — Source data Fig. 4 [file 44321_2026_423_MOESM5_ESM.zip › Figure 4/4F/WB IN GR.tif]

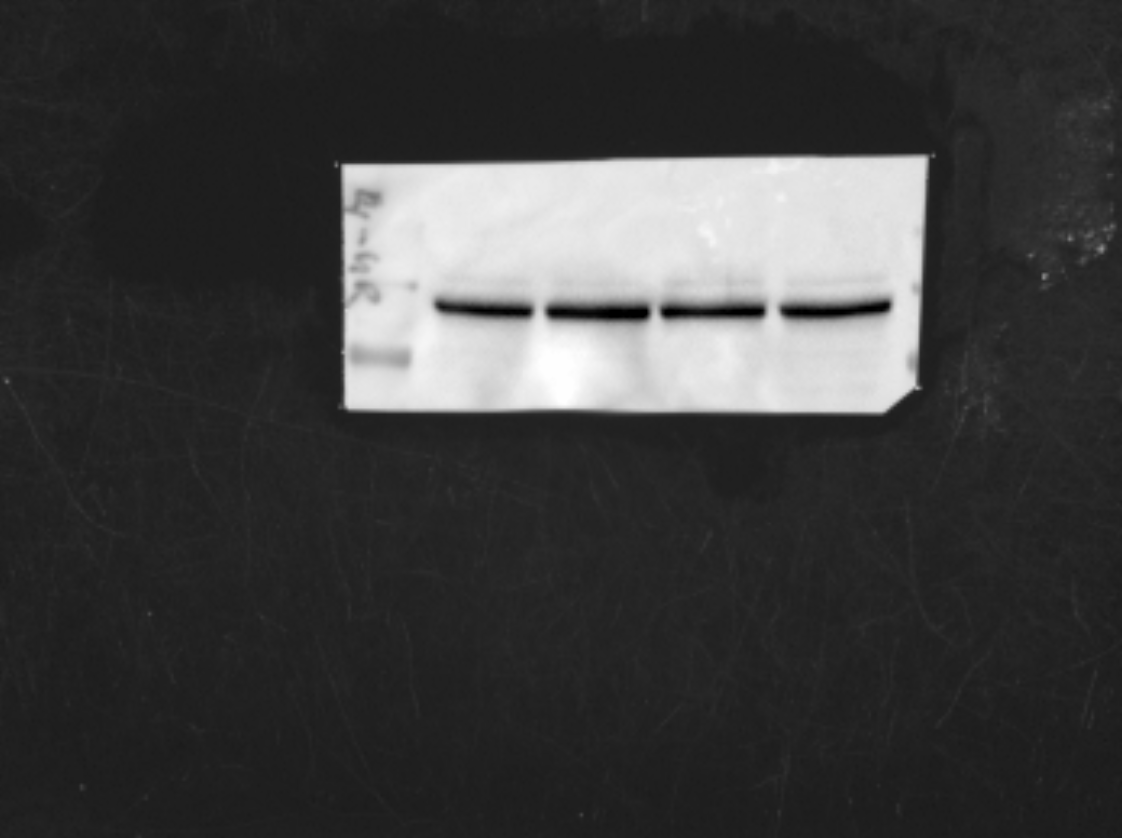

Supplement: Supplementary file 5 — Source data Fig. 4 [file 44321_2026_423_MOESM5_ESM.zip › Figure 4/4F/WB IN VINCULIN.tif]

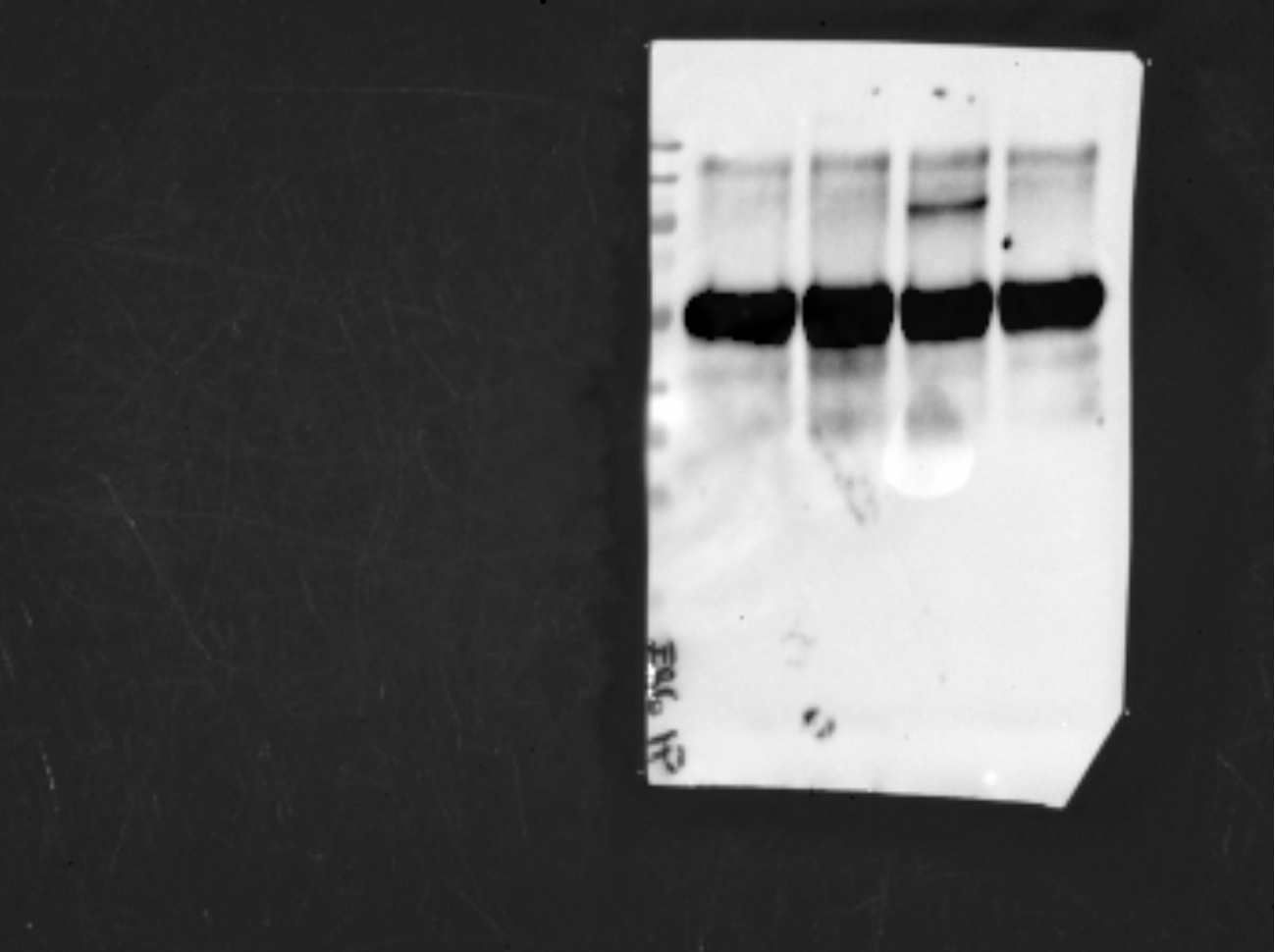

Supplement: Supplementary file 5 — Source data Fig. 4 [file 44321_2026_423_MOESM5_ESM.zip › Figure 4/4F/WB IP ERG IB GR.tif]
