# Supplementary material for: TMPRSS2-ERG confers resistance of prostate cancer to antiandrogens
Source: EMBO Mol Med. 2026 May 12;18(6):2062–97. doi: 10.1038/s44321-026-00423-7 (PMC13269939; doi:10.1038/s44321-026-00423-7)
Supplement: Supplementary file 9 — Expanded View Figures [file 44321_2026_423_MOESM9_ESM.pdf]

# Expanded View Figures

**Figure EV1. Immunohistochemical and computational analyses pre- and post-treatment with ADT plus enzalutamide indicate that tERG positivity and high GR (post-treatment) can predict resistance to antiandrogens (related to Fig. 1).**

(**A**) Targeted biopsies were obtained from 37 men with intermediate to high-risk PCa before receiving ADT plus enzalutamide for 6 months. Shown are representative micrographs of IHC staining with anti-ERG and anti-GR antibodies applied to serial sections of matched baseline (left) and posttreatment (right) prostate tumor tissues from a TMPRSS2-ERG fusion-positive (top) and from a fusion-negative (bottom) case. Bar, 100 µm. (**B**) ERG mRNA levels in responders and nonresponders before treatment. Statistical analysis was performed using paired *t* test (or Wilcoxon signed-rank test). *$P < 0.05$; **$P < 0.01$; ***$P < 0.001$ and ****$P < 0.0001$. In boxplots, the central line represents the median, the box bounds represent the 25th and 75th percentiles, and the whiskers extend to the minimum and maximum values. (**C**) GR protein expression assessed by IHC scoring in matched pre- and post-treatment samples from the responder cohort. Paired comparisons between pre- and post-treatment samples were performed using the Wilcoxon signed-rank test. (**D**) Shown are Spearman rank correlations between ERG and NR3C1 expression in 19 patients (from GSE102124) post-treatment with abiraterone plus ADT. (**E**) Spearman rank correlation between ERG and GR (NR3C1) expression post-ADT treatment, based on analysis of prostate RNA-seq data from 160 patients (WCDT dataset). (**F**) ERG and GR are favored to be mutually co-existing. Thin tissue sections were prepared from sixty-two patients who underwent standard-of-care prostatectomies with no hormonal therapy. Immunohistochemistry analysis was performed using anti-GR and anti-ERG antibodies. (upper panel) The table presents results obtained after performing a one-sided Fisher's exact test that examined whether the co-occurrence of GR and ERG was favored. Listed are the numbers of ETS-positive and ETS-negative tumors, along with the status of GR expression. (lower panel) Shown are example IHC analyses from a representative ERG-positive tumor. Exact *P* values, statistical tests, sample sizes, and error bar definitions for all panels are provided in Appendix Table S3.

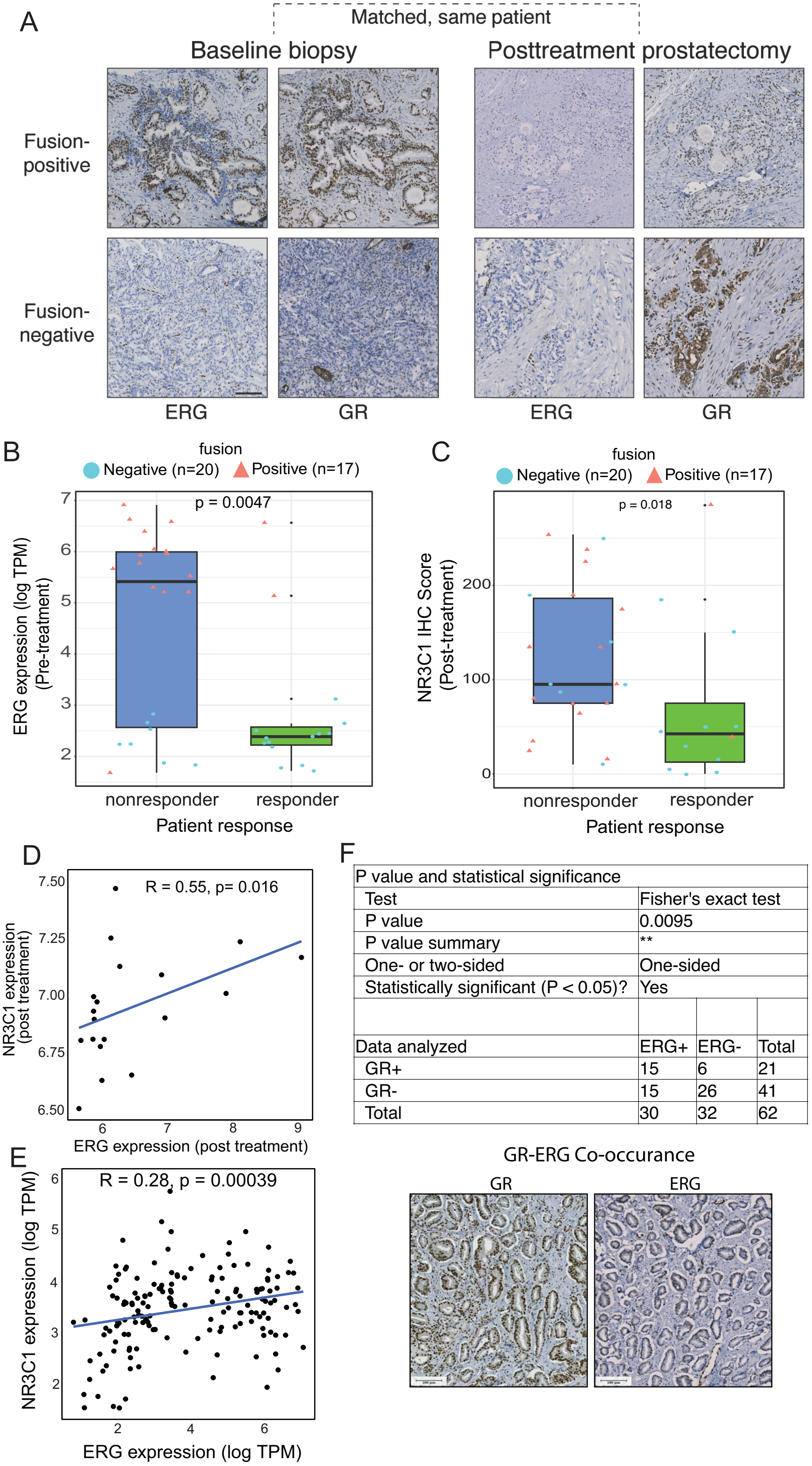
A
Matched, same patient
Baseline biopsy
Posttreatment prostatectomy
Fusion-positive
Fusion-negative
ERG
GR
ERG
GR
B
fusion
Negative (n=20)
Positive (n=17)
p = 0.0047
ERG expression (log TPM) (Pre-treatment)
nonresponder
responder
Patient response
C
fusion
Negative (n=20)
Positive (n=17)
p = 0.018
NR3C1 IHC Score (Post-treatment)
nonresponder
responder
Patient response
D
R = 0.55, p= 0.016
NR3C1 expression (post treatment)
ERG expression (post treatment)
F
P value and statistical significance
Test | Fisher's exact test
P value | 0.0095
P value summary | **
One- or two-sided | One-sided
Statistically significant (P < 0.05)? | Yes
Data analyzed | ERG+ | ERG- | Total
GR+ | 15 | 6 | 21
GR- | 15 | 26 | 41
Total | 30 | 32 | 62
E
R = 0.28, p = 0.00039
NR3C1 expression (log TPM)
ERG expression (log TPM)
GR-ERG Co-occurance
GR
ERG


© The Author(s)

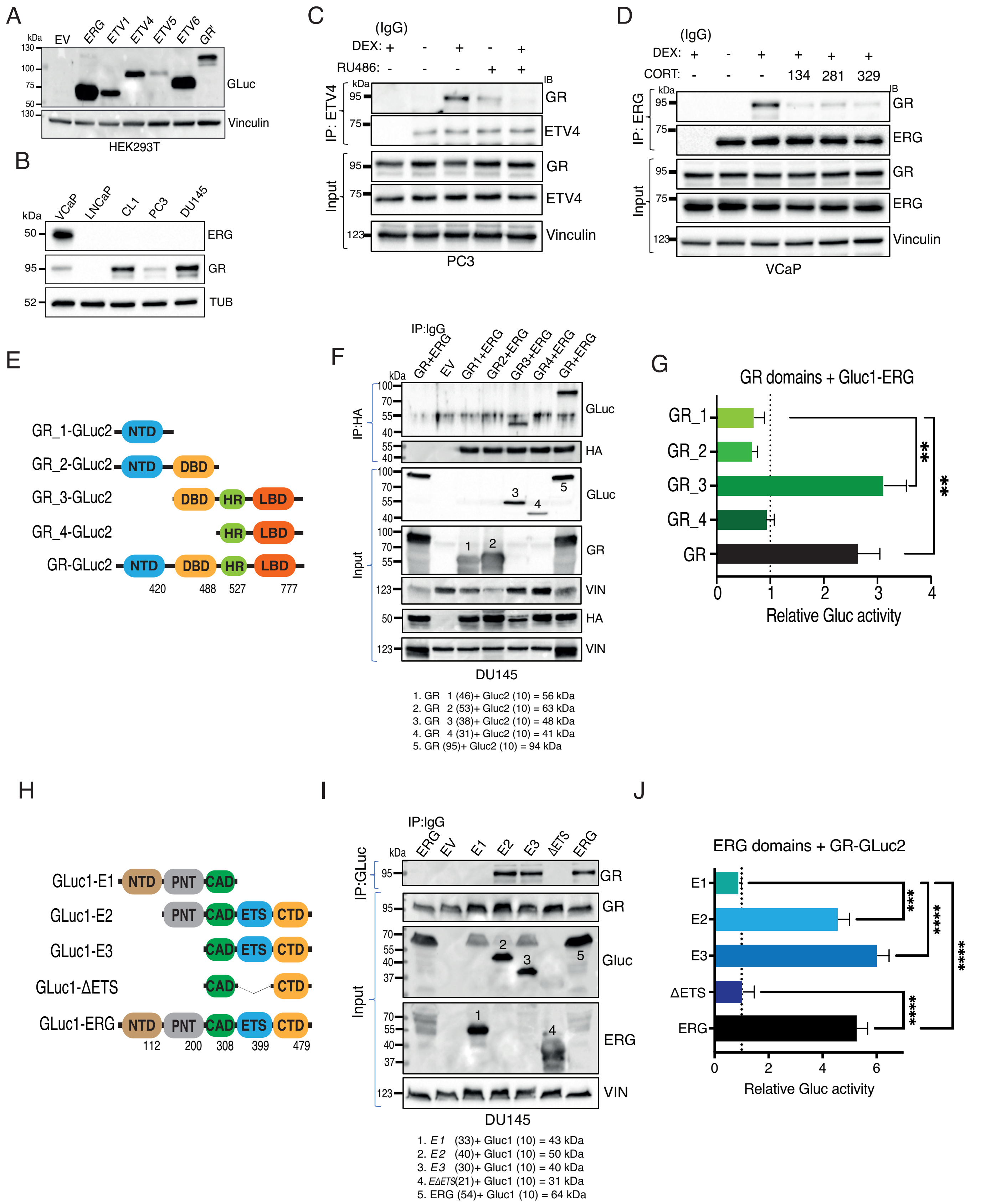
A
EV ERG ETV1 ETV4 ETV5 ETV6 GR'
kDa 130 100 75 50 130
GLuc
Vinculin
HEK293T
B
VCaP LNCaP CL1 PC3 DU145
kDa 50 95 52
ERG
GR
TUB
C
(IgG)
DEX: + - + - +
RU486: - - - + +
IP: ETV4
Input
kDa 95 75 95 75 123
IB
GR
ETV4
GR
ETV4
Vinculin
PC3
D
(IgG)
DEX: + - + + + +
CORT: - - - 134 281 329
IP: ERG
Input
kDa 95 75 95 75 123
IB
GR
ERG
GR
ERG
Vinculin
VCaP
E
GR_1-GLuc2 NTD
GR_2-GLuc2 NTD DBD
GR_3-GLuc2 DBD HR LBD
GR_4-GLuc2 HR LBD
GR-GLuc2 NTD DBD HR LBD
420 488 527 777
F
IP:IgG
GR+ERG EV GR1+ERG GR2+ERG GR3+ERG GR4+ERG GR+ERG
IP:HA
Input
kDa 100 70 55 40 55 40 100 70 55 40 100 70 55 123 50 123
GLuc
HA
GLuc
GR
VIN
HA
VIN
1 2 3 4 5
DU145
1. GR 1 (46)+ Gluc2 (10) = 56 kDa
2. GR 2 (53)+ Gluc2 (10) = 63 kDa
3. GR 3 (38)+ Gluc2 (10) = 48 kDa
4. GR 4 (31)+ Gluc2 (10) = 41 kDa
5. GR (95)+ Gluc2 (10) = 94 kDa
G
GR domains + Gluc1-ERG
GR_1
GR_2
GR_3
GR_4
GR
**
**
0 1 2 3 4
Relative Gluc activity
H
GLuc1-E1 NTD PNT CAD
GLuc1-E2 PNT CAD ETS CTD
GLuc1-E3 CAD ETS CTD
GLuc1-ΔETS CAD CTD
GLuc1-ERG NTD PNT CAD ETS CTD
112 200 308 399 479
I
IP:IgG
ERG EV E1 E2 E3 ΔETS ERG
IP:GLuc
Input
kDa 95 95 70 55 40 37 70 55 40 37 123
GR
GR
Gluc
ERG
VIN
1 2 3 4 5
DU145
1. E1 (33)+ Gluc1 (10) = 43 kDa
2. E2 (40)+ Gluc1 (10) = 50 kDa
3. E3 (30)+ Gluc1 (10) = 40 kDa
4. EΔETS(21)+ Gluc1 (10) = 31 kDa
5. ERG (54)+ Gluc1 (10) = 64 kDa
J
ERG domains + GR-GLuc2
E1
E2
E3
ΔETS
ERG
***
****
****
****
0 2 4 6
Relative Gluc activity

**Figure EV2. The DBD-HR-LBD region of GR interacts with the ETS domain of ERG (related to Fig. 2).**

(**A**) An immunoblot showing the levels of the Gluc fusion proteins we transiently expressed in HEK293T cells ($5x10^5$). Cells were harvested 24 h post-transfection and processed for immunoblotting. Vinculin was used as the gel loading control. (**B**) Comparative analysis of ERG and GR levels in the PCa cell lines we employed. Whole extracts of the indicated cell lines were prepared and subjected to immunoblotting for ERG and GR. Tubulin (TUB) was used to ensure equal gel loading. (**C**) Serum-starved PC3 cells (naturally overexpressing ETV4) were treated for 60 min with vehicle, DEX (1 µM), RU486 (1 µM), or the combination of drugs. Extracts were processed for co-immunoprecipitation (IP) and immunoblotting (IB). IgG, control antibody. (**D**) Serum-starved VCaP cells were treated for 60 min with vehicle (CON) or DEX (1 µM), in the absence or presence of the indicated SGRM compounds (C134, C281, or C329; 10 µM each). Whole-cell extracts were processed for co-immunoprecipitation of ERG and GR, followed by immunoblotting. (**E**, **F**) A schematic representation of the various domains of GR fused to Gluc2, along with their levels of expression in HEK293T cells. The indicated domains were inserted N-terminally to Gluc2. NTD N-terminal domain, DBD DNA-binding domain, HR hinge region, LBD ligand binding domain. DU145 cells were transfected with Gluc1-ERG plasmids encoding different deletion mutants of ERG (see molecular weight calculations at the bottom of the panel). The Gluc1-ERG proteins were immunoprecipitated using an antibody specific to HA. Immunoblotting was performed using antibodies that detected the endogenous forms of GR. (**G**) HEK293T cells ($6 \times 10^3$) were co-transfected with Gluc2 plasmids encoding different domains of GR and a Gluc1 plasmid encoding ERG (full length). Following 24 h of incubation, cells were starved overnight and then treated for 60 min with vehicle, or with DEX (1 µM). The normalized luminescence activity of each construct is presented. Data are presented as mean ± SEM. Statistical analysis was performed using two-way ANOVA with the Tukey's multiple comparisons test. The experiment was repeated thrice with biological triplicates. Error bars represent the means ± SEM. (**H**, **I**) A schematic representation of the various domains of ERG fused to Gluc1, along with their levels of expression in transfected DU145 cells. The indicated domains were inserted C-terminally to Gluc1. NTD N-terminal domain, Pointed domain, CAD central activation domain, ETS DNA-binding domain, CTD C-terminal domain. See cloning primer sequences in Appendix Table S1. (**J**) The Gluc2 plasmid encoding full-length GR was co-transfected in DU145 cells together with the indicated Gluc1 plasmids encoding different domains of ERG. Following 24 h of incubation, cells were starved overnight and then treated for 60 min with vehicle or DEX (1 µM). Luminescence was determined in biological triplicate. All experiments were repeated three times. Data are presented as mean ± SEM. Statistical analysis was performed using two-way ANOVA with Tukey's multiple comparisons test. The experiment was repeated twice with biological triplicates. Error bars represent mean ± SEM. $^{**}P < 0.01$; $^{***}P < 0.001$; $^{****}P < 0.0001$. Note: the quantitative analyses (**F**, **I**) were repeated at least twice. Exact *P* values, statistical tests, sample sizes, and error bar definitions for all panels are provided in Appendix Table S3.


© The Author(s)

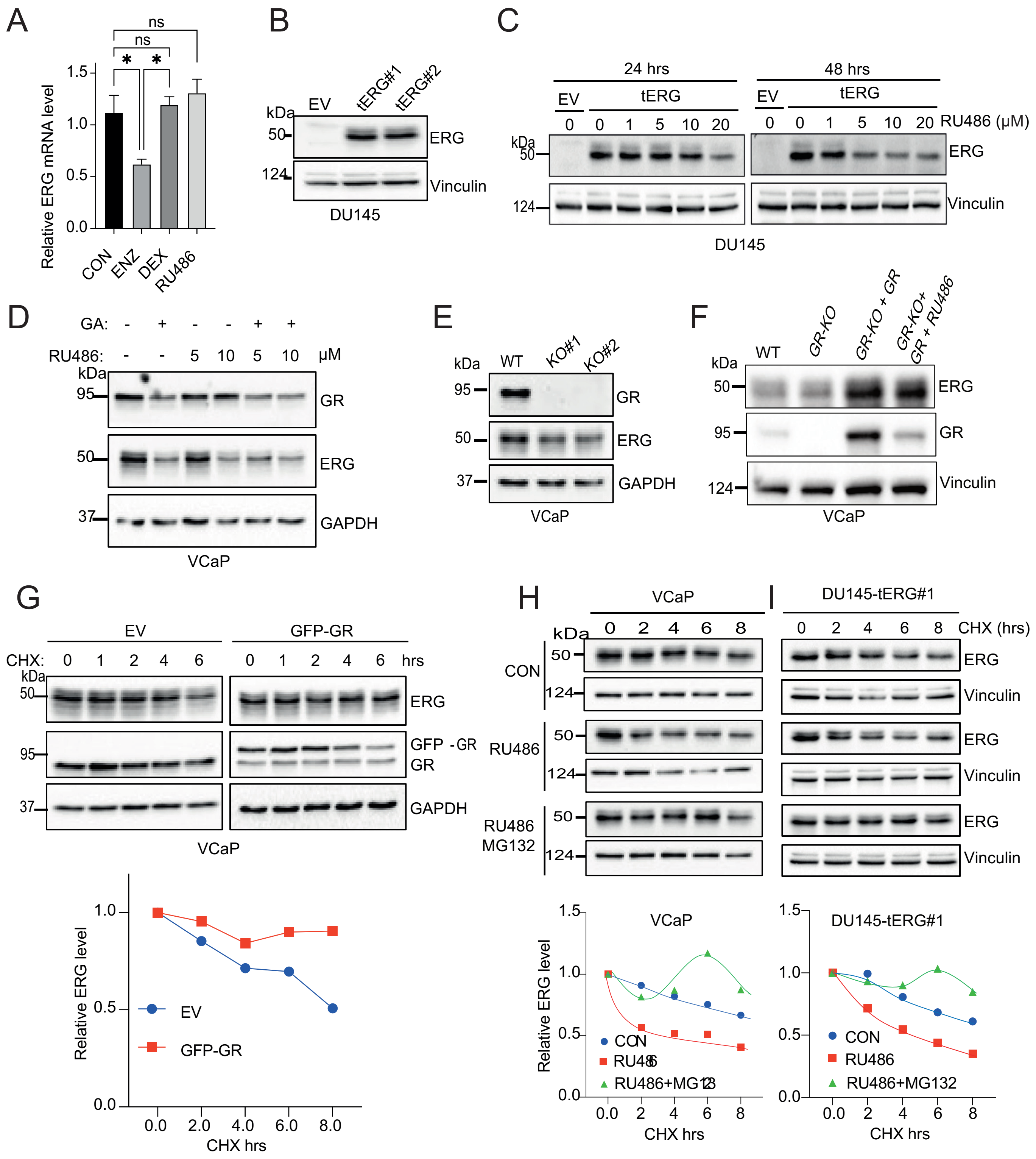
A
B
C
Relative ERG mRNA level
ns
ns
CON
ENZ
DEX
RU486
EV
tERG#1
tERG#2
ERG
Vinculin
DU145
24 hrs
48 hrs
tERG
RU486 (μM)
D
GA:
RU486:
μM
GR
ERG
GAPDH
VCaP
E
WT
KO#1
KO#2
F
GR-KO
GR-KO + GR
GR-KO+ GR + RU486
G
GFP-GR
CHX:
hrs
GFP -GR
H
I
DU145-tERG#1
CHX (hrs)
CON
RU486
RU486 MG132
Relative ERG level
CHX hrs
RU486+MG132

**Figure EV3. Inhibition of GR destabilizes ERG (related to Fig. 3).**

(**A**) VCaP cells were treated with vehicle, DEX, ENZ, or RU486 (10 μM, each) for 48 h and later subjected to RT-qPCR that determined ERG transcript levels. Statistical analysis was performed using one-way ANOVA with Dunnett's multiple comparisons test. The experiment was performed twice with $n = 2$ biological replicates. (**B**) DU145 cells were transfected with EV or with tERG-encoding plasmids and selected with blasticidin for 10 days. The indicated positive clones were used as the stable tERG-overexpressing cells. (**C**) DU145 cells stably overexpressing tERG were treated for 24 or 48 h with increasing concentrations of RU486. Thereafter, whole cell extracts were prepared and subjected to immunoblotting, as indicated. (**D**) VCaP cells were treated with geldanamycin (GA; 1 μg/ml), RU486 (either 5 or 10 μg/ml) or with the combination of drugs for 48 h. Whole-cell extracts were prepared and subjected to immunoblotting. (**E**) GR was stably knocked out in VCaP cells using the CRISPR/Cas9 system and specific sgRNAs. Two cell clones were separately established. WT cells were transfected with a control guide RNA. Cell extracts were examined using immunoblotting for GR and ERG. GAPDH was used to control gel loading. (**F**) GR-knockout VCaP cells were transfected with a GR expression vector and treated with RU486 (10 μM) for 48 h. Whole-cell extracts were subjected to immunoblotting with antibodies against ERG, GR, and vinculin (the loading control). (**G**) VCaP cells were transfected with a GFP-GR plasmid and later treated with cycloheximide (CHX; 50 μg/ml). Cells were harvested for immunoblotting at the indicated time points. The corresponding signals from the blot are presented in a graph. (**H**, **I**) VCaP (**H**) and DU145-tERG#1 (**I**) cells were treated with vehicle or RU486 (10 μM) for 48 h and then treated with cycloheximide (CHX; 50 μg/ml) and/or MG132 (25 μM). Cells were harvested at the indicated time points and extracts prepared for immunoblotting. Western blots that were quantified and normalized are shown as a line graph. Note: all assays were repeated 2–3 times. For the exact *P* values, statistical tests, sample sizes, and error bar definitions for all panels, see Appendix Table S3.

 © The Author(s)

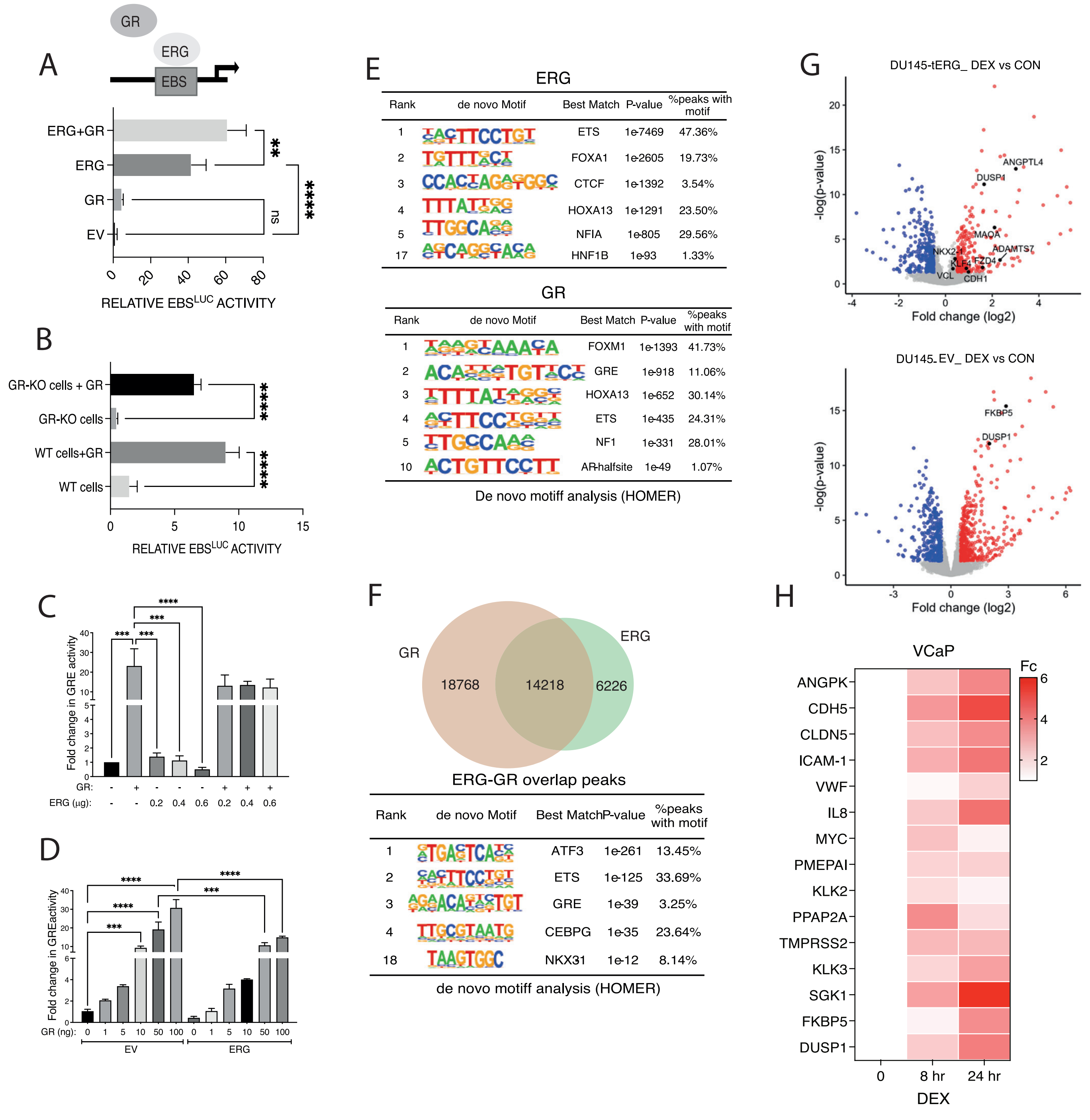
A
GR
ERG
EBS
ERG+GR
ERG
GR
EV
**
****
ns
RELATIVE EBSLUC ACTIVITY
B
GR-KO cells + GR
GR-KO cells
WT cells+GR
WT cells
****
****
RELATIVE EBSLUC ACTIVITY
C
Fold change in GRE activity
GR:
ERG (µg):
D
Fold change in GREactivity
GR (ng):
EV
ERG
E
ERG
Rank
de novo Motif
Best Match
P-value
%peaks with motif
ETS 1e-7469 47.36%
FOXA1 1e-2605 19.73%
CTCF 1e-1392 3.54%
HOXA13 1e-1291 23.50%
NFIA 1e-805 29.56%
HNF1B 1e-93 1.33%
GR
FOXM1 1e-1393 41.73%
GRE 1e-918 11.06%
HOXA13 1e-652 30.14%
ETS 1e-435 24.31%
NF1 1e-331 28.01%
AR-halfsite 1e-49 1.07%
De novo motif analysis (HOMER)
F
GR
ERG
18768
14218
6226
ERG-GR overlap peaks
ATF3 1e-261 13.45%
ETS 1e-125 33.69%
GRE 1e-39 3.25%
CEBPG 1e-35 23.64%
NKX31 1e-12 8.14%
de novo motif analysis (HOMER)
G
DU145-tERG_ DEX vs CON
-log(p-value)
Fold change (log2)
ANGPTL4
DUSP4
MAOA
ADAMTS7
NKX2-1
KLF4
FZD4
VCL
CDH1
DU145_EV_ DEX vs CON
FKBP5
DUSP1
H
VCaP
Fc
ANGPK
CDH5
CLDN5
ICAM-1
VWF
IL8
MYC
PMEPAI
KLK2
PPAP2A
TMPRSS2
KLK3
SGK1
FKBP5
DUSP1
0
8 hr
24 hr
DEX

© The Author(s)

**Figure EV4. Genome-wide analysis of ERG-binding sites following stimulation with DEX (related to Fig. 4).**

(**A**) HEK293T cells were co-transfected with an EBS-luciferase reporter plasmid and the indicated plasmids encoding ERG and GR, or their combination. Luciferase activity was determined 24 h later using the Dual-Luciferase Assay kit (from Promega). Statistical analysis was performed using one-way ANOVA with Tukey's multiple comparisons test. Three biological triplicates were used, and the experiments were repeated twice. Data are presented as mean ± SEM. Error bars represent the mean ± SEM. **P* < 0.05; ***P* < 0.01; ****P* < 0.001; *****P* < 0.0001; ns, not significant. (**B**) Wild-type VCaP cells or the respective GR-KO derivative cells were co-transfected with an EBS-luciferase plasmid. A GR-encoding plasmid, or a control vector, was co-transfected, and 24 h later we performed a luciferase assay, in triplicates. Statistical analysis was performed using one-way ANOVA with Tukey's multiple comparisons test. The experiment was repeated thrice. Data are presented as mean ± SEM. Error bars represent mean ± SEM. **P* < 0.05, ***P* < 0.01, ****P* < 0.001, *****P* < 0.0001. *****P* < 0.0001. (**C**, **D**) Serum-starved HEK293T cells were co-transfected with a GRE-luciferase promoter reporter, along with the indicated amounts of the ERG or/and GR expression vectors. Twenty-four hours later, luciferase activity was measured using a luciferase assay kit. Statistical analysis was performed using one-way ANOVA with Dunnett's multiple comparisons test. We used biological triplicates and repeated the experiment twice. Data are presented as mean ± SEM. Statistical significance is denoted as **P* < 0.05; ***P* < 0.01; ****P* < 0.001 and *****P* < 0.0001. (**E**) Shown are the results of de novo motif analysis featuring top-enriched motifs of ERG and GR peaks (deduced from ChIP-seq analysis of DEX-treated VCaP cells). (**F**) Venn diagram illustrating the overlap of ERG and GR-enriched peaks in DEX-treated DU145-tERG cells. Shown are de novo motif analyses (HOMER) of ERG-GR overlapping peaks featuring the top enriched motifs (ranked by *P* value). (**G**) CSS starved DU145-EV and tERG cells were treated with vehicle or DEX for 24 h. Post treatment, RNA was extracted for sequencing. The volcano plots display the DEGs. ERG and GR target genes are labeled in the plots. Statistical analysis was performed using DESeq2 (Wald test).
(**H**) Serum-starved VCaP cells were treated with DEX (1 µM) for 8 or 24 h. Post-treatment, RNA was harvested and subjected to RT-qPCR using specific primers. GAPDH was used as a housekeeping control transcript. Note: the data shown in (**A**–**D**) were verified in two experiments. Note that the exact *P* values, statistical tests, sample sizes, and error bar definitions for all panels are provided in Appendix Table S3.

 © The Author(s)

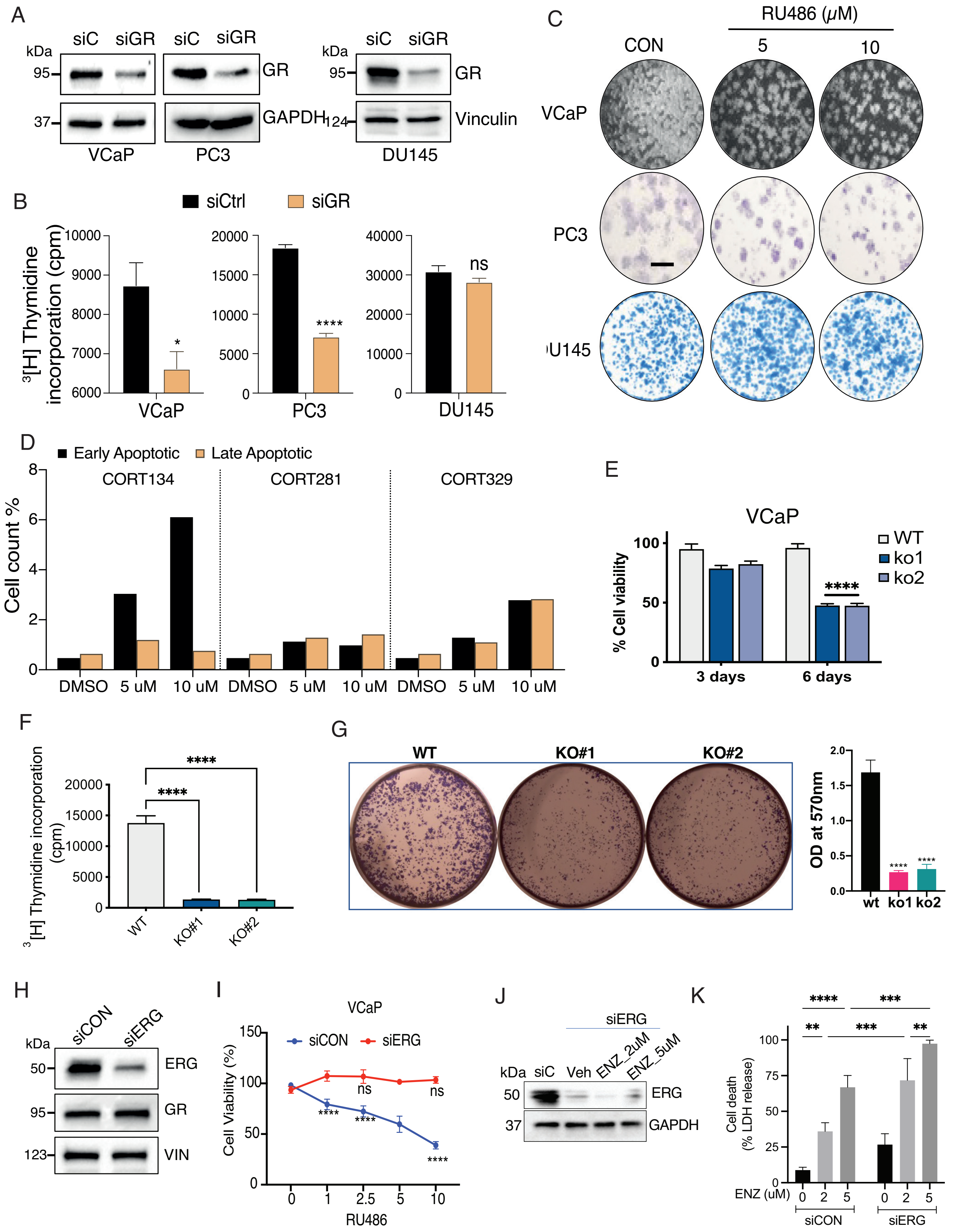

A
siC siGR siC siGR
kDa
95
GR
37
GAPDH
VCaP PC3
kDa siC siGR
95
GR
124
Vinculin
DU145
B
siCtrl siGR
3[H] Thymidine incorporation (cpm)
VCaP PC3 DU145
ns
C
RU486 (μM)
CON 5 10
VCaP
PC3
DU145
D
Early Apoptotic Late Apoptotic
CORT134 CORT281 CORT329
Cell count %
DMSO 5 uM 10 uM DMSO 5 uM 10 uM DMSO 5 uM 10 uM
E
VCaP
% Cell viability
WT
ko1
ko2
3 days 6 days
F
3[H] Thymidine incorporation (cpm)
WT KO#1 KO#2
G
WT KO#1 KO#2
OD at 570nm
wt ko1 ko2
H
siCON siERG
kDa
ERG
GR
VIN
I
VCaP
siCON siERG
Cell Viability (%)
RU486
J
siERG
kDa siC Veh ENZ_2uM ENZ_5uM
ERG
GAPDH
K
Cell death (% LDH release)
ENZ (uM)
siCON siERG

**Figure EV5. Both inhibition and genetic ablation of GR associate with decreased proliferation of PCa cells expressing ETS fusions (related to Fig. 5).**

(**A**, **B**) VCaP, PC3, and DU145 cells were transfected with either GR-specific (siGR) or control (scrambled) siRNAs (siCON). Knockdown efficiency was monitored after 48 h using immunoblotting with antibodies to GR. Cell proliferation was measured 72 h later by applying a radioactive thymidine incorporation assay. Statistical analysis was performed using an unpaired *t* test. The experiment was repeated twice and performed with biological duplicates. Note that representative results are shown. Error bars represent mean ± SEM. **P* < 0.05; *****P* < 0.0001; ns, not significant. (**C**) Shown are representative images corresponding to the colony formation assay and the bar plots shown in Fig. 5. Scale bar, 0.1 mm. (**D**) VCaP cells were seeded in 100-mm dishes. Thereafter, they were treated for 72 h with the vehicle (DMSO; CON) or with the indicated non-steroidal GR antagonists. Shown are the results of an apoptosis assay performed in duplicates using an annexin V/7-AAD kit (from BioLegend). (**E**) GR-knockout VCaP cells were seeded in 96-well plates, and cell viability was measured using the XTT colorimetric assay following 3 or 6 days of incubation. Statistical analysis was performed using unpaired *t* test. The experiment was performed with two biological replicates, and representative results are shown. Data are presented as means ± SEM. (**F**) DNA replication by GR-KO VCaP cells (2 clones) was measured using the thymidine incorporation assay. Statistical analysis was performed using unpaired *t* test. The experiment was repeated twice and performed with two biological replicates; representative results are shown. Data are presented as means ± SEM. (**G**) WT and GR-KO VCaP cells were sparsely seeded in six-well plates. Fifteen days later, cells were fixed and stained with crystal violet. Photos are shown along with bar plots presenting the quantification of colonies. Statistical analysis was performed using unpaired *t* test. The experiment was performed twice with two biological replicates. Data are presented as means ± SEM. *****P* < 0.0001. (**H**, **I**) VCaP cells were transfected with either control oligonucleotides or with siRNAs targeting ERG. Forty-eight hours post transfection, the cells were harvested and subjected to immunoblotting. Alternatively, siCON- or siERG-transfected cells were seeded in 96-well plates and treated with RU486 for 48 h. Cell viability was measured using the XTT assay after 48 additional hours. Statistical analysis was performed using two-way ANOVA with Sidak's multiple comparisons test. The experiment was performed with two biological replicates, and representative results are shown. Data are presented as means ± SEM. *****P* < 0.0001; ns, not significant. (**J**) VCaP cells pre-transfected with siERG or siControl were treated with enzalutamide (2 and 5 μM) for 48 h and subjected to western blot analysis for ERG and GAPDH. (**K**) Shown are the results of LDH cytotoxicity assays measuring cell death in siControl and siERG-transfected VCaP cells treated with enzalutamide. Statistical analysis was performed using two-way ANOVA with Sidak's multiple comparisons test. The experiment was performed twice with two biological replicates; representative results are shown. Data are presented as mean ± SEM. ***P* < 0.01; ****P* < 0.001. Note that the exact *P* values, statistical tests, sample sizes, and error bar definitions for all panels are provided in Appendix Table S3.

 © The Author(s)

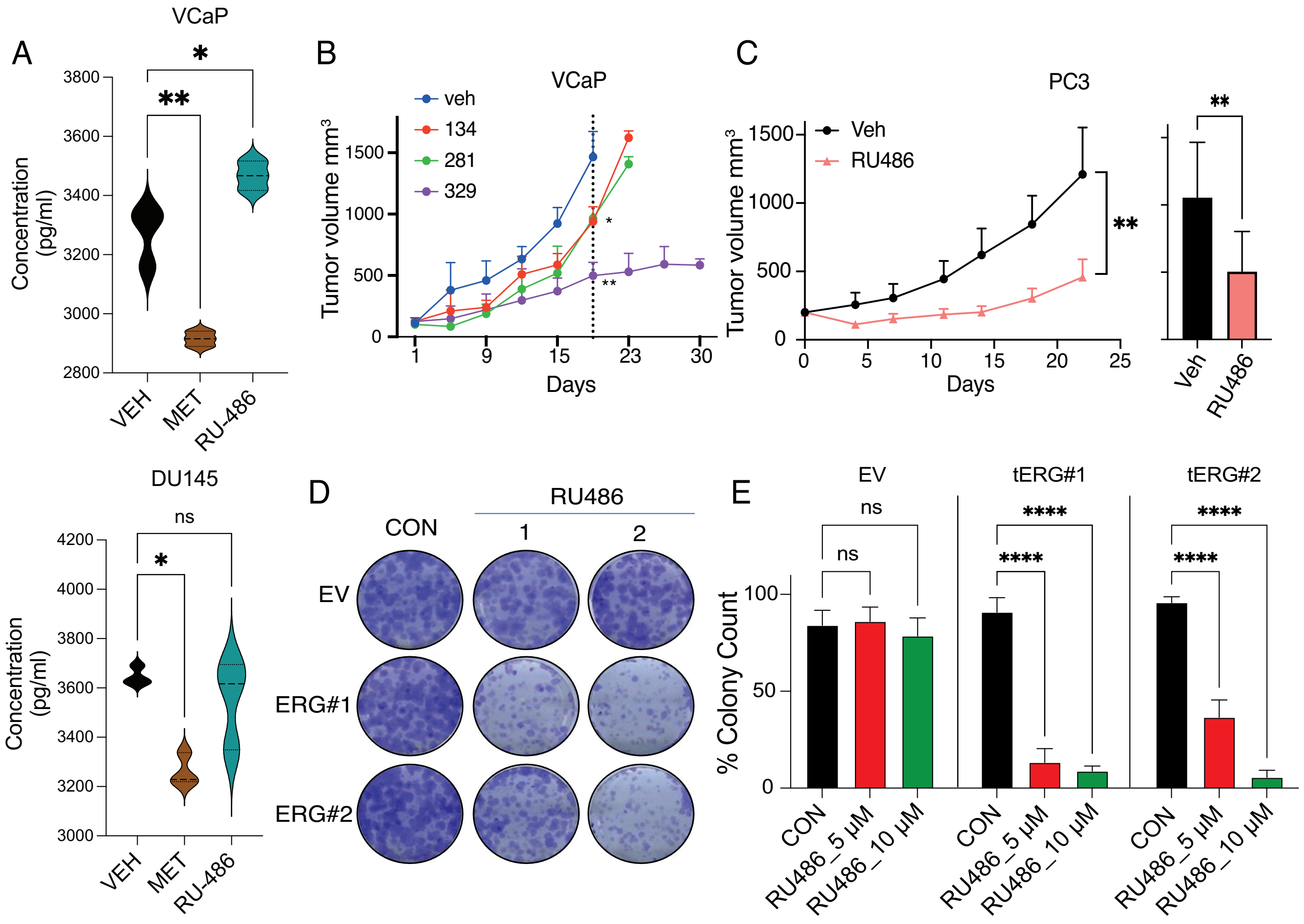


**Figure EV6. GR inhibition suppresses the growth of ERG-expressing PCa cells, whereas cells lacking tERG show no response to the treatment (see Fig. 6).**

(**A**) Corticosterone concentrations (pg/ml) were measured in tumor tissues from Fig. 6A,B. Tumors were processed in parallel, and equal amounts of total protein were analyzed using the DetectX® Corticosterone Enzyme Immunoassay Kit. Data are presented as violin plots with corticosterone concentration and 3 tumors per group. Statistical analysis was performed using one-way ANOVA with Dunnett's multiple comparisons test. $^*P < 0.05$; $^{**}P < 0.01$; n.s., not significant. (**B**) VCaP cells ($2 \times 10^6$) were implanted subcutaneously in athymic mice. Once tumors became palpable, animals were randomized into four groups (3 animals per group), which were daily treated with vehicle or with the indicated non-steroidal GR antagonists (50 mg/kg). The rates of tumor growth are shown. Statistical analysis was performed using two-way RM ANOVA with Dunnett's multiple comparisons test. There were three animals per group. Data are presented as means ± SEM. $^*P < 0.05$; $^{**}P < 0.01$. (**C**) PC3 cells ($5 \times 10^6$) were implanted in animals, which were randomized into groups that were daily treated with vehicle or with RU486 (1 mg/kg). The rates of tumor growth (left panel), along with tumor volumes on day 22 (bar plot), are shown. Statistical analysis was performed using unpaired *t* test. The following numbers of mice were used per group: Vehicle = 8, RU486 = 9. Data are presented as mean ± SEM. $^{**}P < 0.01$. (**D**, **E**) Two clones of DU145 cells stably expressing tERG were sparsely seeded in six-well plates. Cells were later treated once every other day with either vehicle or RU486 (5 μM and 10 μM). Ten days later, all cells were fixed and stained with crystal violet. Representative photos are shown along with bar plots presenting the quantification of colony numbers in five non-overlapping microscope fields. The experiment was repeated twice. Statistical analysis was performed using one-way ANOVA with Dunnett's multiple comparisons test. The number of fields per condition was 5. Data are presented as means ± SEM. $^{****}P < 0.0001$; ns, not significant. Note that the exact *P* values, statistical tests, sample sizes, and error bar definitions for all panels are provided in Appendix Table S3.

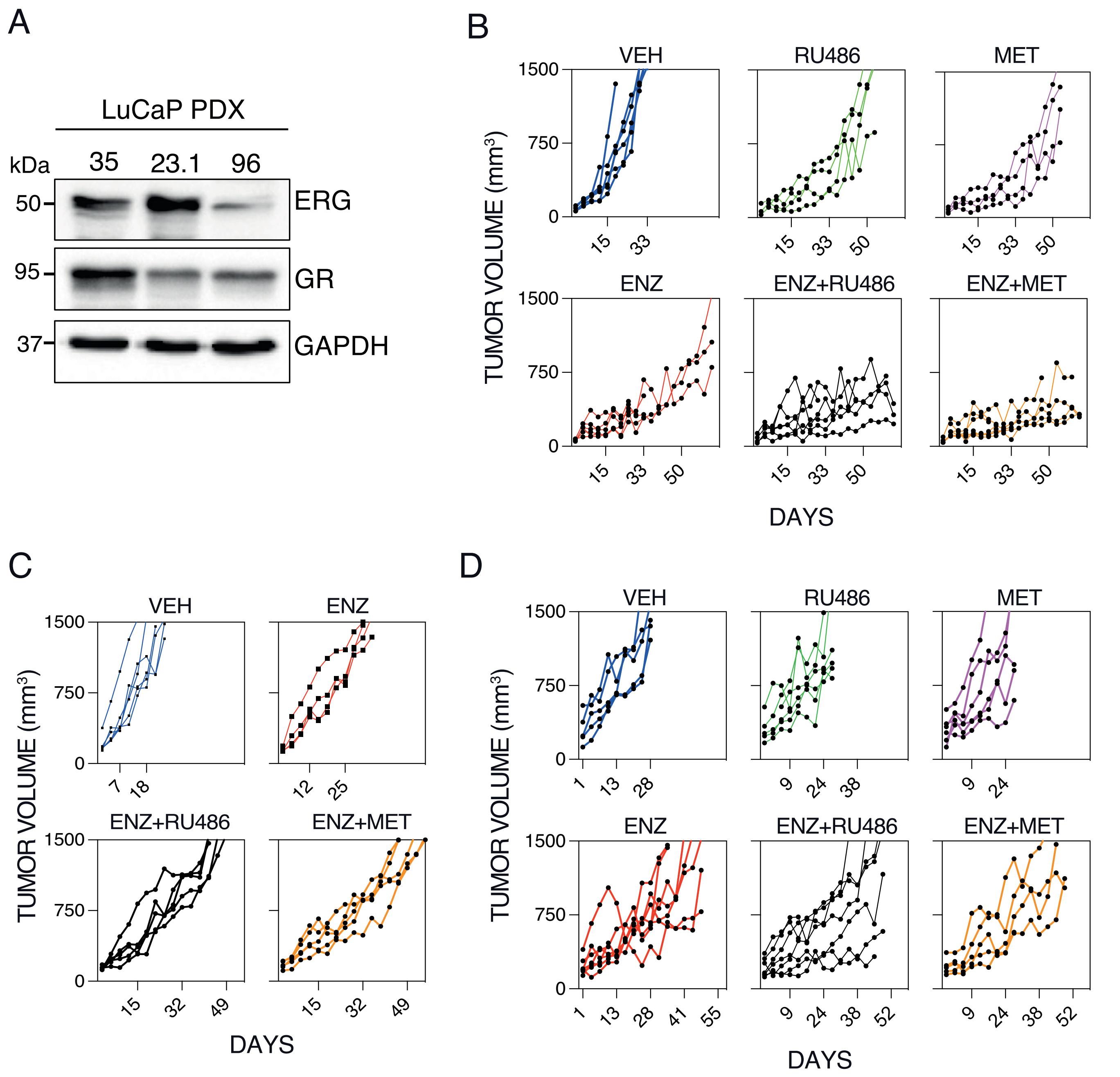


**Figure EV7. Growth rates of three different PDX models in individual mice treated with combinations of GR and AR antagonists (related to Fig. 7).**

(**A**) Whole extracts of the indicated PDX models were subjected to immunoblotting for ERG and GR. GAPDH was used to control gel loading. (**B**) Growth curves of individual tumors derived from the tERG-positive LuCaP 23.1 PDX model. Each line corresponds to one animal. (**C**) Growth curves of individual tumors derived from the tERG-positive LuCaP 35 PDX model. (**D**) Growth curves of individual tumors derived from the tERG-negative LuCaP 96 PDX model.

 © The Author(s)
