# Supplementary material for: TMPRSS2-ERG confers resistance of prostate cancer to antiandrogens
Source: EMBO Mol Med. 2026 May 12;18(6):2062–97. doi: 10.1038/s44321-026-00423-7 (PMC13269939; doi:10.1038/s44321-026-00423-7)
Supplement: Supplementary file 2 — Peer Review File [file 44321_2026_423_MOESM2_ESM.pdf]

EMBO Molecular Medicine

TMPRSS2-ERG confers resistance to antiandrogens: mechanism and therapeutic implications

Arunachalam Sekar, Boobash Raj Selvadurai, Rishita Chatterjee, Lipika Ray, Aakanksha Verma, Nishanth Beluguli Nataraj, Diana Drago Garcia, Suvendu Giri, Alessandro Genna, Feride Karatekin, Nitin Gupta, Deepthi Ramesh-Kumar, Mirie Zerbib, Yaron Vinik, Tamir Avioz, Eviatar Weizman, Eyal David, Alejandro Schäffer, Yunqian Pan, Haojie Huang, Wytske van Weerden, Eva Corey, Hazel Hunt, Andrew Greenstein, Ronnie Blecher-Gonen, Roni Oren, Ariel Afek, Ido Amit, Sima Lev, Anson Ku, Sumeyra Kartal, Jack R. Bright, Rosina T. Lis, William L. Dahut, Adam Sowalsky, Eytan Ruppin, and Yosef Yarden

Corresponding author: Yosef Yarden (yosef.yarden@weizmann.ac.il)

Review Timeline:

Submission Date:	7th Jun 25
Editorial Decision:	2nd Jul 25
Revision Received:	16th Jan 26
Editorial Decision:	16th Feb 26
Revision Received:	21st Mar 26
Accepted:	24th Mar 26

Editor: Zeljko Durdevic

Transaction Report:

(Note: With the exception of the correction of typographical or spelling errors that could be a source of ambiguity, letters and reports are not edited. Depending on transfer agreements, referee reports obtained elsewhere may or may not be included in this compilation. Referee reports are anonymous unless the Referee chooses to sign their reports.)

2nd Jul 2025

Dear Prof. Yarden,

Thank you for the submission of your manuscript to EMBO Molecular Medicine. We have now received feedback from the three reviewers who agreed to evaluate your manuscript. All three referees recognize interest of the study but also raise serious and partially overlapping concerns that should be addressed in a major revision. Considering that the revision will require extensive experimentation we think six months rather than three months would be more appropriate to provide the complete revision. If you would like to discuss further the points raised by the referees, I am available to do so via email or video. Let me know if you are interested in this option.

Further consideration of a revision that addresses reviewers' concerns in full will entail a second round of review. EMBO Molecular Medicine encourages a single round of revision only and therefore, acceptance or rejection of the manuscript will depend on the completeness of your responses included in the next, final version of the manuscript. For this reason, and to save you from any frustrations in the end, I would strongly advise against returning an incomplete revision.

We would welcome the submission of a revised version within six months for further consideration. Please let us know if you require longer to complete the revision.

I look forward to receiving your revised manuscript.

Yours sincerely,

Zeljko Durdevic

Zeljko Durdevic
Senior Editor
EMBO Molecular Medicine

*****

When submitting your revised manuscript, please carefully review the instructions that follow below. We perform an initial quality control of all revised manuscripts before re-review; failure to include requested items will delay the evaluation of your revision.

We require:

1) A .docx formatted version of the manuscript text (including legends for main figures, EV figures and tables). Please make sure that the changes are highlighted to be clearly visible.

2) Individual production quality figure files as .eps, .tif, .jpg (one file per figure). For guidance, download the 'Figure Guide PDF': (https://www.embopress.org/page/journal/17574684/authorguide#figureformat).

3) A .docx formatted letter INCLUDING the reviewers' reports and your detailed point-by-point responses to their comments. As part of the EMBO Press transparent editorial process, the point-by-point response is part of the Review Process File (RPF), which will be published alongside your paper.

4) A complete author checklist, which you can download from our author guidelines (https://www.embopress.org/page/journal/17574684/authorguide#submissionofrevisions). Please insert information in the checklist that is also reflected in the manuscript. The completed author checklist will also be part of the RPF.

5) Please note that all corresponding authors are required to supply an ORCID ID for their name upon submission of a revised manuscript.

6) It is mandatory to include a 'Data Availability' section after the Materials and Methods. Before submitting your revision, primary datasets produced in this study need to be deposited in an appropriate public database, and the accession numbers and database listed under 'Data Availability'. Please remember to provide a reviewer password if the datasets are not yet public (see https://www.embopress.org/page/journal/17574684/authorguide#dataavailability).

In case you have no data that requires deposition in a public database, please state so in this section. Note that the Data Availability Section is restricted to new primary data that are part of this study.

7) For data quantification: please specify the name of the statistical test used to generate error bars and P values, the number (n) of independent experiments (specify technical or biological replicates) underlying each data point and the test used to calculate p-values in each figure legend. The figure legends should contain a basic description of n, P and the test applied. Graphs must include a description of the bars and the error bars (s.d., s.e.m.). See also 'Figure Legend' guidelines: https://www.embopress.org/page/journal/17574684/authorguide#figureformat

8) At EMBO Press we ask authors to provide source data for the main manuscript figures. You will receive a separate email with instructions for providing source data with your revised manuscript, including how to upload and organize the files.

9) Our journal encourages inclusion of *data citations in the reference list* to directly cite datasets that were re-used and obtained from public databases. Data citations in the article text are distinct from normal bibliographical citations and should directly link to the database records from which the data can be accessed. In the main text, data citations are formatted as follows: "Data ref: Smith et al, 2001" or "Data ref: NCBI Sequence Read Archive PRJNA342805, 2017". In the Reference list, data citations must be labeled with "[DATASET]". A data reference must provide the database name, accession number/identifiers and a resolvable link to the landing page from which the data can be accessed at the end of the reference. Further instructions are available at .

10) We replaced Supplementary Information with Expanded View (EV) Figures and Tables that are collapsible/expandable online. A maximum of 5 EV Figures can be typeset. EV Figures should be cited as 'Figure EV1, Figure EV2" etc... in the text and their respective legends should be included in the main text after the legends of regular figures.

- For the figures that you do NOT wish to display as Expanded View figures, they should be bundled together with their legends in a single PDF file called *Appendix*, which should start with a short Table of Content. Appendix figures should be referred to in the main text as: "Appendix Figure S1, Appendix Figure S2" etc.

- Additional Tables/Datasets should be labeled and referred to as Table EV1, Dataset EV1, etc. Legends have to be provided in a separate tab in case of .xls files. Alternatively, the legend can be supplied as a separate text file (README) and zipped together with the Table/Dataset file.

See detailed instructions here:

.

11) The paper explained: EMBO Molecular Medicine articles are accompanied by a summary of the articles to emphasize the major findings in the paper and their medical implications for the non-specialist reader. Please provide a draft summary of your article highlighting

- the medical issue you are addressing,

- the results obtained and

- their clinical impact.

This may be edited to ensure that readers understand the significance and context of the research. Please refer to any of our published articles for an example.

12) Author contributions: You will be asked to provide CRediT (Contributor Role Taxonomy) terms in the submission system. These replace a narrative author contribution section in the manuscript.

13) A Conflict of Interest statement should be provided in the main text.

14) Every published paper now includes a 'Synopsis' to further enhance discoverability. Synopses are displayed on the journal webpage and are freely accessible to all readers. They include a short stand first (maximum of 300 characters, including space) as well as 2-5 one-sentences bullet points that summarizes the paper. Please write the bullet points to summarize the key NEW

findings. They should be designed to be complementary to the abstract - i.e. not repeat the same text. We encourage inclusion of key acronyms and quantitative information (maximum of 30 words / bullet point). Please use the passive voice. Please attach these in a separate file or send them by email, we will incorporate them accordingly.

Please also suggest a striking image or visual abstract to illustrate your article as a PNG file 550 px wide x 300-600 px high.

15) Include a Reagents and Tools Table as part of the Methods section, which can be downloaded from our author guidelines (https://www.embopress.org/page/journal/17574684/authorguide#structuredmethods)

EMBO Molecular Medicine has a "scooping protection" policy, whereby similar findings that are published by others during review or revision are not a criterion for rejection. Should you decide to submit a revised version, I do ask that you get in touch after three months if you have not completed it, to update us on the status.

***** Reviewer's comments *****

Referee #1 (Remarks for Author):

In this study, the authors describe physical and functional interaction between GR and ERG that stabilizes ERG, reshapes its transcriptional program, and promotes resistance to anti-androgen therapy. The work is generally well executed and supported by significant data. However, several points require clarification or correction. In addition, the same group previously described functional ERG-GR interactions which somewhat limits the novelty. Furthermore, two of the cohorts that were examined have few patients and the third has a low R score in the analysis, making it difficult to determine the validity of the model for human prostate cancer.
Specific points:
(1) The sample size for each group in Figures 1C-D and S1B-C should be stated in the figure panels or legends. It is necessary with analysis in additional cohorts.
(2) GR-ERG colocalization experiments presented in Figure 2B should be performed in prostate cancer cells. Similarly, the IP-Western data in Figure 2D should be carried out in prostate cancer cells.
(3) Experiments investigating the domain requirements in GR and ERG for their interactions, presented in Figure S2, should be validated by IP-Western.
(4) In Figure 3, the RU486 toxicity observed in VCaP cells at the dose and time frames used (noted in Results, but data not shown) is a concern. It seems that the authors are using 5-10X higher concentrations of RU486 that are sufficient to fully inhibit GR in other settings. A dose response experiment with ERG integrity and GR activity would clarify this point.
(5) The manuscript notes that "Treatment resistance was also associated with high post-treatment ERG IHC (p = 0.0073; Fig. 1C; upper panel)," yet Figure 1C displays ERG transcript levels. This should be corrected. Additionally, legends for Figure S1D and S1E are also mis-labeled as "C" and "D," respectively.
(6) Although the GR-ERG interaction is well characterized in cell lines, it has not been validated in vivo. In-situ PLA or dual-stain IHC on post-treatment prostatectomy specimens, or on tumors from xenograft experiments, would strengthen the findings.
(7) Because RU486 antagonizes GR and progesterone receptors and shows partial anti-androgen activity, conclusions based only on RU486 are not conclusive. Key viability assays should be repeated with at least one highly selective GR antagonist that lacks PR/AR cross-reactivity (e.g., relacorilant) and, ideally, include GR-knockout controls. In addition, more prostate cancer cell lines should be included.
(8) GR antagonism is tested only in xenograft/PDX models in severely immunodeficient mice. Given that systemic GR inhibition can markedly influence immune function, the translational relevance and safety of combining GR blockade with ADT remain uncertain. Incorporating immunocompetent syngeneic models would address this limitation. In addition, it would be desirable to show global transcriptomic changes also in the in vivo experiments to validate the model that the authors propose.

Referee #2 (Comments on Novelty/Model System for Author):

This is an interesting study. It aligns well with EMM's editorial scope and provides novel clinical insights into the role of ERG overexpression in patients with castration-resistant prostate cancer (CRPC). These findings open promising clinical perspectives, especially given that GR is a pharmacologically accessible target with limited adverse effects. The research employs a relatively broad range of experimental techniques.

However, I would express a reservation regarding the structure of the manuscript. Some key information is introduced gradually, whereas presenting it earlier would improve the reader's understanding. For example, the rationale behind the selection of homologous/heterologous cell lines to dissect molecular mechanisms would be more impactful if discussed upfront.

Despite the limitations imposed by the 3Rs principles, the xenograft-based investigations are of high interest. Nevertheless, the study would benefit from being conducted on a larger number of animals to strengthen the robustness of the conclusions. Including images of the xenograft-derived tumors-such as gross morphology, histological sections, and immunostainings-would also add significant value to the manuscript.

I recommend a revision addressing the points raised in the reviewer comments to elevate the manuscript to a publishable standard in EMM.

Referee #2 (Remarks for Author):

The article and the proposed research focus on the role of glucocorticoids in the development of resistance to anti-androgen therapy in prostate cancer. The authors initially investigate the overexpression of ERG, which commonly occurs following chromosomal rearrangements leading to the TMPRSS2-ERG fusion. ERG overexpression in patients is associated with resistance to androgen deprivation therapy.
An interaction between the glucocorticoid receptor (GR) and ERG is reported, and this interaction can be modulated by GR agonists and antagonists. This crosstalk regulates ERG stability through ubiquitination and proteasome-mediated degradation processes and also influences its transcription factor activity. The GR-ERG interaction modulates signaling pathways involved in cell growth and survival.
In vivo, the combined blockade of GR and AR using antagonists shows selective efficacy, specifically in PDX models exhibiting ERG overexpression.
Overall, the paper is well written, and the experimental findings effectively support the conclusions. However, some points still require clarification:

Major Points
1. Fig. 2A, 2F: Please include vehicle histograms with error bars and corresponding statistical tests.
Fig. 2B: Dex treatment appears to alter ERG localization within the nucleus. The authors should elaborate and provide hypotheses to explain this observation. The rationale for using HeLa cells instead of human prostatic cell lines is unclear-"low signal to noise" is insufficient. Please provide more details (e.g., levels of ERG and GR accumulation, which prostate cell lines were tested, etc.). These results should be included in the supplementary material.
2. Fig. 2D: The authors should perform a reciprocal immunoprecipitation using an anti-GR antibody to confirm the interaction between GR and ERG.
3. The authors used multiple cell models to investigate ERG-GR interaction and crosstalk, including homologous models (VCaP, DU145, PC3, RWPE) and heterologous ones (HeLa, HEK293). Figure S5A, which summarizes the protein accumulation levels of interest, including western blots for ETV1, ETV4, and ETV5, should be moved earlier in the manuscript or at least in the supplementary figures. This information could be useful to justify the choice of specific cell types throughout the study.
4. Fig. 5E and 5F: The authors explored the molecular signature associated with RU486 treatment and proposed several ERG targets potentially responsible for the observed apoptotic phenotype. Knockdown or overexpression experiments of these candidate targets should be performed to identify downstream pathways that significantly contribute to GR antagonism-induced cell death.
5. Fig. 7: Histological analysis or gene expression profiling of tumor xenografts should be included to assess proliferation (e.g., Ki67 staining, CCND1 expression), anti-androgen treatment efficacy (expression of AR target genes), as well as representative images from necropsy (H&E staining or tumor pictures). The number of mice per group (4 to 6, as stated in the legend) is low for a survival study. Additionally, in some Kaplan-Meier curves, fewer than four animals appear to be present-especially in the Enzalutamide groups. Please clarify this and/or increase sample size.

Minor Points
• Page 7, line 3, line 18, etc.: Correct spelling of "Previously."
• Fig. 1D: "Probability of survival" is not appropriate; please use "Overall survival." Also, Fig. 1D is not referenced in the main text.
• Page 7, line 5: The working model is in Fig. 1E, not Fig. 1D.
The working model should be presented at the end of the article or as a graphical abstract, in accordance with EMM recommendations.
• The meaning of "CORT" must be clarified in the legends for Fig. S2C and Fig. 2F. Overall, the naming of CORT-related agents must be standardized across the manuscript text, figure legends, and figures.
• Fig. S2E: According to the text, ERG is tagged with HA. Please include this detail in the figure legend.
• Page 9, line 24: "Slightly stronger interaction"-if the difference between the GR_3 construct and full-length GR is not statistically significant, this statement should be removed.
• Fig. 3C: Please clarify the meaning of histogram colors in the figure legend.
• Page 10, line 15: "Inconsistent changes in the abundance of mRNAs encoding tERG" seems contradictory with the figure, which shows a significant increase from 0 h to 72 h. Please revise the text for clarity.
• Fig. 4E: Revise the legend to clearly distinguish between solid and dashed lines.
• Fig. 7: Standardize the size of curves and legends. Is there a reason why the MET condition is absent in panel 7C? Please indicate which curves were compared to calculate the log-rank statistics.
• Overall, the statistical tests used should be specified in the figure legends, even if they are partially described in the Methods

section.

Referee #3 (Remarks for Author):

The TMPRSS2-ERG fusion gene (tERG) is frequently discovered in prostate cancer patients, which leads to the androgen-inducible overexpression of ERG protein. Although a high level of tERG has been reported to indicate high Gleason scores and more advanced disease, whether tERG confers antiandrogen resistance in prostate cancer is unknown. Emerging evidence suggests that GR overexpression and sustained glucocorticoid could take over androgen-AR's role in promoting prostate cancer progression under the enzalutamide treatment. Because GR physically interacts with other ETS family members, as suggested by the author's previous study, the author proposed to investigate whether TMPRSS2-ERG confers resistance to antiandrogens via direct binding to GR. By analyzing clinical trial data, the study found that patients with tERG-positive fusions have worse outcomes with antiandrogen treatment. Mechanistically, this work revealed that GR binding stabilizes ERG protein, then strengthens ERG's transcriptional activity. Furthermore, the xenograft studies demonstrated that tERG-positive models are more sensitive to a combination of GR and AR inhibitors compared to tERG-negative models. Overall, this is a good study that focuses on enzalutamide-resistant prostate cancer. It has the potential to develop tERG as a biomarker to predict the response of prostate cancer patients to antiandrogen treatment and select the patients who are highly sensitive to the combination treatment of AR and GR inhibitors. The manuscript is well written and referenced. However, there are several major and minor points that need to be addressed by the authors.
Major points:
1. The data for the matched pre- and post-treatment samples should be analyzed and provided to confirm that enzalutamide can increase the transcriptional and protein levels of ERG and GR in nonresponders.
2. The data supporting the induction of ERG levels by enzalutamide in cell models and xenograft models are missing. Although VCaP is tERG positive, it's enzalutamide sensitive. It's unclear if enzalutamide-resistant VCaP will have higher tERG and whether the tERG knockdown will resensitize the cells to enzalutamide. In the PDX models, it's unknown if enzalutamide induces tERG expression. Additional tERG-negative, AR-positive cell models (such as LNCaP, LNCaP/AR, LAPC4, etc.) should be involved to evaluate whether enzalutamide increases ERG expression levels when the cells develop enzalutamide resistance.
3. Does ERG knockdown make the tERG cells become sensitive to enzalutamide?
4. Does reinstating the tERG into tERG-negative, AR-positive cell models (such as LNCaP, LNCaP/AR, LAPC4, ETC.) cause the cells to become insensitive to enzalutamide resistance?
5. Does GR overexpression rescue the ERG protein loss induced by RU486 and GA?
6. Does ERG overexpression rescue the GR inhibitor-suppressed cell and xenograft growth?
7. Does MET suppress the corticosterone levels in mouse serum and tumor tissues? And will DEX reverse the suppression of tumor growth induced by MET?
Minor points:
1. Fig.1 and 1S need more information to be clear. For example, in Fig.1A and B, what do the bars represent? What are the green dashed lines? In Fig.S1, the bar graph of the ERG IHC summary is missing. What's the unit for the y-axis in panel C?
2. Fig.2 shows that the GR and ERG interaction in HeLa cells may not reflect the relationship between ERG and GR in prostate cancer cells.
3. Fig.6 should involve enzalutamide treatment in the experiments.
4. Are the results of Fig. S3H and I reproducible in VCaP cells with endogenous ERG?
5. The discussion of the most recent clinical trial about the combination of ORIC-101 with enzalutamide, which did not produce clinical benefit in men with metastatic prostate cancer resistant to enzalutamide, is missing.

Lists of Corrections Introduced in the Revised Manuscript in Response to the Critical Comments of the Three Referees

A note to the referees

On June 15, 2025, our laboratory together with several neighboring research groups and our institutional animal and core facilities sustained severe damage as a result of ballistic missile strikes launched by the Islamic Republic of Iran. We are deeply grateful to the Editor for granting an extended revision period under these extraordinary circumstances.

Despite the significant challenges imposed by the evacuation of international research fellows and the temporary loss of essential instrumentation, we made every effort to carefully address all substantive comments raised by the reviewers. We believe that this rigorous review process has meaningfully strengthened the manuscript, and we sincerely appreciate the referees' constructive input and patience.

Referee #1 (Remarks for Author):

In this study, the authors describe physical and functional interaction between GR and ERG that stabilizes ERG, reshapes its transcriptional program, and promotes resistance to anti-androgen therapy. The work is generally well executed and supported by significant data. However, several points require clarification or correction. In addition, the same group previously described functional ERG-GR interactions which somewhat limits the novelty. Furthermore, two of the cohorts that were examined have few patients and the third has a low R score in the analysis, making it difficult to determine the validity of the model for human prostate cancer.

Specific points:

(1) The sample size for each group in Figures 1C-D and S1B-C should be stated in the figure panels or legends. It is necessary with analysis in additional cohorts.

In response to this comment from Referee 1, we indicated the numbers of patients in the figures and legends to Figures 1C-D and S1B-C. In addition, the number of patients included in the clinical trial performed by Hussain and colleagues (31 patients; (Hussain *et al*, 2018)) was inserted in panel D of Figure 1. Please find below the revised panels of Figures 1 and S1.

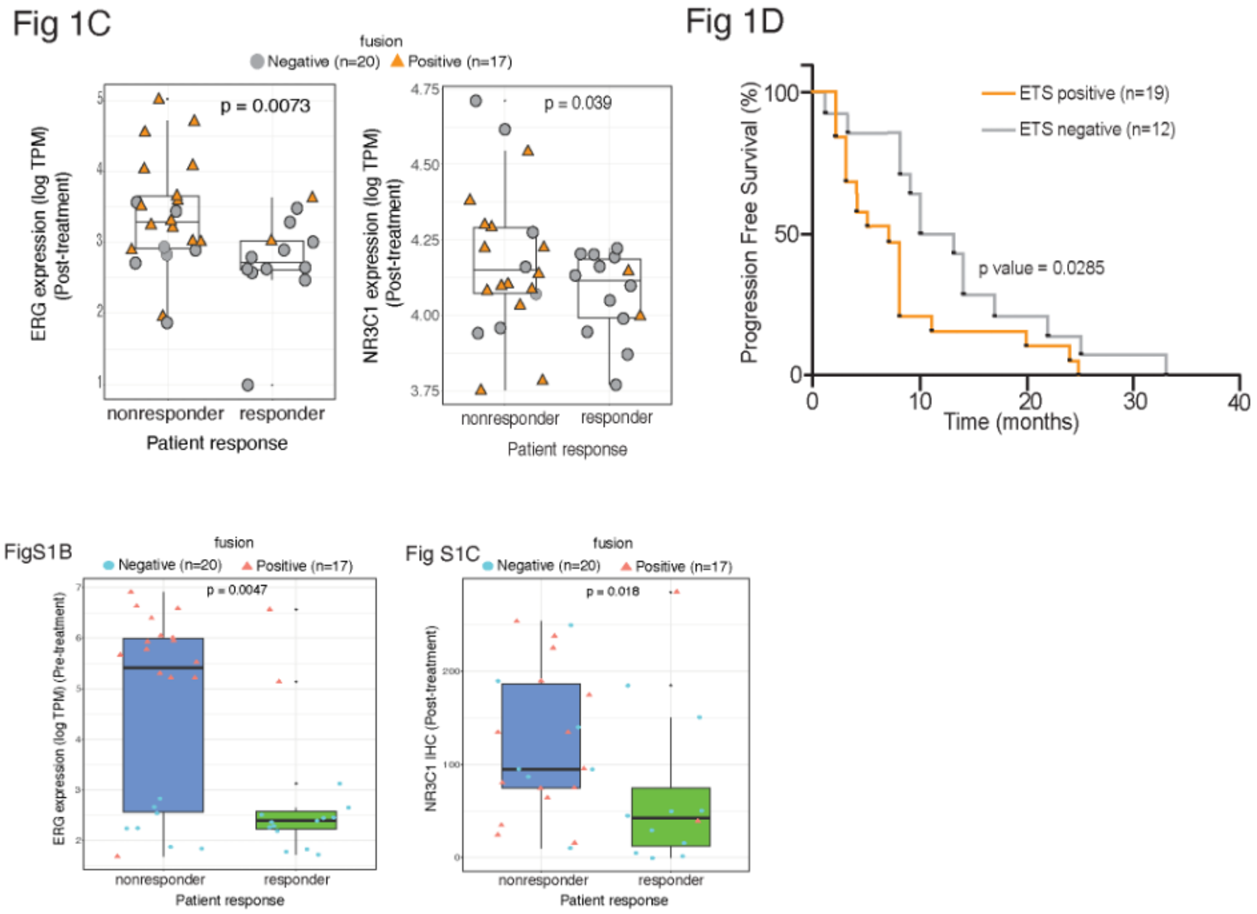

Figure I: Revised panels of Figures 1 and S1. Note that we added the number of patients in each panel, as requested by this referee.

(2) GR-ERG colocalization experiments presented in Figure 2B should be performed in prostate cancer cells. Similarly, the IP-Western data in Figure 2D should be carried out in prostate cancer cells.

(i) As requested by this referee, we addressed GR-ERG co-localization in prostate cancer cells, specifically the VCaP cell line. To detect the inducible co-localization post hormonal stimulation, we used the proximity ligation assay (PLA) and antibodies specific to GR and ERG. The results we obtained (see Figure II, below), independently of the previously presented immunofluorescence assay, supported the ability of the ligand-bound GR to bind with ERG.

(ii) To address the IP-Western comment, we performed co-immunoprecipitation (co-IP) experiments using prostate cancer cell lines. Specifically, co-IP assays were carried out in VCaP and PC3 cells and the results are presented below (Figs. II-B, II-C and II-D).

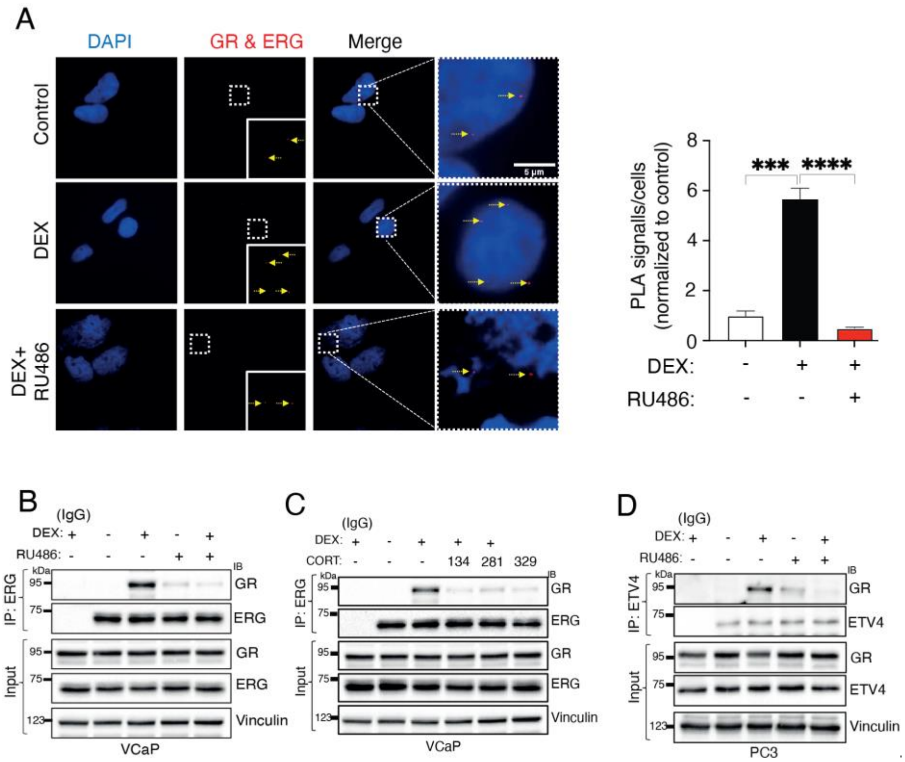

Figure II: Treatment of prostate cancer cells with DEX increases formation of complexes containing ERG and GR. (**A**) Shown are representative images of VCaP prostate cancer cells that were starved overnight for serum factors and then treated for 30 minutes with either DEX (1 µM) or a combination of DEX and RU486 (10 µM). The PLA test detected short-range (10-40 nm) interactions between GR and ERG by means of a pair of oligonucleotide-labeled secondary antibodies and hybridizing connector oligonucleotides, which link the PLA probes only if they are in close proximity to each other. Nuclei were labelled with DAPI (blue). The left panel depicts the images of clusters (in red; arrowheads) and merge panels, whereas the right panel shows a histogram with the quantification of the PLA signals (n>40 cells for each condition). The experiment was repeated twice (***, $p < 0.01$). The error bars indicate SEM. Scale bar, 5 µm. (**B**) Serum-starved VCaP cells were treated for 60 minutes with vehicle, DEX (1 µM), RU486 (1 µM), or the combination of drugs. Extracts were processed for co-immunoprecipitation (IP) and immunoblotting (IB). (**C**) Serum-starved VCaP cells were treated for 60 minutes with vehicle (CON) or DEX (1 µM), in the absence or presence of the indicated SGRM compounds (C134, C281 and C329; 10 µM each). Whole cell extracts were processed for co-IP of ERG and GR, followed by immunoblotting. (**D**) Serum-starved PC3 cells (endogenously overexpressing ETV4) were treated for 60 minutes with vehicle, DEX (1 µM), RU486 (1 µM), or the combination of drugs. Extracts were processed for co-IP and IB. IgG, control antibody.

(3) Experiments investigating the domain requirements in GR and ERG for their interactions, presented in Figure S2, should be validated by IP-Western.

As requested, we repeated the domain mapping analyses using IP-Western and a prostate cancer cell line, namely DU145. The results shown below validate the data presented in the original manuscript.

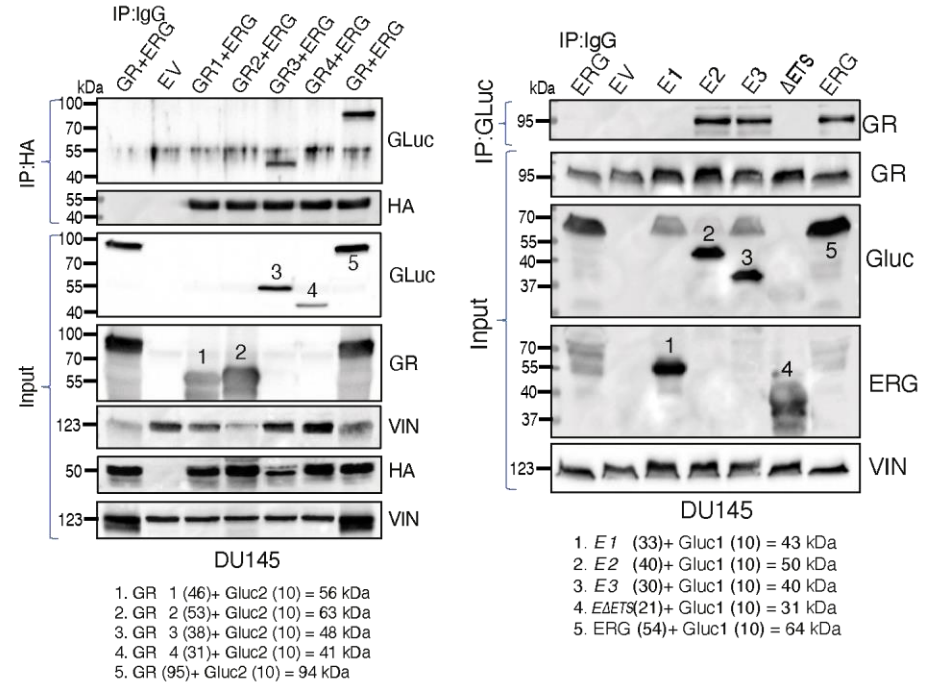

Figure III: The DBD-HR-LBD region of GR interacts with the ETS domain of ERG. (**left panel**) DU145 cells were transfected with Gluc1-ERG plasmids encoding different deletion mutants of ERG (see molecular weight calculations at the bottom of the panel). The Gluc1-ERG proteins were immunoprecipitated (IP) using a specific antibody. Immunoblotting (IB) was performed using antibodies that detected the endogenous forms of GR. (**right panel**) DU145 cells were co-transfected with HA-tagged ERG, along with the indicated GR-Gluc2 plasmids encoding different deletion mutants of GR (see a list at the bottom of the figure). Post-transfection, whole cell extracts were subjected to immunoprecipitation using an anti-HA antibody and immunoblotted for Gluc. A fraction of the lysate was used as an input control. IgG, control immunoglobulin G. VIN, vinculin.

(4) In Figure 3, the RU486 toxicity observed in VCaP cells at the dose and time frames used (noted in Results, but data not shown) is a concern. It seems that the authors are using 5-10X higher concentrations of RU486 that are sufficient to fully inhibit GR in other settings. A dose response experiment with ERG integrity and GR activity would clarify this point.

We thank the reviewer for this important comment. To address the concern regarding RU486 toxicity and dosing, we performed dose–response assays using increasing concentrations of RU486 in multiple prostate cell lines, including VCaP, PC3, CL1, DU145, and the normal prostate epithelial cell line RWPE-1. The logic was to test whether the GR-ERG cooperative mechanism is specific to ERG-positive cells or represents general cytotoxicity. Thus, the cellular panel was designed to test three critical dimensions: (i) normal vs. transformed cells (RWPE-1 vs. the cancer lines), (ii) ERG-positive vs. ERG-low/negative cancers (VCaP vs. DU145), and (iii) ETS family specificity (PC3-ETV4+, CL1-ETV1+). Cell viability was assessed using the XTT assay (in triplicates). Likewise, immunoblotting of cell extracts was used to determine effects on ERG stability. The results shown below demonstrate dose-dependent effects of RU486 across several prostate cancer cell lines and provide context for the concentrations used in the study presented in original Figure 3. Notably, RWPE-1 cells tolerated RU486 at concentrations that inhibited the ERG-positive VCaP cells, which establishes both cancer cell specificity and a therapeutic window. Thus, the dose-response data directly explain why we used 5-10 μM RU486 concentrations. For example, the ERG-positive VCaP cells showed significantly greater RU486

sensitivity compared to ERG-low DU145 and normal RWPE-1 cells. This differential sensitivity validates that higher antagonist concentrations selectively target the GR-ERG axis rather than causes non-specific toxicity. Importantly, the immunoblotting panel complements the viability study by showing dose- and time-dependent ERG degradation in DU145-tERG cells upon RU486 treatment. This demonstrates that disrupting GR function destabilizes the ERG protein, providing a molecular mechanism for the selective sensitivity we observed.

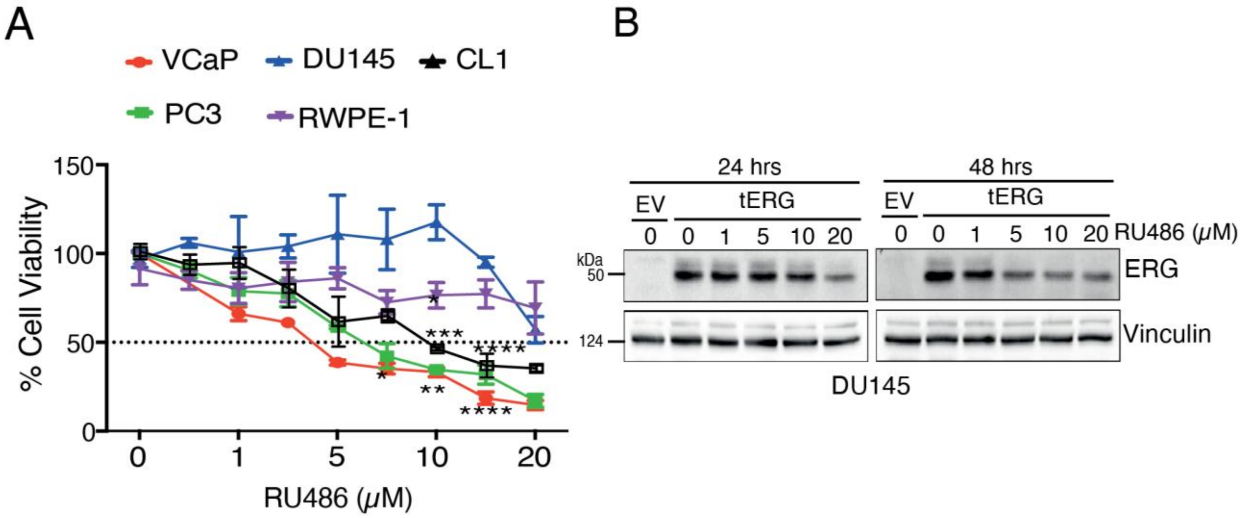

Figure IV: Dose-dependent effects of RU486 on prostate cancer cell lines. (**A**) The XTT cell viability assay was performed with the following prostate cell lines: VCaP (ERG+), PC3 (ETV4+), CL1 (ETV1+), DU145 (ERG-low) and RWPE-1 (normal prostate epithelial cells). All cell lines were treated in triplicates for 96 hours with increasing concentrations of RU486. (**B**) DU145 cells stably overexpressing tERG were treated for 24 or 48 hours with increasing concentrations of RU486. Thereafter, whole cell extracts were prepared and subjected to immunoblotting, as indicated.

(5) The manuscript notes that "Treatment resistance was also associated with high post-treatment ERG IHC (p = 0.0073; Fig. 1C; upper panel)," yet Figure 1C displays ERG transcript levels. This should be corrected. Additionally, legends for Figure S1D and S1E are also mis-labeled as "C" and "D," respectively.

(i) As requested, we corrected the description of Figure 1C, such that the revised text refers to ERG's transcripts rather than to the ERG protein.

(ii) We are not sure that we understand the comment about figure mislabeling.

(6) Although the GR-ERG interaction is well characterized in cell lines, it has not been validated in vivo. In-situ PLA or dual-stain IHC on post-treatment prostatectomy specimens, or on tumors from xenograft experiments, would strengthen the findings.

To address this comment of Referee 1 we performed dual stain IHC, anti-GR and anti-ERG, on serial slides from 62 patients who underwent standard-of-care prostatectomies with no hormonal therapy (see Figure V, below). Next, we performed a one-sided Fisher's exact test to examine whether co-occurrence was favored. This focus-level analysis showed that the same cells expressing tERG are more likely to express GR (p=0.0095). In conclusion, the dataset we newly obtained shows that ERG and GR are favored to be mutually co-existing. Conceivably, tumor

cell survival is optimal in the presence of both ERG and GR, such that mutual exclusivity is not favored.

A

P value and statistical significance			
Test	Fisher's exact test		
P value	0.0095		
P value summary	**		
One- or two-sided	One-sided		
Statistically significant (P < 0.05)?	Yes		
Data analyzed	ERG+	ERG-	Total
GR+	15	6	21
GR-	15	26	41
Total	30	32	62

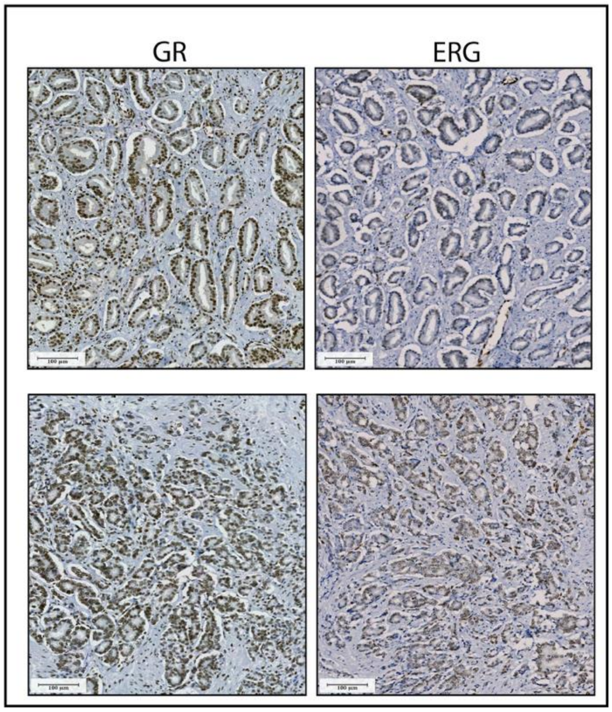

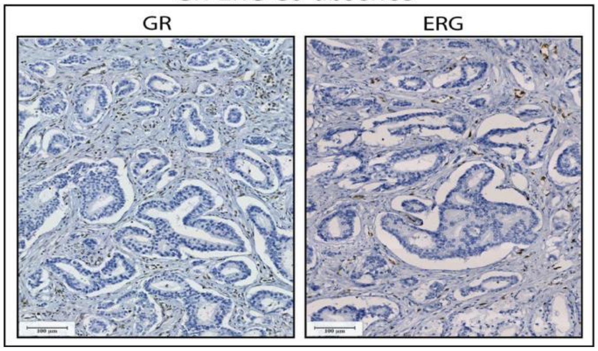

Figure V: ERG and GR are likely to coexist and are not mutually exclusive. Thin tissue sections were prepared from sixty-two patients who underwent standard-of-care prostatectomies with no hormonal therapy. Immunohistochemistry analysis was performed using anti-GR and anti-ERG antibodies. (**A**) The table presents results obtained after performing a one-sided Fisher's exact test that examined whether co-occurrence of GR and ERG was favored. Listed are the numbers of ETS-positive and ETS-negative tumors, along with the status of GR expression. (**B** and **C**) Shown are examples IHC analyses from representative GR and ERG negative tumors and ERG and GR positive tumors.

(7) Because RU486 antagonizes GR and progesterone receptors and shows partial anti-androgen activity, conclusions based only on RU486 are not conclusive. Key viability assays should be repeated with at least one highly selective GR antagonist that lacks PR/AR cross-reactivity (e.g., relacorilant) and, ideally, include GR-knockout controls. In addition, more prostate cancer cell lines should be included.

As requested by this Referee, we performed key viability experiments using highly selective GR antagonists that lack PR/AR cross-reactivity. In addition, we included both parental prostate cancer cells, VCaP, and their derivative, GR-knockout cells. Both lines were treated for 72 hours with the following selective GR modulators (SGRMs): CORT125134 (relacorilant), CORT125281 (exicorilant) and CORT125329 (C329). Cell proliferation was measured by applying a radioactive thymidine incorporation assay. As shown in Figure VI (below), each drug was able to significantly reduce viability of the parental cells but, as anticipated, the GR-KO cells displayed no sensitivity to the three highly selective drugs. Note that our experiments made use of SGRMs that were made available to us by Corcept Therapeutics.

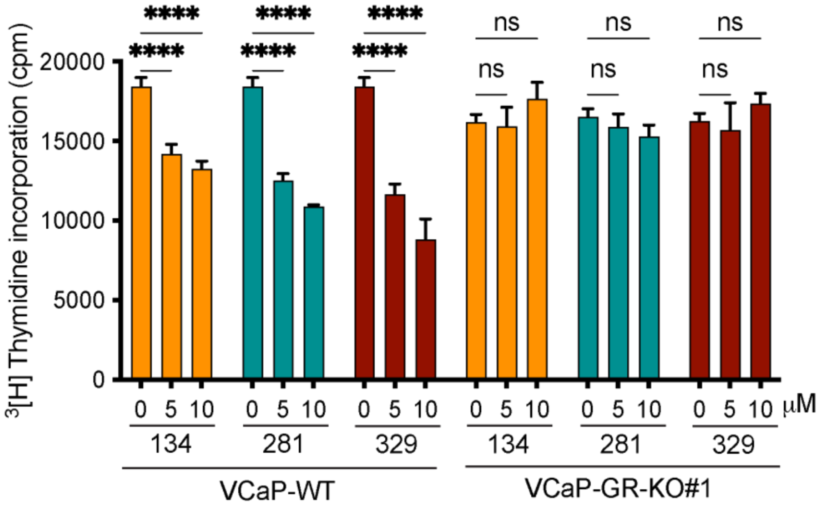

Figure VI: Blocking GR using selective GR modulators (SGRMs) inhibits parental prostate cancer cells while sparing GR-knockout derivatives. Parental VCaP cells or the derivatives, GR-KO, were treated with the indicated inhibitors from Corcept. Cell proliferation was measured 72 hours later by applying a radioactive thymidine incorporation assay. ****, $p < 0.0001$; ns, not significant.

In addition to the data presented in Figure VI, below we indicate additional experiments of the revised manuscript that utilized non-steroidal GR antagonists and three different prostate cancer cell lines:

- Figure 5B (viability assays; VCaP, PC3 and DU145 cells)
- Figure S5E (VCaP cell proliferation test), and
- Figure S6A (time series of VCaP xenograft assays performed in immunocompromised mice).

(8) GR antagonism is tested only in xenograft/PDX models in severely immunodeficient mice. Given that systemic GR inhibition can markedly influence immune function, the translational relevance and safety of combining GR blockade with ADT remain uncertain. Incorporating immunocompetent syngeneic models would address this limitation. In addition, it would be desirable to show global transcriptomic changes also in the in vivo experiments to validate the model that the authors propose.

We thank the reviewer for this constructive suggestion. We have now performed RNA-sequencing analyses on patient-derived tumors from one tERG-positive model (LuCaP 23.1) and the tERG-negative (LuCaP 96) PDX model, with the goal of providing global transcriptomic validation of the proposed working model. As shown in Figure VII-A, PCA analysis of the combined dataset demonstrates that LuCaP 23.1 ($tERG^+$) and LuCaP 96 ($tERG^-$) tumors exhibit fundamentally distinct transcriptional programs, with PC1 capturing 47% of variance between the two models. This is consistent with our in vitro findings demonstrating that the status of ERG fundamentally alters the transcriptional landscape and drug response in prostate cancer.

In addition, PCA analysis (Figs. VII-B and VII-C) of the two PDX models revealed that GR antagonism (ENZ+RU486 or ENZ+MET) induces distinct transcriptional reprogramming compared to ENZ monotherapy, with clear separation of the various treatment groups. GSEA

analysis of Hallmark Pathways (Panel D) demonstrated robust modulation of several key biological processes in response to the combination therapy: (i) suppression of both the Androgen Response and the Estrogen Response signatures, consistent with dual blockade of AR and GR signaling; (ii) downregulation of cell cycle and proliferation pathways, including E2F Targets, G2M Checkpoint, and Mitotic Spindle; (iii) strong suppression of MYC Targets V1/V2 which directly supports our model arguing that GR-tERG complexes drive MYC transcription and that disrupting this complex diminishes MYC-driven oncogenic programs; (iv) reduced growth signaling through the mTORC1 and PI3K-AKT-mTOR pathways; and (v) modulation of metabolic programs, which includes Oxidative Phosphorylation, Glycolysis and Fatty Acid Metabolism.

Expression analysis of AR target genes (i.e., AR and KLK3) and GR/ERG-regulated genes (NKX3-1, SGK1, BRD4, RHOA; Fig. VII-E) confirmed that the combination therapy using GR antagonists effectively suppressed both AR and GR/ERG transcriptional output in vivo. Notably, while ENZ monotherapy suppressed AR targets, GR and ERG target genes remained elevated, supporting our model that GR compensates for AR blockade, specifically in tERG-positive tumors. The addition of GR antagonists effectively suppressed ERG target genes like NKX3-1, consistent with our finding that GR forms a physical complex with tERG, relieves its intrinsic auto-inhibition, and enhances transcription from ERG binding sites. In contrast, the tERG- LuCaP 96 model (Fig. VII-C) showed minimal separation between treatment groups upon GR antagonism, indicating that GR blockade does not substantially alter the transcriptional landscape in the absence of tERG.

Importantly, both GSEA analysis (Fig. VII-D) and heatmap comparisons (Fig. VII-F) demonstrated that GR antagonism generates only minimal transcriptional changes in the tERG-negative model, relative to the robust effects observed in the tERG-positive tumors. Notably, MYC targets and other cell proliferation pathways were not significantly modulated by GR antagonism in the tERG-negative model. This differential response is mechanistically consistent with our model: in the absence of tERG, GR cannot form the oncogenic GR-tERG complex that engages ERG binding sites and drives transcription of MYC and other oncogenic targets.

In conclusion, these global transcriptomic data from in vivo models provide strong validation of our proposed model, wherein GR-tERG complexes drive MYC target gene expression and sustain tumor cell proliferation, conferring enzalutamide resistance specifically in fusion-positive prostate cancer.

Note: The data presented in Figure VII extend substantially beyond the scope of the present manuscript. Moreover, additional independent experiments will be required to statistically validate the transcriptomic landscape revealed by the in vivo models. We therefore plan to pursue further studies in this direction, which will be reported in a future publication.

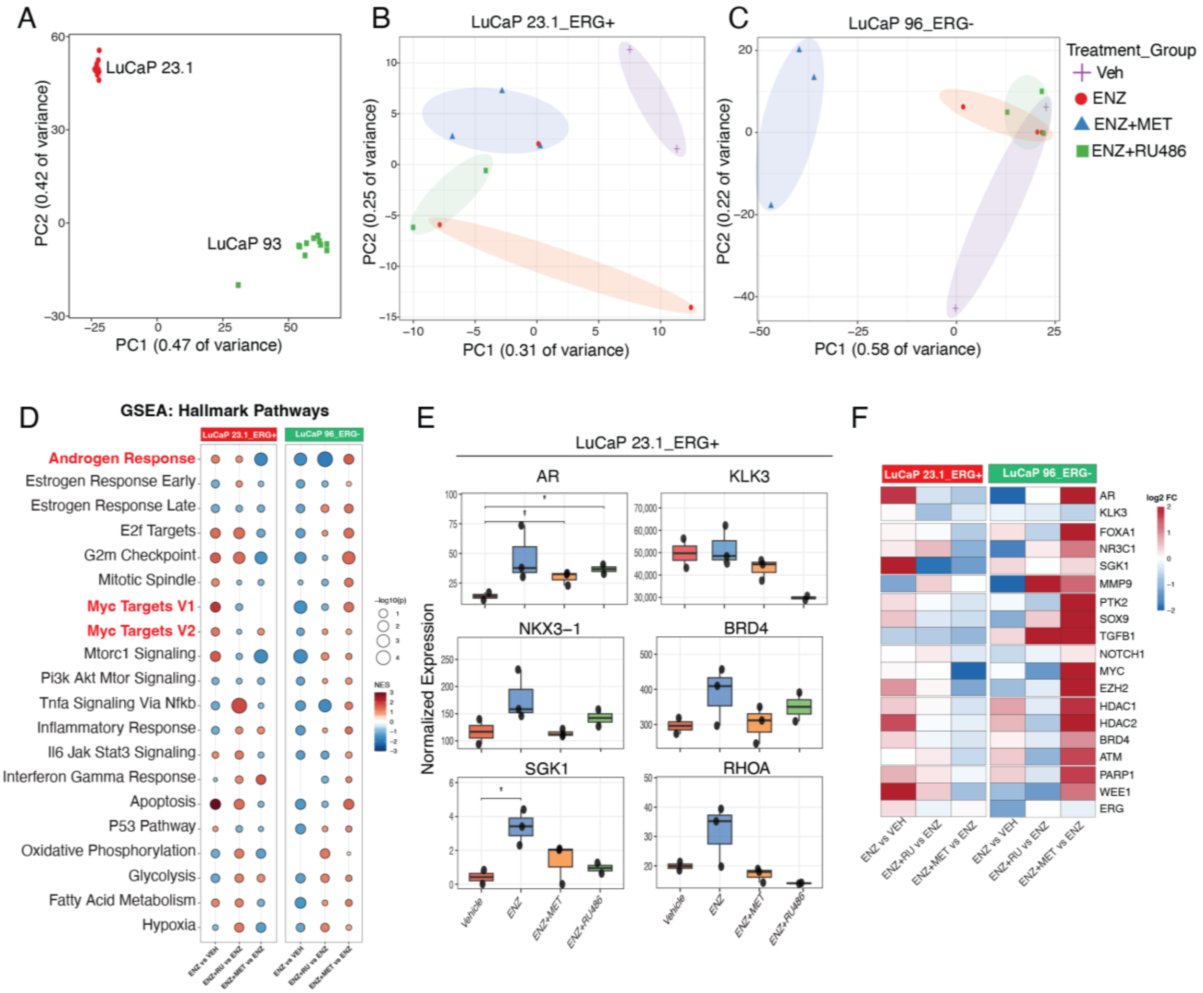

Figure VII: Global transcriptomic analysis of PDX models validates GR-tERG-mediated resistance mechanisms in the tERG-positive PCa model. RNA-sequencing was performed on tumors from one ERG+ (LuCaP 23.1) and one ERG- (LuCaP 96) PDX models to validate the proposed working model in vivo. (**A**) Principal component analysis (PCA) of RNA-seq data from LuCaP 23.1 ($tERG^+$) and LuCaP 96 ($tERG^-$) PDX tumors demonstrates distinct transcriptional programs between the two models, with PC1 capturing 47% of variance. (**B** and **C**) PCA of treatment groups within LuCaP 23.1 (B) and LuCaP 96 tumors (C). The treatment groups were as follows: Vehicle (Veh), Enzalutamide (ENZ), ENZ+Mifepristone (ENZ+MET), and ENZ+RU486. (**D**) Gene Set Enrichment Analysis (GSEA) of Hallmark pathways comparing treatment conditions in both models. Pathways analyzed include hormone signaling (Androgen Response, Estrogen Response Early/Late), cell cycle regulation (E2F Targets, G2M Checkpoint, Mitotic Spindle), MYC targets (V1, canonical targets and V2, indirect targets), growth signaling (mTORC1, PI3K-AKT-mTOR), inflammatory responses (TNFα via NFκB, IL6-JAK-STAT3, Interferon Gamma Response and Inflammatory Response), stress/survival pathways (Apoptosis, P53 Pathway), and metabolic programs (Oxidative Phosphorylation, Glycolysis, Fatty Acid Metabolism, Hypoxia). (**E**) Shown are normalized expression levels of AR target genes (AR, KLK3) and GR/ERG-regulated genes (NKX3-1, SGK1, BRD4, RHOA) across treatment conditions in LuCaP 23.1_ERG+ tumors. (**F**) Shown is a heatmap comparing expression of AR, GR, and ERG target genes between the two animal models and across all treatment conditions.

(ii) We agree that both human-derived xenograft models and genetically engineered mouse models of prostate cancer provide valuable and complementary insights. In principle, we could include immunocompetent syngeneic models to evaluate how systemic GR inhibition affects immune function. Such experiments would indeed strengthen the translational significance of the study, particularly regarding the safety of combining GR blockade with ADT. Nonetheless, direct extrapolation between species is constrained by major anatomical, developmental, genetic, and immunological differences. Specifically, murine prostates do not fully recapitulate the genetic heterogeneity of human tumors and they differ in stromal composition, developmental dynamics, and the tendency for spontaneous tumor formation or bone metastasis. To address the reviewer's concern, we have now added several relevant statements to the Discussion and the Limitations sections of the revised manuscript.

Referee #2 (Remarks for Author):

The article and the proposed research focus on the role of glucocorticoids in the development of resistance to anti-androgen therapy in prostate cancer. The authors initially investigate the overexpression of ERG, which commonly occurs following chromosomal rearrangements leading to the TMPRSS2-ERG fusion. ERG overexpression in patients is associated with resistance to androgen deprivation therapy. An interaction between the glucocorticoid receptor (GR) and ERG is reported, and this interaction can be modulated by GR agonists and antagonists. This crosstalk regulates ERG stability through ubiquitination and proteasome-mediated degradation processes and also influences its transcription factor activity. The GR-ERG interaction modulates signaling pathways involved in cell growth and survival. In vivo, the combined blockade of GR and AR using antagonists shows selective efficacy, specifically in PDX models exhibiting ERG overexpression. Overall, the paper is well written, and the experimental findings effectively support the conclusions.
However, some points still require clarification:
Major Points

1. Fig. 2A, 2F: Please include vehicle histograms with error bars and corresponding statistical tests. Fig. 2B: Dex treatment appears to alter ERG localization within the nucleus. The authors should elaborate and provide hypotheses to explain this observation. The rationale for using HeLa cells instead of human prostatic cell lines is unclear-"low signal to noise" is insufficient. Please provide more details (e.g., levels of ERG and GR accumulation, which prostate cell lines were tested, etc.). These results should be included in the supplementary material.

(i) As requested, we changed both panels. The revised forms include now horizontal lines representing vehicle control (solvent alone), error bars and the corresponding statistical parameters (see Figure VIII, below).

(ii) As requested, we performed Proximity Ligation Assay (PLA) experiments in VCaP prostate cancer cells, which endogenously express ERG. These results are presented in Figure IIA (see Referee 1, Comment 2).

(iii) We assume that DEX treatment alters ERG‘s nuclear distribution due to the previously reported entry into nuclear lipid microdomains and localized sphingomyelin metabolism (Cataldi *et al*, 2014).

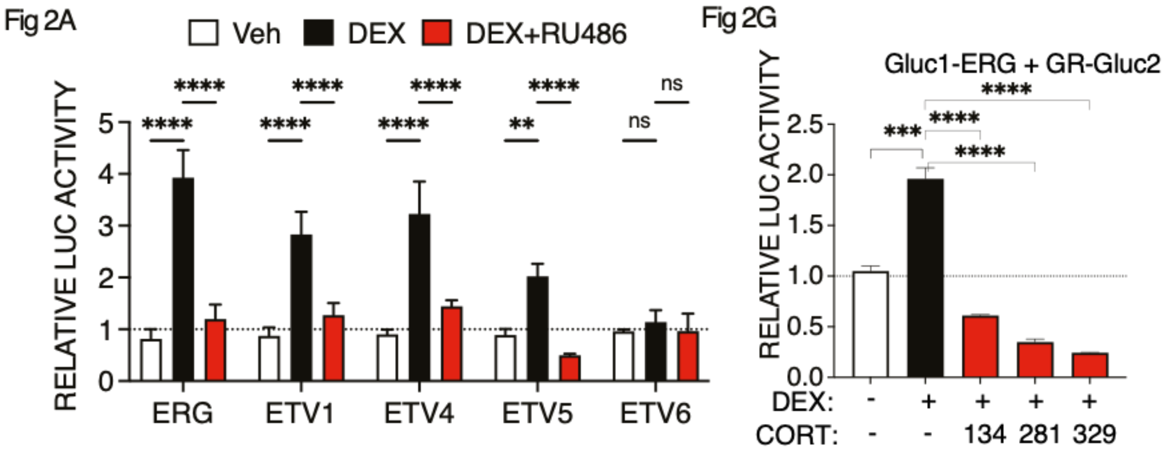

Figure VIII: The physical interaction between GR and ERG takes place in the nucleus and can be disrupted by a GR antagonist. (**left panel**) HEK293T cells growing in charcoal-stripped serum (CSS) were transfected (in sextuplicates) with GR-Gluc2, along with the indicated Gluc1-ETS plasmids. Twenty-four hours post transfection, cells were treated with vehicle, DEX (1 µM), or a combination of DEX and RU486 (1 µM). Shown are normalized fold changes in luminescence. ***, $p < 0.001$; ****, $p < 0.0001$; ns, not significant. Note that ETV6 was used as a negative control. The horizontal line shows the signal obtained in the presence of the vehicle only. (**right panel**) Serum-starved VCaP cells were treated for 60 minutes with DEX (1 µM) in the absence or presence of the following selective GR modulators (SGRMs, 10 µM): CORT125134 (C134, relacorilant), CORT125281 (C281, exicorilant) and CORT125329 (C329). Luminescence was determined in biological triplicates. All experiments were repeated 3 times. ***, $p < 0.001$.

2. Fig. 2D: The authors should perform a reciprocal immunoprecipitation using an anti-GR antibody to confirm the interaction between GR and ERG.

To confirm the interaction between GR and ERG, we performed reciprocal immunoprecipitation experiments using an anti-GR antibody and DU145-ERG cells. In addition, we conducted in vitro pull-down assays using purified recombinant proteins. Briefly, Dynabeads were incubated overnight with an anti-ERG antibody, washed, and then incubated with a recombinant ERG protein (10 µg/ml). The ERG-coated beads were subsequently incubated with purified ERG and GR proteins (each at 10 µg/ml) in the presence of DEX (1 µM), RU486 (10 µM), or prednisone (10 µM). These experiments demonstrate that DEX promotes the formation of a protein complex containing ERG and GR proteins (in vitro; see Fig. IX).

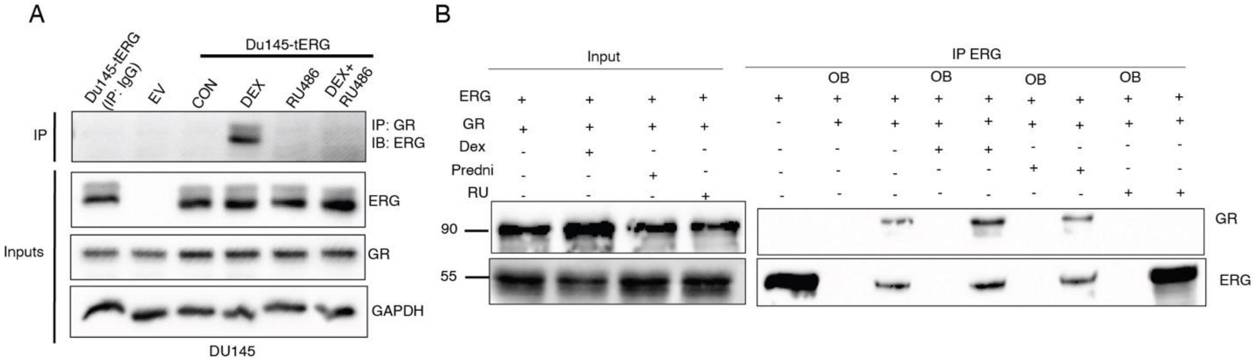

Figure IX: DEX promotes formation of a protein complex containing isolated ERG and GR proteins. (**A**) Serum-starved DU145-EV and ERG-overexpressing cells were treated for 60 minutes with vehicle, DEX (1 µM), RU486 (1 µM), or the combination of drugs. Extracts were processed for co-IP and IB. IgG, control antibody. (**B**). In vitro, DEX promotes formation of a protein complex containing purified recombinant ERG and GR proteins. Dynabeads were incubated overnight with an anti-ERG antibody and washed three times on the next day. The ERG-coated beads were incubated with a recombinant ERG protein at a concentration of 10 µg/ml for 4 hours. Next, the beads were washed thrice and equally distributed into a previously incubated (one hour) mixture of ERG and GR (each at 10 µg/ml), along with DEX (1 µM), RU486 (10 µM) or prednisone (10 µM). Only beads (OB) were used as control. *Input* refers to the mixture of the ERG and GR proteins.

3. The authors used multiple cell models to investigate ERG-GR interaction and crosstalk, including homologous models (VCaP, DU145, PC3, RWPE) and heterologous ones (HeLa, HEK293). Figure S5A, which summarizes the protein accumulation levels of interest, including western blots for ETV1, ETV4, and ETV5, should be moved earlier in the manuscript or at least in the supplementary figures. This information could be useful to justify the choice of specific cell types throughout the study.

As requested, we moved panel S5A to supplementary Figure S2. The revised form of Figure S2 (now called Figure EV2), including the new panel B, is shown below (Figure X of the rebuttal letter).

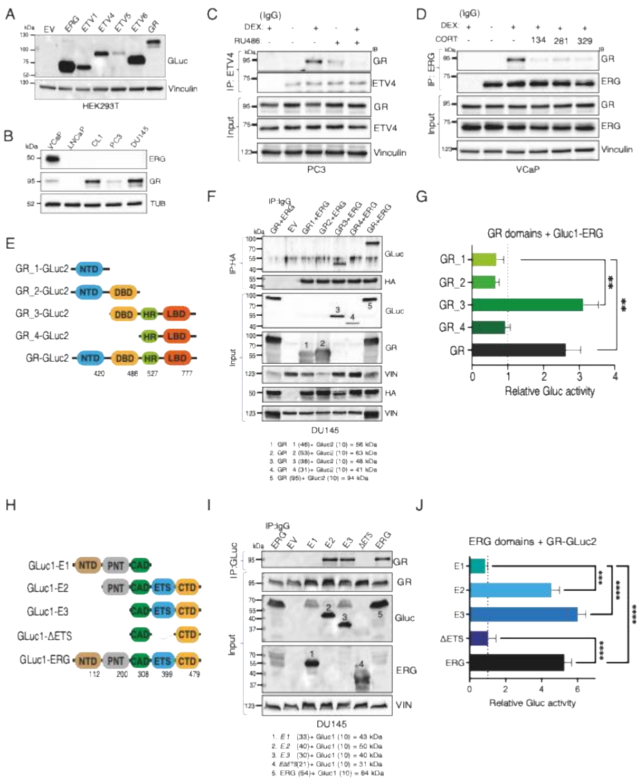

Figure X: Shown is the revised form of Supplementary Figure S2 (now, Figure EV2)**.**

4. Fig. 5E and 5F: The authors explored the molecular signature associated with RU486 treatment and proposed several ERG targets potentially responsible for the observed apoptotic phenotype. Knockdown or overexpression experiments of these candidate targets should be performed to identify downstream pathways that significantly contribute to GR antagonism-induced cell death.

MYC is a prominent direct transcriptional target of TMPRSS2-ERG in prostate cancer, contributing to its oncogenic effects alongside other effectors (Sun *et al*, 2008). Hence, to functionally validate downstream mediators of GR antagonism–induced cell death, we focused on this master transcription factor. Firstly, we utilized siRNA oligonucleotides, either control or MYC-specific siRNAs, and confirmed efficient MYC knockdown in DU145-ERG prostate cancer cells (Fig. XI, left panel). Next, we assessed cell death using an LDH release assay. Importantly, MYC silencing significantly sensitized DU145-ERG cells to RU486-induced cytotoxicity (Fig. XI, right panel), demonstrating that MYC contributes to the apoptosis mediated by GR antagonism.

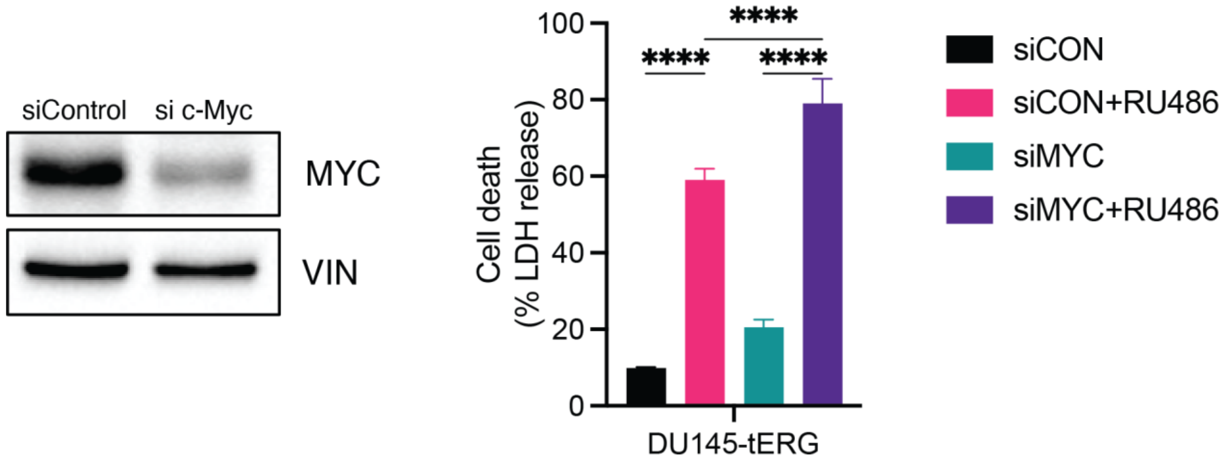

Figure XI: MYC knockdown sensitizes DU145-ERG prostate cancer cells to RU486-induced cell death. (**left panel**) Immunoblot analysis showing efficient siRNA-mediated knockdown of MYC in DU145-ERG cells. Cells were transfected with control siRNA or siMYC, and protein levels were analyzed using Western blotting. (**right panel**) The lactate dehydrogenase (LDH) release assay was employed when measuring cell death in DU145-ERG cells that were transfected with control siRNA or siMYC, as well as treated with RU486. Note that MYC knockdown significantly enhanced RU486-induced cytotoxicity. Data represent mean ± SEM from three independent experiments. ****, $p < 0.0001$.

5. Fig. 7: Histological analysis or gene expression profiling of tumor xenografts should be included to assess proliferation (e.g., Ki67 staining, CCND1 expression), anti-androgen treatment efficacy (expression of AR target genes), as well as representative images from necropsy (H&E staining or tumor pictures). The number of mice per group (4 to 6, as stated in the legend) is low for a survival study. Additionally, in some Kaplan-Meier curves, fewer than four animals appear to be present-especially in the Enzalutamide groups. Please clarify this and/or increase sample size.

Histological and molecular analysis: Unfortunately, paraffin-embedded tissue blocks from our PDX models were lost following the June 15 (2025) attack on Israel. However, frozen tumor tissues from all models remained available. For VCaP and DU145 xenografts (Figure 6), we have included H&E staining (Fig. XII-A) and Ki67 immunohistochemistry (Fig. XII-B). H&E staining of VCaP xenografts showed dense tumor cellularity in vehicle-treated mice, with visibly reduced tumor cell density in the RU486 and metyrapone-treated groups. DU145 xenografts (tERG-negative) showed comparable tumor cellularity across vehicle, RU486, and metyrapone groups, consistent with GR inhibition selectively affecting tERG-positive tumors. Ki67 staining of VCaP xenografts demonstrated high proliferative signal in vehicle-treated tumors, but reduced Ki67 staining in the RU486- and metyrapone-treated groups.

Clarification on Kaplan-Meier curves and sample size: We appreciate the opportunity to clarify the animal numbers. The apparent reduction in animal numbers on the Kaplan-Meier curves does not reflect dropout or exclusion. Per IACUC guidelines, mice were sacrificed upon reaching the maximum allowable tumor volume of 1500 mm^3, which occurred at different timepoints across treatment groups. This is noted in the figure legend and methods section. PDX models exhibit

variable engraftment and growth kinetics, precluding representative tumor photographs at uniform timepoints.

<u>GR-knockout xenografts demonstrate that GR is essential for tumor growth:</u> To further demonstrate the functional requirement for GR in tERG-positive tumor growth, we performed xenograft studies using VCaP wild-type and two independent GR-knockout clones (Figs. XIIC through XI-E). VCaP wild-type cells formed tumors in 5/5 mice, as expected. In contrast, GR-KO#1 formed relatively small tumors in only 3/6 mice, and GR-KO#2 formed tumors in only 1/6 mice. These results provide compelling in vivo evidence that GR is essential for tERG-positive tumor growth, supporting the therapeutic rationale for GR inhibition.

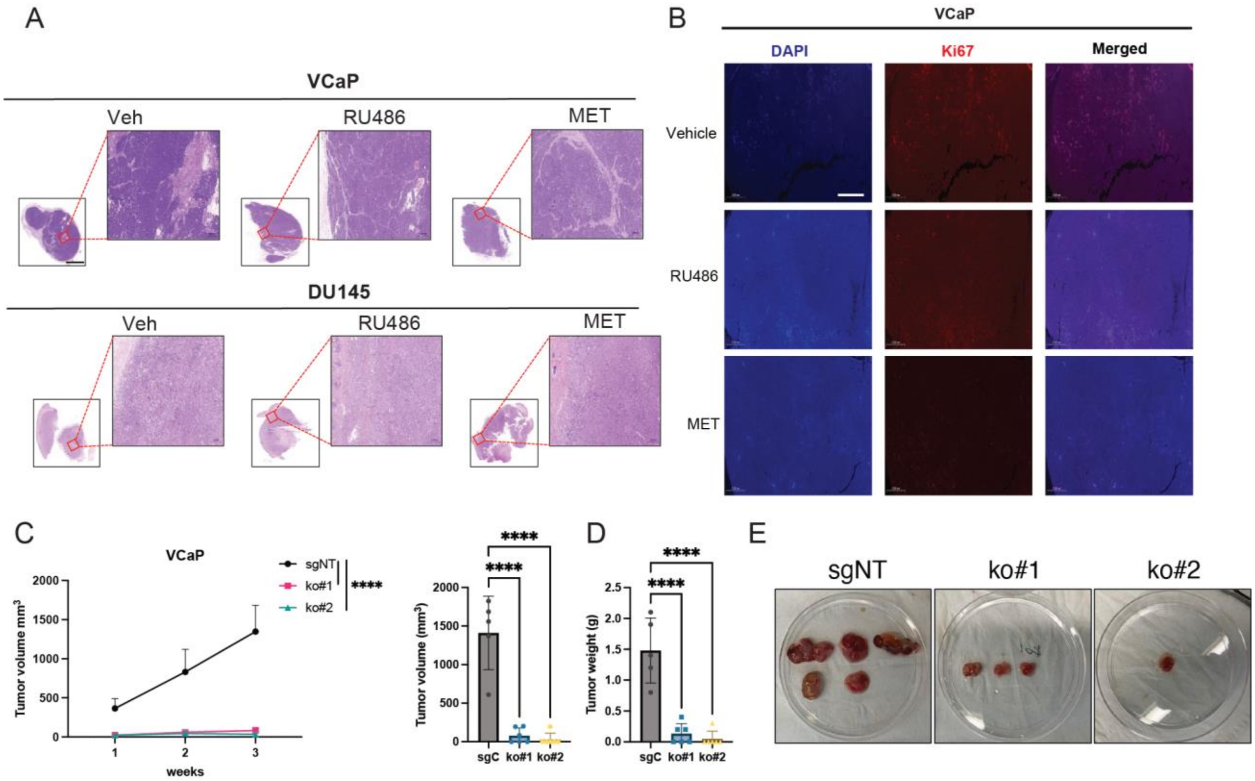

Figure XII: Pharmacological and genetic disruption of GR selectively inhibits tERG-positive tumor growth. (**A**) VCaP (upper panels) and DU145 (lower panels) cells were implanted subcutaneously in athymic mice. Once tumors became palpable, animals were randomized and treated daily with vehicle, RU486 (1 mg/kg), or metyrapone (25 mg/kg; n=5-6 mice per group). Shown are H&E staining of tumor sections. Representative images are shown at low magnification (scale bar, 5 mm) and higher magnification (10X; scale bar, 200 µm). (**B**) Ki67 immunohistochemistry of VCaP xenograft tumors. Representative images are shown at 5X magnification (scale bar, 500 µm). (**C**-**E**) VCaP cells (2×10^6) expressing sgNT (wild-type), GR-KO#1, or GR-KO#2 were implanted subcutaneously in athymic mice. Tumor volume (C), weight at endpoint (D), and representative tumor images (E) are shown. ****, $p < 0.0001$.

Minor Points

• Page 7, line 3, line 18, etc.: Correct spelling of "Previously."

The spelling error was corrected in the revised manuscript.

• Fig. 1D: "Probability of survival" is not appropriate; please use "Overall survival." Also, Fig. 1D is not referenced in the main text.

As requested, we corrected Figure 1D (see Figure XIII, below). Note that "Overall Survival" replaces "Probability of Survival". In addition, we included below the respective legend and the corresponding part of "Results", which was inserted in the revised manuscript.

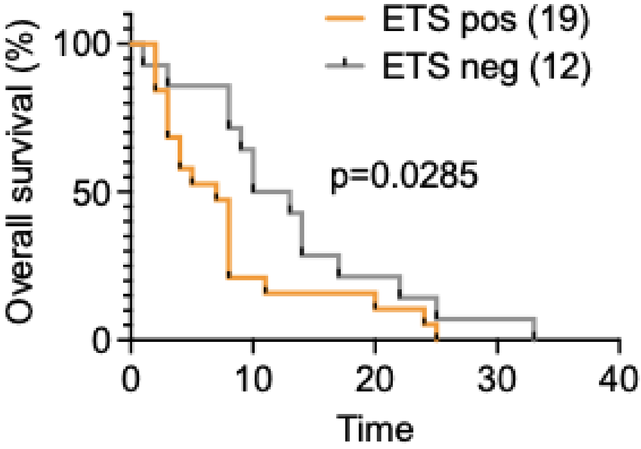

Figure XIII: Revised Figure 1D: Data were derived from the control arm of the NCT01576172 (NCI 9012) clinical trial. All patients included in this control arm (n=31) received ADT plus abiraterone acetate/prednisone for their mCRPC. Biopsy samples were obtained from metastatic sites and analyzed for GR and ERG using RNA-seq and IHC. Shown are Kaplan-Meier progression-free survival curves corresponding to the group of ETS-positive patients (n=19) and the ETS-negative group (n=12) of patients (p=0.0285; Gehan-Breslow-Wilcoxon two-sided test).

Revised text of Results corresponding to Figure 1D: Next, we asked if the correlation between tERG expression and lack of response to anti-androgen treatment extends to metastatic castration-resistant prostate cancer (mCRPC). For this, we analyzed the results of another clinical trial, NCT01576172 (NCI 9012)[1]. All patients of this trial underwent metastatic site biopsy, stratified by the status of ERG and ETV1 (using IHC and in situ hybridization, respectively) and randomly assigned to abiraterone plus prednisone, either without (arm A) or with an inhibitor of poly(ADP-ribose) polymerase-1 (PARP1), veliparib (arm B). We focused on a subset of arm A patients (N=31) for whom survival data were available. These patients presented samples with matching RNA-seq, GR and ERG immunohistochemistry, and their PSA responses were available. A summary Kaplan-Meier survival analysis is shown in Figure 1D. As predicted, the median progression free survival (PFS) of the *tERG*-negative group (11.5 months) was significantly longer (p=0.0285; Gehan-Breslow-Wilcoxon test) than the PFS rate of the *tERG*-positive cohort (7.0 months).

• Page 7, line 5: The working model is in Fig. 1E, not Fig. 1D. The working model should be presented at the end of the article or as a graphical abstract, in accordance with EMM recommendations.

As requested, in due time we will consult the editors of EMM regarding the best location of panel E.

• The meaning of "CORT" must be clarified in the legends for Fig. S2C and Fig. 2F. Overall, the naming of CORT-related agents must be standardized across the manuscript text, figure legends, and figures.
As requested, the revised text refers to the family of CORT inhibitors developed by Corcept Therapeutics (Menlo Park, California). These are selective non-steroidal drugs that antagonize GR and used to treat disorders of cortisol excess, without the full steroid-like side effects of traditional corticosteroids. For example, Relacorilant is a highly selective GR modulator with no PR activity.

• Fig. S2E: According to the text, ERG is tagged with HA. Please include this detail in the figure legend.
The legend to Figure 2E has been revised, such that it refers to the HA-tagged ERG proteins we used.

• Page 9, line 24: "Slightly stronger interaction"-if the difference between the GR_3 construct and full-length GR is not statistically significant, this statement should be removed.
As requested, we removed this statement

• Fig. 3C: Please clarify the meaning of histogram colors in the figure legend.
• Page 10, line 15: "Inconsistent changes in the abundance of mRNAs encoding tERG" seems contradictory with the figure, which shows a significant increase from 0 h to 72 h. Please revise the text for clarity.
In response to these comments, we removed the colors from panel C of Figure 3 and revised the corresponding text. The new text highlights the abilities of enzalutamide and RU486 to respectively reduce and elevate ERG's transcript levels, unlike DEX which did not alter transcription of ERG.

• Fig. 4E: Revise the legend to clearly distinguish between solid and dashed lines.
As requested, we revised Figure 4E in a way that made use of both solid and dotted lines that are clearly distinguishable. See Figure XIV below.

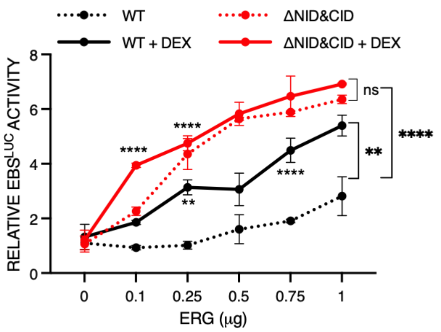

Figure XIV: By relieving allosteric autoinhibition, GR enhances the transcriptional function of ERG. HEK293T cells grown in medium supplemented with charcoal-stripped serum (10%) were co-transfected with an EBS-luciferase reporter, as well as with the WT or mutant ERG plasmids at increasing doses. Sixteen hours post

transfection, cells were stimulated with DEX (1 µM). Luciferase activity was measured using a commercial kit (from Promega).

• Fig. 7: Standardize the size of curves and legends. Is there a reason why the MET condition is absent in panel 7C? Please indicate which curves were compared to calculate the log-rank statistics.

In response to this comment of Referee 2 we present below the results of one of our pilot experiments that used RU486 and Metyrapone, both delivered as monotherapies. The results shown in Figure XV indicate that neither drug caused detectable tumor shrinkage. For this reason, the single-agent experimental arms were not included in the in vivo setup presented in the original Figure 7C.

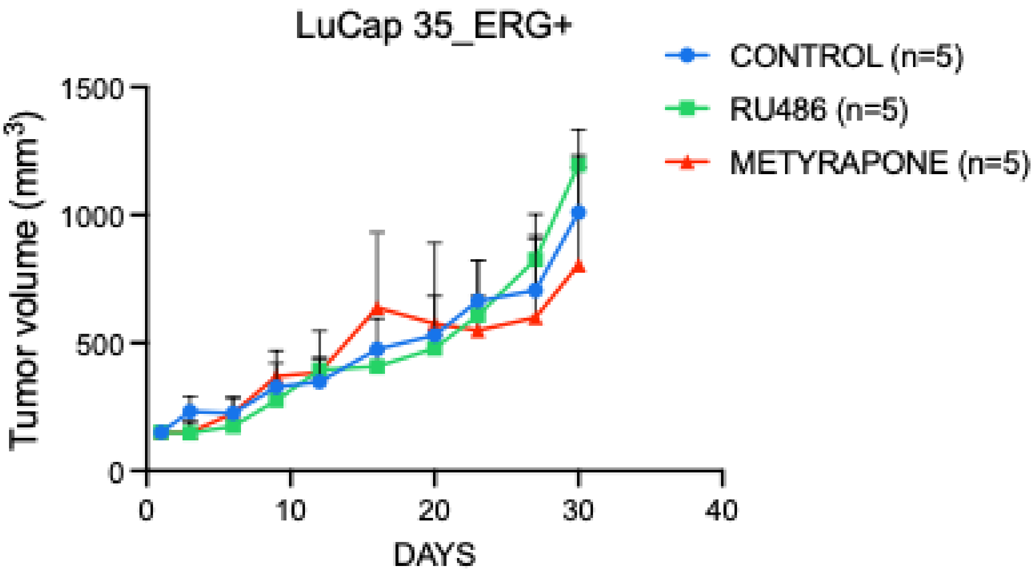

Figure XV: When singly administered, neither RU486 nor MET (metyrapone) caused detectable changes in the tumorigenic growth rate of LuCAP35_ERG$^+$. Fragments of the tERG-positive LuCaP 23.1 patient-derived xenograft (PDX) model were subcutaneously implanted in the flanks of NSG mice. Once tumors reached 150 mm^3, animals were randomized into the indicated groups (5 animals per group), which were daily treated with RU486 (1 mg/kg, intraperitoneal injection) or MET (25 mg/kg, oral gavage). Tumor volumes were monitored twice per week. Average tumor volumes are indicated, along with the corresponding standard deviations.

• Overall, the statistical tests used should be specified in the figure legends, even if they are partially described in the Methods section.

As requested, we reviewed all figures and, where appropriate, indicated the statistical tests within the data figures.

Referee #3 (Remarks for Author):

The TMPRSS2-ERG fusion gene (tERG) is frequently discovered in prostate cancer patients, which leads to the androgen-inducible overexpression of ERG protein. Although a high level of tERG has been reported to indicate high Gleason scores and more advanced disease, whether tERG confers antiandrogen resistance in prostate cancer is unknown. Emerging evidence suggests that GR overexpression and sustained glucocorticoid could take over androgen-AR's role in promoting prostate cancer progression under the enzalutamide treatment. Because GR physically interacts with other ETS family members, as suggested by the author's previous study, the author proposed to investigate whether TMPRSS2-ERG confers resistance to antiandrogens via direct binding to GR. By analyzing clinical trial data, the study found that patients with tERG-positive fusions have worse outcomes with antiandrogen treatment. Mechanistically, this work revealed that GR binding stabilizes ERG protein, then strengthens ERG's transcriptional activity. Furthermore, the xenograft studies demonstrated that tERG-positive models are more sensitive to a combination of GR and AR inhibitors compared to tERG-negative models. Overall, this is a good study that focuses on enzalutamide-resistant prostate cancer. It has the potential to develop tERG as a biomarker to predict the response of prostate cancer patients to antiandrogen treatment and select the patients who are highly sensitive to the combination treatment of AR and GR inhibitors. The manuscript is well written and referenced. However, there are several major and minor points that need to be addressed by the authors.

Major points:

1. The data for the matched pre- and post-treatment samples should be analyzed and provided to confirm that enzalutamide can increase the transcriptional and protein levels of ERG and GR in nonresponders.

To address this comment, we reanalyzed data from a clinical trial involving 37 men with metastatic castration-resistant prostate cancer (mCRPC), in which matched pre- and post-treatment biopsies were subjected to immunohistochemistry (IHC), whole-exome sequencing, and RNA sequencing. Patients received androgen deprivation therapy (ADT) combined with enzalutamide for six months (Karzai *et al*, 2021; Wilkinson *et al*, 2021). Among the 17 ERG-positive patients, 14 were classified as non-responders, whereas among the 20 ERG-negative patients, 7 were non-responders. Our reanalysis (Figure XVI) confirmed post-treatment upregulation of GR at both the mRNA and protein levels and post-treatment downregulation of ERG at the mRNA level. Consistent with the referee's expectation, anti-androgen treatment was associated with increased GR transcript and protein expression specifically in non-responders.

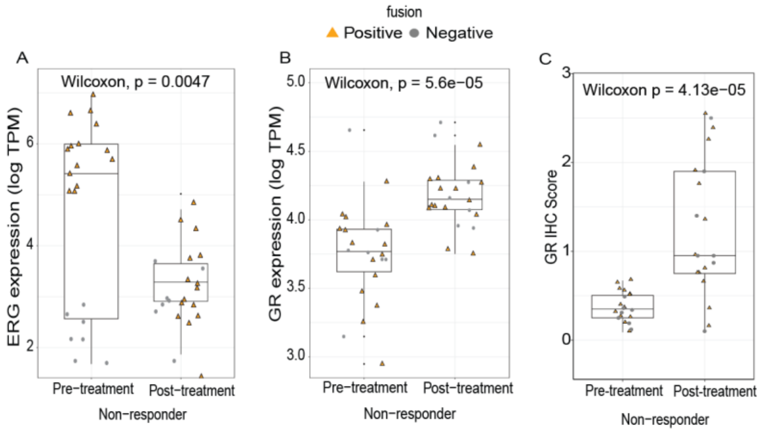

Figure XVI: Analysis of ERG and GR expression in non-responders to antiandrogen therapy. Matched pre- and post-treatment tumor samples were analyzed for ERG and GR expression. (**A**) ERG mRNA levels and (**B**) GR mRNA levels in non-responders before and after treatment. (**C**) GR protein expression assessed by immunohistochemistry (IHC) scoring in matched pre- and post-treatment samples from responder and non-responder cohorts. Paired comparisons between pre- and post-treatment samples were performed using the Wilcoxon signed-rank exact test. Note that p-values are indicated in the figure.

2. The data supporting the induction of ERG levels by enzalutamide in cell models and xenograft models are missing. Although VCaP is tERG positive, it's enzalutamide sensitive. It's unclear if enzalutamide-resistant VCaP will have higher tERG and whether the tERG knockdown will resensitize the cells to enzalutamide. In the PDX models, it's unknown if enzalutamide induces tERG expression. Additional tERG-negative, AR-positive cell models (such as LNCaP, LNCaP/AR, LAPC4, etc.) should be involved to evaluate whether enzalutamide increases ERG expression levels when the cells develop enzalutamide resistance.

<u>(i) Dynamic regulation of ERG's abundance</u>: This comment relates to a thoroughly investigated issue. While the majority of reports and preclinical studies link ERG expression to the development of resistance to enzalutamide, the evidence is not straightforward, likely due to the dynamic nature of AR-regulated expression of TMPRSS2-ERG. For example, an important report demonstrated an expression decline with androgen withdrawal, but then the level of expression was markedly restored (Cai *et al*, 2009). Specifically, these authors demonstrated that both ERG encoding mRNA and ERG protein rapidly declined after androgen withdrawal in cultured VCaP cells, as well as in the respective xenografts, but this was followed by an adaptive restoration. Thus, dynamic longitudinal changes in ERG expression take place in response to AR inhibition and subsequent resistance.

<u>(ii) Answering the comment: "In the PDX models, it's unknown if enzalutamide induces tERG expression":</u> To answer this question, we analyzed tumors generated by the PCA1 PDX model shown in original Figure 7. Tumor tissues from the control and treated groups were processed in parallel for protein analysis. A sample of approximately 50 mg of tissue was washed with saline and homogenized. Lysates were cleared and protein concentrations were determined prior to gel electrophoresis. The results shown below (Fig. XVII) disclose no marked effect of enzalutamide on either ERG or GR, probably due to the ability of the PDX model to recruit from the host substantial fractions of stromal and immune cells.

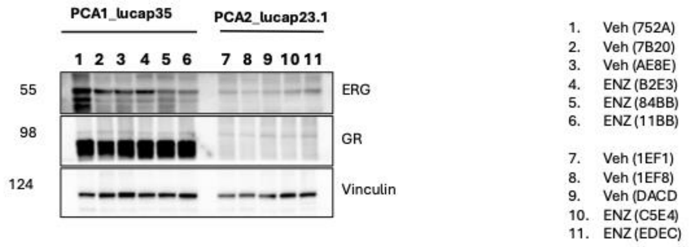

Figure XVII: Analysis of PDX models using immunoblots. Tumor tissues from control and treated groups of both PDX models were processed in parallel for protein analysis. Approximately 50 mg of tissue per sample was washed with saline, homogenized with a Dounce homogenizer, and centrifuged at 5,000 × g for 5 minutes at 4 °C. The resulting pellet was resuspended in RIPA lysis buffer supplemented with protease inhibitors ,and lysed by intermittent vortexing. Lysates were cleared by centrifugation at 15,000 × g for 15 min at 4°C. Protein concentrations were determined using a BCA assay, and equal amounts of protein were subjected to Western blot analysis. Vinculin was used as the loading control.

(iii) Answreing the comment "whether the tERG knockdown will resensitize the cells to enzalutamide". In response to this question and to the related text of comment 3 from this referee, we present new data and demonstrate that tERG knockdown can resensitize cells to enzalutamide. Please read our response to comment number 3 (below).

(iv) Insights from clinical data revealing GR-mediated resistance in tERG-positive patients: Note that our re-analysis of the clinical trial reported by Karzai et al (Karzai *et al.*, 2021) (original Figs. 1 and S1) directly addresses the reviewer's concern. Consistent with ERG being under control of the AR-responsive TMPRSS2 promoter, our data show that ERG's mRNA decreases in tERG-positive patients following enzalutamide + ADT treatment, specifically due to androgen blockade, yet these patients exhibit treatment resistance, suggesting that tERG phenotypes and post-translational mechanisms maintain tERG function despite transcriptional suppression. Notably, anti-androgen treatment significantly increased both transcriptional and protein levels of GR in non-responders (Figs. 1B-1C, $p < 0.05$, Wilcoxon signed-rank test). Among ERG-positive patients, 14 of 17 non-responders demonstrated high IHC signal corresponding to GR post-treatment. These observations highlight the strong association between ERG positivity, GR upregulation, and treatment resistance. In contrast, the same clinical data analysis found that responders display stable or reduced GR expression following treatment. These results demonstrate that treatment-associated upregulation of GR is specifically linked to therapeutic resistance in ERG-positive patients, supporting our mechanistic model of GR-mediated tERG protein stabilization.

(v) Enzalutamide treatment restores ERG protein levels through GR-dependent mechanisms: To directly address the reviewer's question about ERG dynamics during enzalutamide treatment, we performed Western blot analysis of VCaP wild-type and GR-knockout VCaP cells treated with enzalutamide over a 48-96 hours time course (Fig. XVIII). Consistent with published literature, ERG protein levels initially decreased at 48 hours following enzalutamide treatment. However, ERG protein was restored to baseline levels by 72-96 hours in wild-type cells, coinciding robust upregulation of GR across all timepoints. In contrast, GR-knockout cells maintained low ERG protein levels across all timepoints (48, 72, and 96 hours), demonstrating that GR is essential for ERG protein recovery during enzalutamide treatment. These results provide direct experimental evidence that GR mediates ERG protein stabilization during antiandrogen therapy.

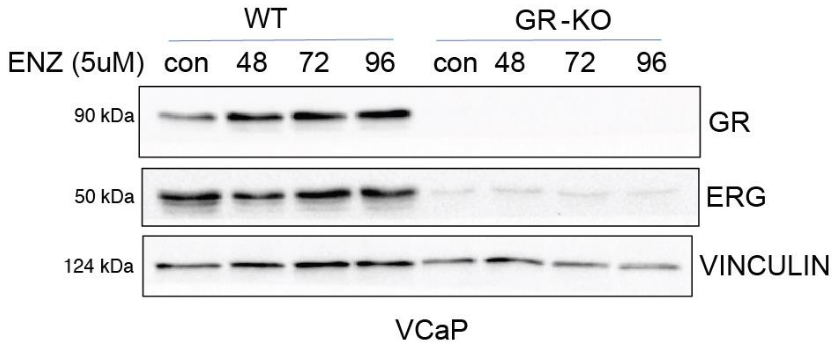

Figure XVIII: GR is required for ERG protein recovery during enzalutamide treatment. VCaP wild-type and GR-knockout cells were treated with enzalutamide (5 µM) for the indicated time intervals and whole cell extracts subjected to Western blot analysis for ERG, GR, and vinculin (loading control).

(vi) ERG induction in tERG-negative models: The reviewer suggested evaluating ERG expression in fusion-negative, AR-positive models (such as LNCaP). We respectfully note that *de novo* ERG induction in fusion-negative cells has limited clinical relevance because TMPRSS2-ERG is a genomic rearrangement involving chromosomal alterations rather than transcription regulation. In addition, published LNCaP resistance studies reported no ERG acquisition during enzalutamide resistance (Arora *et al*, 2013)

In summary, our re-analysis of clinical and preclinical experiments demonstrates that enzalutamide resistance in tERG-positive tumors operates through GR-mediated stabilization of the existing tERG protein rather than transcriptional induction, and this mechanism is specific to tumors harboring the TMPRSS2-ERG fusion.

3. Does ERG knockdown make the tERG cells become sensitive to enzalutamide?

We thank the reviewer for this important question. To test whether ERG contributes to enzalutamide tolerance, we performed ERG knockdown studies in VCaP cells. The cells were transfected with siRNA targeting ERG (siERG) or non-targeting control oligonucleotides, followed by treatment with enzalutamide (2 and 5 µM). Firstly, we performed Western blotting, which confirmed efficient ERG knockdown. Next, we carried out LDH cytotoxicity assays, which demonstrated significantly increased enzalutamide-induced death of siERG-treated cells, indicating that ERG depletion enhances enzalutamide-induced cytotoxicity. While these experiments were performed in parental VCaP cells rather than enzalutamide-resistant derivatives, they demonstrate that ERG functionally protects tERG-positive cells from enzalutamide-induced cell death. Combined with our GR-knockout data showing that GR is essential for ERG protein recovery during enzalutamide treatment, these results support a model wherein targeting the GR-ERG axis can enhance therapeutic response in tERG-positive prostate cancer.

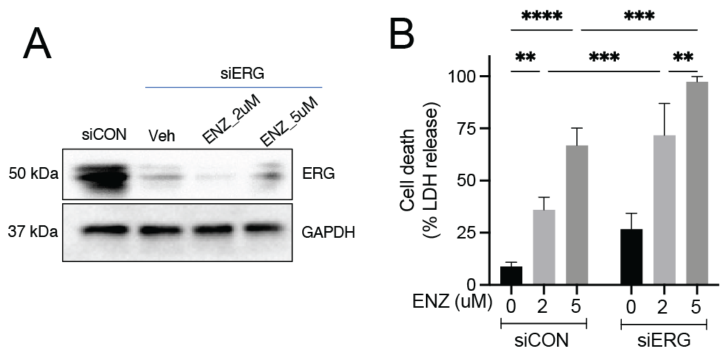

Figure XIX: ERG knockdown enhances enzalutamide-induced cytotoxicity in VCaP cells. (**A**) VCaP cells pre-transfected with siERG or siControl were treated with enzalutamide (2 and 5 μM) for 48 hours, and subjected to Western blot analysis for ERG and GAPDH. (**B**) LDH cytotoxicity assays measuring cell death in siControl and siERG-transfected VCaP cells treated with enzalutamide. **, p < 0.01; ***, p < 0.001.

4. Does reinstating the tERG into tERG-negative, AR-positive cell models (such as LNCaP, LNCaP/AR, LAPC4, ETC.) cause the cells to become insensitive to enzalutamide resistance?

To determine whether ectopic expression of tERG can confer resistance to enzalutamide, we performed ERG overexpression studies in 22Rv1 and CL-1 cells, which are both tERG-negative/AR-positive. Both lines were pre-transfected with either a tERG expression vector or an empty vector (EV; control), then treated with enzalutamide, RU486 or the combination of drugs. Control immunoblots confirmed tERG overexpression in the transfected cells. Importantly, enzalutamide reduced 22RV1 cell viability, but RU486 had no effect on cell survival, and enzalutamide plus RU486 showed enhanced effect only in tERG-expressing cells (see Figure XX, below). These results demonstrate that de novo tERG expression confers vulnerability to GR inhibition.

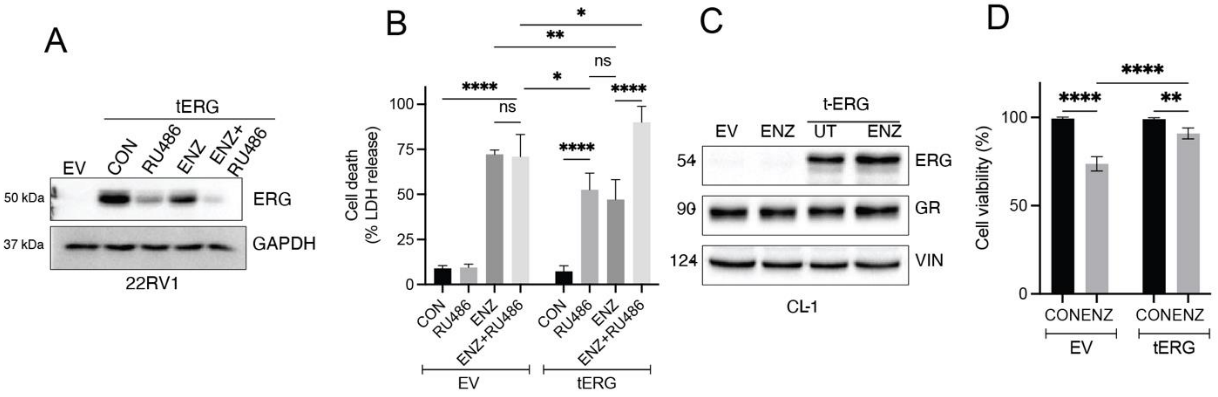

Figure XX: tERG overexpression confers GR-dependent enzalutamide resistance. (**A** and **B**) 22Rv1 cells were transfected with either a tERG expression vector or an empty vector (EV). Thereafter, the cells were incubated for 24 hours with enzalutamide, RU486, or the combination of the two drugs. Whole cell extracts were subjected to Western blot analysis (A). In addition, live cells were subjected to the LDH cytotoxicity assay (B). (**C** and **D**) CL-1 cells were transfected with either a tERG expression vector or an empty vector (EV). Thereafter, the cells were incubated for 24 hours with enzalutamide. Whole cell extracts were subjected to Western blot analysis (C). In addition, live cells were subjected to the MTT cytotoxicity assay (D).

5. Does GR overexpression rescue the ERG protein loss induced by RU486 and GA?
To address this question, we performed GR overexpression experiments using two cellular models and presented the results below. The following cellular systems were employed (Fig. XXI):
(i) VCaP cells with CRISPR-mediated GR knockout were transfected with a GR expression construct and treated with RU486 (10 µM) for 48 hours. Immunoblot analysis demonstrates that ectopic GR expression could rescue ERG protein levels in the presence of RU486. This confirms that the RU486-induced ERG degradation is specifically mediated through GR inhibition and can be reversed by restoring GR expression. Note that the results of this experiment are shown if Figure EV3F of the revised manuscript).
(ii) DU145 cells stably expressing tERG were transfected with a GR plasmid and treated with either RU486 (10 µM) or geldanamycin (GA, 200 nM) for 48 hours. Immunoblot analysis showed that GR overexpression can rescue ERG protein levels that would otherwise be degraded by both RU486 and GA treatment. This demonstrates that increasing GR levels can protect ERG from degradation induced by pharmacological disruption of GR function, either through direct antagonism (using RU486) or Hsp90 inhibition-mediated GR destabilization (using geldanamycin).

In conclusion, these complementary loss-of-function and gain-of-function approaches established that GR levels directly determine ERG protein stability, confirming that GR functionally maintains ERG abundance in prostate cancer cells.

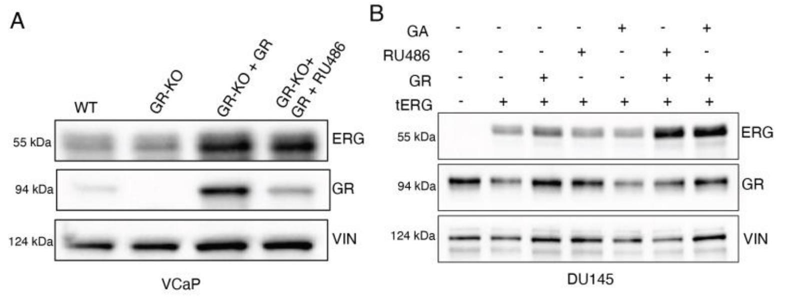

Figure XXI: GR overexpression rescues ERG protein loss induced by a GR antagonist. (**A**) GR-knockout VCaP cells were transfected with a GR expression vector and treated with RU486 (10 µM) for 48 hours. Whole cell extracts were subjected to immunoblotting with antibodies against ERG, GR, and vinculin (loading control). (**B**) DU145 cells stably overexpressing tERG were transfected with a GR expression vector or with an empty vector and treated with either RU486 (10 µM) or GA (0.2 µM) for 48 hours. Whole cell extracts were subjected to immunoblotting with antibodies against ERG, GR, and vinculin (as loading control).

6. Does ERG overexpression rescue the GR inhibitor-suppressed cell and xenograft growth?
Below we examine this point using DU145-tERG cells both in vitro and in vivo. The DU145 cell line, which lacks endogenous ERG expression, was engineered to stably express tERG and used directly to address the reviewer's question by showing that ERG overexpression confers sensitivity to GR inhibition.

(i) **In vitro tests (Figures XXII-A and XXII-B):** DU145-tERG cells displayed significant growth suppression upon RU486 treatment (5 and 10 µM) in terms of colony formation assays, while the parental cells (empty vector control) displayed

resistance to RU486. Note that the results of this experiment are shown in Figure EV6F of the revised manuscript.

(ii) **In vivo tests (Figures XXII-C and XXII-D):** DU145-tERG xenografts responded significantly to both RU486 (1 mg/kg) and metyrapone (25 mg/kg) treatment, exhibiting substantial tumor suppression compared to the vehicle control. In contrast, control DU145-EV xenografts showed no response to GR inhibition.

Together, these gain-of-function experiments complemented our loss-of-function approach and established that ERG expression is a key determinant of response to GR-targeted therapy.

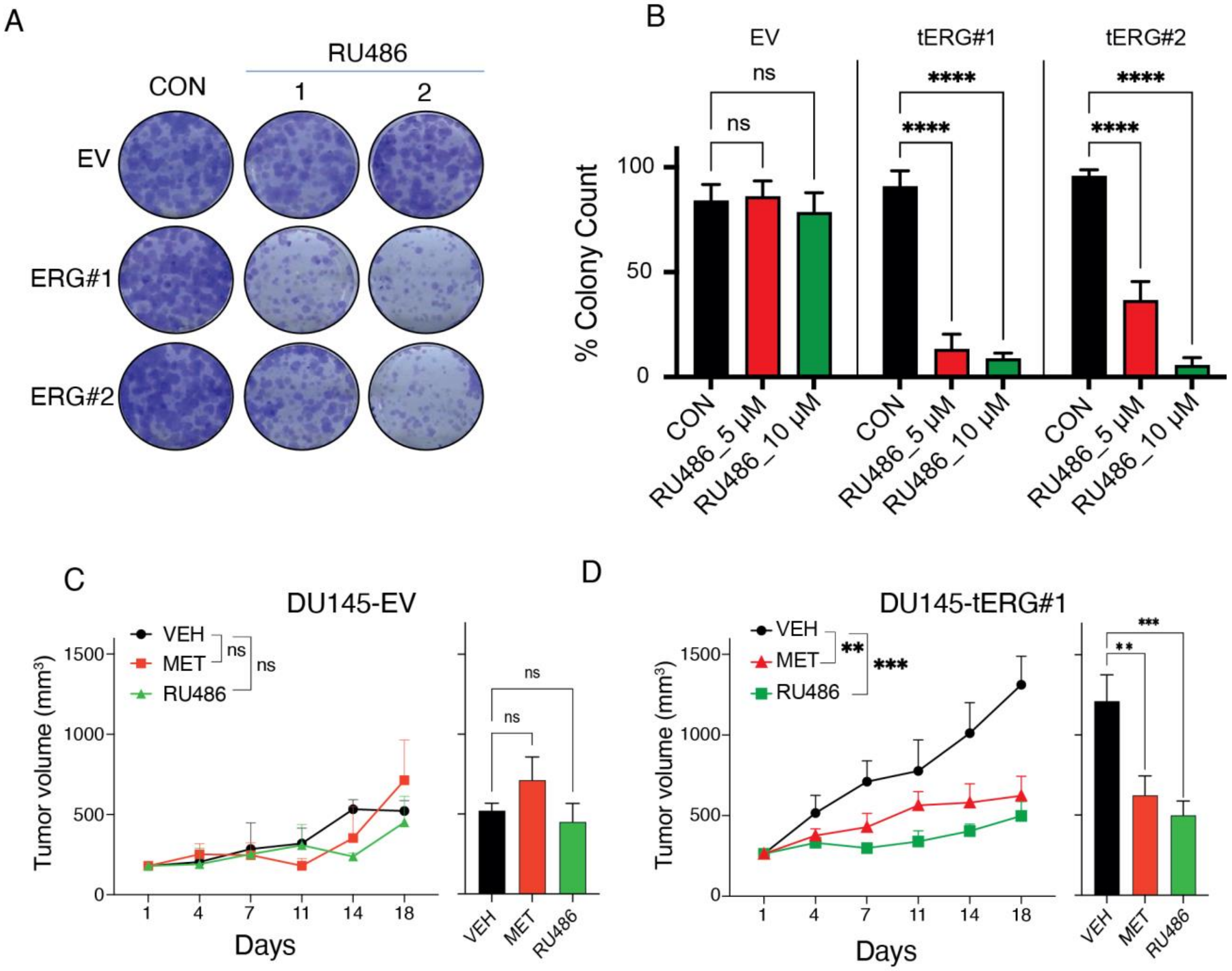

Figure XXII (original Figure S6): GR inhibition decreases growth of tERG-expressing PCa cells but cells expressing no ERG do not respond to the inhibitors. (**A** and **B**) Two clones of DU145 cells stably expressing tERG were sparsely seeded in 6-well plates. Cells were later treated once every other day with either vehicle or RU486 (5 µM and 10 µM). Ten days later, all cells were fixed and stained with crystal violet. Representative photos are shown, along with bar plots presenting the quantification of colony numbers in 5 non-overlapping microscope fields. (**C** and **D**) DU145 cells stably expressing tERG ($5x10^6$) and control DU145 cells (EV; $5x10^6$) were implanted subcutaneously in nude mice and, once tumors became palpable, animals were randomized into three groups, which were treated daily with vehicle, RU486 (1 mg/kg), or with metyrapone (25 mg/kg). The bar graphs present tumor volumes corresponding to day 18. **, p < 0.01; ***, p < 0.001; ****, p<0.0001; ns, not significant.

7. Does MET suppress the corticosterone levels in mouse serum and tumor tissues? And will DEX reverse the suppression of tumor growth induced by MET?

To determine whether MET affects glucocorticoid levels in vivo, we performed ELISA measurements of corticosterone in tumor tissues. These analyses revealed that MET treatment suppresses corticosterone levels within the tumor microenvironment, whereas no reduction was observed in vehicle- or RU486-treated tumors (see Fig. XXIII).

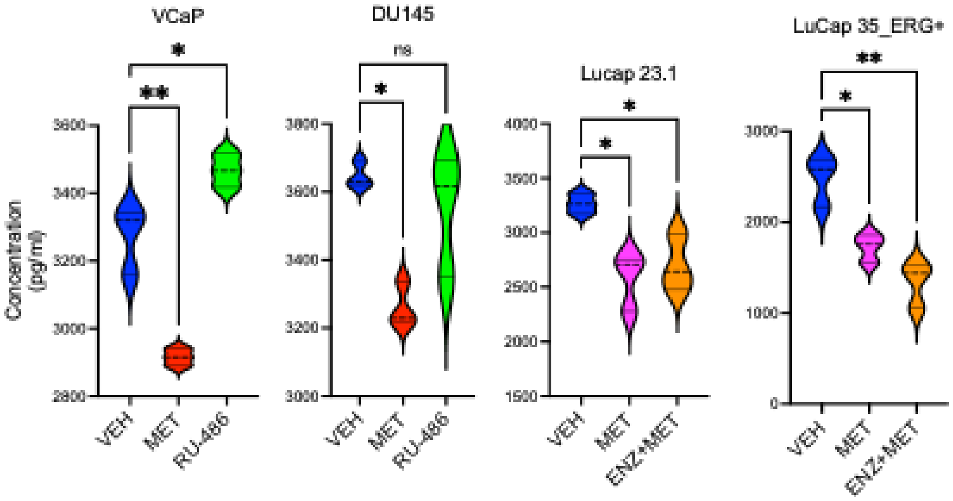

Figure XXIII: Determination of corticosterone levels in prostate cancer xenografts. Corticosterone concentrations (pg/mL) were measured in tumor tissues from xenografts of VCaP (vehicle, met, RU486), DU145 (vehicle, met, RU486), LuCaP 23.1 ERG^+ (vehicle, met, enzalutamide + met), and LuCaP 33 ERG^+ (vehicle, met, enzalutamide + met) xenograft models. Tumors were processed in parallel, and equal amounts of total protein were analyzed using the DetectX® Corticosterone Enzyme Immunoassay Kit (K014-H1) according to the manufacturer's instructions. Data are presented as violin plots with corticosterone concentration (pg/mL), with n = 3 tumors per group. Statistical significance is indicated: *, $p < 0.05$; **, $p < 0.01$ (**), n.s., not significant.

Minor points:

1. Fig.1 and 1S need more information to be clear.

For example, in Fig.1A and B, what do the bars represent? What are the green dashed lines?

In Fig.S1, the bar graph of the ERG IHC summary is missing. What's the unit for the y-axis in panel C?

Please provide the detailed writeup on the description of the Figure 1 and S1.

Note: below we separately aswer each sub-question.

In Fig.1A and B, what do the bars represent? What are the green dashed lines?

The Y-axis now indicates "Number of patients." The green dashed lines mark the mean expression level of each gene. These details have been added to the figure legend. Below we present the revised legend to Figures 1A and 1B:

In Fig.S1, the bar graph of the ERG IHC summary is missing.

ERG status in this study was determined by means of TMPRSS2-ERG fusion detection using whole exome sequencing, RNA sequencing, and clinical pathology reports rather than semi-quantitative IHC scoring. Therefore, IHC for ERG was used for representative validation of fusion status (original Figures S1A and S1B), but was not systematically quantified across the

cohort. In contrast, scoring IHC for GR was performed because GR expression levels rather than rearrangement status were the relevant variable for this analysis.

What's the unit for the y-axis in panel C?
The Y-axis unit is the semi-quantitative H-score.

2. Fig.2 shows that the GR and ERG interaction in HeLa cells may not reflect the relationship between ERG and GR in prostate cancer cells. Any in vitro data on prostrate cell line?
This comment is similar to comment 1 of Referee 2. Please refer to the corresponding response.

3. Fig.6 should involve enzalutamide treatment in the experiments.
We appreciate this suggestion. Unfortunately, we are unable to conduct additional animal experiments at this time due to constraints imposed by the night raid from Iran, which paralyzed our institutional animal facility. However, we believe that the existing data provides strong support for our hypothesis through: (i) three PDX model experiments demonstrating biological relevance, (ii) comprehensive in vitro enzalutamide treatment studies (as suggested by this reviewer in their earlier comments) establishing the underlying molecular mechanisms, and iii) transcription analyses from three PDX models, VCaP, and DU145 xenograft tumors that further validated our findings at the mRNA level. Together, these complementary approaches provide robust evidence without requiring additional animal studies.

4. Are the results of Fig. S3H and I reproducible in VCaP cells with endogenous ERG?
The results presented in the original forms of Figures S3H and S3I refer to DU145 cells overexpressing tERG (DU145-tERG#1 cells). These cells were treated with vehicle or RU486 for 48 hours and later treated with cycloheximide and/or MG132. To address the question raised by Referee 3, we re-performed the experiments using VCaP cells, which express an endogenous form of ERG. As shown below (Fig. XXIV) the results reported in original Figures S3H and S3I were, in general, reproducible in VCaP cells. Note that the new resuls were incorporated in the revised manuscript as Figure EV6H.

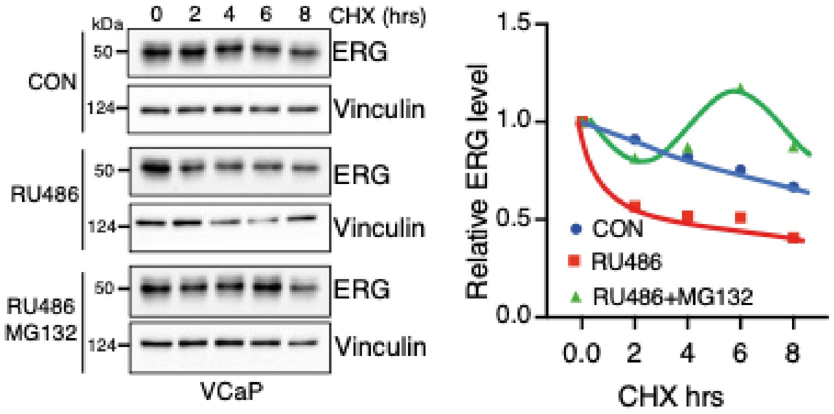

Figure XXIV: Inhibition of GR destabilizes ERG. VCaP cells were treated with vehicle or RU486 (10 µM) for 48 hours and then treated with cycloheximide (CHX; 50 µg/ml) and/or MG132 (25 µM). Cells were harvested at the indicated time points and extracts were prepared for immunoblotting. The signals observed in Western blots, quantified and normalized are shown in the line graph.

5. The discussion of the most recent clinical trial about the combination of ORIC-101 with enzalutamide, which did not produce clinical benefit in men with metastatic prostate cancer resistant to enzalutamide, is missing.

As requested, the revised manuscript contains the followint text: “Notably, because GR can circumvent AR inhibition and sustain tumor growth, targeting GR represents a potential strategy to restore sensitivity to anti-androgen therapy. Nevertheless, a clinical trial evaluating ORIC-101 an orally bioavailable small-molecule GR antagonist—in metastatic castration-resistant prostate cancer reported no meaningful antitumor activity in an unselected mCRPC population progressing on enzalutamide (Abida *et al*, 2024)”.

References

Abida W, Hahn AW, Shore N, Agarwal N, Sieber P, Smith MR, Dorff T, Monk P, Rettig M, Patel R *et al* (2024) Phase I Study of ORIC-101, a Glucocorticoid Receptor Antagonist, in Combination with Enzalutamide in Patients with Metastatic Castration-resistant Prostate Cancer Progressing on Enzalutamide. *Clin Cancer Res* 30: 1111-1120

Arora VK, Schenkein E, Murali R, Subudhi SK, Wongvipat J, Balbas MD, Shah N, Cai L, Efstathiou E, Logothetis C *et al* (2013) Glucocorticoid receptor confers resistance to antiandrogens by bypassing androgen receptor blockade. *Cell* 155: 1309-1322

Cai C, Wang H, Xu Y, Chen S, Balk SP (2009) Reactivation of androgen receptor-regulated TMPRSS2:ERG gene expression in castration-resistant prostate cancer. *Cancer Res* 69: 6027-6032

Cataldi S, Codini M, Cascianelli G, Tringali S, Tringali AR, Lazzarini A, Floridi A, Bartoccini E, Garcia-Gil M, Lazzarini R *et al* (2014) Nuclear lipid microdomain as resting place of dexamethasone to impair cell proliferation. *Int J Mol Sci* 15: 19832-19846

Hussain M, Daignault-Newton S, Twardowski PW, Albany C, Stein MN, Kunju LP, Siddiqui J, Wu YM, Robinson D, Lonigro RJ *et al* (2018) Targeting Androgen Receptor and DNA Repair in Metastatic Castration-Resistant Prostate Cancer: Results From NCI 9012. *J Clin Oncol* 36: 991-999

Karzai F, Walker SM, Wilkinson S, Madan RA, Shih JH, Merino MJ, Harmon SA, VanderWeele DJ, Cordes LM, Carrabba NV *et al* (2021) Sequential Prostate Magnetic Resonance Imaging in Newly Diagnosed High-risk Prostate Cancer Treated with Neoadjuvant Enzalutamide is Predictive of Therapeutic Response. *Clin Cancer Res* 27: 429-437

Sun C, Dobi A, Mohamed A, Li H, Thangapazham RL, Furusato B, Shaheduzzaman S, Tan SH, Vaidyanathan G, Whitman E *et al* (2008) TMPRSS2-ERG fusion, a common genomic alteration in prostate cancer activates C-MYC and abrogates prostate epithelial differentiation. *Oncogene* 27: 5348-5353

Wilkinson S, Ye H, Karzai F, Harmon SA, Terrigino NT, VanderWeele DJ, Bright JR, Atway R, Trostel SY, Carrabba NV *et al* (2021) Nascent Prostate Cancer Heterogeneity Drives Evolution and Resistance to Intense Hormonal Therapy. *Eur Urol* 80: 746-757

16th Feb 2026

Dear Prof. Yarden,

Thank you for the submission of your revised manuscript to EMBO Molecular Medicine. I am pleased to inform you that we will be able to accept your manuscript pending the following final amendments:

1) Please address referee #1 remaining concerns.
2) Authors:
- E-mail correspondence to Arunachalam Sekar, Aakanksha Verma and Haojie Huang could not be delivered. Please update the e-mail address and make sure to enter correct e-mail addresses for all authors in our submission system.
- We note name discrepancies in our submission system and in the manuscript. Rishita Chatterjee (manuscript text) vs Rishita Chattaerjee (submission system), Lipika R. Pal (manuscript text) vs Lipika Ray (submission system), Deepthi Ramesh-Kumar (manuscript text) vs Deepthi Ramesh Kumar (submission system), Alejändro A. Schaffer (manuscript) vs Alejandro Schäffer (submission system). Please correct.
3) Figures: During our routine image checks, we noticed that the blot images across the figure set appear pixelated under analysis. This is a common result of converting original 16-bit TIFF images to RGB format for publication, and while not a cause for concern, it can sometimes give the impression of image alteration to critical readers. To resolve this, please upload individual blot source data at the original captured higher resolution.
4) Source data: We note that the image source data are missing. Please provide completed checklist and upload source data as one (zipped) file per figure, combining the numerical, image and western blot source data.
5) In the main manuscript file, please do the following:
- Please address all comments suggested by our data editors listed below:
o Figure legends:
1. Please define the annotated p values ****/***/**/* as well as provide the exact p-values for the same in the legend of figure 5A, C; EV4 C, D as appropriate.
2. Please note that the exact p values are not provided in the legends of figures 2A, D, G; 4A-C; 6A, D; 7B, D; EV2 G, J, EV4 A, B; EV6 A, B, C, E.
3. Please indicate the statistical test used for data analysis in the legends of figures 4G, J; 5A, EV1 B, C; EV4 A, B, C, D, G.
4. Please note that the box plots need to be defined in terms of minima, maxima, centre, bounds of box and whiskers, and percentile in the legends of figures 1C, EV1 B, C.
5. Please note that information related to n is missing in the legends of figures 6C, D; 7A, C, E; EV4 A, C, D, G; EV6 C.
6. Please note that n=2 in figures 3C, 4E, 5B, EV3 A, EV5 B, E, F, G, I, K.
7. Please note that the error bars are not defined in the legends of figures EV2 G, J, EV4 A, B, C, D.
8. Please note that the scale bar is missing for figure 2B.
9. Please note that the yellow arrows are not defined in the legend of figure 2D. This needs to be rectified.
- In Methods, provide the antibody dilutions that were used for each antibody.
- In Methods, provide the statement that the experiments involving human subjects conformed to the principles set out in the WMA Declaration of Helsinki and the Department of Health and Human Services Belmont Report.
- In Methods, a statistical paragraph should reflect all information that you have filled in the Authors Checklist, especially regarding randomization, blinding, replication.
- Rename "Declaration of interests" to "Disclosure and competing interests statement". We updated our journal's competing interests policy in January 2022 and request authors to consider both actual and perceived competing interests. Please review the policy https://www.embopress.org/competing-interests and update your competing interests if necessary.
- Author contributions: Please remove it from the manuscript and specify author contributions in our submission system. CRediT has replaced the traditional author contributions section because it offers a systematic machine-readable author contributions format that allows for more effective research assessment. You are encouraged to use the free text boxes beneath each contributing author's name to add specific details on the author's contribution. More information is available in our guide to authors:
https://www.embopress.org/page/journal/17574684/authorguide#authorshipguidelines
- In expanded view figure legend please correct the nomenclature to "Figure EV1" etc.
- Indicate in legends number and nature of replicates and exact p= values, not a range, along with the statistical test used. To keep the figures "clear" some authors found providing an Appendix table Sx with all exact p-values preferable. You are welcome to do this if you want to.
- Please remove Reagents and Tools Table and upload it as a separate .docx file.
- Raw data from large-scale datasets (e.g. RNA seq) should be deposited in one of the relevant databases and made freely available prior the publication of the manuscript. Use the following format to report the accession number of your data:

The datasets produced in this study are available in the following databases:
[data type]: [full name of the resource] [accession number/identifier] ([doi or URL or identifiers.org/DATABASE:ACCESSION])

Please check "Author Guidelines" for more information.

https://www.embopress.org/page/journal/17574684/authorguide#availabilityofpublishedmaterial
6) Funding: Please ensure that the list in our system is complete and provide project numbers, if available, in both the Acknowledgements section and in our system.
7) Appendix: Please add page numbers to the table of contents.
8) The Paper Explained: Please provide "The Paper Explained" and add it to the main manuscript text. Please check "Author Guidelines" for more information. https://www.embopress.org/page/journal/17574684/authorguide#researcharticleguide
9) Synopsis: Every published paper now includes a 'Synopsis' to further enhance discoverability. Synopses are displayed on the journal webpage and are freely accessible to all readers. They include separate synopsis image and synopsis text.
- Synopsis image: Please provide a visual abstract as a high-resolution jpeg file 550 px-wide x 300-600 pixels high to illustrate your article.
- Synopsis text: Please provide a short standfirst (maximum of 300 characters, including space) as well as 2-5 one sentence bullet points that summarise the paper as a .doc file. Please write the bullet points to summarise the key NEW findings. They should be designed to be complementary to the abstract - i.e. not repeat the same text. We encourage inclusion of key acronyms and quantitative information (maximum of 30 words / bullet point). Please use the passive voice.
- Please check your synopsis text and image before submission with your revised manuscript. Please be aware that in the proof stage minor corrections only are allowed (e.g., typos).
10) As part of the EMBO Publications transparent editorial process (see our Editorial at http://embomolmed.embopress.org/content/2/9/329), EMBO Molecular Medicine will publish online a Review Process File (RPF) to accompany accepted manuscripts. This file will be published in conjunction with your paper and will include the anonymous referee reports, your point-by-point response and all pertinent correspondence relating to the manuscript. Let us know if you want to remove or not any figures from it prior to publication. Please note that the Authors checklist will be published at the end of the RPF.
11) Please provide a point-by-point letter INCLUDING my comments as well as the reviewer's reports and your detailed responses (as Word file).

I look forward to reading a new revised version of your manuscript as soon as possible.

Yours sincerely,

Zeljko Durdevic

Zeljko Durdevic
Senior Editor
EMBO Molecular Medicine

*** Instructions to submit your revised manuscript ***

*** PLEASE NOTE *** As part of the EMBO Publications transparent editorial process initiative (see our Editorial at https://www.embopress.org/doi/pdf/10.1002/emmm.201000094), EMBO Molecular Medicine will publish online a Review Process File to accompany accepted manuscripts.

In the event of acceptance, this file will be published in conjunction with your paper and will include the anonymous referee reports, your point-by-point response and all pertinent correspondence relating to the manuscript. If you do NOT want this file to be published, please inform the editorial office at contact@embomolmed.org.

*****

When preparing your revised manuscript, please refer to our guidelines: https://link.springer.com/journal/44321/submission-guidelines#cms-Revised-submissions. We perform an initial quality control of all revised manuscripts before re-review; failure to include requested items will delay the evaluation of your revision.

We require:

1) A .docx formatted version of the manuscript text (including legends for main figures, EV figures and tables). Please make sure

that the changes are highlighted to be clearly visible.

2) Individual production quality figure files as .eps, .tif, .jpg (one file per figure). For guidance, download the 'Figure Guide PDF': https://media.springernature.com/original/springer-cms/rest/v1/content/27825798/data/v1.

3) A .docx formatted letter INCLUDING the reviewers' reports and your detailed point-by-point responses to their comments. As part of the EMBO Press transparent editorial process, the point-by-point response is part of the Review Process File (RPF), which will be published alongside your paper.

4) A complete author checklist, which you can download from our author guidelines. Please insert information in the checklist that is also reflected in the manuscript. The completed author checklist will also be part of the RPF.

5) Please note that all corresponding authors are required to supply an ORCID ID for their name upon submission of a revised manuscript.

6) It is mandatory to include a 'Data Availability' section after the Materials and Methods. Before submitting your revision, primary datasets produced in this study need to be deposited in an appropriate public database, and the accession numbers and database listed under 'Data Availability'. Please remember to provide a reviewer password if the datasets are not yet public.

In case you have no data that requires deposition in a public database, please state so in this section. Note that the Data Availability Section is restricted to new primary data that are part of this study.

7) For data quantification: please specify the name of the statistical test used to generate error bars and P values, the number (n) of independent experiments (specify technical or biological replicates) underlying each data point and the test used to calculate p-values in each figure legend. The figure legends should contain a basic description of n, P and the test applied. Graphs must include a description of the bars and the error bars (s.d., s.e.m.).

8) At EMBO Press we ask authors to provide source data for the main manuscript figures. You will receive a separate email with instructions for providing source data with your revised manuscript, including how to upload and organize the files.

9) Our journal encourages inclusion of *data citations in the reference list* to directly cite datasets that were re-used and obtained from public databases. Data citations in the article text are distinct from normal bibliographical citations and should directly link to the database records from which the data can be accessed. In the main text, data citations are formatted as follows: "Data ref: Smith et al, 2001" or "Data ref: NCBI Sequence Read Archive PRJNA342805, 2017". In the Reference list, data citations must be labeled with "[DATASET]". A data reference must provide the database name, accession number/identifiers and a resolvable link to the landing page from which the data can be accessed at the end of the reference.

10) We replaced Supplementary Information with Expanded View (EV) Figures and Tables that are collapsible/expandable online. A maximum of 5 EV Figures can be typeset. EV Figures should be cited as 'Figure EV1, Figure EV2" etc... in the text and their respective legends should be included in the main text after the legends of regular figures.

- For the figures that you do NOT wish to display as Expanded View figures, they should be bundled together with their legends in a single PDF file called *Appendix*, which should start with a short Table of Content. Appendix figures should be referred to in the main text as: "Appendix Figure S1, Appendix Figure S2" etc.

- Additional Tables/Datasets should be labeled and referred to as Table EV1, Dataset EV1, etc. Legends have to be provided in a separate tab in case of .xls files. Alternatively, the legend can be supplied as a separate text file (README) and zipped together with the Table/Dataset file.

11) The paper explained: EMBO Molecular Medicine articles are accompanied by a summary of the articles to emphasize the major findings in the paper and their medical implications for the non-specialist reader. Please provide a draft summary of your article highlighting

- the medical issue you are addressing,

- the results obtained and

- their clinical impact.

This may be edited to ensure that readers understand the significance and context of the research. Please refer to any of our published articles for an example.

12) Author contributions: You will be asked to provide CRediT (Contributor Role Taxonomy) terms in the submission system.

These replace a narrative author contribution section in the manuscript.

13) A Conflict of Interest statement should be provided in the main text.

14) Every published paper includes a 'Synopsis' to further enhance discoverability. Synopses are displayed on the journal webpage and are freely accessible to all readers. They include a short stand first (maximum of 300 characters, including space) as well as 2-5 one-sentences bullet points that summarizes the paper. Please write the bullet points to summarize the key NEW findings. They should be designed to be complementary to the abstract - i.e. not repeat the same text. We encourage inclusion of key acronyms and quantitative information (maximum of 30 words / bullet point). Please use the passive voice. Please attach these in a separate file or send them by email, we will incorporate them accordingly.

Please also suggest a striking image or visual abstract to illustrate your article as a PNG file 550 px wide x 300-600 px high.

15) Include a Reagents and Tools Table as part of the Methods section, which can be downloaded from our author guidelines.

EMBO Molecular Medicine has a "scooping protection" policy, whereby similar findings that are published by others during review or revision are not a criterion for rejection. Should you decide to submit a revised version, I do ask that you get in touch after three months if you have not completed it, to update us on the status.

*Additional important information regarding Figures

Each figure should be given in a separate file and should have the following resolution:
Graphs 800-1,200 DPI
Photos 400-800 DPI
Colour (only CMYK) 300-400 DPI"

Figures are not edited by the production team. All lettering should be the same size and style; figure panels should be indicated by capital letters (A, B, C etc). Gridlines are not allowed except for log plots. Figures should be numbered in the order of their appearance in the text with Arabic numerals. Each Figure must have a separate legend and a caption is needed for each panel.

*Additional important information regarding figures and illustrations can be found at
https://media.springernature.com/original/springer-cms/rest/v1/content/27825798/data/v1

***** Reviewer's comments *****

Referee #1 (Remarks for Author):

The authors have substantially revised the manuscript and have addressed the major concerns raised in the first round of review in a thorough and convincing manner. In particular, they have strengthened the disease relevance by validating key GR-ERG interactions in prostate cancer cell lines, expanded the pharmacological analysis using highly selective GR antagonists and GR-knockout controls, and provided additional in vivo and transcriptomic evidence supporting their mechanistic model.
While the clinical cohorts remain modest in size and the conceptual advance is incremental, the revised manuscript now offers a strong combination of clinical anchoring, mechanistic depth, and translational relevance. As I noted in the first round of reviews, experiments in syngeneic models would significantly add to the potential impact of this study as the systemic GR inhibition in tERG+ PCa, which the data in this manuscript suggest, can markedly influence immune function; this raises important questions on the translational validity of this approach. In addition, the translational relevance and safety of combining GR blockade with ADT in these cases remain uncertain.
Minor points:
(1) "In contrast to ERG, GR displayed a unimodal pre-treatment distribution but, irrespective of ERG's status, GR's IHC increased substantially post treatment (Figs. 1A and 1B)," This is shown in Fig. EV1A, instead of Figs. 1A and 1B.
(2) "To initiate validation of this model, we performed dual-stain IHC (using anti-GR and anti-ERG antibodies) on serial sections from prostate tumors of 62 patients who underwent prostatectomy without prior hormonal therapy (Fig. EV2F)." It should be Fig. EV1F.
(3) The claims around 'prediction vs association' in patient cohorts should be carefully examined.

Referee #2 (Comments on Novelty/Model System for Author):

The TMPRSS2-ERG translocation is well known in the field of prostate cancer. Its link with androgen resistance has already

been documented in several articles. However, the work carried out here allows us to delve deeper into the molecular mechanisms involved.

Referee #2 (Remarks for Author):

The revisions answer the questions that were asked. I therefore consider the paper acceptable for publication.

Referee #3 (Comments on Novelty/Model System for Author):

The authors addressed all my concerns.

Title: *TMPRSS2-ERG confers resistance of prostate cancer to antiandrogens*
Authors: A. Sekar et al.
Second Rebuttal Letter

Referee #1 (Remarks for Author):

The authors have substantially revised the manuscript and have addressed the major concerns raised in the first round of review in a thorough and convincing manner. In particular, they have strengthened the disease relevance by validating key GR-ERG interactions in prostate cancer cell lines, expanded the pharmacological analysis using highly selective GR antagonists and GR-knockout controls, and provided additional in vivo and transcriptomic evidence supporting their mechanistic model.

We are glad that this referee found the 1st revision cycle to be both thorough and convincing. We are especially satisfied learning that the new version of the manuscript improved the disease relevance, as well as the mechanistic aspects of the study.

While the clinical cohorts remain modest in size and the conceptual advance is incremental, the revised manuscript now offers a strong combination of clinical anchoring, mechanistic depth, and translational relevance. As I noted in the first round of reviews, experiments in syngeneic models would significantly add to the potential impact of this study as the systemic GR inhibition in tERG+ PCa, which the data in this manuscript suggest, can markedly influence immune function; this raises important questions on the translational validity of this approach. In addition, the translational relevance and safety of combining GR blockade with ADT in these cases remain uncertain.

We concur that the manuscript would be significantly strengthened by incorporating additional (larger) cohorts of clinically relevant prostate cancer patients and by employing immunocompetent animal models. Unfortunately, despite considerable efforts, we were unable to identify appropriate patient cohorts or suitable syngeneic models. As the reviewer correctly noted, the translational implications and safety profile of combining GR antagonists and androgen deprivation therapy (ADT) warrant further investigation.

Minor points:

(1) "In contrast to ERG, GR displayed a unimodal pre-treatment distribution but, irrespective of ERG's status, GR's IHC increased substantially post treatment (Figs. 1A and 1B)," This is shown in Fig. EV1A, instead of Figs. 1A and 1B.

We thank the reviewer for drawing our attention to this error, which has been corrected in the revised manuscript.

(2) "To initiate validation of this model, we performed dual-stain IHC (using anti-GR and anti-ERG antibodies) on serial sections from prostate tumors of 62 patients who underwent prostatectomy without prior hormonal therapy (Fig. EV2F)." It should be Fig. EV1F.

We also thank the reviewer for drawing our attention to this additional error, which has been corrected in the revised manuscript.

(3) The claims around 'prediction vs association' in patient cohorts should be carefully examined.
As requested, we rewrote the respective part of the new manuscript version.

Referee #2 (Comments on Novelty/Model System for Author):

The TMPRSS2-ERG translocation is well known in the field of prostate cancer. Its link with androgen resistance has already been documented in several articles. However, the work carried out here allows us to delve deeper into the molecular mechanisms involved.

Referee #2 (Remarks for Author):

The revisions answer the questions that were asked. I therefore consider the paper acceptable for publication.

No further corrections or additions were made.

Referee #3 (Comments on Novelty/Model System for Author):

The authors addressed all my concerns.

No further corrections or additions were made.

"The Paper Explained" section has been added to the manuscript text, positioned between the Abstract and the Introduction.

7. Please upload the synopsis image in the size 550 pixels wide x 300 - 600 pixels high, which is the size in which synopsis images are published on our website. Please provide it in jpg or png format.

The synopsis image has been updated and uploaded in the required dimensions

24th Mar 2026

Dear Prof. Yarden,

We are pleased to inform you that your manuscript is accepted for publication and is now being sent to our publisher to be included in the next available issue of EMBO Molecular Medicine.

Your manuscript will be processed for publication by EMBO Press. It will be copy edited and you will receive page proofs prior to publication. Please note that you will be contacted by Springer Nature Author Services to complete licensing and payment information.

You may qualify for financial assistance for your publication charges - either via a Springer Nature fully open access agreement or an EMBO initiative. Check your eligibility: https://link.springer.com/journal/44321/how-to-publish-with-us

Should you be planning a Press Release on your article, please get in contact with embo_production@springernature.com as early as possible in order to coordinate publication and release dates.

If you have any questions, please do not hesitate to contact the Editorial Office. Thank you for your contribution to EMBO Molecular Medicine.

Yours sincerely,
Zeljko Durdevic

Zeljko Durdevic
Senior Editor
EMBO Molecular Medicine

>>> Please note that it is EMBO Molecular Medicine policy for the transcript of the editorial process (containing referee reports and your response letter) to be published as an online supplement to each paper. If you do NOT want this, you will need to inform the Editorial Office via email immediately. More information is available here: https://link.springer.com/partners/embo-press/editorial-policies#Peer%20review

EMBO Press Author Checklist

Corresponding Author Name: Yosef Yarden
Journal Submitted to: EMBO Molecular Medicine
Manuscript Number: EMM-2025-22016-V2

USEFUL LINKS FOR COMPLETING THIS FORM
The EMBO Journal - Author Guidelines
EMBO Reports - Author Guidelines
Molecular Systems Biology - Author Guidelines
EMBO Molecular Medicine - Author Guidelines

Reporting Checklist for Life Science Articles (updated January
This checklist is adapted from Materials Design Analysis Reporting (MDAR) Checklist for Authors. MDAR establishes a minimum set of requirements in transparent reporting in the life sciences (see Statement of Task: 10.31222/osf.io/9sm4x). Please follow the journal's guidelines in preparing your
Please note that a copy of this checklist will be published alongside your article.

Abridged guidelines for figures
1. Data
The data shown in figures should satisfy the following conditions:
- the data were obtained and processed according to the field's best practice and are presented to reflect the results of the experiments in an accurate and unbiased manner.
- ideally, figure panels should include only measurements that are directly comparable to each other and obtained with the same assay.
- plots include clearly labeled error bars for independent experiments and sample sizes. Unless justified, error bars should not be shown for technical
- if n<5, the individual data points from each experiment should be plotted. Any statistical test employed should be justified.
- Source Data should be included to report the data underlying figures according to the guidelines set out in the authorship guidelines on Data

2. Captions
Each figure caption should contain the following information, for each panel where they are relevant:
- a specification of the experimental system investigated (eg cell line, species name).
- the assay(s) and method(s) used to carry out the reported observations and measurements.
- an explicit mention of the biological and chemical entity(ies) that are being measured.
- an explicit mention of the biological and chemical entity(ies) that are altered/varied/perturbed in a controlled manner.
- the exact sample size (n) for each experimental group/condition, given as a number, not a range;
- a description of the sample collection allowing the reader to understand whether the samples represent technical or biological replicates (including how many animals, litters, cultures, etc.).
- a statement of how many times the experiment shown was independently replicated in the laboratory.
- definitions of statistical methods and measures:
  - common tests, such as t-test (please specify whether paired vs. unpaired), simple χ2 tests, Wilcoxon and Mann-Whitney tests, can be unambiguously identified by name only, but more complex techniques should be described in the methods section;
  - are tests one-sided or two-sided?
  - are there adjustments for multiple comparisons?
  - exact statistical test results, e.g., P values = x but not P values < x;
  - definition of 'center values' as median or average;
  - definition of error bars as s.d. or s.e.m.

Please complete ALL of the questions below.
Select "Not Applicable" only when the requested information is not relevant for your study.

Materials

Newly Created Materials	Information included in the manuscript?	In which section is the information available? (Reagents and Tools Table, Materials and Methods, Figures, Data Availability Section)
New materials and reagents need to be available; do any restrictions apply?	Not Applicable	

Antibodies	Information included in the manuscript?	In which section is the information available? (Reagents and Tools Table, Materials and Methods, Figures, Data Availability Section)
For **antibodies** provide the following information:<br>- Commercial antibodies: RRID (if possible) or supplier name, catalogue number and or/clone number<br>- Non-commercial: RRID or citation	Yes	Reagents and Tools Table

DNA and RNA sequences	Information included in the manuscript?	In which section is the information available? (Reagents and Tools Table, Materials and Methods, Figures, Data Availability Section)
**Short novel DNA or RNA including primers, probes**: provide the sequences.	Yes	Appendix Table S1

Cell materials	Information included in the manuscript?	In which section is the information available? (Reagents and Tools Table, Materials and Methods, Figures, Data Availability Section)
**Cell lines:** Provide species information, strain. Provide accession number in repository **OR** supplier name, catalog number, clone number, and/**OR** RRID.	Yes	Reagents and Tools Table
**Primary cultures:** Provide species, strain, sex of origin, genetic modification status.	Not Applicable	
Report if the cell lines were recently **authenticated** (e.g., by STR profiling) and tested for mycoplasma contamination.	Yes	Materials and Methods

Experimental animals	Information included in the manuscript?	In which section is the information available? (Reagents and Tools Table, Materials and Methods, Figures, Data Availability Section)
**Laboratory animals or Model organisms:** Provide species, strain, sex, age, genetic modification status. Provide accession number in repository **OR** supplier name, catalog number, clone number, **OR** RRID.	Yes	Reagents and Tools Table
**Animal observed in or captured from the field:** Provide species, sex, and age where possible.	Not Applicable	
Please detail **housing and husbandry conditions**.	Yes	Materials and Methods

Plants and microbes	Information included in the manuscript?	In which section is the information available? (Reagents and Tools Table, Materials and Methods, Figures, Data Availability Section)
**Plants:** provide species and strain, ecotype and cultivar where relevant, unique accession number if available, and source (including location for collected wild specimens).	Not Applicable	
**Microbes:** provide species and strain, unique accession number if available, and source.	Not Applicable	

Human research participants	Information included in the manuscript?	In which section is the information available? (Reagents and Tools Table, Materials and Methods, Figures, Data Availability Section)
If collected and within the bounds of privacy constraints report on age, sex and gender or ethnicity for all study participants.	Not Applicable	

Core facilities	Information included in the manuscript?	In which section is the information available? (Reagents and Tools Table, Materials and Methods, Figures, Data Availability Section)
If your work benefited from core facilities, was their service mentioned in the acknowledgments section?	Not Applicable	

Design

Study protocol	Information included in the manuscript?	In which section is the information available? (Reagents and Tools Table, Materials and Methods, Figures, Data Availability Section)
If study protocol has been **pre-registered, provide DOI in the manuscript**. For clinical trials, provide the trial registration number **OR** cite DOI.	Not Applicable	
Report the **clinical trial registration number** (at ClinicalTrials.gov or equivalent), where applicable.	Not Applicable	

Laboratory protocol	Information included in the manuscript?	In which section is the information available? (Reagents and Tools Table, Materials and Methods, Figures, Data Availability Section)
Provide DOI OR other citation details if **external detailed step-by-step protocols** are available.	Not Applicable	

Experimental study design and statistics	Information included in the manuscript?	In which section is the information available? (Reagents and Tools Table, Materials and Methods, Figures, Data Availability Section)
Include a statement about **sample size** estimate even if no statistical methods were used.	Yes	Figures
Were any steps taken to minimize the effects of subjective bias when allocating animals/samples to treatment (e.g. **randomization procedure**)? If yes, have they been described?	Not Applicable	
Include a statement about **blinding** even if no blinding was done.	Not Applicable	
Describe **inclusion/exclusion criteria** if samples or animals were excluded from the analysis. Were the criteria pre-established?<br><br>If sample or data points were omitted from analysis, report if this was due to attrition or intentional exclusion and provide justification.	Not Applicable	
For every figure, are **statistical tests** justified as appropriate? Do the data meet the assumptions of the tests (e.g., normal distribution)? Describe any methods used to assess it. Is there an estimate of variation within each group of data? Is the variance similar between the groups that are being statistically compared?	Yes	Figures

Sample definition and in-laboratory replication	Information included in the manuscript?	In which section is the information available? (Reagents and Tools Table, Materials and Methods, Figures, Data Availability Section)
In the figure legends: state number of times the experiment was **replicated** in laboratory.	Yes	Figure legends
In the figure legends: define whether data describe **technical or biological replicates**.	Yes	Figure legends

Ethics

Ethics	Information included in the manuscript?	In which section is the information available? (Reagents and Tools Table, Materials and Methods, Figures, Data Availability Section)
Studies involving **human participants**: State details of **authority granting ethics approval** (IRB or equivalent committee(s), provide reference number for approval.	Not Applicable	
Studies involving **human participants**: Include a statement confirming that **informed consent** was obtained from all subjects and that the experiments conformed to the principles set out in the WMA Declaration of Helsinki and the Department of Health and Human Services Belmont Report.	Yes	Materials and Methods
Studies involving **human participants: For publication of patient photos**, include a statement confirming that consent to publish was obtained.	Not Applicable	
Studies involving experimental **animals**: State details of **authority granting ethics approval** (IRB or equivalent committee(s), provide reference number for approval. Include a statement of compliance with ethical regulations.	Yes	Materials and Methods
Studies involving **specimen and field samples:** State if relevant **permits** obtained, provide details of authority approving study; if none were required, explain why.	Not Applicable	

Dual Use Research of Concern (DURC)	Information included in the manuscript?	In which section is the information available? (Reagents and Tools Table, Materials and Methods, Figures, Data Availability Section)
Could your study fall under dual use research restrictions? Please check biosecurity documents and list of **select agents and toxins** (CDC): https://www.selectagents.gov/sat/list.htm	Not Applicable	
If you used a select agent, is the security level of the lab appropriate and reported in the manuscript?	Not Applicable	
If a study is subject to dual use research of concern regulations, is the name of the **authority granting approval and reference number** for the regulatory approval provided in the manuscript?	Not Applicable	

Reporting

The MDAR framework recommends adoption of discipline-specific guidelines, established and endorsed through community initiatives. Journals have their own policy about requiring specific guidelines and recommendations to complement MDAR.

Adherence to community standards	Information included in the manuscript?	In which section is the information available? (Reagents and Tools Table, Materials and Methods, Figures, Data Availability Section)
State if relevant guidelines or checklists (e.g., **ICMJE, MIBBI, ARRIVE, PRISMA**) have been followed or provided.	Not Applicable	
For **tumor marker prognostic studies,** we recommend that you follow the **REMARK** reporting guidelines (see link list at top right). See author guidelines, under 'Reporting Guidelines'. Please confirm you have followed these guidelines.	Not Applicable	
For **phase II and III randomized controlled trials**, please refer to the **CONSORT** flow diagram (see link list at top right) and submit the CONSORT checklist (see link list at top right) with your submission. See author guidelines, under 'Reporting Guidelines'. Please confirm you have submitted this list.	Not Applicable	

Data Availability

Data availability	Information included in the manuscript?	In which section is the information available? (Reagents and Tools Table, Materials and Methods, Figures, Data Availability Section)
Have **primary datasets** been deposited according to the journal's guidelines (see 'Data Deposition' section) and the respective accession numbers provided in the Data Availability Section?	Yes	Data Availability Section
Were **human clinical and genomic datasets** deposited in a public access-controlled repository in accordance to ethical obligations to the patients and to the applicable consent agreement?	Not Applicable	
Are **computational models** that are central and integral to a study available without restrictions in a machine-readable form? Were the relevant accession numbers or links provided?	Not Applicable	
If publicly available data were reused, provide the respective **data citations in the reference list**.	Yes	Figure legends and Reference
